# Access to Pyrazolo[1,5‑*a*]pyrimidinone Regioisomers from Acylated Meldrum’s Acids

**DOI:** 10.1021/acs.orglett.5c04435

**Published:** 2025-12-22

**Authors:** Maxime Donzel, Erik Chorell

**Affiliations:** Department of Chemistry, 8075 Umeå University, SE 901 87 Umeå, Sweden

## Abstract

Two complementary and efficient methods for the regioselective synthesis of functionalized pyrazolo­[1,5-*a*]­pyrimidinones were developed from 3-aminopyrazoles and acylated Meldrum’s acids. Fine-tuning the reaction conditions enables selective access to either pyrazolo­[1,5-*a*]­pyrimidin-5-ones or -7-ones in high yields. This protocol offers a reliable route to pyrazolo­[1,5-*a*]­pyrimidin-5-ones, a subclass with few reported syntheses, and highlights the value of acylated Meldrum’s acids as building blocks for regioselective heterocycle synthesis and biologically relevant scaffold generation.

## Graphical abstract

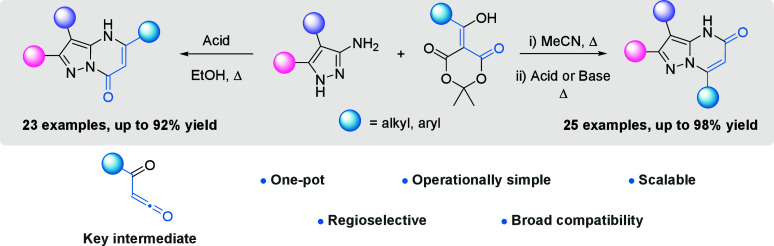

The pyrazolo­[1,5-*a*]­pyrimidine (PP) scaffold has attracted a considerable amount of interest in recent years, with numerous reports on both its synthetic accessibility and its medicinal chemistry applications.^1^ This scaffold is found in approved drugs, such as zaleplon, and in drug candidates targeting cancer,^2^ diabetes,^3^ and microbial,^4^ viral,^5^ or parasitic infections.^6^ Reflecting this biological relevance, diverse synthetic strategies have been reported to access^7^ and selectively functionalize PPs.^8^ Within this family, two regioisomeric subclasses can be distinguished: pyrazolo­[1,5-*a*]­pyrimidin-7­(4*H*)-ones (PP-7-Os) and pyrazolo­[1,5-*a*]­pyrimidin-5­(4*H*)-ones (PP-5-Os) (Figure 1A). The PP-7-O scaffold is well-established, with numerous syntheses reported over the past several decades. The most common method involves condensing 3-aminopyrazoles with β-ketoesters to afford substituted PP-7-Os in good yields (Figure 1B). First described in 1981, this reaction is typically performed in boiling acetic acid, which serves as both the solvent and the acid catalyst.^9^ PP-7-Os have been reported with multiple applications in the field of medicinal chemistry as potential anticancer,^10^ antibacterial,^11^ or antidiabetic^12^ agents. In contrast, PP-5-Os remain underexplored, with few synthetic reports. The regioselective synthesis of an unsubstituted PP-5-O was described in 2007 from 3-aminopyrazole and 1,3-dimethyluracil.^13^ Previously, Remmingler had reported a similar cyclocondensation with ethyl phenylpropiolate, though in low yield and with limited data.^14^ Arbabri later improved this approach using activated alkynes, giving phenyl- and methyl-substituted PP-5-Os,^15^ and extended it to 7-trifluoromethyl derivatives.^16^ A related two-pot Sonogashira/cyclocondensation route to 7-heteroaryl-PP-5-Os was also developed, albeit with low yields (Figure 1C).^17^ These challenges have limited access to PP-5-Os, though their frequent appearance in patents underscores their pharmaceutical potential.^18^

**Figure 1:**
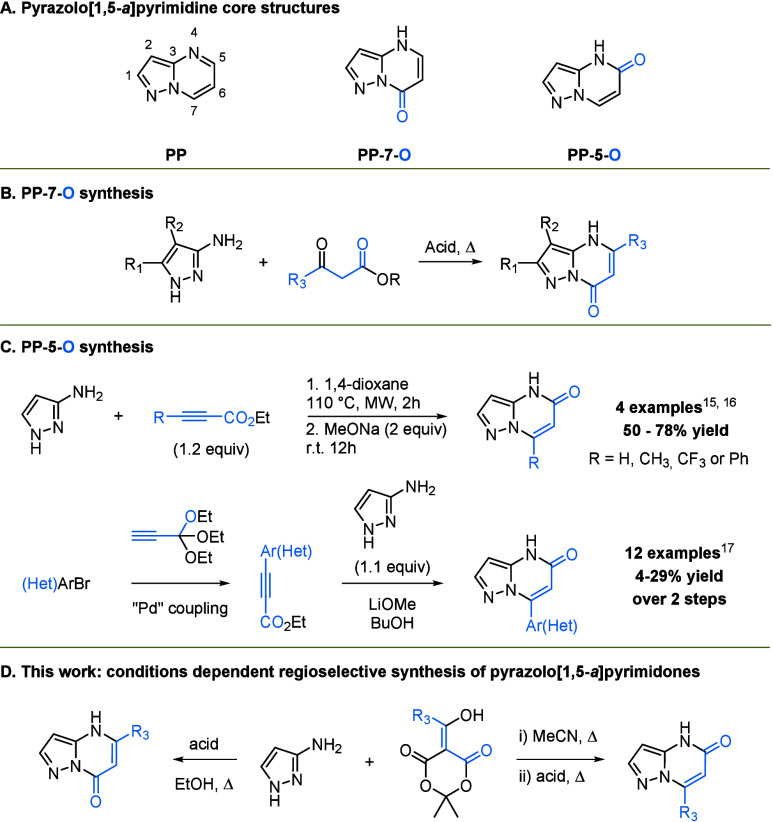
(A) Pyrazolo­[1,5-*a*]­pyrimidine core structures. (B) PP-7-O synthesis. (C) PP-5-O synthesis. (D) This work.

Herein, we report two straightforward and high-yielding one-pot synthetic routes that enable the regioselective access to both 5-substituted pyrazolo­[1,5-*a*]­pyrimidin-7­(4*H*)-ones (PP-7-Os) and 7-substituted pyrazolo­[1,5-*a*]­pyrimidin-5­(4*H*)-ones (PP-5-Os) from the same 3-aminopyrazole/acylated Meldrum’s acid building blocks (Figure 1D).

In our attempt to access isomers of both PP-5-Os and PP-7-Os, we identified acylated Meldrum’s acids as ideal starting materials. PP-7-Os are typically obtained by cyclocondensation of 3-aminopyrazoles with β-ketoesters, but the limited commercial availability of these intermediates often requires an in-house synthesis. In contrast, acylated Meldrum’s acids are readily prepared in one step^19^ and thermally decompose in ethanol through transient ketene intermediate **I** to release β-ketoesters.^20^ We therefore envisioned a one-pot process in which the β-ketoester, generated in situ, would react with 3-aminopyrazole to afford the desired PP-7-O **3** (Scheme 1, path A). In a non-nucleophilic solvent, we anticipated that reactive ketene intermediate **I** would form upon heating, as previously demonstrated,^21^ and react with the 3-aminopyrazole to give intermediate **4**. Previous studies have reported amine,^22^ amide,^23^ and 3-aminopyrazole^24^ additions to ketenes generated from acylated Meldrum’s acids. One similar intermediate was shown to undergo intramolecular cyclization under acidic conditions. However, in that case, it was synthesized through several successive steps.^25^ We envisioned generating this intermediate directly from acylated Meldrum’s acid **2**, enabling intracyclization to substituted PP-5-Os **5** (Scheme 1, path B). Unknown to us at the start of this project, a related patent described a similar concept, but it lacked experimental details and scope exploration and required a two-pot sequence,^26^ prompting us to investigate and optimize the process.

**Scheme 1:**
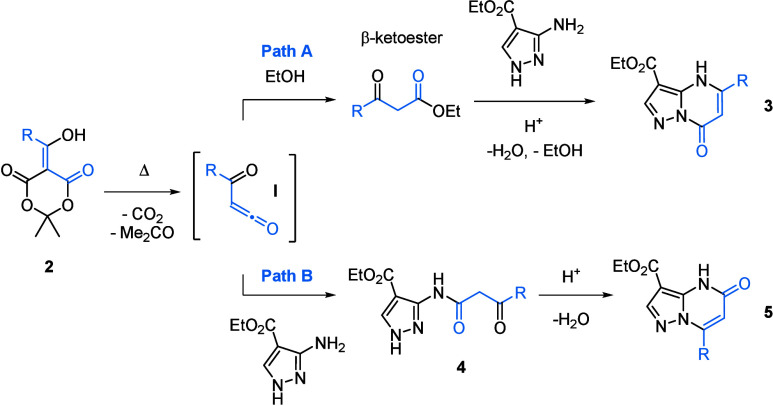
Synthetic Pathways to PP-5-Os and PP-7-Os

We first investigated the possibility of forming the β-ketoester intermediate from acylated Meldrum’s acid **2a** (1.5 equiv), followed by direct cyclization with aminopyrazole **1a** to obtain target PP-7-O regioisomer **3a**. When both starting materials were stirred in ethanol for 16 h at 80 °C in a sealed tube, less than 10% conversion to **3a** was observed, along with unreacted **1a** and the expected β-ketoester intermediate (Table 1, entry 1). As β-ketoester condensation with aminopyrazoles has often been performed in acetic acid at high temperatures,^9^ 10 equiv of acetic acid was added. This resulted in the formation of regioisomer **3a** in 52% yield; however, full consumption of **1** was not achieved (Table 1, entry 2). Replacing acetic acid with only 1 equiv of TFA improved the reaction, affording **3a** in 86% yield, and simultaneously reduced the amount of **2a** to only 1.2 equiv (Table 1, entry 3).

**Table 1:**
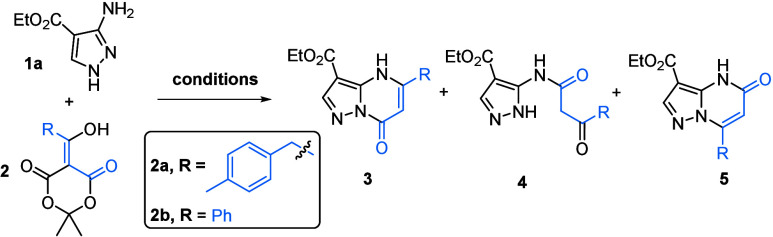
Optimization of the Reaction Conditions

entry	**2** (equiv)	conditions^a^	**3**:**4**:**5** ratio^b^	yield (**3**/**4**/**5**) (%)^c^
1	**2a**, 1.5	EtOH (80 °C, 16 h)	1:0:0	<10/–/–
2	**2a**, 1.5	EtOH, AcOH (10 equiv, 80 °C, 16 h)	1:0:0	55/–/–
3	**2a**, 1.2	EtOH, TFA (1 equiv, 80 °C, 16 h)	1:0:0	85/–/–
4	**2a**, 1.5	DCE, TFA (5 equiv, 80 °C, 16 h)	19:0:81	nd^a^/–/63
5	**2a**, 1.5	MeCN (80 °C, 16 h)	0:62:38	–/46/35
6	**2a**, 1.5	(i) MeCN (80 °C, 1 h)	0:0:1	–/–/86
		(ii) TFA (1 equiv, 80 °C, 16 h)		
7	**2b**, 1.5	(i) MeCN (80 °C, 1 h)	0:65:35	–/50/12
		(ii) TFA (1 equiv, 80 °C, 16 h)		
8	**2b**, 1.5	(i) MeCN (80 °C, 1 h)	0:0:1	–/–/75
		(ii) *p*-TsOH (1.5 equiv, 80 °C, 16 h)		
9	**2a**, 1.5	EtOH, *p-*TsOH (1 equiv, 80 °C, 16 h)	1:0:0	91/–/–
10	**2a**, 1.5	(i) MeCN (80 °C, 1 h)	0:0:1	–/–/94
		(ii) *p*-TsOH (1 equiv, 80 °C, 16 h)		

a Reaction conditions: aminopyrazole **1** (0.25 mmol), acylated Meldrum’s acid **2** (1.2–1.5 equiv), acid (0–10 equiv), solvent (1 mL, 0.25 M), 80 °C, sealed tube, indicated time. For two-step reactions, the acid was added directly to the reaction mixture.

b Ratio from crude ^1^H NMR.

c Isolated yield.

d Not determined.

In parallel, we examined the conditions to selectively form PP-5-O regioisomer **5a**. Initial attempts started by combining the reagents in dichloroethane with TFA and gave a mixture of regioisomers in a 2:8 ratio, in favor of **5a**, with a 63% isolated yield (Table 1, entry 4). When the acid was added at the beginning of the reaction, complete regioselectivity in favor of **5a** over **3a** was never achieved. In acetonitrile, without acid, **4a** and **5a** were obtained exclusively, with no **3a** detected, but conversion of **4a** to **5a** remained incomplete after 16 h at 80 °C (Table 1, entry 5). These results prompted us to adopt a stepwise strategy to ensure full conversion and preserve complete regioselectivity. First, **1a** and **2a** were reacted in acetonitrile for 1 h at 80 °C, and then, upon formation of **4a** and full consumption of **1a**, TFA (1 equiv) was added to induce cyclization and gave **5a** exclusively after 16 h at 80 °C (86%, Table 1, entry 6). However, when expanded to aryl-substituted acylated Meldrum’s acid **2b** to access **5b**, TFA proved to be inefficient, giving intermediate **4b** as the main product (Table 1, entry 7). *p*-Toluenesulfonic acid (*p*-TsOH, 1.5 equiv) proved to be a more efficient reagent and gave the desired **5b** in satisfactory yield (75%) (Table 1, entry 8). Finally, we found that 1 equiv of *p*-TsOH was also sufficient to produce both **3a** and **5a** in excellent yields and selectivity (Table 1, entries 9 and 10, respectively). Finally, to verify the proposed pathway, the β-ketoester formed from **2a** and β-ketoamide **4a** were isolated and subjected to the optimized conditions, successfully affording **3a** and **5a**, respectively, in high yield (Scheme S1).

With the optimized conditions in hand, we next investigated the scope of the reaction for the formation of pyrazolo­[1,5-*a*]­pyrimidin-7-ones (Scheme 2). **3a** was first synthesized in 81% yield on a 2.5 mmol scale. At this scale, the pure adduct could be obtained analytically after precipitation from the reaction mixture. We next investigated the substitution pattern tolerated on starting acylated Meldrum’s acid **2**, using **1a** as the model aminopyrazole. Arylated substrates were obtained in good yield, although complete conversion was only achieved upon increasing the amount of **2** to 1.5 equiv (72–87%, **3b**–**g**). Both the electron-donating group and the electron-withdrawing group were well tolerated with a similar reactivity, including the hindered *ortho*-brominated aryl adduct (87%, **3g**). As observed for the arylated derivatives, while ethyl-substituted **3h** was obtained in 77% yield under the standard conditions, with secondary alkyl adducts, full conversion and good yields of **3i** and **3j** could be obtained only with 1.5 equiv of **2** (90% and 84% yields, respectively). We then conducted experiments with a broad range of alkyl adducts. The reaction proved to be compatible and afforded good to excellent yields with sterically hindered groups (84–90%, **3i**–**k**), a thiophene (87%, **3m**), a terminal alkene (75%, **3m**), a terminal alkyne (77%, **3o**), and highly valuable motifs such as an ethyl chloride group (86%, **3p**) or a benzyl-protected alcohol (79%, **3q**). In parallel, we explored diversification of the substitution pattern of starting aminopyrazole **1**. Unsubstituted 3-aminopyrazole underwent cyclocondensation to afford a 69% yield (**3r**). The 2-cyano-substituted derivative was obtained in high yield (88%, **3u**), and substitution at position 1 of the pyrazole ring with a *tert*-butyl (92%, **3t**), a phenyl (70%, **3v**), and an ethyl ester (81%, **3w**) also showed excellent compatibility under the optimized conditions. However, 3-amino-4-bromopyrazole led to complete decomposition with no trace of product **3s**. Likewise, N-alkylated products **3x** and **3y** were not formed. We next investigated the substrate scope of pyrazolo­[1,5-*a*]­pyrimidin-5-ones using the same starting materials **1** and **2** (Scheme 2). As previously shown for the other regioisomer, the synthesis of analytically pure **5a** could be scaled up to 2.5 mmol, affording **5a** in very good yield (85%) by simple filtration of the precipitate. With arylated Meldrum’s acid derivatives, using 1.5 equiv of *p*-TsOH, good yields were obtained with the phenyl (75%, **5b**) and electro-donating groups such as *p*-OMe (87%, **5d**) and *p*-Me (78%, **5f**). Electron-withdrawing substituents were also tolerated but gave lower yields, as observed for *p*-F (60%, **5c**) and *p*-CF_3_ (50%, **5e**) derivatives. Unexpectedly, the *ortho*-brominated adduct underwent complete degradation under the standard ring-closing conditions (**5g**). Alkylated acyl-Meldrum’s acids were well tolerated and afforded excellent yields. For instance, target compounds containing ethyl (**5h**), isopropyl (**5i**), 1-phenylethyl (**5j**), methyl-naphthyl (**5k**), thiophene (**5m**), terminal alkene (**5n**), and terminal alkyne (**5o**) were all obtained in yields exceeding 90%. Chloromethyl adduct **5p** was obtained in excellent yield (89%) on a small scale and in good yield (76%) on a 2.5 mmol scale. Benzylated alcohol **5q** was also obtained in good yield (73%). When diversifying aminopyrazoles **1**, 1-*tert*-butyl (87%, **5t**), 2-cyano (98%, **5u**), 1-phenyl (76%, **5v**), and 1-ethyl ester (84%, **5w**) analogues displayed excellent compatibility under the optimized conditions. In contrast, nonsubstituted (**5r**) and bromo-substituted (**5s**) analogues degraded under the standard conditions. Using TFA instead of *p*-TsOH afforded **5r** and **5s** in 67% and 58% yields, respectively. Notably, ≈30% of debrominated **5r** was isolated, suggesting the acid-promoted debromination of **5s**. Disubstitution of the amine was well tolerated in the PP-5-O regioisomer pathway, and *N*-benzylated adduct **5x** was obtained in 88% yield. However, for the *N*-methylated pyrazole, product **5y** was not observed.

**Scheme 2:**
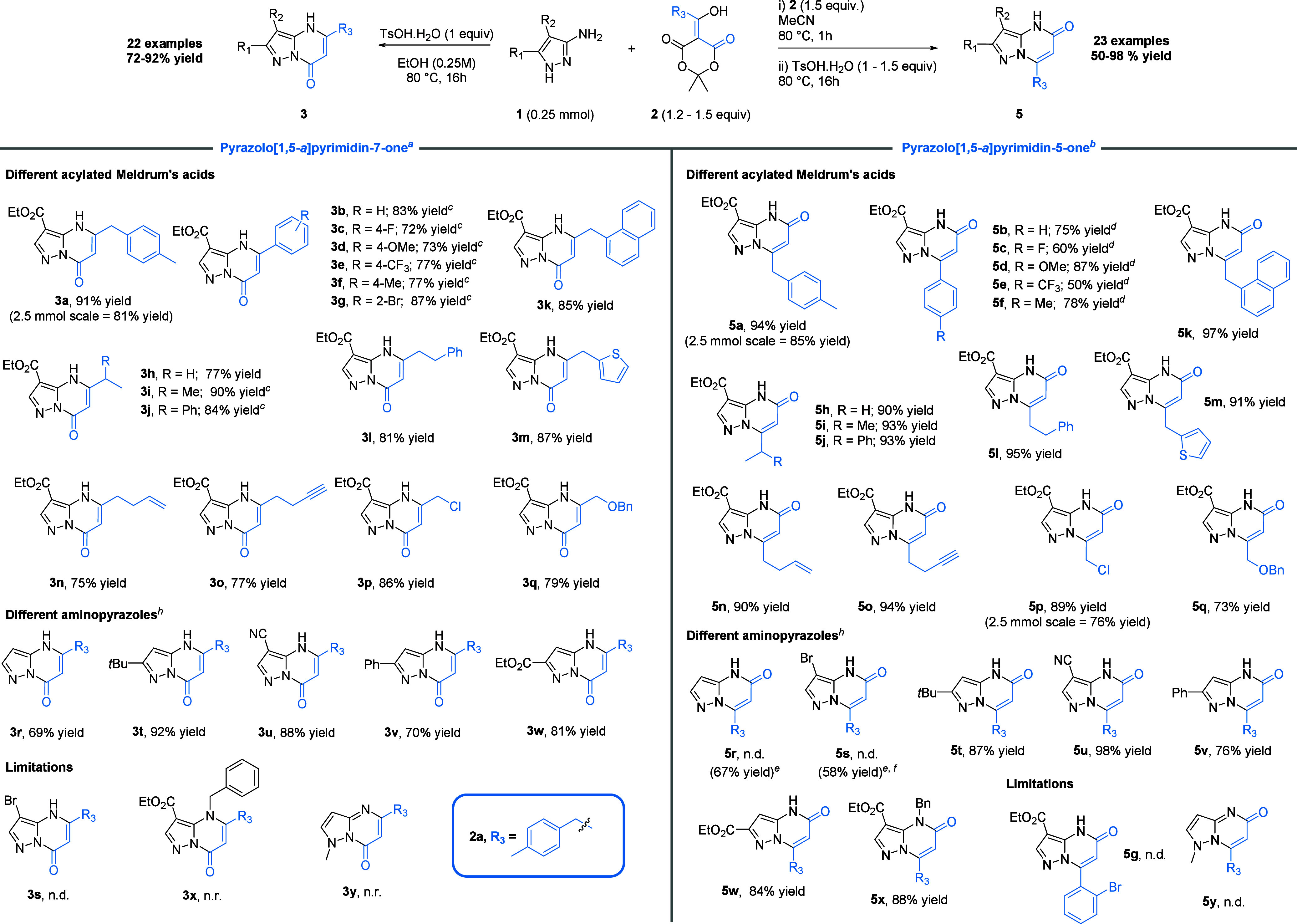
Substrate Scope

a Conditions: **1** (0.25 mmol), **2** (1.2 equiv), and TsOH·H_2_O (1 equiv) in EtOH (0.25 M) at 80 °C for 16 h in a sealed tube.
b Conditions: (i) **1** (0.25 mmol) and **2** (1.5 equiv) in MeCN (0.25 M) at 80 °C for 1 h and (ii) *p*-TsOH (1 equiv) at 80 °C for 16 h in a sealed tube.
c
**2** (1.5 equiv).
d
*p*-TsOH (1.5 equiv).
e TFA (1 equiv) instead of *p*-TsOH.
f Debrominated **5r** isolated in 33% yield.
h
**2** = **2a**. n.d., not detected; n.r., no reaction.

We next explored the introduction of a piperidine substituent into both regioisomers, which is a common motif in biologically relevant pyrazolo­[1,5-*a*]­pyrimidinones.^26,27^ Acylated Meldrum’s acid **6** was treated under the previously developed conditions using 2 equiv of acid to ensure complete Boc deprotection, affording salts **7** and **9** in good yields (67% and 86%, respectively) after filtration. From a synthetic perspective, retaining the Boc group was advantageous and was achieved by replacing the acid with DIPEA. Compound **10** was obtained in 79% yield (Scheme 3A), showing that the method is compatible with acid-sensitive substrates. However, attempts to prepare isomer **8** in a good yield were unsuccessful. DIPEA could also be used successfully to obtain model substrate **5a** in 71% yield; however, it was unable to circumvent the limitations that we observed in the overall reaction scope (Scheme S2).

**Scheme 3:**
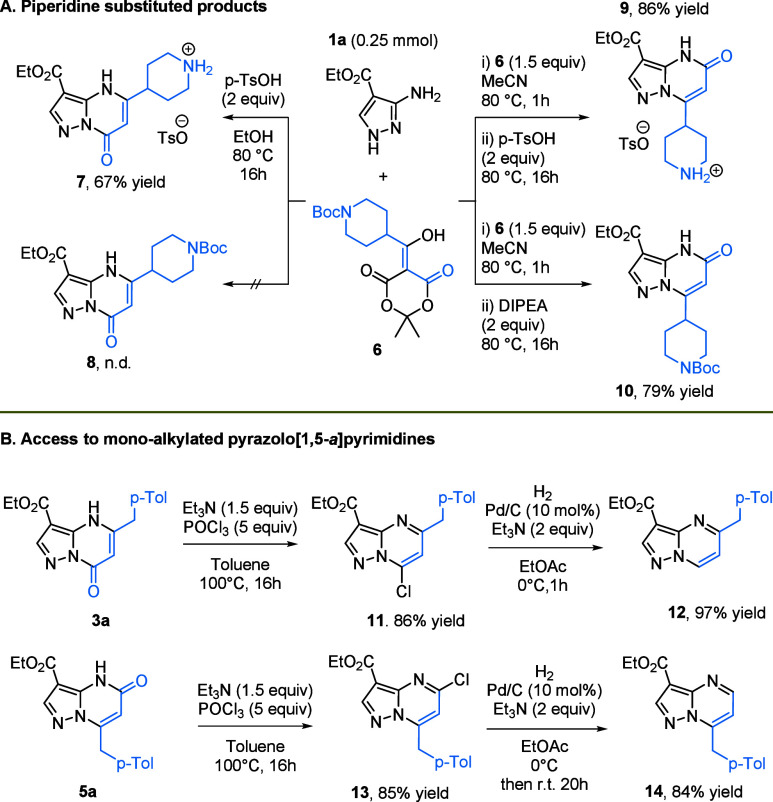
Piperidine Introduction and Synthetic Utility

We also aimed to demonstrate that pyrazolo­[1,5-*a*]­pyrimidinones can serve as useful intermediates for the preparation of substituted pyrazolo­[1,5-*a*]­pyrimidines. We performed the chlorination of **3a** and **5a**, affording derivatives **11** and **13** in 86% and 87% yields, respectively. Chlorinated PPs can be further used in SNAr reactions with nucleophiles, such as amines^28^ and thiols,^29^ or Pd-catalyzed cross-coupling,^30^ to provide disubstituted PPs. To broaden the scope, we investigated dechlorination as a route to monofunctionalized PPs. While dechlorination of 7-chloropyrazolopyrimidines has been reported with moderate yields,^31^ no examples of 7-substituted 5-chloropyrazolopyrimidines like **13** have been described. We applied and adapted the hydrodechlorination conditions developed by Sajiki’s group, using palladium on carbon and triethylamine.^32^ We found that in ethyl acetate at 0 °C under a hydrogen atmosphere, the palladium-catalyzed hydrodechlorination of compound **11** to PP **12** proceeded smoothly within 1 h. This gave the desired product in 97% yield without any detectable over-reduction of the pyrimidine ring, an issue reported in protic solvents.^33^ When applied to compound **13**, the reaction required 20 h at room temperature to reach full conversion, affording product **14** in a good 84% yield (Scheme 3B). Overall, these transformations provide a mild and selective approach to monofunctionalized PPs, further expanding the synthetic utility of PP-O scaffolds.

In summary, we have developed two complementary and robust methods for the regioselective synthesis of functionalized pyrazolo­[1,5-*a*]­pyrimidinones from 3-aminopyrazoles and acylated Meldrum’s acids. Fine-tuning of the reaction conditions afforded both pyrazolo­[1,5-*a*]­pyrimidin-5- and -7-ones in high yields with excellent selectivity. These methods provide reliable access to pyrazolo­[1,5-*a*]­pyrimidin-5-ones, a subclass previously lacking robust synthetic routes. Their simplicity, scalability, and compatibility with diverse substrates make them valuable tools for the generation of biologically relevant derivatives and versatile intermediates. This work also underscores the potential of acylated Meldrum’s acids as efficient building blocks for regioselective functionalization and heterocycle synthesis.

## Supplementary Material



## Data Availability

The data underlying this study are available in the published article and its Supporting Information.

## Notes

Experimental procedures, NMR (^1^H and ^13^C) spectra for all compounds, and HRMS data for the final compounds (PDF)
