# Supplementary material for: Access to Pyrazolo[1,5‑a]pyrimidinone Regioisomers from Acylated Meldrum’s Acids
Source: Org Lett. 2025 Dec 22;28(1):157–62. doi: 10.1021/acs.orglett.5c04435 (PMC12797321; doi:10.1021/acs.orglett.5c04435)
Supplement: Supplementary file 1 [file ol5c04435_si_001.pdf]

Supporting Information

Access to Pyrazolo[1,5-a]pyrimidinone Regioisomers from Acylated Meldrum's Acids

Maxime Donzel*, Erik Chorell*

Department of Chemistry, Umeå University, 901 87, Umeå, Sweden

Table of Contents

General Experimental 3

General procedure for optimization 3

Table S1. Optimization Table for Regioselective Cyclisation 3

Scheme S1. 2-pots-2-steps synthesis of pyrazolo[1,5-*a*]pyrimidinones 3a and 5a. 4

Scheme S2. Scope of DIPEA assisted synthesis of pyrazolo[1,5-*a*]pyrimidin-5-ones 5. 4

2-Aminopyrazoles 1 synthesis 5

Acylated Meldrum's acids synthesis 6

Pyrazolo[1,5-*a*]pyrimidin-7-ones 3 synthesis 12

Pyrazolo[1,5-*a*]pyrimidin-5-ones 5 synthesis 18

Nitrogen containing pyrazolo[1,5-*a*]-pyrimidones synthesis. 24

Mono-alkylated pyrazolo[1,5-*a*]pyrimidines synthesis. 25

Intermediates 2a' and 4a synthesis. 27

NMR spectra of synthesized compounds 28

References 102

General Experimental

All reagents and solvents were purchased from Sigma-Aldrich, Chemtronica and Fischer Scientific and used as received unless stated otherwise. All reactions were performed under air atmosphere and without strictly anhydrous conditions unless stated otherwise. For reactions requiring heating, the mixture was heated using an oil bath placed on a hot plate and monitored with a thermometer. Meldrum's acid was recrystallized before use from heptane and acetone (1:1). TLC was performed on aluminum backed silica gel plates (median pore size 60 Å, fluorescent indicator 254 nm) and detected with UV light. Flash column chromatography was performed using silica gel with an average particle diameter of 50 µm (range 40–65 µm, pore diameter 53 Å), eluents are given in brackets. ^{1}H and ^{13}C NMR spectra were recorded on a Bruker 400 MHz or 600 MHz spectrometer at 298 K, calibrated by using the residual peak of the solvents as the internal standard ($CDCl_3$: δ (ppm) H = 7.26; δ (ppm) C = 77.16. DMSO-d^{6}: δ (ppm) H = 2.50; δ (ppm) C = 39.50. MeOD: δ (ppm) H = 3.31; δ (ppm) C = 49.00.). HRMS was performed by using an Agilent 1290 binary LC System connected to an Agilent 6230 Accurate-Mass TOF LC/MS (ESI+); calibrated with Agilent G1969-85001 ESTOF Reference Mix containing ammonium trifluoroacetate, purine and hexakis (1*H*, 1*H*, 3*H* tetrafluoropropoxy) phosphazine in 90:10 CH_3CN:H_2O.

General procedure for optimization

In a sealable tube, **aminopyrazole 1a (0.25 mmol)** and **acylated Meldrum's acid 2 (1.2 – 1.5 equiv)** were suspended in **the appropriate solvent (1 mL)**.

One step procedure: **Acid (1 - 10 equiv)** was added, the tube was sealed and placed in a pre-heated oil bath at 80 °C for 16 hours. After cooling to room temperature, completion was checked by TLC analysis, and the solvent was removed under reduced pressure. The crude product was purified by silica gel chromatography

Two-steps procedure: The tube was sealed and placed in a pre-heated oil bath at 80 °C for 1 hour. The appropriate **acid (1 – 2 equiv)** was added, the tube was sealed and placed in a pre-heated oil bath at 80 °C for 16 hours. After cooling to room temperature, completion was checked by TLC analysis, and the solvent was removed. The crude product was purified by silica gel chromatography.

Table S1. Optimization Table for Regioselective Cyclisation

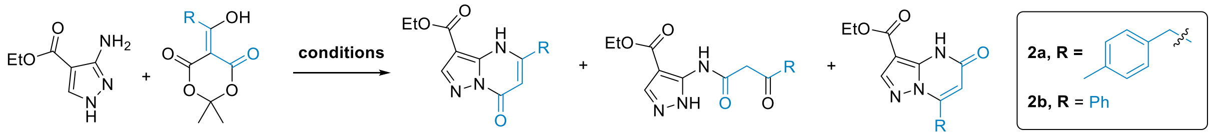

entry	**2** (equiv)	acid	conditions[a]	ratio **3**:**4**:**5**[b]	**3** (yield)[c]	**4** (yield)[c]	**5** (yield)[c]
1	**2a**, 1.5	none	EtOH, acid, 80°C, 16h	1:0:0	<10%	-	-
2	**2a**, 1.5	AcOH (10 equiv)	EtOH, acid, 80°C, 16h	1:0:0	55%	-	-
3	**2a**, 1.5	HCl (2 equiv)	EtOH, acid, 80°C, 16h	1:0:0	84%	-	-
4	**2a**, 1.5	TFA (2 equiv)	EtOH, acid, 80°C, 16h	1:0:0	84%	-	-
5	**2a**, 1.2	TFA (1 equiv)	EtOH, acid, 80°C, 16h	1:0:0	85%	-	-
6	**2a**, 1.5	HCl (4 equiv)	DCE, acid, 80°C, 16h	47:0:53	n.d.	-	17%
7	**2a**, 1.5	TFA (5 equiv)	DCE, acid, 80°C, 16h	19:0:81	n.d.	-	63%
8	**2a**, 1.5	none	Toluene, 80°C, 16h	0:81:19	-	73%	15%
9	**2a**, 1.5	none	MeCN, 80°C, 16h	0:62:38	-	46%	35%
10	**2a**, 1.5	TFA (2 equiv)	i) MeCN, 80°C, 1h<br>ii) acid, 80°C, 16h	0:0:1	-	-	89%

11	**2a**, 1.5	TFA (1 equiv)	i) MeCN, 80°C, 1h<br>ii) acid, 80°C, 16h	0:0:1	-	-	86%
12	**2b**, 1.5	TFA (1 equiv)	i) MeCN, 80°C, 1h<br>ii) acid, 80°C, 16h	0:65:35	-	50%	12%
13	**2b**, 1.5	TsOH.H_2O (1.5 equiv)	i) MeCN, 80°C, 1h<br>ii) acid, 80°C, 16h	0:0:1	-	-	75%
14	**2a**, 1.5	TsOH.H_2O (1 equiv)	EtOH, acid, 80°C, 16h	1:0:0	91%	-	-
15	**2a**, 1.5	TsOH.H_2O (1 equiv)	i) MeCN, 80°C, 1h<br>ii) acid, 80°C, 16h	0:0:1	-	-	94%

[a] Reaction conditions: aminopyrazole **1** (0.25 mmol), acylated Meldrum's acid **2** (1.2–1.5 equiv), acid (0–10 equiv), solvent (1 mL, 0.25 M), 80 °C, sealed tube, indicated time. For two-step reactions, the acid was added directly to the reaction mixture. [b] Ratio from crude ^{1}H NMR. [c] Isolated yield. n.d.: not determined

Scheme S1. 2-pots-2-steps synthesis of pyrazolo[1,5-*a*]pyrimidinones 3a and 5a.

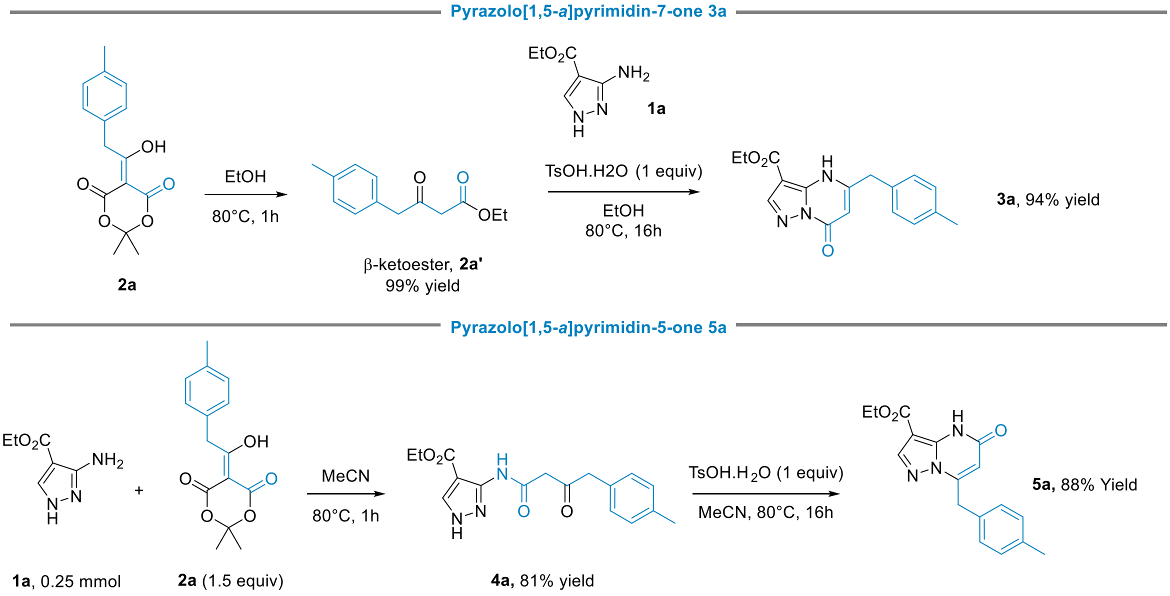

Scheme S2. Scope of DIPEA assisted synthesis of pyrazolo[1,5-*a*]pyrimidin-5-ones 5.

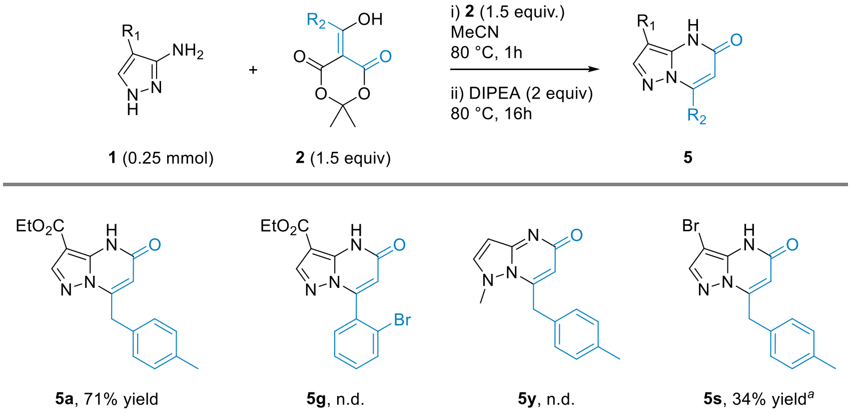

[a] debrominated **5r** was observed. n.d.: not detected.

2-Aminopyrazoles 1 synthesis

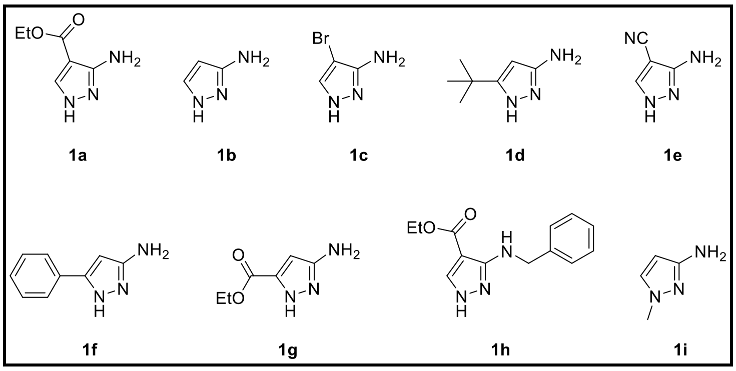

2-aminopyrazoles **1a**, **1b**, **1c**, **1d**, **1e**, **1f**, **1g** and **1i** were purchased from commercial suppliers.

ethyl 3-(benzylamino)-1*H*-pyrazole-4-carboxylate (1h):

1h was synthesized with modifications from a published procedure.[1]

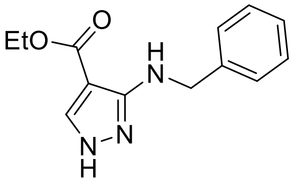

Ethyl 3-amino-4-pyrazolecarboxylate 1h (310 mg, 2 mmol) was suspended in **CH_2Cl_2 (10 mL, 0.2 M)**, and **benzaldehyde (244 µL, 1.2 equiv)** was added. **TFA (461 µL, 3 equiv)** was added dropwise, and the mixture was heated at reflux. **Et_3SiH (831 µL, 2.6 equiv)** was then added dropwise under reflux conditions, and the mixture was kept under heating for 90 minutes until TLC analysis showed completion. After cooling to room temperature, CH_2Cl_2 (10 mL) and water (10 mL) were added. Under stirring, the aqueous layer was made slightly basic (pH ~ 10) with dropwise addition of 15% aqueous NaOH. The layers were separated, and the aqueous layer was extracted with CH_2Cl_2 (20 mL). The combined organic layers were washed with brine and dried over $MgSO_4$. The solvent quantity was reduced under vacuum to a minimal amount, and heptane (50 mL) was added under stirring. The cloudy mixture was stirred in an ice bath until a precipitate formed and was then kept in the fridge overnight. The precipitate was filtered, dried, and purified by silica gel chromatography, using heptane:EtOAc (6:4) as the eluent system, to afford **1x (346 mg, 71% yield)** as a white solid.

^{1}H NMR (600 MHz, $CDCl_3$) δ 7.79 (br, 1H), 7.64 (s, 1H), 7.40 – 7.31 (m, 4H), 7.30 – 7.26 (m, 1H), 6.14 (s, 1H), 4.47 (s, 2H), 4.27 (q, *J* = 7.1 Hz, 2H), 1.33 (t, *J* = 7.2 Hz, 3H). **^{13}C NMR (151 MHz, $CDCl_3$) δ** 164.9, 155.2, 138.6, 135.9, 128.9, 127.6, 127.3, 96.8, 59.8, 47.6, 14.6.

Acylated Meldrum's acids synthesis

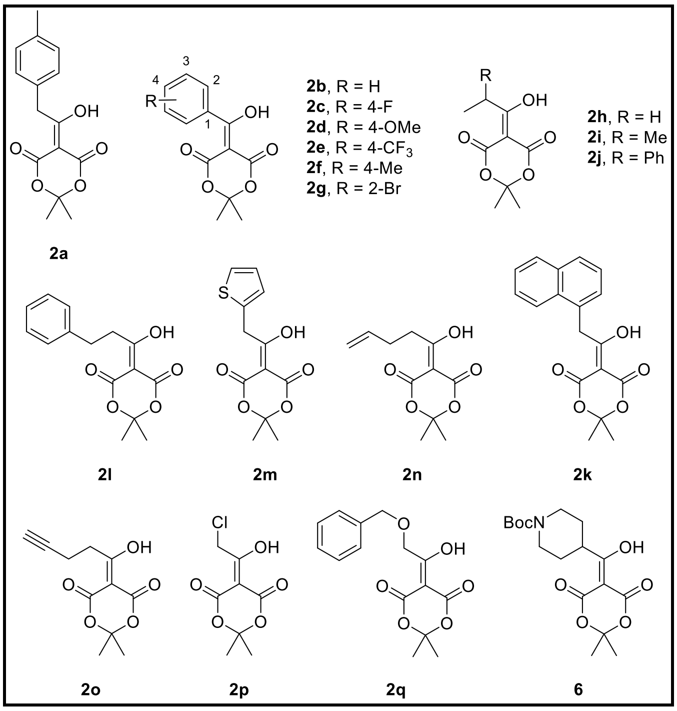

Acylated Meldrum's acids **2k**[2] and **2p**[3] were generously provided by the group of Fredrik Almqvist and were synthesized according to a published procedure.

5-(1-hydroxy-2-(p-tolyl)ethylidene)-2,2-dimethyl-1,3-dioxane-4,6-dione (2a):

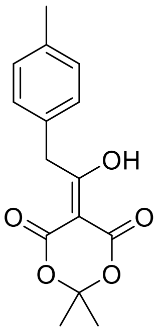

***p*-Tolylacetic acid (1502 mg, 10 mmol)**, **Meldrum's acid (1441 mg, 1 equiv)**, and **DMAP (3054 mg, 2.5 equiv)** were dissolved in **CH_2Cl_2 (100 mL, 0.1 M)**. The mixture was cooled with an ice bath, and **EDC·HCl (2684 mg, 1.4 equiv)** was added at once. The reaction was allowed to warm to room temperature in the thawing ice bath and stirred for 16 hours. The resulting mixture was diluted with CH_2Cl_2 and washed with aqueous 6% $KHSO_4$ (4 x 50 mL). The aqueous layer was extracted with CH_2Cl_2 (100 mL), and the combined organic layers were washed with brine, dried over $MgSO_4$, and filtered. The solvent was removed under reduced pressure at 30 °C to afford crude acylated Meldrum's acid. It was then dissolved in the minimum amount of acetone, and water (100 mL) was added. The resulting mixture was stirred for 30 minutes at 0 °C and the resulting precipitate was filtered out and dried to afford pure acylated Meldrum's acid **2b (2.313 g, 84% yield)**.

^{1}H NMR (400 MHz, $CDCl_3$) δ 15.31 (s, 1H), 7.27 (d, *J* = 5.8 Hz, 2H), 7.13 (d, *J* = 7.8 Hz, 2H), 4.39 (s, 2H), 2.33 (s, 3H), 1.72 (s, 6H). **^{13}C NMR (100 MHz, $CDCl_3$) δ** 195.1, 170.7, 160.5, 137.4, 131.2, 129.6, 129.6, 105.1, 91.5, 40.6, 27.0, 21.2. **HRMS** (ESI) *m/z*: $[M-H]^-$ calcd for $C_{15}H_{15}O_5$ 275.0925; found 275.0936.

5-(hydroxy(phenyl)methylene)-2,2-dimethyl-1,3-dioxane-4,6-dione (2b):

2b was synthesized with modifications from a published procedure.[4]

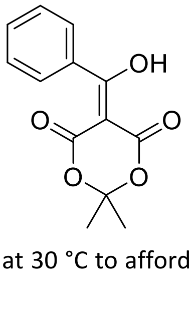

In a flame-dried flask under a nitrogen atmosphere, **Meldrum's acid (433 mg, 3 mmol)** and **DMAP (733 mg, 2 equiv)** were dissolved in dry **CH_2Cl_2 (15 mL, 0.2 M)**. The mixture was cooled to -10 °C using a salt-ice bath, and **benzoyl chloride (419 µL, 1.2 equiv)**, dissolved in dry **CH_2Cl_2 (5 mL)**, was added dropwise. The resulting mixture was stirred for 1 hour at -10 °C and 1 hour at room temperature until TLC analysis showed complete conversion. The solution was diluted with CH_2Cl_2 (20 mL) and washed with 1 M HCl (3 x 10 mL) and water (30 mL). The organic layer was dried over $MgSO_4$, and the solvent was removed under reduced pressure at 30 °C to afford an orange crude solid. The solid was suspended in a minimum amount of acetone and mildly

heated (less than 40 °C) until all solids dissolved. The solution was placed in the freezer at -20 °C for 16 hours. The resulting solid was filtered out and washed with cold acetone to afford 300 mg of a white solid. The solvent was removed from the filtrate, and the recrystallization was repeated to afford 100 mg of a white solid. Combination of the two batches of recrystallization afforded **2b (404 mg, 54% yield)** as a white solid.

^{1}H NMR (400 MHz, $CDCl_3$) δ 15.47 (s, 1H), 7.71 – 7.66 (m, 2H), 7.63 – 7.57 (m, 1H), 7.51 – 7.44 (m, 2H), 1.85 (s, 6H). **^{13}C NMR (100 MHz, $CDCl_3$) δ** 189.4, 171.2, 160.0, 133.5, 132.9, 129.6, 128.2, 105.2, 91.1, 27.0.

5-((4-fluorophenyl)(hydroxy)methylene)-2,2-dimethyl-1,3-dioxane-4,6-dione (2c):

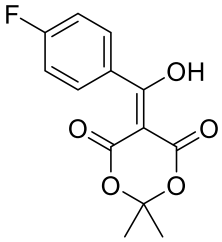

4-Fluorobenzoic acid (141 mg, 1 mmol), **Meldrum's acid (145 mg, 1 equiv)**, and **DMAP (306 mg, 2.5 equiv)** were dissolved in **CH_2Cl_2 (10 mL, 0.1 M)**. The mixture was cooled with an ice bath, and **EDC·HCl (269 g, 1.4 equiv)** was added at once. The reaction was allowed to warm to room temperature in the thawing ice bath and stirred for 16 hours. The resulting mixture was diluted with CH_2Cl_2 and washed with aqueous 6% $KHSO_4$ (3 x 25 mL). The aqueous layer was extracted with CH_2Cl_2 (25 mL), and the combined organic layers were washed with brine, dried over $MgSO_4$, and filtered. The solvent was removed under reduced pressure at 30 °C to afford crude acylated Meldrum's acid. The solid was suspended in Et_2O and stirred for 1 hour, it was then filtered and the solvent was removed under reduced pressure to afford pure acylated Meldrum's acid **2c** (**235 mg, 88% yield)**.

^{1}H NMR (400 MHz, $CDCl_3$) δ 15.57 (s, 1H), 7.80 – 7.68 (m, 2H), 7.19 – 7.11 (m, 2H), 1.84 (s, 6H). **^{13}C NMR (100 MHz, $CDCl_3$) δ** 188.1, 171.2, 166.0 (d, J = 256.0 Hz), 160.0, 132.5 (d, J = 9.5 Hz), 128.8 (d, J = 3.3 Hz), 115.6 (d, J = 22.4 Hz), 105.2, 90.9, 27.0. **^{19}F NMR (376 MHz, $CDCl_3$) δ** -103.66 – -103.78 (m). Spectral data were in accordance with published data.[5]

5-(hydroxy(4-methoxyphenyl)methylene)-2,2-dimethyl-1,3-dioxane-4,6-dione (2d):

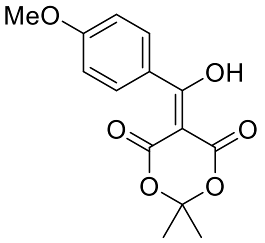

4-methoxybenzoic acid (305 mg, 2 mmol), **Meldrum's acid (303 mg, 1.05 equiv)**, and **DMAP (611 mg, 2.5 equiv)** were dissolved in **CH_2Cl_2 (20 mL, 0.1 M)**. The mixture was cooled with an ice bath, and **EDC·HCl (441 mg, 1.15 equiv)** was added at once. The reaction was allowed to warm to room temperature in the thawing ice bath and stirred for 16 hours. The resulting mixture was diluted with CH_2Cl_2 and washed successively with aqueous 1M $KHSO_4$ (3 x 20 mL), water (20 mL), and brine. It was then dried over $MgSO_4$ and filtered. The solvent was removed under reduced pressure at 30 °C to afford the crude acylated Meldrum's acid. The residue was purified by silica gel chromatography using heptane:EtOAc (gradient 0 to 100% EtOAc) with AcOH (1%) as the eluant. The obtained solid was suspended in water, sonicated for 10 minutes, filtered, and thoroughly dried under vacuum to afford **2d (203 mg, 37% yield)** as a light orange solid.

^{1}H NMR (600 MHz, $CDCl_3$) δ 15.48 (s, 1H), 7.74 (d, J = 8.9 Hz, 2H), 6.95 (d, J = 8.9 Hz, 2H), 3.89 (s, 3H), 1.84 (s, 6H). **^{13}C NMR (151 MHz, $CDCl_3$) δ** 188.4, 171.3, 164.5, 160.6, 132.6, 124.6, 113.7, 104.8, 89.8, 55.7, 26.9. Spectral data were in accordance with published data.[6]

5-(hydroxy(4-(trifluoromethyl)phenyl)methylene)-2,2-dimethyl-1,3-dioxane-4,6-dione (2e):

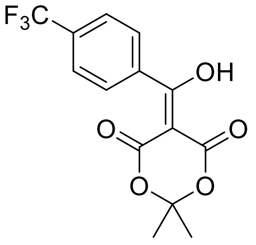

4-(Trifluoromethyl)benzoic acid (381 mg, 2 mmol), **Meldrum's acid (289 mg, 1 equiv)**, and **DMAP (611 mg, 2.5 equiv)** were dissolved in **CH_2Cl_2 (20 mL, 0.1 M)**. The mixture was cooled with an ice bath, and **EDC·HCl (537 mg, 1.4 equiv)** was added at once. The reaction was allowed to warm to room temperature in the thawing ice bath and stirred for 16 hours. The resulting mixture was diluted with CH_2Cl_2 and washed with aqueous 6% $KHSO_4$ (3 x 20 mL). The aqueous layer was extracted with CH_2Cl_2 (20 mL), and the combined organic layers were washed with brine, dried over $MgSO_4$, and filtered. The solvent was removed under reduced pressure at 30 °C to afford acylated Meldrum's acid **2e (580 mg, 92% yield)** as a yellow solid, which was judged sufficiently pure. No further purification was performed.

^{1}H NMR (600 MHz, $CDCl_3$) δ 15.56 (s, 1H), 7.78 – 7.75 (m, 2H), 7.75 – 7.71 (m, 2H), 1.85 (s, 6H). **^{13}C NMR (151 MHz, $CDCl_3$) δ** 187.9, 171.0, 159.5, 136.3, 134.5 (q, *J* = 33.0 Hz), 129.8, 125.3 (q, *J* = 3.9 Hz), 123.6 (q, *J* = 272.3 Hz), 105.6, 91.9, 27.1. **^{19}F NMR (565 MHz, $CDCl_3$) δ** -63.21. **HRMS** (ESI) *m/z*: $[M-H]^-$ calcd for $C_{14}H_{10}F_3O_5$ 315.0486; found 315.0488.

5-(hydroxy(*p*-tolyl)methylene)-2,2-dimethyl-1,3-dioxane-4,6-dione (2f):

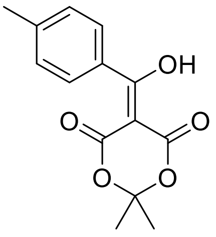

***p*-Toluic acid (273 mg, 2 mmol)**, **Meldrum's acid (289 mg, 1 equiv)**, and **DMAP (611 mg, 2.5 equiv)** were dissolved in **CH_2Cl_2 (20 mL, 0.1 M)**. The mixture was cooled with an ice bath, and **EDC·HCl (537 mg, 1.4 equiv)** was added at once. The reaction was allowed to warm to room temperature in the thawing ice bath and stirred for 16 hours. The resulting mixture was diluted with CH_2Cl_2 and washed successively with aqueous 6% $KHSO_4$ (3 x 20 mL), water (20 mL), and brine. It was then dried over $MgSO_4$ and filtered. The solvent was removed under reduced pressure at 30 °C to afford the crude acylated Meldrum's acid. The solid was dissolved in the minimum amount of acetone, placed under stirring at 0 °C, and heptane (50 mL) was added. The mixture was stirred for 15 minutes until a precipitate formed. It was then kept overnight in a freezer at -20 °C, filtered, washed with heptane, and thoroughly dried under vacuum to afford **2f (346 mg, 66% yield)** as a light-yellow solid.

^{1}H NMR (600 MHz, $CDCl_3$) δ 15.44 (s, 1H), 7.62 – 7.58 (m, 2H), 7.30 – 7.25 (m, 2H), 2.44 (s, 3H), 1.84 (s, 6H). **^{13}C NMR (151 MHz, $CDCl_3$) δ** 189.3, 171.2, 160.2, 144.9, 129.9, 129.0, 105.0, 90.6, 26.9, 22.0. **HRMS** (ESI) *m/z*: $[3M+K-2H]^-$ calcd for $C_{42}H_{40}KO_{15}$ 823.2009; found 823.2055.

5-((2-bromophenyl)(hydroxy)methylene)-2,2-dimethyl-1,3-dioxane-4,6-dione (2g):

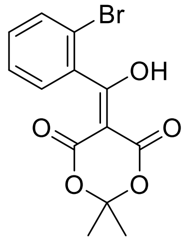

2-Bromobenzoic acid (403 mg, 2 mmol), **Meldrum's acid (289 mg, 1 equiv)**, and **DMAP (611 mg, 2.5 equiv)** were dissolved in **CH_2Cl_2 (20 mL, 0.1 M)**. The mixture was cooled with an ice bath, and **EDC·HCl (537 mg, 1.4 equiv)** was added at once. The reaction was allowed to warm to room temperature in the thawing ice bath and stirred for 16 hours. The resulting mixture was diluted with CH_2Cl_2 and washed successively with aqueous 6% $KHSO_4$ (3 x 20 mL), water (20 mL), and brine. It was then dried over $MgSO_4$ and filtered. The solvent was removed under reduced pressure at 30 °C to afford the crude acylated Meldrum's acid. The solid was dissolved in the minimum amount of acetone, placed under stirring at 0 °C, and heptane (50 mL) was added. The mixture was stirred for 15 minutes until a precipitate formed. It was then kept overnight in a freezer at -20 °C, filtered, washed with heptane, and thoroughly dried under vacuum to afford **2g (541 mg, 83% yield)** as a light-yellow solid.

^{1}H NMR (600 MHz, $CDCl_3$) δ 15.14 (s, 1H), 7.64 (d, *J* = 8.0 Hz, 1H), 7.45 (t, *J* = 7.5 Hz, 1H), 7.41 – 7.33 (m, 2H), 1.82 (s, 6H). **^{13}C NMR (151 MHz, $CDCl_3$) δ** 187.2, 170.5, 158.9, 135.4, 133.1, 132.2, 128.7, 127.6, 120.2, 105.8, 94.0, 27.4. **HRMS** (ESI) *m/z*: $[3M+K-2H]^-$ calcd for $C_{39}H_{31}Br_3KO_{15}$ 1014.8855; found 1014.8919.

5-(1-hydroxypropylidene)-2,2-dimethyl-1,3-dioxane-4,6-dione (2h):

2h was synthesized with modifications from a published procedure.[7]

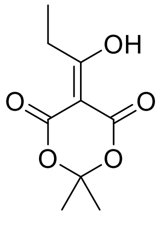

In a flame-dried flask under a nitrogen atmosphere, **Meldrum's acid (721 mg, 5 mmol)** was dissolved in dry **CH_2Cl_2 (5 mL, 1M)**. The mixture was cooled to 0 °C, and **pyridine (806 µL, 10 mmol, 2 equiv)** was added at once, followed by a dropwise addition of **propionyl chloride (480 µL, 5.5 mmol, 1.1 equiv)**. The resulting mixture was stirred for 1 hour at 0 °C and then for 1 hour at room temperature. The resulting orange cloudy mixture was diluted with CH_2Cl_2 (20 mL) and poured into icy 1M aqueous HCl (20 mL) and stirred for 15 minutes. The organic layer was recovered and washed successively with 1M HCl (20 mL), water (2 x 20 mL), and brine (20 mL), then dried over $MgSO_4$. The solvent was removed under reduced pressure at 30 °C to afford an oil which still contained starting Meldrum's acid and acyl chloride derivatives. The oil was sonicated in water (25 mL) and extracted with Et_2O (2 x 25 mL). The solvent was removed under reduced pressure, and the resulting oil was

placed at -20 °C overnight. The resulting wet solid was crushed and placed under high vacuum for 6 hours to afford **2h (342 mg, 34% yield)** as a light orange powder.

^{1}H NMR (400 MHz, $CDCl_3$) δ 15.38 (s, 1H), 3.11 (q, *J* = 7.4 Hz, 2H), 1.73 (s, 6H), 1.26 (t, *J* = 7.4 Hz, 3H). **^{13}C NMR (100 MHz, $CDCl_3$) δ** 199.1, 170.8, 160.3, 105.0, 91.1, 29.6, 27.0, 9.9.

5-(1-hydroxy-2-methylpropylidene)-2,2-dimethyl-1,3-dioxane-4,6-dione (2i):

2i was synthesized with modifications from a published procedure.[8]

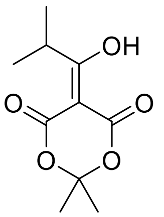

In a flame-dried flask under a nitrogen atmosphere, **Meldrum's acid (433 mg, 3 mmol)** was dissolved in dry **CH_2Cl_2 (15 mL, 0.2 M)**. **Pyridine (484 µL, 2 equiv)** was added at 0 °C, and the mixture was stirred for 15 minutes before the dropwise addition of **isobutyryl chloride (385 µL, 1.2 equiv)**. The resulting mixture was stirred for 1.5 hours at 0 °C and for 1.5 hours at room temperature until TLC analysis showed complete conversion. The organic layer was washed with 1 M aqueous HCl (30 mL). The aqueous layer was extracted with CH_2Cl_2 (30 mL), and the combined organic layers were dried with $MgSO_4$. The solvent was removed under reduced pressure, and the residue was purified by silica gel chromatography using heptane:EtOAc (9:1 with 1% AcOH) to afford **2i (355 mg, 55% yield)** as a translucent oil, which solidified in the freezer.

^{1}H NMR (400 MHz, $CDCl_3$) δ 15.54 (d, *J* = 1.3 Hz, 1H), 4.09 (heptd, *J* = 6.6, 0.9 Hz, 1H), 1.74 (s, 6H), 1.24 (d, *J* = 6.8 Hz, 6H). **^{13}C NMR (100 MHz, $CDCl_3$) δ** 202.6, 171.1, 160.2, 104.9, 90.3, 33.2, 26.9, 19.3.

5-(1-hydroxy-2-phenylpropylidene)-2,2-dimethyl-1,3-dioxane-4,6-dione (2j):

2j was synthesized with modifications from a published procedure.[9]

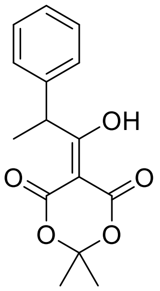

In a flame-dried flask under a nitrogen atmosphere, **2-phenylpropionic acid (451 mg, 3 mmol)**, **Meldrum's acid (476 mg, 1.1 equiv)**, and **DMAP (587 mg, 1.6 equiv)** were dissolved in dry **CH_2Cl_2 (15 mL)** and cooled in an ice bath. **DCC (3.45 mL, 1 M in CH_2Cl_2, 1.15 equiv)** was added dropwise at 0 °C to the reaction mixture. The reaction was allowed to warm to room temperature in the thawing ice bath and stirred for 16 hours. After aging, the yellow solution was cooled to 0 °C for 10 minutes and filtered. The filtrate was washed with 1 M aqueous $KHSO_4$ (3 × 20 mL). The combined aqueous layers were extracted with CH_2Cl_2 (50 mL). The organic layers were combined, washed with brine, dried over $MgSO_4$, filtered, and concentrated under reduced pressure to afford an oily residue. This residue was purified by silica gel chromatography, eluting with CH_2Cl_2, to afford a pure fraction of **2j (400 mg, 48% yield)**. All remaining product in the column after further elution with more polar solvent showed degradation.

^{1}H NMR (600 MHz, $CDCl_3$) δ 15.70 (d, *J* = 1.4 Hz, 1H), 7.45 – 7.41 (m, 2H), 7.34 – 7.31 (m, 2H), 7.30 – 7.24 (m, 1H), 5.46 (q, *J* = 7.0 Hz, 1H), 1.74 (s, 3H), 1.63 (s, 3H), 1.59 (d, *J* = 7.2 Hz, 3H). **^{13}C NMR (151 MHz, $CDCl_3$) δ** 198.6, 171.1, 160.3, 140.2, 128.8, 128.4, 127.7, 105.0, 90.9, 42.8, 27.0, 26.8, 18.2.

5-(1-hydroxy-3-phenylpropylidene)-2,2-dimethyl-1,3-dioxane-4,6-dione (2l):

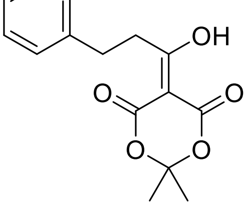

3-Phenylpropionic acid (301 mg, 2 mmol), **Meldrum's acid (317 mg, 1.1 equiv)**, and **DMAP (611 mg, 2.5 equiv)** were dissolved in **CH_2Cl_2 (20 mL, 0.1 M)**. The mixture was cooled with an ice bath, and **EDC·HCl (441 mg, 1.15 equiv)** was added at once. The reaction was allowed to warm to room temperature in the thawing ice bath and stirred for 16 hours. The resulting mixture was diluted with CH_2Cl_2 and washed successively with aqueous 1M $KHSO_4$ (3 x 20 mL), water (20 mL), and brine. It was then dried over $MgSO_4$ and filtered. The solvent was removed under reduced pressure at 30 °C to afford the crude acylated Meldrum's acid. The solid was suspended in water, sonicated for 10 minutes, filtered, and thoroughly dried under vacuum to afford **2l (433 mg, 78% yield)** as a white solid.

^{1}H NMR (600 MHz, $CDCl_3$) δ 15.30 (s, 1H), 7.31 – 7.27 (m, 2H), 7.27 – 7.24 (m, 2H), 7.23 – 7.18 (m, 1H), 3.41 (dd, *J* = 8.8, 6.6 Hz, 2H), 3.02 (dd, *J* = 8.8, 6.6 Hz, 2H), 1.67 (s, 6H). **^{13}C NMR (151 MHz, $CDCl_3$) δ** 196.6, 170.6, 160.3, 139.8, 128.7, 128.6, 126.7, 105.0, 91.2, 37.3, 32.1, 26.9. Spectral data were in accordance with published data.[10]

5-(1-hydroxy-2-(thiophen-2-yl)ethylidene)-2,2-dimethyl-1,3-dioxane-4,6-dione (2m):

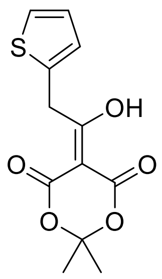

2-Thiopheneacetic acid (285 mg, 2 mmol), **Meldrum's acid (318 mg, 1 equiv)**, and **DMAP (611 mg, 2.5 equiv)** were dissolved in **CH_2Cl_2 (20 mL, 0.1 M)**. The mixture was cooled with an ice bath, and **EDC·HCl (461 mg, 1.2 equiv)** was added at once. The reaction was allowed to warm to room temperature in the thawing ice bath and stirred for 16 hours. The resulting mixture was diluted with CH_2Cl_2 and washed with aqueous 6% $KHSO_4$ (4 x 20 mL). The aqueous layer was extracted with CH_2Cl_2 (25 mL), and the combined organic layers were washed with brine, dried over $MgSO_4$, and filtered. The solvent was removed under reduced pressure at 30 °C to afford crude acylated Meldrum's acid. It was then dissolved in the minimum amount of acetone, and water (25 mL) was added. The resulting mixture was stirred for 30 minutes at 0 °C and the resulting precipitate was filtered out and dried to afford pure acylated Meldrum's acid **2m** (500 g, 93% yield).

^{1}H NMR (600 MHz, $CDCl_3$) δ 15.33 (s, 1H), 7.23 (dd, *J* = 5.2, 1.3 Hz, 1H), 7.06 (dd, *J* = 3.5, 1.0 Hz, 1H), 6.97 (dd, *J* = 5.2, 3.5 Hz, 1H), 4.61 (s, 2H), 1.74 (s, 6H). **^{13}C NMR (151 MHz, $CDCl_3$) δ** 193.1, 170.6, 160.4, 134.7, 128.0, 127.1, 125.8, 105.4, 91.4, 35.4, 27.1. **HRMS** (ESI) *m/z*: $[M-H]^-$ calcd for $C_{12}H_{11}O_5S$ 267.0332; found 267.0304.

5-(1-hydroxypent-4-en-1-ylidene)-2,2-dimethyl-1,3-dioxane-4,6-dione (2n):

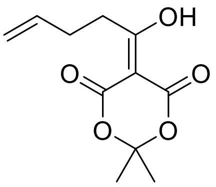

4-Pentenoic acid (204 µL, 2 mmol), **Meldrum's acid (289 mg, 1 equiv)**, and **DMAP (611 mg, 2.5 equiv)** were dissolved in **CH_2Cl_2 (20 mL, 0.1 M)**. The mixture was cooled with an ice bath, and **EDC·HCl (537 mg, 1.4 equiv)** was added at once. The reaction was allowed to warm to room temperature in the thawing ice bath and stirred for 16 hours. The resulting mixture was diluted with CH_2Cl_2 and washed with aqueous 6% $KHSO_4$ (3 x 20 mL). The aqueous layer was extracted with CH_2Cl_2 (20 mL), and the combined organic layers were washed with brine, dried over $MgSO_4$, and filtered. The solvent was removed under reduced pressure at 30 °C to afford acylated Meldrum's acid **2n (418 mg, 92% yield)** as a light orange oil, which was judged sufficiently pure. No further purification was performed.

^{1}H NMR (600 MHz, $CDCl_3$) δ 15.33 (s, 1H), 5.91 – 5.80 (m, 1H), 5.09 (dq, *J* = 17.1, 1.6 Hz, 1H), 5.03 (dq, *J* = 10.2, 1.3 Hz, 1H), 3.20 (t, *J* = 7.5 Hz, 2H), 2.52 – 2.43 (m, 2H), 1.73 (s, 6H). **^{13}C NMR (151 MHz, $CDCl_3$) δ** 197.2, 170.7, 160.4, 136.2, 116.4, 105.1, 91.8, 35.1, 30.0, 27.0. **HRMS** (ESI) *m/z*: $[M-H]^-$ calcd for $C_{11}H_{13}O_5$ 225.0768; found 225.0784.

5-(1-hydroxypent-4-yn-1-ylidene)-2,2-dimethyl-1,3-dioxane-4,6-dione (2o):

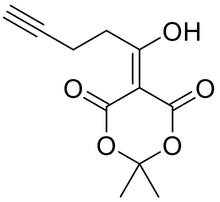

4-Pentynoic acid (207 mg, 2 mmol), **Meldrum's acid (289 mg, 1 equiv)**, and **DMAP (611 mg, 2.5 equiv)** were dissolved in **CH_2Cl_2 (20 mL, 0.1 M)**. The mixture was cooled with an ice bath, and **EDC·HCl (537 mg, 1.4 equiv)** was added at once. The reaction was allowed to warm to room temperature in the thawing ice bath and stirred for 16 hours. The resulting mixture was diluted with CH_2Cl_2 and washed with aqueous 6% $KHSO_4$ (3 x 20 mL). The aqueous layer was extracted with CH_2Cl_2 (20 mL), and the combined organic layers were washed with brine, dried over $MgSO_4$, and filtered. The solvent was removed under reduced pressure at 30 °C to afford crude acylated Meldrum's acid. The solid was dissolved in the minimum amount of acetone, and water (25 mL) was added. The mixture was stirred at 0 °C for 15 minutes, and the resulting precipitate was filtered out and dried to afford **2o (365 mg, 81% yield)** as a white solid.

^{1}H NMR (600 MHz, $CDCl_3$) δ 15.45 (s, 1H), 3.35 (t, *J* = 7.2 Hz, 2H), 2.63 (td, *J* = 7.2, 2.7 Hz, 2H), 1.99 (t, *J* = 2.7 Hz, 1H), 1.75 (s, 6H). **^{13}C NMR (151 MHz, $CDCl_3$) δ** 195.0, 170.6, 160.3, 105.3, 92.1, 81.8, 69.8, 34.6, 27.0, 14.9. . Spectral data were in accordance with published data.[11]

5-(2-(benzyloxy)-1-hydroxyethylidene)-2,2-dimethyl-1,3-dioxane-4,6-dione (2q):

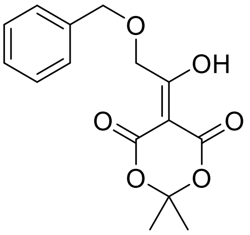

Benzyloxyacetic acid (295 µL, 2 mmol), **Meldrum's acid (289 mg, 1 equiv)**, and **DMAP (611 mg, 2.5 equiv)** were dissolved in **CH_2Cl_2 (20 mL, 0.1 M)**. The mixture was cooled with an ice bath, and **EDC·HCl (537 mg, 1.4 equiv)** was added at once. The reaction was allowed to warm to room temperature in the thawing ice bath and stirred for 16 hours. The resulting mixture was diluted with CH_2Cl_2 and washed successively with aqueous 1 M $KHSO_4$ (3 x 20 mL), water (20 mL), and brine. It was then dried over $MgSO_4$ and filtered. The solvent was removed under reduced pressure at 30 °C to afford the crude acylated Meldrum's acid. The solid was dissolved in the minimum amount of acetone, placed under stirring at 0 °C, and heptane (25 mL) was added. The mixture was stirred for 15 minutes until a precipitate formed. It was then kept overnight in a freezer at -20 °C, filtered, and thoroughly dried under vacuum to afford **2q (375 mg, 64% yield)** as a white solid.

^{1}H NMR (600 MHz, $CDCl_3$) δ 15.70 (s, 1H), 7.37 (dd, *J* = 12.7, 5.9 Hz, 4H), 7.35 – 7.29 (m, 1H), 4.94 (s, 2H), 4.69 (s, 2H), 1.73 (s, 6H). **^{13}C NMR (151 MHz, $CDCl_3$) δ** 194.3, 170.6, 160.0, 137.0, 128.7, 128.4, 128.2, 106.0, 90.3, 74.2, 69.8, 27.0. **HRMS** (ESI) *m/z*: $[2M+Na-H]^-$ calcd for $C_{30}H_{30}NaO_{12}$ 605.1640; found 605.1578.

***tert*-butyl 4-((2,2-dimethyl-4,6-dioxo-1,3-dioxan-5-ylidene)(hydroxy)methyl)piperidine-1-carboxylate (6):**

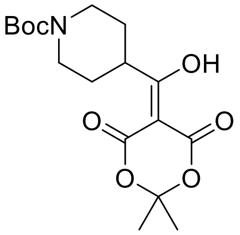

Boc-isonipecotic acid (1147 mg, 5 mmol), **Meldrum's acid (721 mg, 1 equiv)**, and **DMAP (1527 mg, 2.5 equiv)** were dissolved in **CH_2Cl_2 (50 mL, 0.1 M)**. The mixture was cooled with an ice bath, and **EDC·HCl (1342 mg, 1.4 equiv)** was added at once. The reaction was allowed to warm up to room temperature in the thawing ice bath under stirring overnight. The resulting mixture was diluted with CH_2Cl_2 and washed with aqueous 6% $KHSO_4$ (3 x 40 mL). The aqueous layer was extracted with CH_2Cl_2 (50 mL), and the combined organic layers were washed with brine, dried over $MgSO_4$, and filtered. The solvent was removed under reduced pressure at 30 °C to afford crude acylated Meldrum's acid. The solid was dissolved in the minimum amount of acetone, and water (25 mL) was added. The mixture was stirred at 0 °C for 15 minutes, and the resulting precipitate was filtered out and dried to afford **6 (1582 mg, 82% yield)** as a white solid

^{1}H NMR (600 MHz, $CDCl_3$) δ 15.58 (s, 1H), 4.22 (br, 2H), 3.95 (tt, *J* = 11.6, 3.5 Hz, 1H), 2.82 (br, 2H), 1.86 – 1.78 (m, 2H), 1.74 (s, 6H), 1.73 – 1.67 (m, 2H), 1.47 (s, 9H). **^{13}C NMR (151 MHz, $CDCl_3$) δ** 199.3, 171.0, 160.2, 154.8, 105.1, 90.8, 79.9, 43.2 (br), 41.3, 28.6, 28.3, 27.0. Spectral data were in accordance with published data.[12]

Pyrazolo[1,5-*a*]pyrimidin-7-ones 3 synthesis

General procedure A

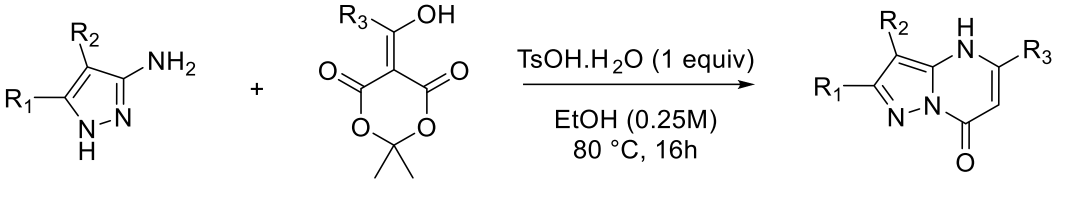

1 **2** (1.2 - 1.5 equiv) **3**

In a sealable tube, the appropriate **aminopyrazole 1 (1 equiv)** and **acylated Meldrum's acid 2 (1.2 – 1.5 equiv)** were suspended in **EtOH (0.25 M)**. **TsOH.H$_2$O (1 equiv)** was added, the tube was sealed, and placed in a pre-heated oil bath at 80 °C for 16 hours. After cooling to room temperature, completion was checked by TLC analysis, and the solvent was removed under reduced pressure. The crude product was purified by silica gel chromatography to afford pure **5-substituted pyrazolo[1,5-*a*]pyrimidin-7-one 3**.

General rules and tips

(1) Reactions involving acylated Meldrum's acids **2** bearing secondary alkyl or aryl substituents typically reach full conversion when 1.5 equivalents of **2** are used.
(2) If a precipitate is observed at the end of the reaction, addition of Et$_2$O often enhances precipitation, enabling easier isolation of the pure product without silica gel chromatography.
(3) Most target compounds **3** can be visualized by TLC using a heptane:EtOAc eluant system. However, when this solvent system is applied to silica gel chromatography, significant tailing and poor separation are usually observed. A CH$_2$Cl$_2$:MeOH gradient is therefore recommended for optimal purification.

ethyl 5-(4-methylbenzyl)-7-oxo-4,7-dihydropyrazolo[1,5-*a*]pyrimidine-3-carboxylate (3a):

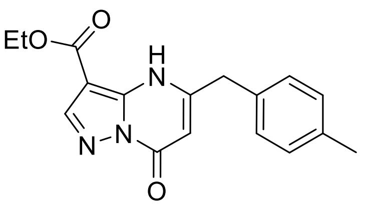

According to the general procedure A, **ethyl 3-amino-4-pyrazolecarboxylate 1a (39 mg, 0.25 mmol)**, **2a (83 mg, 1.2 equiv)** and **TsOH.H$_2$O (48 mg, 1 equiv)** were used. Purification by silica gel chromatography, using CH$_2$Cl$_2$:MeOH (gradient 0% MeOH up to 2%) as the eluant system, to afford **3a (71 mg, 91% yield)** as a white solid.

^{1}H NMR (400 MHz, CDCl$_3$) δ 9.25 (s, 1H), 8.10 (s, 1H), 7.22 (d, *J* = 8.0 Hz, 2H), 7.16 (d, *J* = 8.2 Hz, 2H), 5.88 (d, *J* = 2.4 Hz, 1H), 4.28 (q, *J* = 7.2 Hz, 2H), 3.95 (s, 2H), 2.36 (s, 3H), 1.30 (t, *J* = 7.2 Hz, 3H). **^{13}C NMR (100 MHz, CDCl$_3$) δ** 163.2, 156.0, 151.3, 143.8, 143.0, 138.4, 130.5, 130.4, 129.2, 99.8, 97.3, 60.8, 39.1, 21.3, 14.4. **HRMS** (ESI) *m/z*: [M-H]$^-$ calcd for $C_{17}H_{16}N_3O_3$ 310.1197; found 310.1187.

2.5 mmol scale:

In a round-bottom flask equipped with a reflux condenser, **ethyl 3-amino-4-pyrazolecarboxylate 1a (388 mg, 2.5 mmol)** and **2a (829 mg, 1.2 equiv)** were suspended in **EtOH (10 mL)**. **TsOH·H2O (476 mg, 1 equiv)** was added, and the mixture was placed in a pre-heated bath at 80 °C for 16 hours. After cooling to room temperature, completion was checked by TLC analysis. The crude mixture was diluted with Et$_2$O (50 mL) and stirred for 15 minutes in an ice bath. The resulting precipitate was filtered under vacuum and dried to afford **3a (633 mg, 81% yield)** as a white solid.

With β-ketoester 2a':

In a sealable tube **ethyl 3-amino-4-pyrazolecarboxylate 1a (39 mg, 0.25 mmol)** and β-ketoester **2a' (66 mg, 1.2 equiv)** were suspended in **EtOH (1 mL)**. The tube was sealed, and placed in a pre-heated bath at 80 °C for 1 hour. After cooling to room temperature, completion was checked by TLC analysis, and the solvent was removed under reduced pressure. The crude product was purified by silica gel chromatography, using CH$_2$Cl$_2$:MeOH (gradient 0% MeOH up to 2%) as the eluant system, to afford **2a' (73 mg, 94% yield)** as a white solid.

ethyl 7-oxo-5-phenyl-4,7-dihydropyrazolo[1,5-*a*]pyrimidine-3-carboxylate (3b):

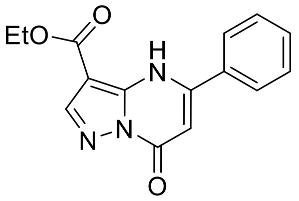

According to the general procedure A, **ethyl 3-amino-4-pyrazolecarboxylate 1a (39 mg, 0.25 mmol)**, **2a (94 mg, 1.5 equiv)** and **TsOH.H$_2$O (48 mg, 1 equiv)** were used. Purification by silica gel chromatography, using CH$_2$Cl$_2$:MeOH (gradient 0% MeOH up to 1.5%) as the eluant system, to afford **3b (59 mg, 83% yield)** as a white solid.

^{1}H NMR (400 MHz, CDCl$_3$) δ 9.83 (s, 1H), 8.19 (s, 1H), 7.74 – 7.65 (m, 2H), 7.65 – 7.54 (m, 3H), 6.28 (d, *J* = 2.3 Hz, 1H), 4.39 (q, *J* = 7.1 Hz, 2H), 1.42 (t, *J* = 7.1 Hz, 3H). **^{13}C NMR (100 MHz, CDCl$_3$) δ** 163.8, 156.2, 149.5, 144.3, 143.0, 132.2, 131.7, 130.0, 126.5, 98.3, 97.7, 61.1, 14.5. **HRMS** (ESI) *m/z*: [M-H]$^-$ calcd for $C_{15}H_{12}N_3O_3$ 282.0884; found 282.0861.

ethyl 5-(4-fluorophenyl)-7-oxo-4,7-dihydropyrazolo[1,5-a]pyrimidine-3-carboxylate (3c):

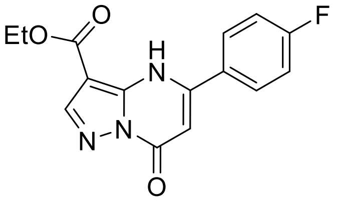

According to the general procedure A, using **ethyl 3-amino-4-pyrazolecarboxylate 1a (39 mg, 0.25 mmol)**, **2c (100 mg, 1.5 equiv)** and **TsOH.H$_2$O (48 mg, 1 equiv)**. Purification by silica gel chromatography, using CH$_2$Cl$_2$:MeOH (gradient 0% MeOH up to 1.5%) as the eluant system, to afford **3c (54 mg, 72% yield)** as a white solid.

^{1}H NMR (600 MHz, CDCl$_3$) δ 9.78 (s, 1H), 8.18 (s, 1H), 7.72 – 7.67 (m, 2H), 7.31 – 7.27 (m, 2H), 6.22 (d, *J* = 2.4 Hz, 1H), 4.39 (q, *J* = 7.2 Hz, 2H), 1.42 (t, *J* = 7.2 Hz, 3H). **^{13}C NMR (151 MHz, CDCl$_3$) δ** 165.0 (d, *J* = 254.7 Hz), 163.9, 156.0, 148.5, 144.3, 143.0, 128.7 (d, *J* = 8.8 Hz), 127.9 (d, *J* = 3.3 Hz), 117.3 (d, *J* = 22.0 Hz), 98.3, 97.7, 61.2, 14.5. **^{19}F NMR (565 MHz, CDCl$_3$) δ** -106.42 – -106.49 (m). **HRMS** (ESI) *m/z*: [M-H]$^-$ calcd for $C_{15}H_{11}FN_3O_3$ 300.0790; found 300.0783.

ethyl 5-(4-methoxyphenyl)-7-oxo-4,7-dihydropyrazolo[1,5-*a*]pyrimidine-3-carboxylate (3d):

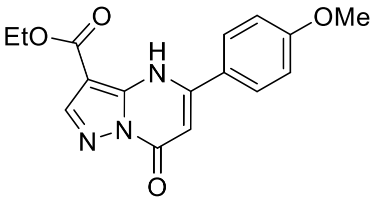

According to the general procedure A, **ethyl 3-amino-4-pyrazolecarboxylate 1a (39 mg, 0.25 mmol)**, **2d (104 mg, 1.5 equiv)** and **TsOH.H$_2$O (48 mg, 1 equiv)** were used. Purification by silica gel chromatography, using CH$_2$Cl$_2$:MeOH (gradient 0% MeOH up to 1.5%) as the eluant system, to afford **3d (57 mg, 73% yield)** as a white solid.

^{1}H NMR (600 MHz, CDCl$_3$) δ 9.77 (s, 1H), 8.16 (s, 1H), 7.66 – 7.60 (m, 2H), 7.09 – 7.05 (m, 2H), 6.21 (d, *J* = 2.4 Hz, 1H), 4.39 (q, *J* = 7.1 Hz, 2H), 3.90 (s, 3H), 1.42 (t, *J* = 7.1 Hz, 3H). **^{13}C NMR (151 MHz, CDCl$_3$) δ** 163.9, 162.8, 156.3, 149.2, 144.3, 142.9, 128.0, 123.6, 115.4, 97.5, 97.0, 61.0, 55.8, 14.5. **HRMS** (ESI) *m/z*: [M+H]$^+$ calcd for $C_{16}H_{16}N_3O_4$ 314.1136 ; found 314.1163.

ethyl 7-oxo-5-(4-(trifluoromethyl)phenyl)-4,7-dihydropyrazolo[1,5-*a*]pyrimidine-3-carboxylate (3e):

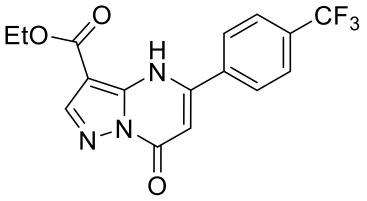

According to the general procedure A, **ethyl 3-amino-4-pyrazolecarboxylate 1a (39 mg, 0.25 mmol)**, **2e (119 mg, 1.5 equiv)** and **TsOH.H$_2$O (48 mg, 1 equiv)** were used. Purification by silica gel chromatography, using CH$_2$Cl$_2$:MeOH (gradient 0% MeOH up to 2%) as the eluant system, to afford **3e (68 mg, 77% yield)** as a white solid.

^{1}H NMR (600 MHz, CDCl$_3$) δ 9.86 (s, 1H), 8.21 (s, 1H), 7.86 (d, *J* = 8.5 Hz, 2H), 7.83 (d, *J* = 8.5 Hz, 2H), 6.30 (d, *J* = 2.5 Hz, 1H), 4.40 (q, *J* = 7.2 Hz, 2H), 1.42 (t, *J* = 7.1 Hz, 3H). **^{13}C NMR (151 MHz, CDCl$_3$) δ** 163.9, 155.8, 148.0, 144.3, 143.1, 135.2, 134.0 (q, *J* = 33.6 Hz), 127.1, 127.0 (q, *J* = 3.9 Hz), 123.5 (q, *J* = 272.9 Hz), 99.4, 98.0, 61.2, 14.5. **^{19}F NMR (565 MHz, CDCl$_3$) δ** -63.10. **HRMS** (ESI) *m/z*: [M+H]$^+$ calcd for $C_{16}H_{13}F_3N_3O_3$ 352.0904 ; found 352.0922.

ethyl 7-oxo-5-(p-tolyl)-4,7-dihydropyrazolo[1,5-*a*]pyrimidine-3-carboxylate (3f):

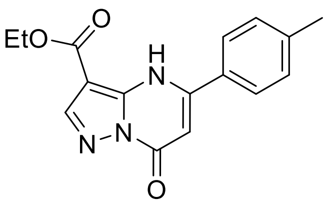

According to the general procedure A, **ethyl 3-amino-4-pyrazolecarboxylate 1a (39 mg, 0.25 mmol)**, **2f (99 mg, 1.5 equiv)** and **TsOH.H₂O (48 mg, 1 equiv)** were used. Purification by silica gel chromatography, using CH_2Cl_2:MeOH (gradient 0% MeOH up to 2%) as the eluant system, to afford **3f (57 mg, 77% yield)** as a white solid.

^{1}H NMR (600 MHz, $CDCl_3$) δ 9.80 (s, 1H), 8.17 (s, 1H), 7.70 – 7.51 (m, 2H), 7.38 (d, *J* = 8.1 Hz, 2H), 6.25 (d, *J* = 2.3 Hz, 1H), 4.39 (q, *J* = 7.2 Hz, 2H), 2.46 (s, 3H), 1.42 (t, *J* = 7.2 Hz, 3H). **^{13}C NMR (151 MHz, $CDCl_3$) δ** 163.9, 156.3, 149.5, 144.3, 143.0, 142.9, 130.7, 128.7, 126.3, 97.7, 97.6, 61.0, 21.6, 14.5. **HRMS** (ESI) *m/z*: [M-H]$^-$ calcd for $C_{16}H_{14}N_3O_3$ 296.1040; found 296.1031.

ethyl 5-(2-bromophenyl)-7-oxo-4,7-dihydropyrazolo[1,5-*a*]pyrimidine-3-carboxylate (3g):

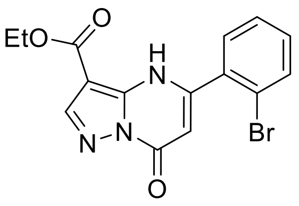

According to the general procedure A, **ethyl 3-amino-4-pyrazolecarboxylate 1a (39 mg, 0.25 mmol)**, **2g (123 mg, 1.5 equiv)** and **TsOH.H₂O (48 mg, 1 equiv)** were used. Purification by silica gel chromatography, using CH_2Cl_2:MeOH (gradient 0% MeOH up to 2%) as the eluant system, to afford **3g (79 mg, 87% yield)** as a white solid.

^{1}H NMR (600 MHz, $CDCl_3$) δ 9.72 (s, 1H), 8.21 (s, 1H), 7.76 (d, *J* = 8.0 Hz, 1H), 7.50 (d, *J* = 4.1 Hz, 2H), 7.47 – 7.41 (m, 1H), 6.09 (d, *J* = 2.3 Hz, 1H), 4.37 (q, *J* = 7.1 Hz, 2H), 1.39 (t, *J* = 7.2 Hz, 3H). **^{13}C NMR (151 MHz, $CDCl_3$) δ** 163.6, 155.9, 149.1, 143.8, 143.2, 134.3, 133.2, 132.7, 131.0, 128.4, 121.4, 101.8, 97.8, 61.1, 14.5. **HRMS** (ESI) *m/z*: [M+H]$^+$ calcd for $C_{15}H_{13}BrN_3O_3$ 362,0135 ; found 362,0069.

ethyl 5-ethyl-7-oxo-4,7-dihydropyrazolo[1,5-*a*]pyrimidine-3-carboxylate (3h):

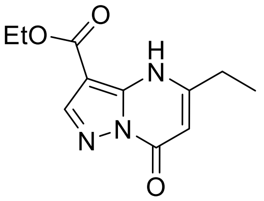

According to the general procedure A, **ethyl 3-amino-4-pyrazolecarboxylate 1a (39 mg, 0.25 mmol)**, **2h (61 mg, 1.2 equiv)** and **TsOH.H₂O (48 mg, 1 equiv)** were used. Purification by silica gel chromatography, using CH_2Cl_2:MeOH (gradient 0% MeOH up to 1.5%) as the eluant system, to afford **3h (45 mg, 77% yield)** as a white solid.

^{1}H NMR (400 MHz, $CDCl_3$) δ 9.41 (s, 1H), 8.13 (s, 1H), 5.87 (d, *J* = 2.3 Hz, 1H), 4.37 (q, *J* = 7.1 Hz, 2H), 2.70 (q, *J* = 7.6 Hz, 2H), 1.40 (t, *J* = 7.1 Hz, 3H), 1.37 (t, *J* = 7.6 Hz, 3H). **^{13}C NMR (100 MHz, $CDCl_3$) δ** 163.8, 156.2, 153.9, 144.2, 142.8, 98.6, 97.1, 61.0, 26.7, 14.5, 11.9. **HRMS** (ESI) *m/z*: [M-H]$^-$ calcd for $C_{11}H_{12}N_3O_3$ 234.0884; found 234.0865.

ethyl 5-isopropyl-7-oxo-4,7-dihydropyrazolo[1,5-*a*]pyrimidine-3-carboxylate (3i):

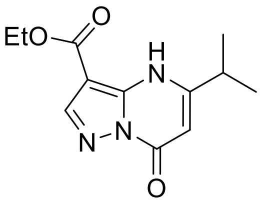

According to the general procedure A, **ethyl 3-amino-4-pyrazolecarboxylate 1a (39 mg, 0.25 mmol)**, **2i (81 mg, 1.5 equiv)** and **TsOH.H₂O (48 mg, 1 equiv)** were used. Purification by silica gel chromatography, using CH_2Cl_2:MeOH (gradient 0% MeOH up to 1.5%) as the eluant system, to afford **3i (61 mg, 90% yield)** as a white solid.

^{1}H NMR (600 MHz, $CDCl_3$) δ 9.44 (s, 1H), 8.12 (s, 1H), 5.88 (d, *J* = 2.4 Hz, 1H), 4.37 (q, *J* = 7.2 Hz, 2H), 2.89 (hept, *J* = 6.9 Hz, 1H), 1.40 (t, *J* = 7.2 Hz, 3H), 1.37 (d, *J* = 7.0 Hz, 6H). **^{13}C NMR (151 MHz, $CDCl_3$) δ** 163.9, 157.9, 156.4, 144.2, 142.8, 97.3, 97.1, 61.0, 32.5, 21.3, 14.5. **HRMS** (ESI) *m/z*: [M-H]$^-$ calcd for $C_{12}H_{14}N_3O_3$ 248.1040; found 248.1026.

ethyl 7-oxo-5-(1-phenylethyl)-4,7-dihydropyrazolo[1,5-*a*]pyrimidine-3-carboxylate (3j):

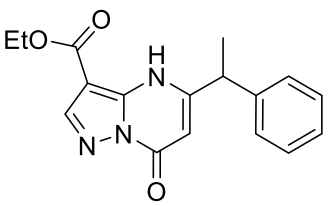

According to the general procedure A, **ethyl 3-amino-4-pyrazolecarboxylate 1a (39 mg, 0.25 mmol)**, **2i (104 mg, 1.5 equiv)** and **TsOH.H₂O (48 mg, 1 equiv)** were used. Purification by silica gel chromatography, using CH_2Cl_2:MeOH (gradient 0% MeOH up to 1.5%) as the eluant system, to afford **3j (65 mg, 84% yield)** as a white solid.

^{1}H NMR (600 MHz, $CDCl_3$) δ 9.16 (s, 1H), 8.11 (s, 1H), 7.43 (t, *J* = 7.5 Hz, 2H), 7.36 (t, *J* = 7.4 Hz, 1H), 7.32 – 7.28 (m, 2H), 6.03 (d, *J* = 2.6 Hz, 1H), 4.27 (q, *J* = 7.2 Hz, 2H), 4.08 (q, *J* = 7.2 Hz, 1H), 1.72

(d, *J* = 7.2 Hz, 3H), 1.30 (t, *J* = 7.2 Hz, 3H). **^{13}C NMR (151 MHz, CDCl$_3$) δ** 163.2, 156.2, 155.3, 143.9, 143.1, 139.6, 129.8, 128.6, 127.6, 98.5, 97.3, 60.8, 43.1, 19.6, 14.5. **HRMS** (ESI) *m/z*: $[M+H]^+$ calcd for $C_{17}H_{18}N_3O_3$ 312.1343; found 312.1371.

ethyl 5-(naphthalen-1-ylmethyl)-7-oxo-4,7-dihydropyrazolo[1,5-*a*]pyrimidine-3-carboxylate (3k):

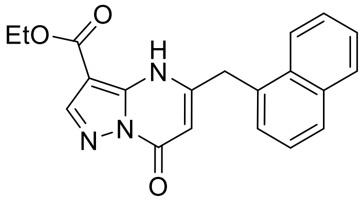

According to the general procedure A, **ethyl 3-amino-4-pyrazolecarboxylate 1a (39 mg, 0.25 mmol)**, **2k (94 mg, 1.2 equiv)** and **TsOH.H$_2$O (48 mg, 1 equiv)** were used. Purification by silica gel chromatography, using CH_2Cl_2:MeOH (gradient 0% MeOH up to 2%) as the eluant system, to afford **3k (74 mg, 85% yield)** as a white solid.

^{1}H NMR (400 MHz, CDCl$_3$) δ 9.24 (s, 1H), 8.08 (s, 1H), 7.96 – 7.90 (m, 2H), 7.91 – 7.85 (m, 1H), 7.60 – 7.47 (m, 4H), 5.97 (d, *J* = 2.3 Hz, 1H), 4.45 (s, 2H), 4.18 (q, *J* = 7.1 Hz, 2H), 1.21 (t, *J* = 7.1 Hz, 3H). **^{13}C NMR (100 MHz, CDCl$_3$) δ** 162.9, 156.1, 150.8, 143.6, 143.1, 134.4, 131.8, 129.8, 129.4, 129.3, 128.6, 127.5, 126.8, 125.8, 123.1, 99.8, 97.4, 60.8, 37.1, 14.4. **HRMS** (ESI) *m/z*: $[M+H]^+$ calcd for $C_{20}H_{18}N_3O_3$ 348.1343; found 348.1374.

ethyl 7-oxo-5-phenethyl-4,7-dihydropyrazolo[1,5-*a*]pyrimidine-3-carboxylate (3l):

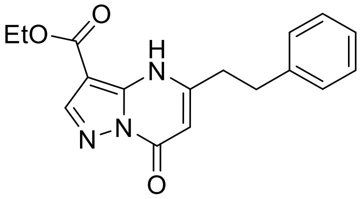

According to the general procedure A, **ethyl 3-amino-4-pyrazolecarboxylate (39 mg, 0.25 mmol)**, **2l (83 mg, 1.2 equiv)** and **TsOH.H$_2$O (48 mg, 1 equiv)** were used. Purification by silica gel chromatography, using CH_2Cl_2:MeOH (gradient 0% MeOH up to 1.5%) as the eluent system, to afford **3l (63 mg, 81% yield)** as a white solid.

^{1}H NMR (600 MHz, CDCl$_3$) δ 9.26 (s, 1H), 8.11 (s, 1H), 7.34 – 7.30 (m, 2H), 7.28 – 7.22 (m, 1H), 7.20 – 7.17 (m, 2H), 5.84 (d, *J* = 2.3 Hz, 1H), 4.34 (q, *J* = 7.2 Hz, 2H), 3.05 (t, *J* = 7.6 Hz, 2H), 2.96 (t, *J* = 7.6 Hz, 2H), 1.38 (t, *J* = 7.2 Hz, 3H). **^{13}C NMR (151 MHz, CDCl$_3$) δ** 163.6, 156.0, 151.8, 143.1, 142.8, 138.6, 129.2, 128.3, 127.3, 99.6, 97.2, 61.0, 35.3, 34.2, 14.5. **HRMS** (ESI) *m/z*: $[M-H]^-$ calcd for $C_{17}H_{16}N_3O_3$ 310.1197; found 310.1205.

ethyl 7-oxo-5-(thiophen-2-ylmethyl)-4,7-dihydropyrazolo[1,5-*a*]pyrimidine-3-carboxylat (3m):

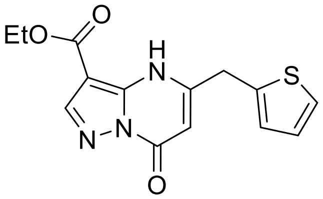

According to the general procedure A, **ethyl 3-amino-4-pyrazolecarboxylate 1a (39 mg, 0.25 mmol)**, **2m (81 mg, 1.2 equiv)** and **TsOH.H$_2$O (48 mg, 1 equiv)**. Purification by silica gel chromatography, using CH_2Cl_2:MeOH (gradient 0% MeOH up to 1.5%) as the eluent system, to afford **3m (66 mg, 87% yield)** as a beige solid.

^{1}H NMR (400 MHz, CDCl$_3$) δ 9.38 (s, 1H), 8.12 (s, 1H), 7.35 (dd, *J* = 4.7, 1.8 Hz, 1H), 7.10 – 7.02 (m, 2H), 5.95 – 5.92 (m, 1H), 4.31 (q, *J* = 7.1 Hz, 2H), 4.22 (s, 2H), 1.33 (t, *J* = 7.2 Hz, 3H). **^{13}C NMR (100 MHz, CDCl$_3$) δ** 163.3, 156.0, 149.9, 143.8, 143.1, 134.7, 128.3, 128.0, 126.9, 99.5, 97.5, 60.9, 33.4, 14.5. **HRMS** (ESI) *m/z*: $[M+H]^+$ calcd for $C_{14}H_{14}N_3O_3S$ 304.0751 ; found 304.0733.

ethyl 5-(but-3-en-1-yl)-7-oxo-4,7-dihydropyrazolo[1,5-*a*]pyrimidine-3-carboxylate (3n):

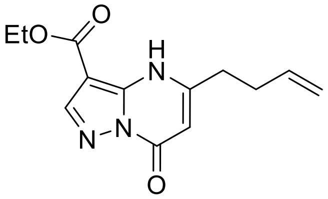

According to the general procedure A, **ethyl 3-amino-4-pyrazolecarboxylate 1a (39 mg, 0.25 mmol)**, **2n (68 mg, 1.2 equiv)** and **TsOH.H$_2$O (48 mg, 1 equiv)** were used. Purification by silica gel chromatography, using CH_2Cl_2:MeOH (gradient 0% MeOH up to 2%) as the eluent system, to afford **3n(49 mg, 75% yield)** as a white solid.

^{1}H NMR (600 MHz, CDCl$_3$) δ 9.50 (s, 1H), 8.12 (s, 1H), 5.90 – 5.81 (m, 2H), 5.20 – 5.14 (m, 2H), 4.36 (q, *J* = 7.1 Hz, 2H), 2.76 (t, *J* = 7.4 Hz, 2H), 2.50 (q, *J* = 6.9 Hz, 2H), 1.39 (t, *J* = 7.2 Hz, 3H). **^{13}C NMR (151 MHz, CDCl$_3$) δ** 163.8, 156.1, 152.1, 144.1, 142.8, 135.1, 118.0, 99.5, 97.2, 61.0, 32.7, 31.7, 14.5. **HRMS** (ESI) *m/z*: $[M-H]^-$ calcd for $C_{13}H_{14}N_3O_3$ 260.1040; found 260.1037.

ethyl 5-(but-3-yn-1-yl)-7-oxo-4,7-dihydropyrazolo[1,5-*a*]pyrimidine-3-carboxylate (3o):

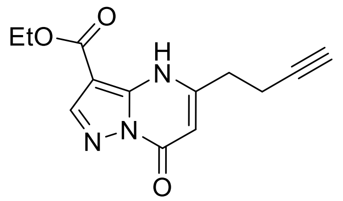

According to the general procedure A, **ethyl 3-amino-4-pyrazolecarboxylate 1a (39 mg, 0.25 mmol)**, **2o (68 mg, 1.2 equiv)** and **TsOH.H$_2$O (48 mg, 1 equiv)** were used. Purification by silica gel chromatography, using CH$_2$Cl$_2$:MeOH (gradient 0% MeOH up to 1.5%) as the eluent system, to afford **3o (50 mg, 77% yield)** as a white solid.

^{1}H NMR (600 MHz, CDCl$_3$) δ 10.03 (s, 1H), 8.14 (s, 1H), 5.88 (d, *J* = 2.3 Hz, 1H), 4.35 (q, *J* = 7.2 Hz, 2H), 2.87 (t, *J* = 6.6 Hz, 2H), 2.65 (td, *J* = 6.7, 2.8 Hz, 2H), 2.28 (t, *J* = 2.7 Hz, 1H), 1.39 (t, *J* = 7.2 Hz, 3H). **^{13}C NMR (151 MHz, CDCl$_3$) δ** 163.6, 156.1, 151.0, 144.0, 143.0, 99.8, 97.4, 81.4, 72.6, 60.9, 32.0, 17.6, 14.5. **HRMS** (ESI) *m/z*: [M-H]$^-$ calcd for $C_{13}H_{12}N_3O_3$ 258.0884; found 258.0878.

ethyl 5-(chloromethyl)-7-oxo-4,7-dihydropyrazolo[1,5-*a*]pyrimidine-3-carboxylate (3p):

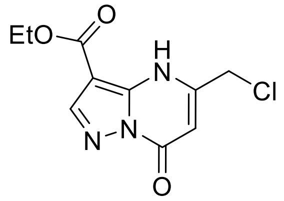

According to the general procedure A, **ethyl 3-amino-4-pyrazolecarboxylate 1a (39 mg, 0.25 mmol)**, **2p (67 mg, 1.2 equiv)** and **TsOH.H$_2$O (48 mg, 1 equiv)** were used. Purification by silica gel chromatography, using CH$_2$Cl$_2$:MeOH (gradient 0% MeOH up to 2%) as the eluent system, to afford **3p (55 mg, 86% yield)** as a white solid.

^{1}H NMR (400 MHz, CDCl$_3$) δ 9.87 (s, 1H), 8.18 (s, 1H), 6.02 (d, J = 2.4 Hz, 1H), 4.58 (s, 2H), 4.38 (q, J = 7.2 Hz, 2H), 1.41 (t, J = 7.2 Hz, 3H). **^{13}C NMR (100 MHz, CDCl$_3$) δ** 163.3, 155.4, 146.2, 143.8, 143.4, 99.9, 98.1, 61.2, 41.1, 14.5. **HRMS** (ESI) *m/z*: [M-H]$^-$ calcd for $C_{10}H_9ClN_3O_3$ 254.0338; found 254.0331.

ethyl 5-((benzyloxy)methyl)-7-oxo-4,7-dihydropyrazolo[1,5-a]pyrimidine-3-carboxylate (3q):

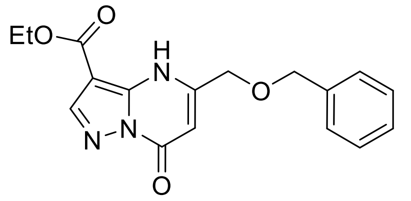

According to the general procedure A, **ethyl 3-amino-4-pyrazolecarboxylate 1a (39 mg, 0.25 mmol)**, **2q (88 mg, 1.2 equiv)** and **TsOH.H$_2$O (48 mg, 1 equiv)**. Purification by silica gel chromatography, using CH$_2$Cl$_2$:MeOH (gradient 0% MeOH up to 2%) as the eluent system, to afford **3q (65 mg, 79% yield)** as a white solid.

^{1}H NMR (600 MHz, CDCl$_3$) δ 9.97 (s, 1H), 8.17 (s, 1H), 7.48 – 7.33 (m, 5H), 5.83 (d, *J* = 1.1 Hz, 1H), 4.70 (s, 2H), 4.54 (s, 2H), 4.36 (q, *J* = 7.1 Hz, 2H), 1.35 (t, *J* = 7.2 Hz, 3H). **^{13}C NMR (151 MHz, CDCl$_3$) δ** 163.1, 155.8, 148.8, 143.5, 143.2, 136.0, 129.0, 128.8, 128.3, 97.7, 96.9, 73.9, 66.2, 60.9, 14.5. **HRMS** (ESI) *m/z*: [M+H]$^+$ calcd for $C_{17}H_{18}N_3O_4$ 328.1292; found 328.1323.

5-(4-methylbenzyl)pyrazolo[1,5-*a*]pyrimidin-7(4*H*)-one (3r):

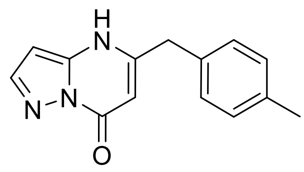

According to the general procedure A, **3-aminopyrazole 1b (21 mg, 0.25 mmol)**, **2a (83 mg, 1.2 equiv)** and **TsOH.H$_2$O (48 mg, 1 equiv)** were used. Purification by silica gel chromatography, using CH$_2$Cl$_2$:MeOH (gradient 1% MeOH up to 8%) as the eluant system, to afford **3r (41 mg, 69% yield)** as a light brown solid.

^{1}H NMR (400 MHz, MeOD) δ 7.87 (d, *J* = 2.2 Hz, 1H), 7.23 – 7.14 (m, 4H), 6.15 (d, *J* = 2.1 Hz, 1H), 5.64 (s, 1H), 3.93 (s, 2H), 2.32 (s, 3H). **^{13}C NMR (100 MHz, MeOD) δ** 159.6, 155.6, 144.6, 143.3, 138.3, 134.1, 130.6, 130.0, 96.3, 90.0, 39.5, 21.1. **HRMS** (ESI) *m/z*: [M+H]$^+$ calcd for $C_{14}H_{14}N_3O$ 240.1132; found 240.1161.

2-(*tert*-butyl)-5-(4-methylbenzyl)pyrazolo[1,5-*a*]pyrimidin-7(4*H*)-one (3t):

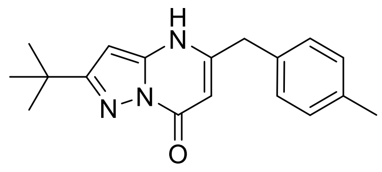

According to the general procedure A, **3-amino-5-*tert*-butylpyrazole 1d (36 mg, 97% purity, 0.25 mmol)**, **2a (83 mg, 1.2 equiv)** and **TsOH.H$_2$O (48 mg, 1 equiv)** were used. Purification by silica gel chromatography, using CH$_2$Cl$_2$:MeOH (gradient 1% MeOH up to 6%) as the eluant system, to afford **3t (68 mg, 92% yield)** as a light brown solid.

^{1}H NMR (400 MHz, CDCl$_3$) δ 10.95 (s, 1H), 7.14 – 7.05 (m, 4H), 5.89 (s, 1H), 5.60 (s, 1H), 3.94 (s, 2H), 2.29 (s, 3H), 1.22 (s, 9H). **^{13}C NMR (100 MHz, CDCl$_3$) δ** 166.2, 158.1, 152.7, 141.9, 137.4, 132.1, 129.8, 129.3, 96.1, 86.7, 39.0, 32.9, 30.2, 21.2. **HRMS** (ESI) *m/z*: [M-H]$^-$ calcd for $C_{18}H_{20}N_3O$ 294.1612; found 294.1602.

5-(4-methylbenzyl)-7-oxo-4,7-dihydropyrazolo[1,5-*a*]pyrimidine-3-carbonitrile (3u):

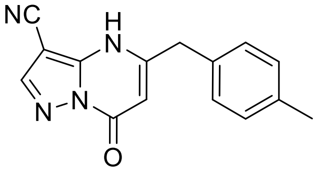

According to the general procedure A, **3-aminopyrazole-4-carbonitrile 1e (28 mg, 0.25 mmol)**, **2a (83 mg, 1.2 equiv)** and **TsOH.H$_2$O (48 mg, 1 equiv)** were used. The crude mixture was diluted with Et$_2$O (5 mL) and stirred for 15 minutes in an ice-bath. The resulting precipitate was filtered under vacuum and dried to afford **3u (58 mg, 88% yield)** as a white solid.

^{1}H NMR (400 MHz, DMSO) δ 13.46 (br, 1H), 8.21 (s, 1H), 7.20 (d, *J* = 7.9 Hz, 2H), 7.12 (d, *J* = 7.9 Hz, 2H), 5.62 (s, 1H), 3.83 (s, 2H), 2.27 (s, 4H). **^{13}C NMR (151 MHz, DMSO) δ** 159.7 (br), 156.6, 149.2 (br), 144.5, 135.6, 135.0, 129.1, 128.9, 114.5, 96.8, 75.1, 40.5 (br), 20.7. **HRMS** (ESI) *m/z*: [M+H]$^+$ calcd for $C_{15}H_{13}N_4O$ 265.1084; found 265.1071.

5-(4-methylbenzyl)-2-phenylpyrazolo[1,5-*a*]pyrimidin-7(4*H*)-one (3v):

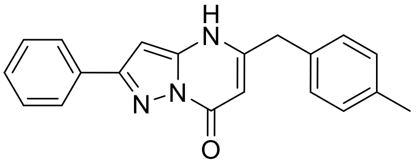

According to the general procedure A, **3-amino-5-phenylpyrazole 1f (41 mg, 0.25 mmol)**, **1a (83 mg, 1.2 equiv)** and **TsOH.H$_2$O (48 mg, 1 equiv)** were used. The crude mixture was diluted with Et$_2$O (5 mL) and stirred for 15 minutes in an ice-bath. The resulting precipitate was filtered under vacuum and dried to afford **3v (55 mg, 70% yield)** as a white solid.

^{1}H NMR (600 MHz, DMSO) δ 12.41 (d, *J* = 1.8 Hz, 1H), 7.98 – 7.95 (m, 2H), 7.50 – 7.44 (m, 2H), 7.43 – 7.36 (m, 1H), 7.25 (d, *J* = 8.1 Hz, 2H), 7.17 (d, *J* = 8.1 Hz, 2H), 6.58 (s, 1H), 5.57 (d, *J* = 1.9 Hz, 1H), 3.88 (s, 2H), 2.28 (s, 3H). **^{13}C NMR (151 MHz, DMSO) δ** 156.2, 153.0, 152.9, 142.8, 136.7, 133.6, 132.4, 129.2, 128.9, 128.8, 128.7, 126.2, 95.4, 85.8, 37.7, 20.7. **HRMS** (ESI) *m/z*: [M-H]$^-$ calcd for $C_{20}H_{16}N_3O$ 314,1299; found 314.1304.

ethyl 5-(4-methylbenzyl)-7-oxo-4,7-dihydropyrazolo[1,5-a]pyrimidine-2-carboxylate (3w):

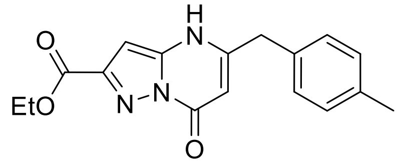

According to the general procedure A, **ethyl 3-amino-1*H*-pyrazole-5-carboxylate 1g (39 mg, 0.25 mmol)**, **2a (83 mg, 1.2 equiv)** and **TsOH.H$_2$O (48 mg, 1 equiv)** was used. Purification by silica gel chromatography, using CH_2Cl_2:MeOH (gradient 0% MeOH up to 4%) as the eluant system, to afford **3w (63 mg, 81% yield)** as a white solid.

^{1}H NMR (600 MHz, MeOD) δ 7.22 – 7.14 (m, 4H), 6.57 (s, 1H), 5.72 (s, 1H), 4.38 (q, *J* = 7.2 Hz, 2H), 3.93 (s, 2H), 2.32 (s, 3H), 1.38 (t, *J* = 7.1 Hz, 3H). **^{13}C NMR (151 MHz, MeOD) δ** 163.6, 159.2, 157.3, 147.5, 144.6, 138.3, 134.2, 130.6, 130.0, 96.9, 92.3, 62.6, 40.1, 21.1, 14.5. **HRMS** (ESI) *m/z*: [M-H]$^-$ calcd for $C_{17}H_{16}N_3O_3$ 310.1197; found 310.1195.

Pyrazolo[1,5-*a*]pyrimidin-5-ones 5 synthesis

General procedure B

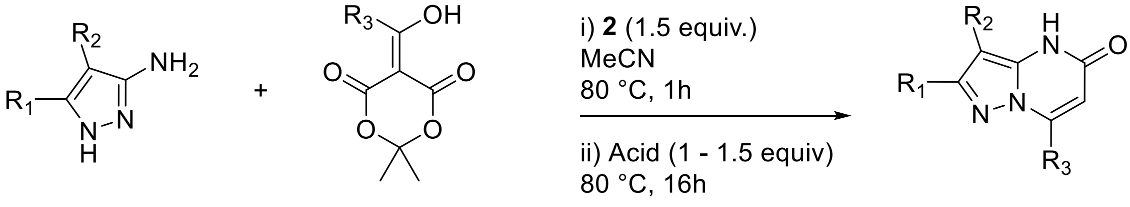

1 (0.25 mmol) **2** (1.5 equiv) **5**

In a sealable tube, the appropriate **aminopyrazole 1 (1 equiv)** and **acylated Meldrum's acid 2 (1.5 equiv)** were suspended in **MeCN (0.25 M)**. The tube was sealed and placed in a pre-heated oil bath at 80 °C for 1 hour. After cooling, consumption of the aminopyrazole was checked by TLC analysis.

The appropriate **acid (1 – 1.5 equiv)** was added, the tube was sealed, and placed in a pre-heated oil bath at 80 °C for 16 hours. After cooling to room temperature, completion was checked by TLC analysis, and the solvent was removed. The crude product was purified by silica gel chromatography to afford pure **7-substituted pyrazolo[1,5-*a*]pyrimidin-5-one 5**.

General rules and tips

(1) Reactions involving acylated Meldrum's acids **2** bearing secondary aryl substituents typically reach full conversion when 1.5 equivalents of **TsOH.H$_2$O** are used.
(2) In cases where degradation occurs during the ring-closing step, screening alternative acids or switching to bases (e.g., DIPEA) can prove effective.
(3) If a precipitate is observed at the end of the reaction, addition of Et$_2$O often enhances precipitation, enabling easier isolation of the pure product without silica gel chromatography.
(4) Most target compounds **5** can be visualized by TLC using a heptane:EtOAc eluant system. However, when this solvent system is applied to silica gel chromatography, significant tailing and poor separation are usually observed. A CH$_2$Cl$_2$:MeOH gradient is therefore recommended for optimal purification.

ethyl 7-(4-methylbenzyl)-5-oxo-4,5-dihydropyrazolo[1,5-*a*]pyrimidine-3-carboxylate (5a):

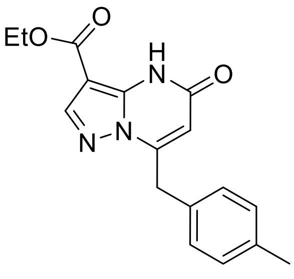

According to the general procedure B, **ethyl 3-amino-4-pyrazolecarboxylate 1a (39 mg, 0.25 mmol)**, **2a (104 mg, 1.5 equiv)** and **TsOH·H$_2$O (48 mg, 1 equiv)** were used. Purification by silica gel chromatography, using hept:EtOAc (6:4 up to 1:2) as the eluant system, to afford **MD1027 (73 mg, 94% yield)** as a white solid.

^{1}H NMR (400 MHz, CDCl$_3$) δ 9.51 (s, 1H), 8.04 (s, 1H), 7.21 – 7.14 (m, 4H), 5.73 (t, *J* = 1.3 Hz, 1H), 4.36 (q, *J* = 7.1 Hz, 2H), 4.22 (d, *J* = 1.2 Hz, 2H), 2.35 (s, 3H), 1.38 (t, *J* = 7.1 Hz, 3H). **^{13}C NMR (100 MHz, CDCl$_3$) δ** 162.7, 159.1, 152.6, 143.0, 142.6, 137.7, 130.8, 129.8, 129.6, 106.3, 96.7, 60.9, 35.9, 21.2, 14.6. **HRMS** (ESI) *m/z*: [M+H]$^+$ calcd for $C_{17}H_{18}N_3O_3$ 312.1343; found 312.1355.

2.5 mmol scale:

In a round-bottom flask equipped with a reflux condenser, **ethyl 3-amino-4-pyrazolecarboxylate 1a (388 mg, 2.5 mmol) and MD1015 (1036 mg, 1.5 equiv)** were suspended in **MeCN (10 mL)**. The mixture was placed in a pre-heated oil bath at 80 °C for 1 hour. After cooling, consumption of the aminopyrazole was checked by TLC analysis, and **TsOH·H$_2$O (476 mg, 1 equiv)** was added. The mixture was then heated at 80 °C for an additional 16 hours. After cooling to room temperature, completion was checked by TLC analysis. The crude mixture was diluted with Et$_2$O (50 mL) and stirred for 15 minutes in an ice bath. The resulting precipitate was filtered under vacuum and dried to afford **MD1027 (665 mg, 85% yield)** as a white solid.

With β-ketoamide 4a:

4a (67 mg, 0.20 mmol) was suspended in **MeCN (1 mL)** and **TsOH·H_2O (39 mg, 1 equiv)** was added, the tube was sealed, and placed in a pre-heated bath at 80 °C for 16 hours. After cooling to room temperature, completion was checked by TLC analysis, and the solvent was removed. The crude product was purified by silica gel chromatography using CH_2Cl_2:MeOH (gradient 0% MeOH up to 1%) as the eluant system, to afford **5a (56 mg, 88% yield)** as a white solid.

ethyl 5-oxo-7-phenyl-4,5-dihydropyrazolo[1,5-*a*]pyrimidine-3-carboxylate (5b):

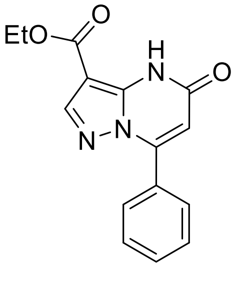

According to the general procedure B, **ethyl 3-amino-4-pyrazolecarboxylate 1a (39 mg, 0.25 mmol)**, **2b (94 mg, 1.5 equiv)** and **TsOH.H_2O (72 mg, 1.5 equiv)** were used. Purification by silica gel chromatography, using CH_2Cl_2:MeOH (gradient 0% MeOH up to 1.5%) as the eluant system, to afford **5b (53 mg, 75% yield)** as a white solid.

^1H NMR (400 MHz, $CDCl_3$) δ 9.71 (s, 1H), 8.06 (s, 1H), 7.80 (dd, *J* = 8.0, 1.6 Hz, 2H), 7.64 – 7.49 (m, 3H), 6.21 (s, 1H), 4.39 (q, *J* = 7.2 Hz, 2H), 1.40 (t, *J* = 7.1 Hz, 3H). **^{13}C NMR (100 MHz, $CDCl_3$) δ** 162.7, 159.0, 150.6, 143.5, 143.2, 131.6, 129.8, 129.5, 128.9, 106.9, 96.6, 61.0, 14.6. **HRMS** (ESI) *m/z*: $[M+H]^+$ calcd for $C_{15}H_{14}N_3O_3$ 284.1030; found 284.1051.

ethyl 7-(4-fluorophenyl)-5-oxo-4,5-dihydropyrazolo[1,5-*a*]pyrimidine-3-carboxylate (5c):

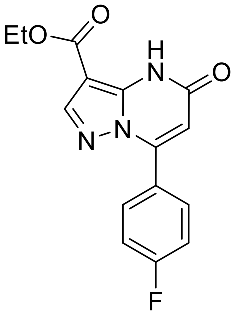

According to the general procedure B, **ethyl 3-amino-4-pyrazolecarboxylate 1a (39 mg, 0.25 mmol)**, **2c (100 mg, 1.5 equiv)** and **TsOH.H_2O (72 mg, 1.5 equiv)** were used. Purification by silica gel chromatography, using CH_2Cl_2:MeOH (gradient 0% MeOH up to 1.5%) as the eluant system, to afford **5c (45 mg, 60% yield)** as a white solid.

^1H NMR (600 MHz, $CDCl_3$) δ 9.72 (s, 1H), 8.05 (s, 1H), 7.86 – 7.79 (m, 2H), 7.23 (t, *J* = 8.6 Hz, 2H), 6.19 (s, 1H), 4.39 (q, *J* = 7.1 Hz, 2H), 1.40 (t, *J* = 7.1 Hz, 3H). **^{13}C NMR (151 MHz, $CDCl_3$) δ** 164.7 (d, *J* = 253.6 Hz), 162.7, 158.9, 149.5, 143.5, 143.2, 131.8 (d, *J* = 8.8 Hz), 125.8 (d, *J* = 3.3 Hz), 116.2 (d, *J* = 22.0 Hz), 106.8, 96.7, 61.0, 14.6. **^{19}F NMR (565 MHz, $CDCl_3$) δ** -107.31 – -107.38 (m). **HRMS** (ESI) *m/z*: $[M+H]^+$ calcd for $C_{15}H_{13}FN_3O_3$ 302.0936; found 302.0964.

ethyl 7-(4-methoxyphenyl)-5-oxo-4,5-dihydropyrazolo[1,5-*a*]pyrimidine-3-carboxylate (5d):

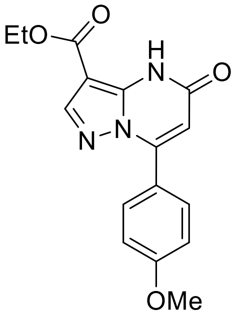

According to the general procedure B, **ethyl 3-amino-4-pyrazolecarboxylate 1a (39 mg, 0.25 mmol)**, **2d (104 mg, 1.5 equiv)** and **TsOH.H_2O (72 mg, 1.5 equiv)** were used. Purification by silica gel chromatography, using CH_2Cl_2:MeOH (gradient 0% MeOH up to 1.25%) as the eluant system, to afford **5d (68 mg, 87% yield)** as a light yellow solid.

^1H NMR (600 MHz, $CDCl_3$) δ 9.66 (br, 1H), 8.06 (s, 1H), 7.82 – 7.78 (m, 2H), 7.06 – 7.02 (m, 2H), 6.17 (s, 1H), 4.38 (q, *J* = 7.2 Hz, 2H), 3.89 (s, 3H), 1.40 (t, *J* = 7.2 Hz, 3H). **^{13}C NMR (151 MHz, $CDCl_3$) δ** 162.7, 162.3, 159.2, 150.3, 143.5, 143.1, 131.2, 121.9, 114.3, 105.7, 96.5, 60.9, 55.7, 14.6. **HRMS** (ESI) *m/z*: $[M+H]^+$ calcd for $C_{16}H_{16}N_3O_4$ 314.1136; found 314.1161.

ethyl 5-oxo-7-(4-(trifluoromethyl)phenyl)-4,5-dihydropyrazolo[1,5-*a*]pyrimidine-3-carboxylate (5e):

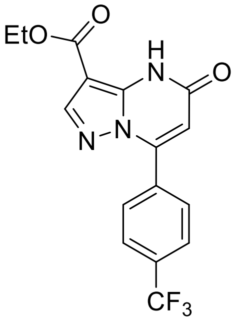

According to the general procedure B, **ethyl 3-amino-4-pyrazolecarboxylate 1a (39 mg, 0.25 mmol)**, **2e (119 mg, 1.5 equiv)** and **TsOH.H_2O (72 mg, 1.5 equiv)** were used. Purification by silica gel chromatography, using CH_2Cl_2:MeOH (gradient 0% MeOH up to 1%) as the eluant system, to afford **5e (44 mg, 50% yield)** as a light yellow solid.

^1H NMR (400 MHz, $CDCl_3$) δ 9.77 (br, 1H), 8.05 (s, 1H), 7.93 (d, *J* = 8.3 Hz, 2H), 7.81 (d, *J* = 8.3 Hz, 2H), 6.24 (s, 1H), 4.39 (q, *J* = 7.1 Hz, 2H), 1.40 (t, *J* = 7.1 Hz, 3H). **^{13}C NMR (100 MHz, $CDCl_3$) δ** 162.6, 158.7, 149.0, 143.5, 143.4, 133.4 (q, *J* = 32.6 Hz), 133.2, 130.0, 125.9 (q, *J* = 3.7 Hz), 123.7 (q, *J* = 272.2 Hz), 107.7, 96.9, 61.1, 14.6. **^{19}F NMR (376 MHz,

$CDCl_3$) δ -63.15. **HRMS** (ESI) *m/z*: $[M+2Na-H]^+$ calcd for $C_{16}H_{11}F_3N_3Na_2O_3$ 396.0543; found 396.0580.

ethyl 5-oxo-7-(p-tolyl)-4,5-dihydropyrazolo[1,5-*a*]pyrimidine-3-carboxylate (5f):

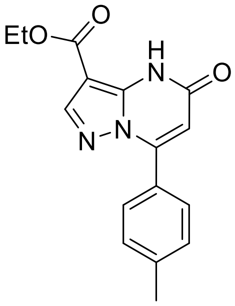

According to the general procedure B, **ethyl 3-amino-4-pyrazolecarboxylate 1a (39 mg, 0.25 mmol)**, **2f (99 mg, 1.5 equiv)** and **TsOH.H_2O (72 mg, 1.5 equiv)** were used. Purification by silica gel chromatography, using CH_2Cl_2:MeOH (gradient 0% MeOH up to 1%) as the eluant system, to afford **5f (58 mg, 78% yield)** as a white solid.

^1H NMR (400 MHz, $CDCl_3$) δ 9.67 (s, 1H), 8.05 (s, 1H), 7.73 – 7.67 (m, 2H), 7.38 – 7.32 (m, 2H), 6.19 (s, 1H), 4.38 (q, *J* = 7.2 Hz, 2H), 2.44 (s, 3H), 1.40 (t, *J* = 7.2 Hz, 3H). **^{13}C NMR (100 MHz, $CDCl_3$) δ** 162.8, 159.2, 150.7, 143.4, 143.1, 142.3, 129.6, 129.4, 126.9, 106.38, 96.5, 60.9, 21.7, 14.6. **HRMS** (ESI) *m/z*: $[M+H]^+$ calcd for $C_{16}H_{16}N_3O_3$ 298.1186; found 298.1205.

ethyl 7-ethyl-5-oxo-4,5-dihydropyrazolo[1,5-*a*]pyrimidine-3-carboxylate (5h):

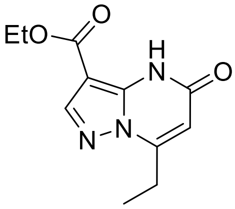

According to the general procedure B, **ethyl 3-amino-4-pyrazolecarboxylate 1a (39 mg, 0.25 mmol)**, **2h (76 mg, 1.5 equiv)** and **TsOH.H_2O (72 mg, 1.5 equiv)** were used. Purification by silica gel chromatography, using CH_2Cl_2:MeOH (gradient 0% MeOH up to 1%) as the eluant system, to afford **5h (53 mg, 90% yield)** as a white solid.

^1H NMR (400 MHz, $CDCl_3$) δ 9.58 (br, 1H), 8.03 (s, 1H), 5.98 (t, *J* = 1.3 Hz, 1H), 4.36 (q, *J* = 7.2 Hz, 2H), 2.99 (qd, *J* = 7.4, 1.2 Hz, 2H), 1.37 (q, *J* = 7.4 Hz, 6H). **^{13}C NMR (100 MHz, $CDCl_3$) δ** 162.7, 159.3, 154.4, 142.9, 142.6, 104.5, 96.6, 60.8, 23.7, 14.6, 10.8. **HRMS** (ESI) *m/z*: $[M+H]^+$ calcd for $C_{11}H_{14}N_3O_3$ 236.1030; found 236.1052.

ethyl 7-isopropyl-5-oxo-4,5-dihydropyrazolo[1,5-*a*]pyrimidine-3-carboxylate (5i):

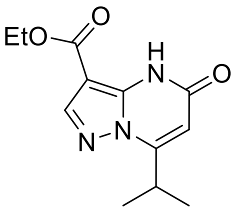

According to the general procedure B, **ethyl 3-amino-4-pyrazolecarboxylate 1a (39 mg, 0.25 mmol)**, **2i (81 mg, 1.5 equiv)** and **TsOH.H_2O (48 mg, 1 equiv)** were used. Purification by silica gel chromatography, using CH_2Cl_2:MeOH (gradient 0% MeOH up to 1.5%) as the eluant system, to afford **5i (58 mg, 93% yield)** as a white solid.

^1H NMR (600 MHz, $CDCl_3$) δ 9.55 (s, 1H), 8.03 (s, 1H), 5.98 (s, 1H), 4.36 (q, *J* = 7.2 Hz, 2H), 3.57 (hept, *J* = 6.7 Hz, 1H), 1.41 – 1.34 (m, 9H)**. ^{13}C NMR (151 MHz, $CDCl_3$) δ** 162.7, 159.5, 158.6, 142.8, 142.7, 102.9, 96.4, 60.8, 28.8, 20.5, 14.6. **HRMS** (ESI) *m/z*: $[M+H]^+$ calcd for $C_{12}H_{16}N_3O_3$ 250.1186; found 250.1213.

ethyl 5-oxo-7-(1-phenylethyl)-4,5-dihydropyrazolo[1,5-*a*]pyrimidine-3-carboxylate (5j):

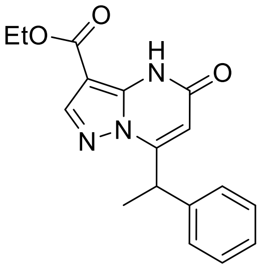

According to the general procedure B, **ethyl 3-amino-4-pyrazolecarboxylate 1a (39 mg, 0.25 mmol)**, **2j (104 mg, 1.5 equiv)** and **TsOH.H_2O (48 mg, 1 equiv)** were used. Purification by silica gel chromatography, using CH_2Cl_2:MeOH (gradient 0% MeOH up to 1%) as the eluant system, to afford **5j (72 mg, 93% yield)** as a white solid.

^1H NMR (600 MHz, $CDCl_3$) δ 9.57 (s, 1H), 7.99 (s, 1H), 7.34 (d, *J* = 4.2 Hz, 4H), 7.30 – 7.23 (m, 1H), 5.97 (s, 1H), 4.80 (q, *J* = 7.3 Hz, 1H), 4.34 (q, *J* = 7.1 Hz, 2H), 1.70 (d, *J* = 7.2 Hz, 3H), 1.36 (t, *J* = 7.1 Hz, 3H). **^{13}C NMR (151 MHz, $CDCl_3$) δ** 162.7, 159.4, 156.4, 142.8, 142.6, 140.4, 129.0, 127.9, 127.7, 104.9, 96.6, 60.8, 39.4, 19.5, 14.6. **HRMS** (ESI) *m/z*: $[M+H]^+$ calcd for $C_{17}H_{18}N_3O_3$ 312.1343; found 312.1364.

ethyl 7-(naphthalen-1-ylmethyl)-5-oxo-4,5-dihydropyrazolo[1,5-*a*]pyrimidine-3-carboxylate (5k):

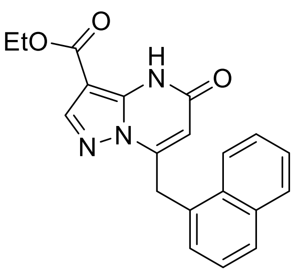

According to the general procedure B, **ethyl 3-amino-4-pyrazolecarboxylate 1a (39 mg, 0.25 mmol)**, **2k (118 mg, 1.5 equiv)** and **TsOH.H$_2$O (48 mg, 1 equiv)** were used. Purification by silica gel chromatography, using CH_2Cl_2:MeOH (gradient 0% MeOH up to 1%) as the eluant system, to afford **5k (84 mg, 97% yield)** as a white solid.

^{1}H NMR (400 MHz, CDCl$_3$) δ 9.56 (s, 1H), 8.13 (s, 1H), 7.95 – 7.84 (m, 2H), 7.81 – 7.75 (m, 1H), 7.55 – 7.40 (m, 4H), 5.42 (t, *J* = 1.5 Hz, 1H), 4.74 (d, *J* = 1.6 Hz, 2H), 4.38 (q, *J* = 7.2 Hz, 2H), 1.39 (t, *J* = 7.1 Hz, 3H). **^{13}C NMR (100 MHz, CDCl$_3$) δ** 162.7, 159.1, 151.9, 143.1, 142.6, 134.2, 131.9, 129.7, 129.2, 129.0, 128.8, 127.0, 126.3, 125.7, 123.5, 106.7, 96.9, 60.9, 33.4, 14.6. **HRMS** (ESI) *m/z*: [M+H]$^+$ calcd for $C_{20}H_{18}N_3O_3$ 348.1343; found 348.1357.

ethyl 5-oxo-7-phenethyl-4,5-dihydropyrazolo[1,5-*a*]pyrimidine-3-carboxylate (5l):

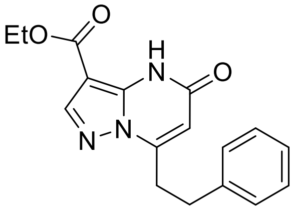

According to the general procedure B, **ethyl 3-amino-4-pyrazolecarboxylate 1a (39 mg, 0.25 mmol)**, **2l (104 mg, 1.5 equiv)** and **TsOH.H$_2$O (48 mg, 1 equiv)** were used. Purification by silica gel chromatography, using CH_2Cl_2:MeOH (gradient 0% MeOH up to 1%) as the eluant system, to afford **5l (74 mg, 95% yield)** as a white solid.

^{1}H NMR (600 MHz, CDCl$_3$) δ 9.56 (s, 1H), 8.05 (s, 1H), 7.33 – 7.28 (m, 2H), 7.25 – 7.19 (m, 3H), 5.90 (s, 1H), 4.37 (q, *J* = 7.1 Hz, 2H), 3.25 (t, *J* = 7.8 Hz, 2H), 3.10 (t, *J* = 7.8 Hz, 2H), 1.39 (t, *J* = 7.1 Hz, 3H). **^{13}C NMR (151 MHz, CDCl$_3$) δ** 162.7, 159.0, 152.0, 143.0, 142.7, 139.5, 128.9, 128.5, 126.9, 105.8, 96.6, 60.9, 32.7, 32.3, 14.6. **HRMS** (ESI) *m/z*: [M+H]$^+$ calcd for $C_{17}H_{18}N_3O_3$ 312.1343; found 312.1367.

ethyl 5-oxo-7-(thiophen-2-ylmethyl)-4,5-dihydropyrazolo[1,5-*a*]pyrimidine-3-carboxylate (5m):

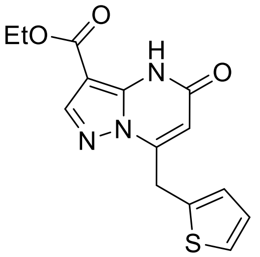

According to the general procedure B, **ethyl 3-amino-4-pyrazolecarboxylate 1a (39 mg, 0.25 mmol)**, **2m (101 mg, 1.5 equiv)** and **TsOH.H$_2$O (48 mg, 1 equiv)** were used. Purification by silica gel chromatography, using heptane:EtOAc (4:6 up to 2:1, tailing) as the eluent system, to afford **5m (69 mg, 91% yield)** as a white solid.

^{1}H NMR (600 MHz, CDCl$_3$) δ 9.53 (s, 1H), 8.06 (s, 1H), 7.27 (dd, *J* = 5.1, 1.3 Hz, 1H), 7.03 (dd, *J* = 3.5, 1.2 Hz, 1H), 7.01 (dd, *J* = 5.1, 3.4 Hz, 1H), 5.89 (t, *J* = 1.3 Hz, 1H), 4.48 (s, 2H), 4.37 (q, *J* = 7.2 Hz, 2H), 1.38 (t, *J* = 7.1 Hz, 3H). **^{13}C NMR (151 MHz, CDCl$_3$) δ** 162.6, 159.1, 151.3, 143.0, 142.6, 135.0, 128.3, 127.5, 126.0, 106.2, 96.8, 60.9, 30.3, 14.6. **HRMS** (ESI) *m/z*: [M+H]$^+$ calcd for $C_{14}H_{14}N_3O_3S$ 304.0751; found 304.0783.

ethyl 7-(but-3-en-1-yl)-5-oxo-4,5-dihydropyrazolo[1,5-*a*]pyrimidine-3-carboxylate (5n):

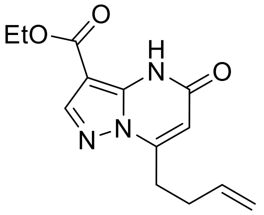

According to the general procedure B, **ethyl 3-amino-4-pyrazolecarboxylate 1a (39 mg, 0.25 mmol)**, **2n (85 mg, 1.5 equiv)** and **TsOH.H$_2$O (48 mg, 1 equiv)** were used. Purification by silica gel chromatography, using CH_2Cl_2:MeOH (gradient 0% MeOH up to 1%) as the eluant system, to afford **5n (59 mg, 90% yield)** as a white solid.

^{1}H NMR (400 MHz, CDCl$_3$) δ 9.57 (s, 1H), 8.03 (s, 1H), 5.97 (t, *J* = 1.2 Hz, 1H), 5.90 – 5.79 (m, 1H), 5.15 – 5.05 (m, 2H), 4.36 (q, *J* = 7.1 Hz, 2H), 3.05 (td, *J* = 7.5, 1.0 Hz, 2H), 2.60 – 2.50 (m, 2H), 1.38 (t, *J* = 7.1 Hz, 3H). **^{13}C NMR (100 MHz, CDCl$_3$) δ** 162.7, 159.1, 152.2, 142.9, 142.6, 135.7, 116.9, 105.7, 96.6, 60.9, 30.4, 29.7, 14.6. **HRMS** (ESI) *m/z*: [M+2Na-H]$^+$ calcd for $C_{13}H_{14}N_3Na_2O_3$ 306.0826; found 306.085.

ethyl 7-(but-3-yn-1-yl)-5-oxo-4,5-dihydropyrazolo[1,5-*a*]pyrimidine-3-carboxylate (5o):

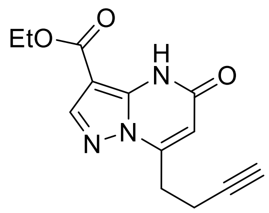

According to the general procedure B, **ethyl 3-amino-4-pyrazolecarboxylate 1a (39 mg, 0.25 mmol)**, **2o (85 mg, 1.5 equiv)** and **TsOH.H₂O (48 mg, 1 equiv)** were used. Purification by silica gel chromatography, using CH_2Cl_2:MeOH (gradient 0% MeOH up to 1%) as the eluant system, to afford **5o (61 mg, 94% yield)** as a white solid.

^{1}H NMR (600 MHz, $CDCl_3$) δ 9.55 (s, 1H), 8.02 (s, 1H), 6.08 (s, 1H), 4.37 (q, *J* = 7.2 Hz, 2H), 3.15 (t, *J* = 6.9 Hz, 2H), 2.74 (td, *J* = 7.0, 2.7 Hz, 2H), 2.05 (t, *J* = 2.7 Hz, 1H), 1.38 (t, *J* = 7.2 Hz, 3H). **^{13}C NMR (151 MHz, $CDCl_3$) δ** 162.6, 158.9, 150.3, 143.0, 142.7, 106.6, 96.7, 81.3, 71.0, 60.9, 29.7, 16.2, 14.6. **HRMS** (ESI) *m/z*: $[M+H]^+$ calcd for $C_{13}H_{14}N_3O_3$ 260.1030; found 260.1060.

ethyl 7-(chloromethyl)-5-oxo-4,5-dihydropyrazolo[1,5-*a*]pyrimidine-3-carboxylate (5p):

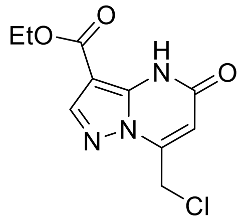

According to the general procedure B, **ethyl 3-amino-4-pyrazolecarboxylate 1a (39 mg, 0.25 mmol)**, **2p (83 mg, 1.5 equiv)** and **TsOH.H₂O (48 mg, 1 equiv)** were used. Purification by silica gel chromatography, using CH_2Cl_2:MeOH (gradient 0% MeOH up to 1%) as the eluant system, to afford **5p (57 mg, 89% yield)** as a white solid

^{1}H NMR (600 MHz, $CDCl_3$) δ 9.66 (s, 1H), 8.06 (s, 1H), 6.36 (t, *J* = 1.2 Hz, 1H), 4.81 (d, *J* = 1.1 Hz, 2H), 4.37 (q, *J* = 7.1 Hz, 2H), 1.39 (t, *J* = 7.1 Hz, 3H). **^{13}C NMR (151 MHz, $CDCl_3$) δ** 162.5, 158.7, 147.1, 143.4, 142.7, 107.1, 97.0, 61.1, 38.3, 14.6. **HRMS** (ESI) *m/z*: $[2M+K]^+$ calcd for $C_{20}H_{20}Cl_2KN_6O_6$ 549.0453; found 549.043.

2.5 mmol scale:

In a round-bottom flask equipped with a reflux condenser, **ethyl 3-amino-4-pyrazolecarboxylate 1a (388 mg, 2.5 mmol)** and **2p (828 mg, 1.5 equiv)** were suspended in **MeCN (10 mL)**. The mixture was placed in a pre-heated oil bath at 80 °C for 1 hour. After cooling, consumption of the aminopyrazole was checked by TLC analysis, and **TsOH·H₂O (476 mg, 1 equiv)** was added. The mixture was then heated at 80 °C for an additional 16 hours. After cooling to room temperature, completion was checked by TLC analysis. The crude mixture was diluted with Et_2O (25 mL) and stirred for 15 minutes in an ice bath. The resulting precipitate was filtered under vacuum and dried to afford **5p (485 mg, 76% yield)** as a white solid.

ethyl 7-((benzyloxy)methyl)-5-oxo-4,5-dihydropyrazolo[1,5-*a*]pyrimidine-3-carboxylate (5q):

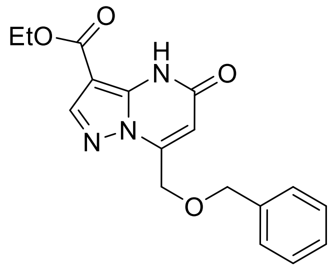

According to the general procedure B, **ethyl 3-amino-4-pyrazolecarboxylate 1a (39 mg, 0.25 mmol)**, **2q (110 mg, 1.5 equiv)** and **TsOH.H₂O (48 mg, 1 equiv)** were used. Purification by silica gel chromatography, using CH_2Cl_2:MeOH (gradient 0% MeOH up to 1%) as the eluent system, to afford **5q (60 mg, 73% yield)** as a white solid.

^{1}H NMR (600 MHz, $CDCl_3$) δ 9.53 (s, 1H), 7.99 (s, 1H), 7.38 (d, *J* = 4.4 Hz, 4H), 7.34 (q, *J* = 3.9 Hz, 1H), 6.37 (t, *J* = 1.7 Hz, 1H), 4.82 (d, *J* = 1.7 Hz, 2H), 4.73 (s, 2H), 4.36 (q, *J* = 7.1 Hz, 2H), 1.38 (t, *J* = 7.2 Hz, 3H). **^{13}C NMR (151 MHz, $CDCl_3$) δ** 162.5, 159.0, 149.2, 143.3, 142.7, 136.9, 128.8, 128.4, 128.0, 104.2, 96.6, 73.8, 64.8, 60.9, 14.6. **HRMS** (ESI) *m/z*: $[M+H]^+$ calcd for $C_{17}H_{18}N_3O_4$ 328.1292; found 328.1325.

7-(4-methylbenzyl)pyrazolo[1,5-*a*]pyrimidin-5(4*H*)-one (5r):

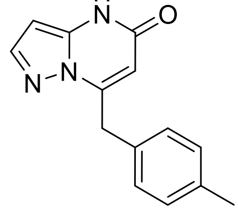

According to the general procedure B, **3-aminopyrazole 1b (21 mg, 0.25 mmol)**, **2a (104 mg, 1.5 equiv)** and **TFA (20 µL, 1 equiv)** were used. Purification by silica gel chromatography, using hept:EtOAc (1:1 up to 1:2) as the eluant system, to afford **5r (40 mg, 67% yield)** as a white solid.

^{1}H NMR (400 MHz, $CDCl_3$) δ 11.84 (br, 1H), 7.76 (d, *J* = 2.1 Hz, 1H), 7.23 – 7.12 (m, 4H), 5.97 (d, *J* = 2.1 Hz, 1H), 5.62 (t, *J* = 1.3 Hz, 1H), 4.27 (s, 2H), 2.35 (s, 3H). **^{13}C NMR (100 MHz, $CDCl_3$) δ** 162.8, 153.3, 143.4, 140.0, 137.4, 131.3, 129.8, 129.8, 103.9, 88.5, 36.1, 21.2. **HRMS** (ESI) *m/z*: $[M+H]^+$ calcd for $C_{14}H_{14}N_3O$ 240.1132; found 240.1144.

3-bromo-7-(4-methylbenzyl)pyrazolo[1,5-*a*]pyrimidin-5(4*H*)-one (5s):

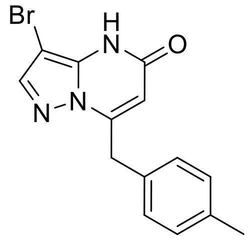

According to the general procedure B, **3-amino-4-bromopyrazole 1c (43 mg, 95% purity, 0.25 mmol), 2a (104 mg, 1.2 equiv)** and **TFA (20 µL, 1 equiv)** were used. Purification by silica gel chromatography, using CH_2Cl_2:MeOH (gradient 0% MeOH up to 1%) as the eluant system, to afford **5s (46 mg, 58% yield)** as a white solid.

^{1}H NMR (600 MHz, $CDCl_3$) δ 9.62 (br, 1H), 7.71 (s, 1H), 7.22 – 7.14 (m, 4H), 5.68 (t, *J* = 1.3 Hz, 1H), 4.21 (s, 2H), 2.35 (s, 3H). **^{13}C NMR (151 MHz, $CDCl_3$) δ** 160.4, 152.6, 142.7, 141.1, 137.8, 137.6, 131.0, 129.8, 129.7, 105.1, 35.5, 21.3. **HRMS** (ESI) *m/z*: [M+H]$^+$ calcd for $C_{14}H_{13}BrN_3O$ 318.0237; found 318.0268.

Note : Debrominated product **5r** was isolated in 33% yield.

2-(*tert*-butyl)-7-(4-methylbenzyl)pyrazolo[1,5-*a*]pyrimidin-5(4*H*)-one (5t):

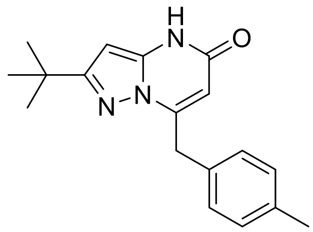

According to the general procedure B, **3-amino-5-*tert*-butylpyrazole 1d (36 mg, 97% purity, 0.25 mmol), 2a (104 mg, 1.5 equiv)** and **TsOH.H_2O (48 mg, 1 equiv)**. Purification by silica gel chromatography, using CH_2Cl_2:MeOH (gradient 0% MeOH up to 2.5%) as the eluant system, to afford **5t (64 mg, 87% yield)** as a white solid.

^{1}H NMR (400 MHz, $CDCl_3$) δ 11.73 (br, 1H), 7.23 (d, *J* = 8.2 Hz, 2H), 7.14 (d, *J* = 7.8 Hz, 2H), 5.84 (s, 1H), 5.53 (t, *J* = 1.2 Hz, 1H), 4.23 (s, 2H), 2.34 (s, 3H), 1.33 (s, 9H). **^{13}C NMR (100 MHz, $CDCl_3$) δ** 166.2, 162.8, 153.8, 140.0, 137.2, 131.7, 130.0, 129.6, 102.1, 85.5, 36.2, 33.0, 30.3, 21.2. **HRMS** (ESI) *m/z*: [M+H]$^+$ calcd for $C_{18}H_{22}N_3O$ 296.1758; found 296.1767.

7-(4-methylbenzyl)-5-oxo-4,5-dihydropyrazolo[1,5-*a*]pyrimidine-3-carbonitrile (5u):

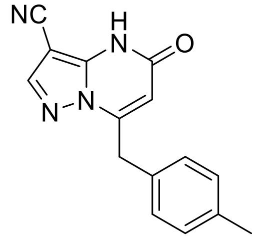

According to the general procedure B, **3-aminopyrazole-4-carbonitrile 1e (28 mg, 0.25 mmol), 2a (104 mg, 1.5 equiv)** and **TsOH.H_2O (48 mg, 1 equiv)** were used. Purification by silica gel chromatography, using CH_2Cl_2:MeOH (gradient 0% MeOH up to 4%) as the eluant system, to afford **5u (65 mg, 98% yield)** as a white solid.

^{1}H NMR (400 MHz, $CDCl_3$) δ 11.96 (br, 1H), 7.96 (s, 1H), 7.17 (s, 4H), 5.87 (t, *J* = 1.3 Hz, 1H), 4.23 (d, *J* = 1.3 Hz, 2H), 2.36 (s, 3H). **^{13}C NMR (100 MHz, $CDCl_3$) δ** 161.3, 153.3, 144.1, 143.7, 137.9, 130.3, 130.0, 129.7, 111.6, 106.7, 76.0, 36.1, 21.3. **HRMS** (ESI) *m/z*: [M+H]$^+$ calcd for $C_{15}H_{13}N_4O$ 265.1084; found 265.1109.

7-(4-methylbenzyl)-2-phenylpyrazolo[1,5-*a*]pyrimidin-5(4*H*)-one (5v):

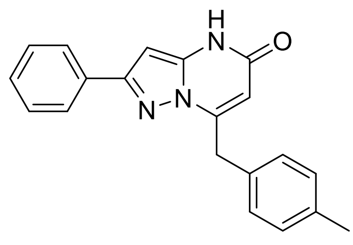

According to the general procedure B, **5-amino-3-phenylpyrazole 1f (40 mg, 0.25 mmol), 2a (104 mg, 1.5 equiv)** and **TsOH.H_2O (48 mg, 1 equiv)** were used. Purification by silica gel chromatography, using CH_2Cl_2:MeOH (gradient 0% MeOH up to 2%) as the eluant system, to afford **5v (60 mg, 76% yield)** as a white solid.

^{1}H NMR (400 MHz, DMSO) δ 12.15 (s, 1H), 7.98 – 7.89 (m, 2H), 7.51 – 7.43 (m, 2H), 7.42 – 7.36 (m, 1H), 7.35 (d, *J* = 8.0 Hz, 2H), 7.16 (d, *J* = 8.0 Hz, 2H), 6.32 (s, 1H), 5.66 (s, 1H), 4.24 (s, 2H), 2.27 (s, 3H). **^{13}C NMR (151 MHz, DMSO) δ** 159.6, 152.9, 151.1, 141.8, 136.1, 132.6, 132.2, 129.5, 129.1, 128.83, 128.78, 125.9, 104.1, 84.8, 34.9, 20.7. **HRMS** (ESI) *m/z*: [M+NH]$^+$ calcd for $C_{20}H_{18}N_3O$ 316.1445; found 316.1464.

Note: **MD1241** is insoluble in CH_2Cl_2 and MeOH individually, but a 1:1 mixture of both solvents allows its solubilization for solid deposit preparation for chromatography.

ethyl 7-(4-methylbenzyl)-5-oxo-4,5-dihydropyrazolo[1,5-*a*]pyrimidine-2-carboxylate (5w):

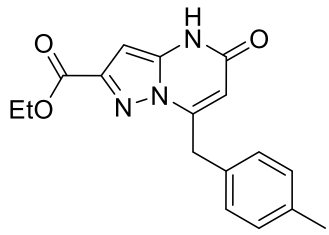

According to the general procedure B, **ethyl 3-amino-1*H*-pyrazole-5-carboxylate 1g (39 mg, 0.25 mmol), 2a (83 mg, 1.2 equiv)** and **TsOH.H$_2$O (48 mg, 1 equiv)** were used. Purification by silica gel chromatography, using CH_2Cl_2:MeOH (gradient 0% MeOH up to 2%) as the eluant system, to afford **5w (65 mg, 84% yield)** as a white solid.

^{1}H NMR (600 MHz, CDCl$_3$) δ 11.47 (s, 1H), 7.21 – 7.15 (m, 4H), 6.45 (s, 1H), 5.67 (t, J = 1.5 Hz, 1H), 4.44 (q, J = 7.2 Hz, 2H), 4.33 (d, J = 1.3 Hz, 2H), 2.36 (s, 3H), 1.41 (t, J = 7.2 Hz, 3H). **^{13}C NMR (151 MHz, CDCl$_3$) δ** 162.0, 161.8, 153.6, 146.5, 140.4, 137.6, 130.7, 130.0, 129.9, 106.5, 90.5, 61.8, 35.9, 21.3, 14.4. **HRMS** (ESI) *m/z*: [M+H]$^+$ calcd for $C_{17}H_{18}N_3O_3$ 312.1343; found 312.1378.

ethyl 4-benzyl-7-(4-methylbenzyl)-5-oxo-4,5-dihydropyrazolo[1,5-*a*]pyrimidine-3-carboxylate (5x):

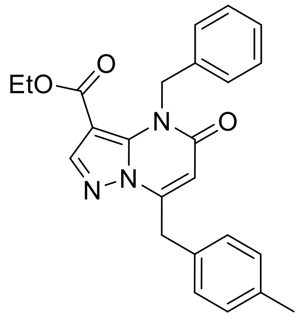

According to the general procedure B, **ethyl 3-(benzylamino)-1*H*-pyrazole-4-carboxylate 1h (62 mg, 0.25 mmol)**, **2a (104 mg, 1.5 equiv)** and **TsOH.H$_2$O (48 mg, 1 equiv)** were used. Purification by silica gel chromatography, using heptane:EtOAc (95:5 up to 80:20) as the eluent system, to afford **5x (88 mg, 88% yield)** as a white solid.

^{1}H NMR (600 MHz, CDCl$_3$) δ 8.13 (s, 1H), 7.28 – 7.14 (m, 9H), 5.99 (s, 2H), 5.83 (t, J = 1.3 Hz, 1H), 4.26 (q, J = 7.1 Hz, 2H), 4.23 (s, 2H), 2.35 (s, 3H), 1.29 (t, J = 7.2 Hz, 3H). **^{13}C NMR (151 MHz, CDCl$_3$) δ** 162.1, 160.1, 151.8, 146.0, 142.2, 137.5, 136.6, 131.0, 129.8, 129.7, 128.6, 127.49, 127.45, 105.9, 98.9, 61.0, 47.5, 36.2, 21.2, 14.4. **HRMS** (ESI) *m/z*: [M+H]$^+$ calcd for $C_{24}H_{23}N_3O_3$ 402.1812; found 402.1836.

Nitrogen containing pyrazolo[1,5-*a*]-pyrimidones synthesis.

ethyl 7-oxo-5-(piperidin-4-yl)-4,7-dihydropyrazolo[1,5-*a*]pyrimidine-3-carboxylate 4-methylbenzenesulfonate (7):

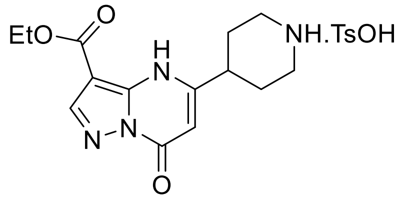

According to the general procedure A, **ethyl 3-amino-4-pyrazolecarboxylate (39 mg, 0.25 mmol)**, **acylated Meldrum's acid 6 (107 mg, 1.2 equiv)**, and **TsOH·H2O (96 mg, 2 equiv)** were used. After completion, the crude mixture was stirred for 15 minutes in an ice bath. The resulting precipitate was filtered under vacuum, washed with cold EtOH (2 x 2 mL) and Et_2O, and dried to afford **7 (77 mg, 67% yield)** as a white solid.

^{1}H NMR (600 MHz, D$_2$O) δ 8.30 (s, 1H), 7.70 – 7.62 (m, 2H), 7.32 (d, J = 8.3 Hz, 2H), 6.02 (s, 1H), 4.39 (q, J = 7.1 Hz, 2H), 3.67 – 3.55 (m, 2H), 3.26 – 3.09 (m, 3H), 2.36 (s, 3H), 2.34 – 2.28 (m, 2H), 2.03 – 1.90 (m, 2H), 1.38 (t, J = 7.2 Hz, 3H). **^{13}C NMR (151 MHz, D$_2$O) δ** 163.7, 158.7, 157.7, 144.5, 143.1, 142.4, 139.3, 129.3, 125.3, 97.8, 96.0, 61.7, 43.5, 36.7, 27.1, 20.4, 13.5. **HRMS** (ESI) *m/z*: [M-$C_7H_9O_3$]$^-$ calcd for $C_{14}H_{17}N_4O_3$ 289.1306; found 289.1306.

ethyl 5-oxo-7-(piperidin-4-yl)-4,5-dihydropyrazolo[1,5-*a*]pyrimidine-3-carboxylate 4-methylbenzenesulfonate (9):

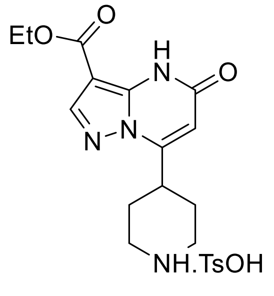

According to the general procedure B, **ethyl 3-amino-4-pyrazolecarboxylate (39 mg, 0.25 mmol)**, **acylated Meldrum's acid 6 (134 mg, 1.5 equiv)**, and **TsOH·H2O (96 mg, 2 equiv)** were used. After completion, the crude mixture was stirred for 15 minutes in an ice bath. The resulting precipitate was filtered under vacuum, washed with cold MeCN (2 x 2 mL) and Et_2O, and dried to afford **9 (99 mg, 86% yield)** as a white solid.

^{1}H NMR (600 MHz, D$_2$O) δ 8.22 (s, 1H), 7.66 (d, J = 8.3 Hz, 2H), 7.33 (d, J = 8.0 Hz, 2H), 6.16 (s, 1H), 4.38 (q, J = 7.2 Hz, 2H), 3.61 (d, J = 12.8 Hz, 2H), 3.55 (tt, J = 11.7, 3.1 Hz, 1H), 3.24 (td, J = 13.2, 3.0 Hz, 2H), 2.37 (s, 3H), 2.37 – 2.33 (m, 2H), 1.99 – 1.89 (m, 2H), 1.37 (t, J = 7.2 Hz, 3H). **^{13}C NMR (151 MHz, D$_2$O) δ** 163.7, 162.3, 154.4, 143.8, 142.4, 141.9, 139.4, 129.3,

125.3, 103.4, 96.9, 61.7, 43.5, 33.8, 25.9, 20.4, 13.6. **HRMS** (ESI) *m/z*: $[M\text{-}C_7H_7O_3]^+$ calcd for $C_{14}H_{19}N_4O_3$ 291.1452; found 291.1486.

ethyl 7-(1-(*tert*-butoxycarbonyl)piperidin-4-yl)-5-oxo-4,5-dihydropyrazolo[1,5-*a*]pyrimidine-3-carboxylate (10):

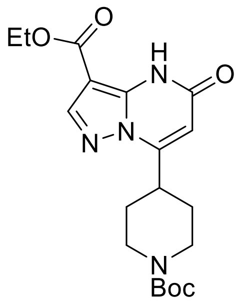

In a modification of the general procedure B, in a sealable tube, **ethyl 3-amino-4-pyrazolecarboxylate 1a (39 mg, 0.25 mmol)** and **acylated Meldrum's acid 6 (1.5 equiv)** were suspended in **MeCN (0.25 M)**. The tube was sealed and placed in a pre-heated oil bath at 80 °C for 1 hour. After cooling, consumption of the aminopyrazole was checked by TLC analysis.

DIPEA (2 equiv) was added, the tube was sealed, and placed in a pre-heated oil bath at 80 °C for 16 hours. After cooling to room temperature, completion was checked by TLC analysis, and the solvent was removed under reduced pressure. The residue was purified by silica gel chromatography using CH_2Cl_2:EtOAc (gradient 0% EtOAc up to 50%) as the eluant system, to afford **10 (77 mg, 79% yield)** as a white solid.

^{1}H NMR (400 MHz, $CDCl_3$) δ 9.62 (br, 1H), 8.02 (s, 1H), 5.93 (s, 1H), 4.36 (q, *J* = 7.1 Hz, 2H), 4.29 (br, 2H), 3.40 (tt, *J* = 12.1, 3.2 Hz, 1H), 3.05 – 2.75 (m, 2H), 2.08 (d, *J* = 13.4 Hz, 2H), 1.57 (qd, *J* = 12.5, 4.3 Hz, 2H), 1.48 (s, 9H), 1.38 (t, *J* = 7.1 Hz, 3H). **^{13}C NMR (151 MHz, $CDCl_3$) δ** 162.6, 159.3, 155.6, 154.8, 142.9, 142.7, 103.7, 96.7, 80.1, 60.9, 43.7 (br), 36.6, 29.8, 28.6, 14.6. **HRMS** (ESI) *m/z*: $[M+Na]^+$ calcd for $C_{19}H_{26}N_4O_5Na$ 413.1795; found 413.1826.

Mono-alkylated pyrazolo[1,5-*a*]pyrimidines synthesis.

ethyl 7-chloro-5-(4-methylbenzyl)pyrazolo[1,5-*a*]pyrimidine-3-carboxylate (11):

Chlorination conditions were adapted from a recent report.[13]

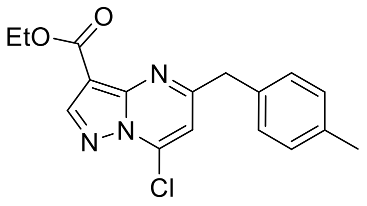

Starting **pyrazolo[1,5-*a*]pyrimidin-7-one 3a (156 mg, 0.5 mmol)** was suspended in **toluene (10 ml)**. **Triethylamine (105 µL, 1.25 equiv)** and **$POCl_3$ (233 µL, 5 equiv)** were added. The resulting mixture was stirred overnight at 100 °C, and TLC showed complete disappearance of the starting material. After cooling to room temperature, the mixture was poured into a stirring mixture of ice, aqueous saturated $NaHCO_3$, and CH_2Cl_2, and stirred for 15 minutes. The organic layer was separated, and the aqueous layer was extracted with CH_2Cl_2 (2 × 15 mL). The combined organic layers were washed with brine, dried over MgSO4, filtered, and the solvent was removed under reduced pressure. The residue was purified by silica gel chromatography using heptane:EtOAc (8:2 → 7:3) as the eluant system to afford **11 (141 mg, 86% yield)** as a light transparent oil which partially solidified over time in a freezer.

^{1}H NMR (400 MHz, $CDCl_3$) δ 8.59 (s, 1H), 7.20 (d, *J* = 8.2 Hz, 2H), 7.16 (d, *J* = 8.0 Hz, 2H), 6.87 (s, 1H), 4.43 (q, *J* = 7.1 Hz, 2H), 4.26 (s, 2H), 2.34 (s, 3H), 1.43 (t, *J* = 7.1 Hz, 3H). **^{13}C NMR (100 MHz, $CDCl_3$) δ** 165.3, 162.3, 148.8, 147.9, 139.9, 137.2, 133.8, 129.9, 129.5, 110.1, 104.1, 60.7, 44.7, 21.2, 14.6. **HRMS** (ESI) *m/z*: $[M+Na]^+$ calcd for $C_{17}H_{16}ClN_3O_2Na$ 352.0823; found 352.0813.

Note: The product reverts to the pyrazolo[1,5-*a*]pyrimidin-7-one within a few days at room temperature in open air and must be stored in a freezer.

ethyl 5-(4-methylbenzyl)pyrazolo[1,5-*a*]pyrimidine-3-carboxylate (12):

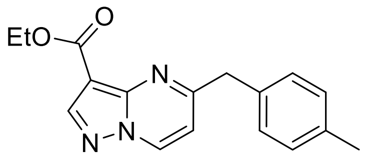

Chlorinated **11 (116 mg, 0.35 mmol)** was dissolved in **EtOAc (15 mL)** under a nitrogen atmosphere. **Pd/C (10 wt%, 38 mg, 0.1 equiv)** and **Et_3N (98 µL, 2 equiv)** were added successively. The mixture was cooled in an ice bath and bubbled with **hydrogen** for 30 min. The reaction mixture was stirred for 30 more minutes under a hydrogen atmosphere at 0°C, until TLC analysis confirmed complete consumption of the starting material. The catalyst was removed by filtration through Celite,

and the filtrate was washed with 1 M HCl (20 mL). The aqueous layer was extracted with EtOAc (2 × 20 mL), and the combined organic layers were washed with brine, dried over $MgSO_4$, and filtered. The solvent was removed under reduced pressure, and the crude product was purified by silica gel chromatography (heptane:EtOAc 6:4) to afford **12 (100 mg, 97% yield)** as a white solid.

^{1}H NMR (400 MHz, CDCl$_3$) δ 8.53 (d, *J* = 7.2 Hz, 1H), 8.50 (s, 1H), 7.22 – 7.17 (m, 2H), 7.14 (d, *J* = 8.0 Hz, 2H), 6.74 (d, *J* = 7.2 Hz, 1H), 4.42 (q, *J* = 7.1 Hz, 2H), 4.27 (s, 2H), 2.33 (s, 3H), 1.42 (t, *J* = 7.1 Hz, 3H). **^{13}C NMR (100 MHz, CDCl$_3$) δ** 165.7, 162.7, 147.9, 147.6, 137.0, 135.7, 134.3, 129.8, 129.4, 110.0, 102.7, 60.4, 44.9, 21.2, 14.6. **HRMS** (ESI) *m/z*: [M+Na]$^+$ calcd for $C_{17}H_{17}N_3O_2Na$ 318.1213; found 318.1231.

ethyl 7-benzyl-5-chloropyrazolo[1,5-*a*]pyrimidine-3-carboxylate (13):

Chlorination conditions were adapted from a recent report.[13]

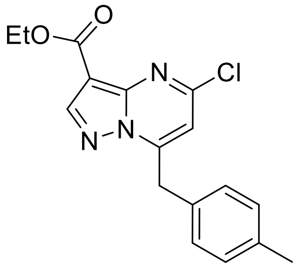

Starting **pyrazolo[1,5-*a*]pyrimidin-5-one 5a (156 mg, 0.5 mmol)** was suspended in **toluene (10 mL)**. **Triethylamine (105 µL, 1.25 equiv)** and **POCl$_3$ (233 µL, 5 equiv)** were added. The resulting mixture was stirred overnight at 90 °C, and TLC showed complete disappearance of the starting material. After cooling to room temperature, the mixture was poured into a stirring mixture of ice, aqueous saturated $NaHCO_3$, and CH_2Cl_2, and stirred for 15 minutes. The organic layer was separated, and the aqueous layer was extracted with CH_2Cl_2 (2 × 15 mL). The combined organic layers were washed with brine, dried over MgSO4, filtered, and the solvent was removed under reduced pressure. The residue was purified by silica gel chromatography using heptane:EtOAc (8:2) as the eluant system to afford **13 (140 mg, 85% yield)** as a transparent oil which solidified on standing.

^{1}H NMR (400 MHz, CDCl$_3$) δ 8.57 (s, 1H), 7.21 (s, 4H), 6.54 (t, *J* = 1.2 Hz, 1H), 4.46 (d, *J* = 1.1 Hz, 2H), 4.42 (q, *J* = 7.1 Hz, 2H), 2.38 (s, 3H), 1.42 (t, *J* = 7.1 Hz, 3H). **^{13}C NMR (100 MHz, CDCl$_3$) δ** 162.3, 154.3, 152.0, 147.7, 147.1, 137.9, 130.2, 130.0, 129.7, 109.7, 103.6, 60.7, 36.1, 21.3, 14.6. **HRMS** (ESI) *m/z*: [M+Na]$^+$ calcd for $C_{17}H_{16}ClN_3O_2Na$ 352.0823; found 352.0790.

ethyl 7-(4-methylbenzyl)pyrazolo[1,5-*a*]pyrimidine-3-carboxylate (14):

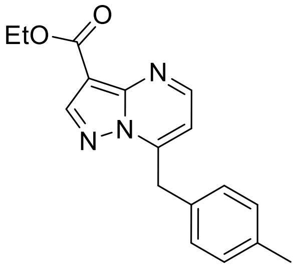

Chlorinated **14 (116 mg, 0.35 mmol)** was dissolved in **EtOAc (15 mL)** under a nitrogen atmosphere. **Pd/C (10 wt%, 38 mg, 0.1 equiv)** and **Et$_3$N (98 µL, 2 equiv)** were added successively. The mixture was cooled in an ice bath and bubbled with **hydrogen** for 30 min. The bath was then removed and the reaction mixture was stirred at room temperature under a hydrogen atmosphere for 20 h, until TLC analysis confirmed complete consumption of the starting material. The catalyst was removed by filtration through Celite, and the filtrate was washed with 1 M HCl (20 mL). The aqueous layer was extracted with EtOAc (2 × 20 mL), and the combined organic layers were washed with brine, dried over $MgSO_4$, and filtered. The solvent was removed under reduced pressure, and the crude product was purified by silica gel chromatography (heptane:EtOAc 3:2 → 1:1) to afford **14 (87 mg, 84% yield)** as a white solid.

^{1}H NMR (400 MHz, CDCl$_3$) δ 8.63 (d, *J* = 4.4 Hz, 1H), 8.61 (s, 1H), 7.25 – 7.15 (m, 4H), 6.66 – 6.56 (m, 1H), 4.50 (s, 2H), 4.45 (q, *J* = 7.1 Hz, 2H), 2.36 (s, 3H), 1.42 (t, *J* = 7.1 Hz, 3H). **^{13}C NMR (100 MHz, CDCl$_3$) δ** 162.8, 152.3, 150.7, 148.1, 147.3, 137.6, 131.0, 129.9, 129.7, 108.8, 103.5, 60.5, 36.1, 21.3, 14.7. **HRMS** (ESI) *m/z*: [M+Na]$^+$ calcd for $C_{17}H_{17}N_3O_2Na$ 318.1213; found 318.1236.

Intermediates 2a' and 4a synthesis.

ethyl 3-oxo-4-(*p*-tolyl)butanoate (2a'):

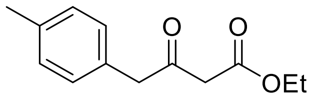

In a sealable tube, **2a (0.5 mmol)** was suspended in **EtOH (1 mL)**. The tube was sealed, and placed in a pre-heated oil bath at 80 °C for 1 hour. After cooling to room temperature, completion was checked by TLC analysis, and the solvent was removed under reduced pressure. The β-ketoester **2a' (109 mg, 99% yield)** was obtained as a colorless oil and used in the next step without any further purification.

^{1}H NMR (600 MHz, DMSO) δ 7.12 (d, *J* = 7.9 Hz, 2H), 7.06 (d, *J* = 8.0 Hz, 2H), 4.07 (q, *J* = 7.1 Hz, 2H), 3.80 (s, 2H), 3.62 (s, 2H), 2.28 (s, 3H), 1.17 (t, *J* = 7.1 Hz, 3H). **^{13}C NMR (151 MHz, DMSO) δ** 201.38, 167.13, 135.81, 130.92, 129.59, 128.93, 60.50, 48.43, 48.37, 20.66, 13.97. Spectral data were in accordance with published data.[14]

ethyl 3-(3-oxo-4-(*p*-tolyl)butanamido)-1*H*-pyrazole-4-carboxylate (4a):

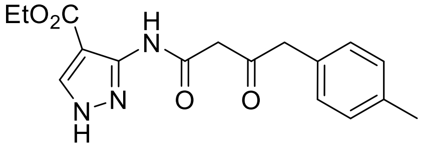

In a sealable tube, ethyl 3-amino-4-pyrazolecarboxylate **1a (1 equiv)** and **2a (1.5 equiv)** were suspended in **MeCN (1 mL)**. The tube was sealed and placed in a pre-heated oil bath at 80 °C for 1 hour. After cooling, consumption of the aminopyrazole was checked by TLC analysis and the solvent was removed under reduced pressure. The crude product was purified by silica gel chromatography, using CH_2Cl_2:MeOH (gradient 0% MeOH up to 1.5%) as the eluant system, to afford **4a (67 mg, 81% yield)** as a white solid.

^{1}H NMR (600 MHz, DMSO) δ 10.10 (s, 1H), 7.86 (s, 1H), 7.20 (d, J = 1.6 Hz, 1H), 7.15 (d, J = 8.0 Hz, 2H), 7.08 (d, J = 7.8 Hz, 2H), 4.29 – 4.17 (m, 2H), 3.48 (d, J = 13.6 Hz, 1H), 3.37 (d, J = 13.7 Hz, 1H), 3.07 (dd, J = 16.3, 1.7 Hz, 1H), 2.39 (dd, J = 16.3, 1.1 Hz, 1H), 2.26 (s, 3H), 1.25 (t, J = 7.1 Hz, 3H). **^{13}C NMR (151 MHz, DMSO) δ** 165.89, 161.81, 140.99, 139.88, 135.93, 131.96, 130.47, 128.64, 97.66, 84.98, 59.56, 43.06, 42.04, 20.68, 14.38. **HRMS** (ESI) *m/z*: $[M-H]^-$ calcd for $C_{17}H_{18}N_3O_4$ 328.1303; found 328.1284.

NMR spectra of synthesized compounds

^{1}H NMR (400 MHz, $CDCl_3$) (1h)

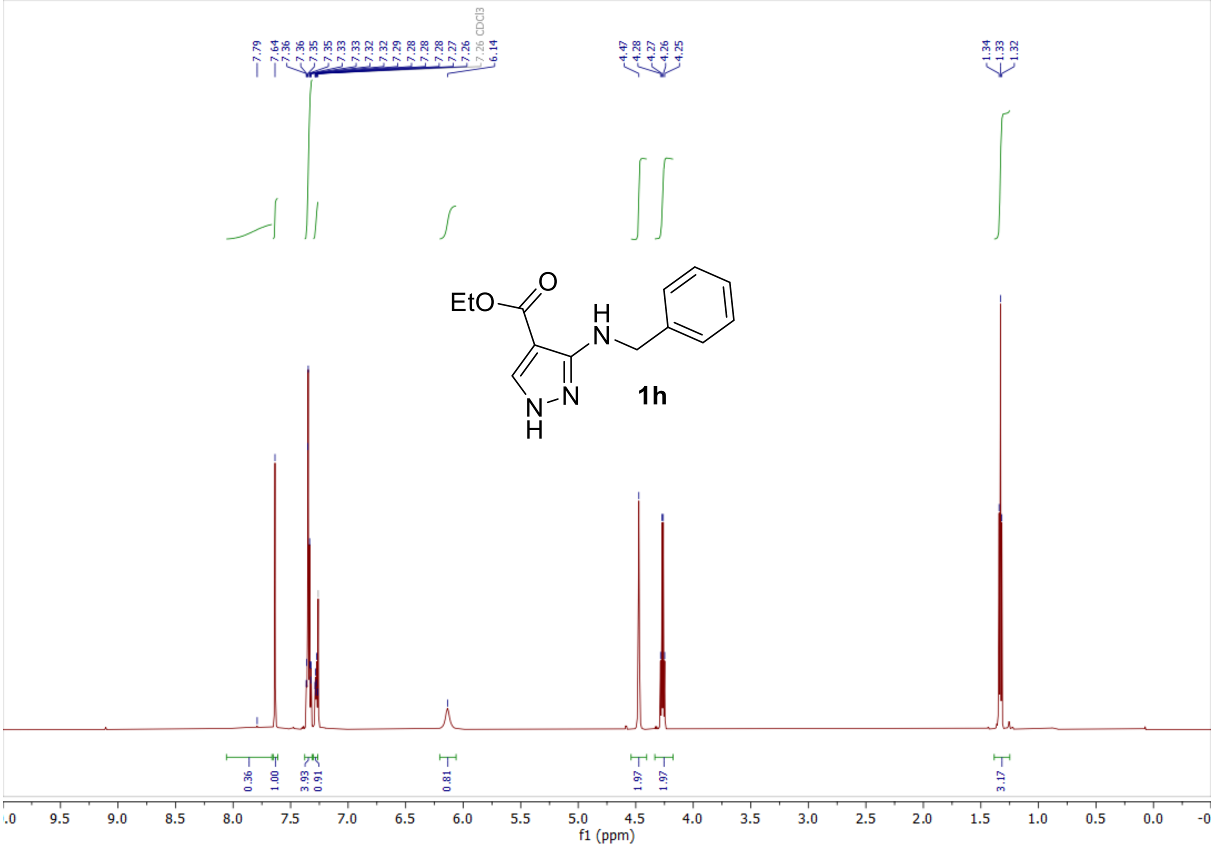

^{13}C NMR (100 MHz, $CDCl_3$) (1h)

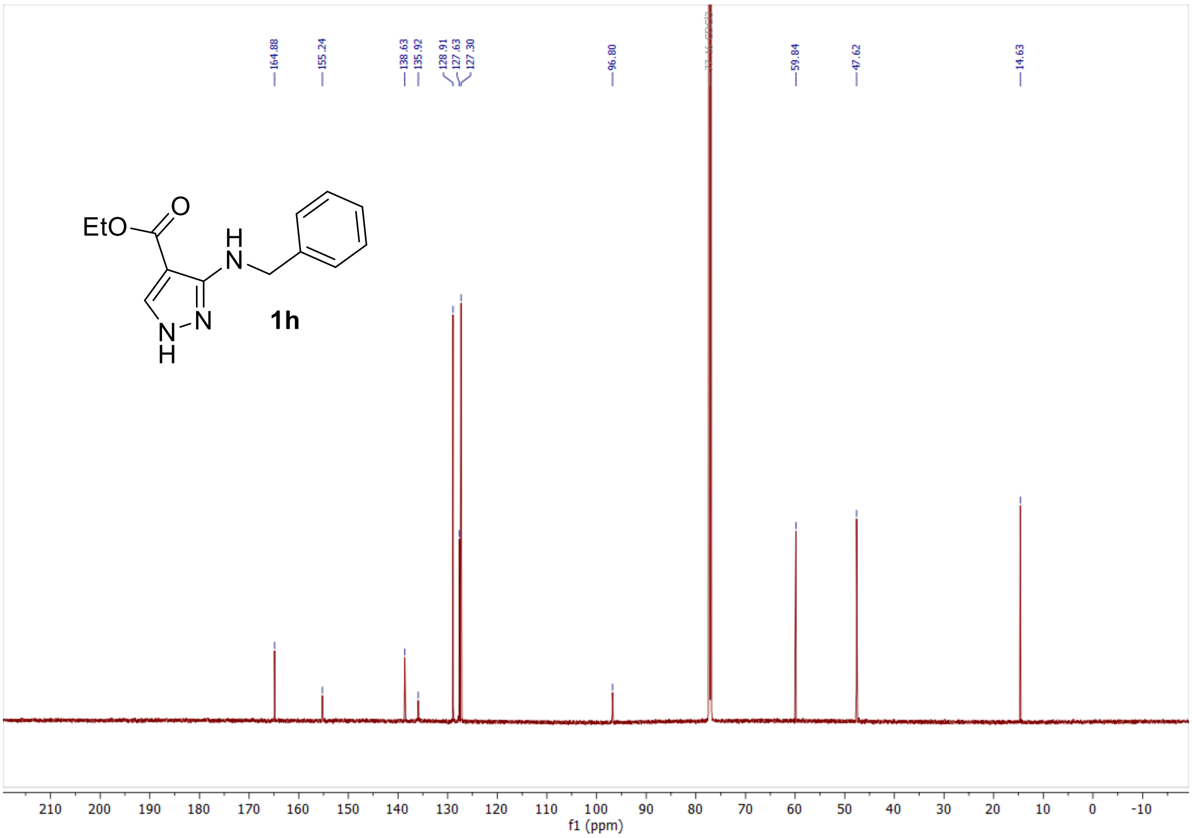

^{1}H NMR (400 MHz, $CDCl_3$) (2a)

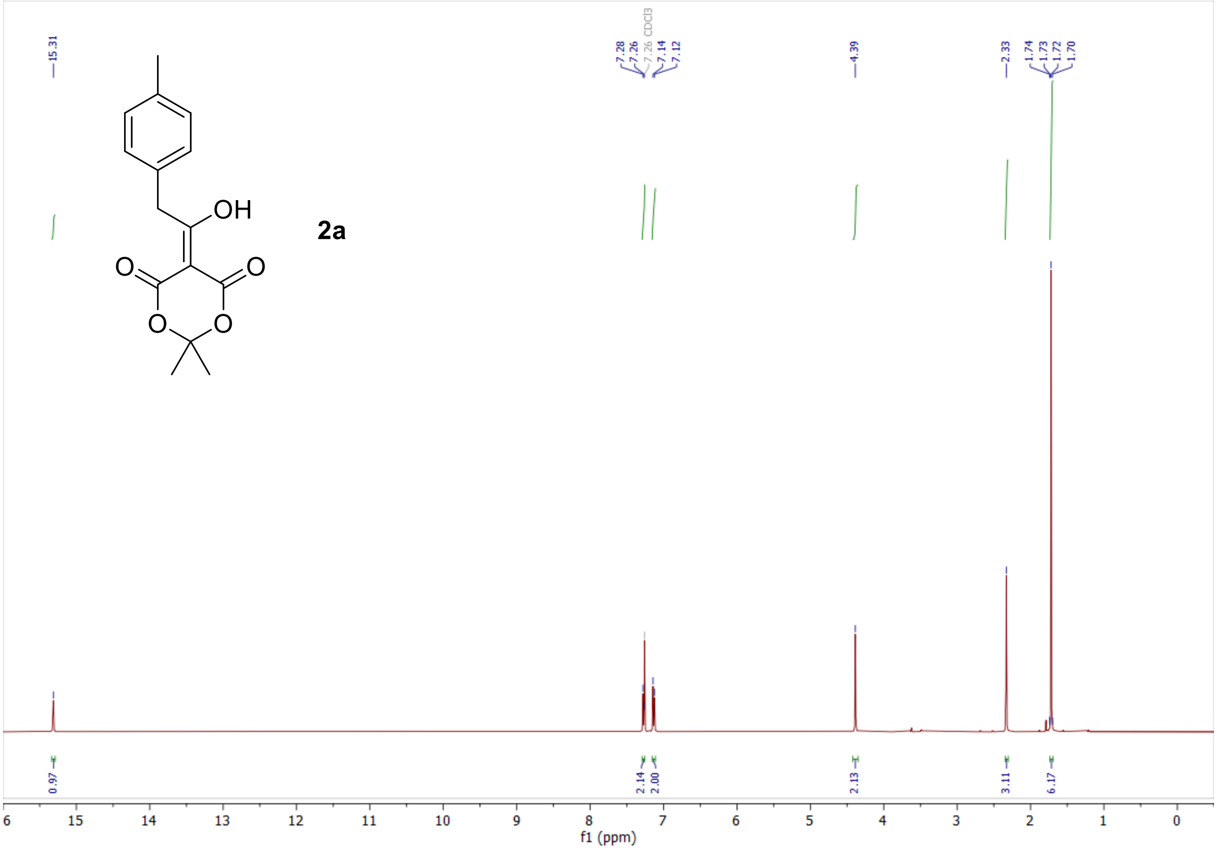

^{13}C NMR (100 MHz, $CDCl_3$) (2a)

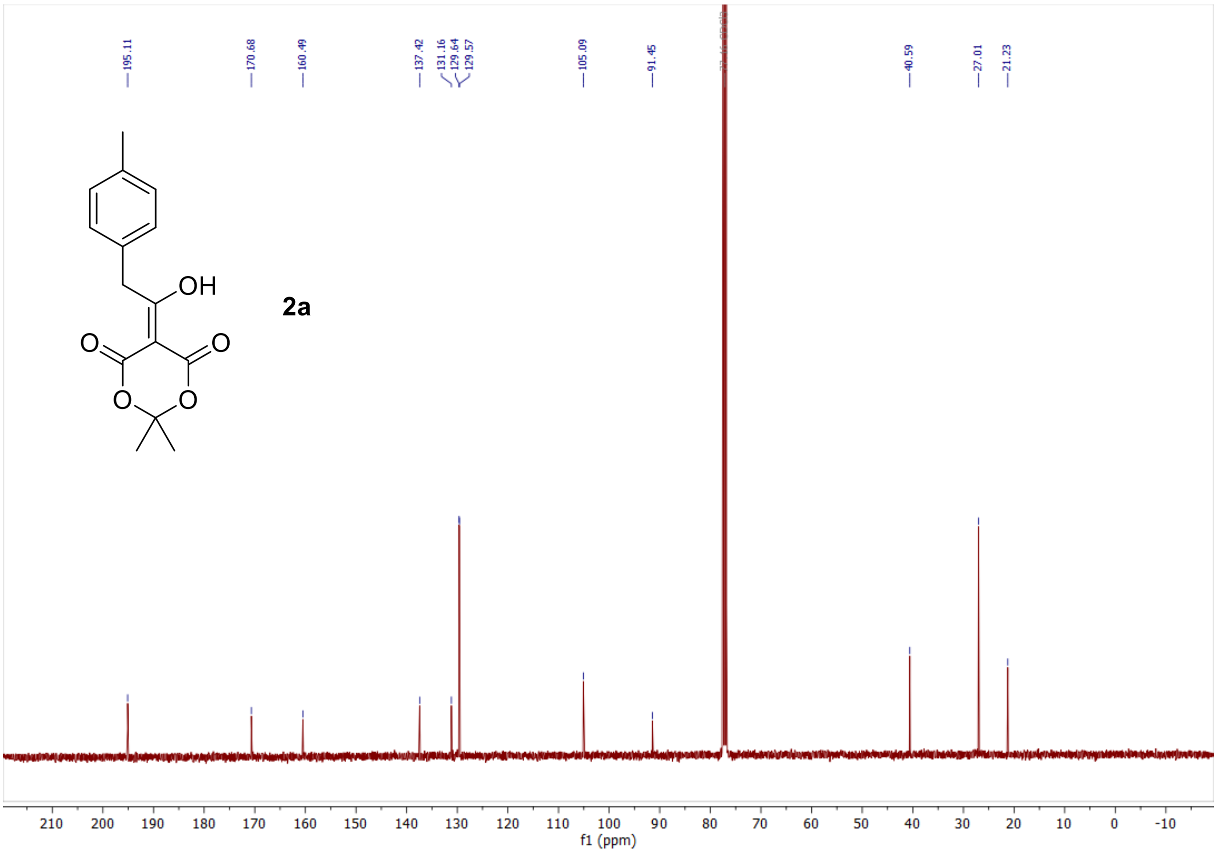

^{1}H NMR (400 MHz, $CDCl_3$) (2b)

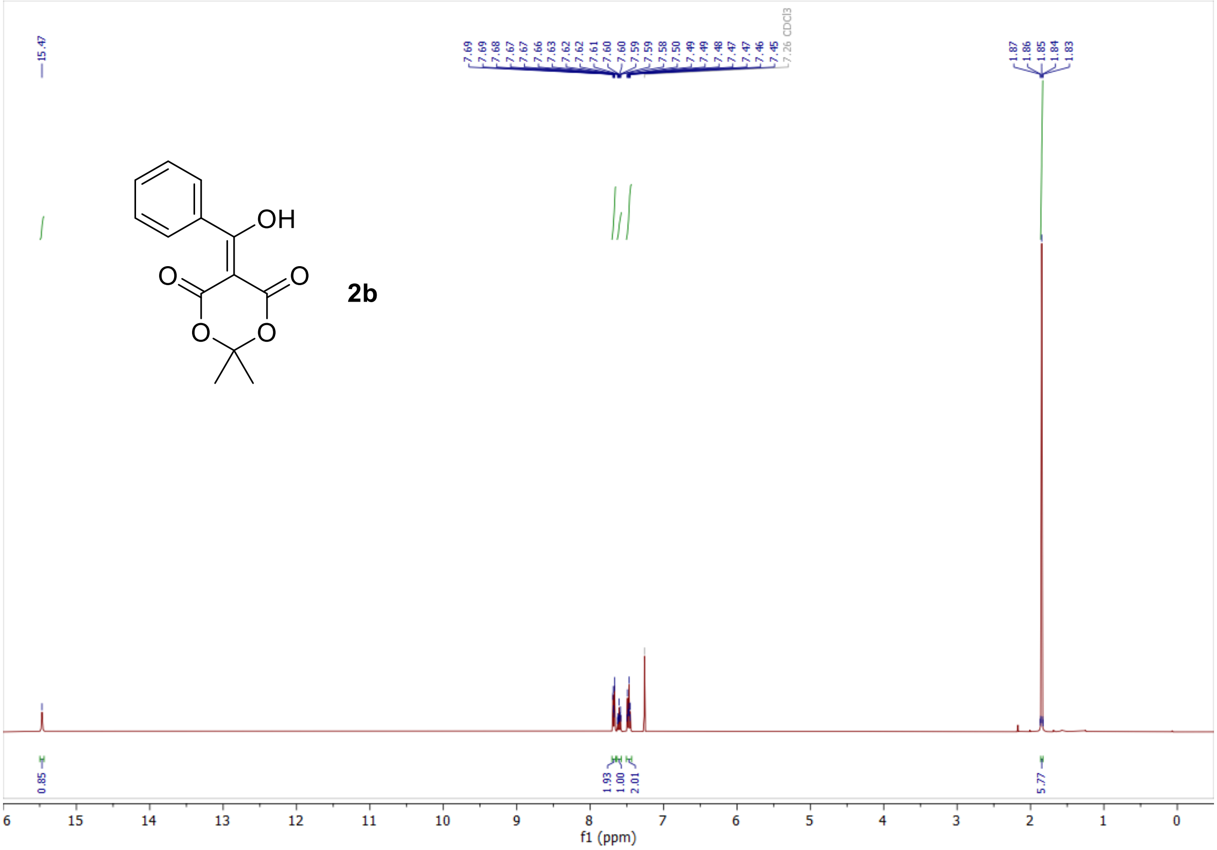

^{13}C NMR (100 MHz, $CDCl_3$) (2b)

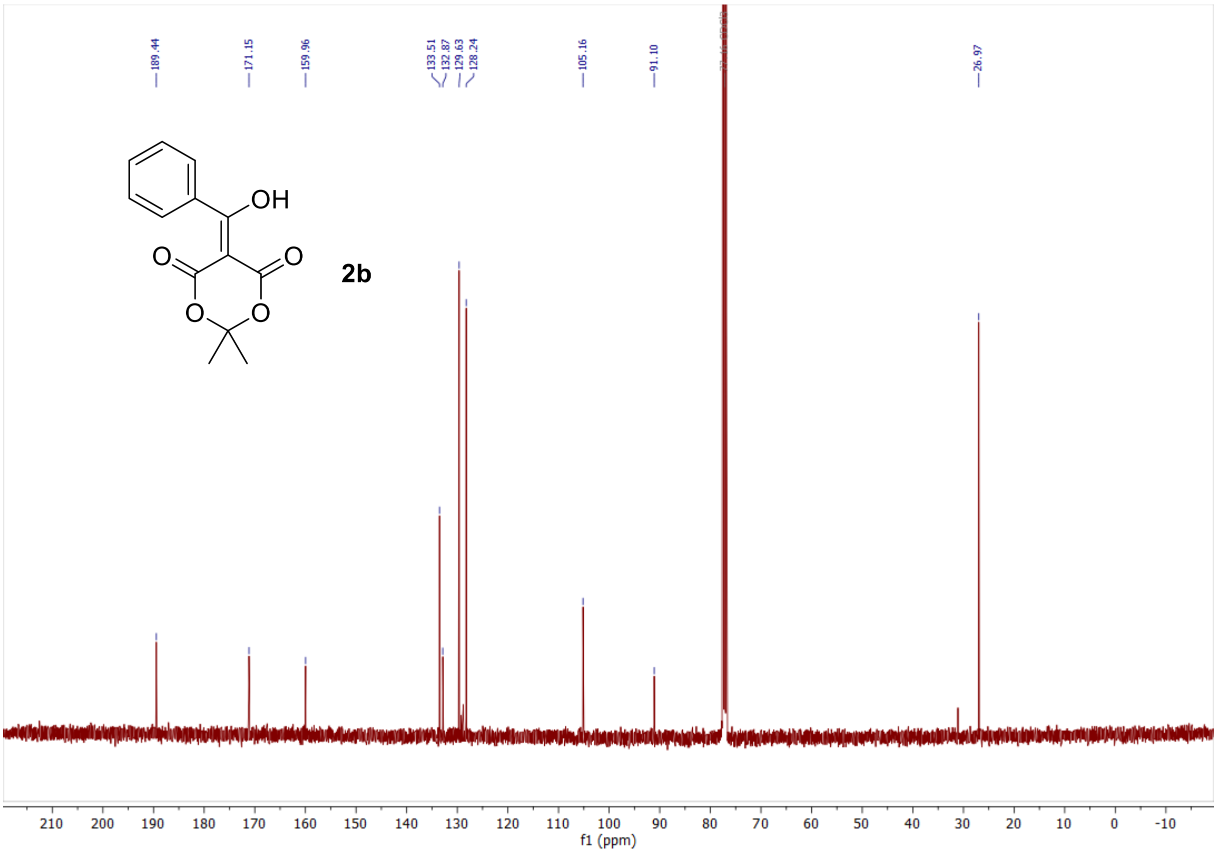

^{1}H NMR (400 MHz, $CDCl_3$) (2c)

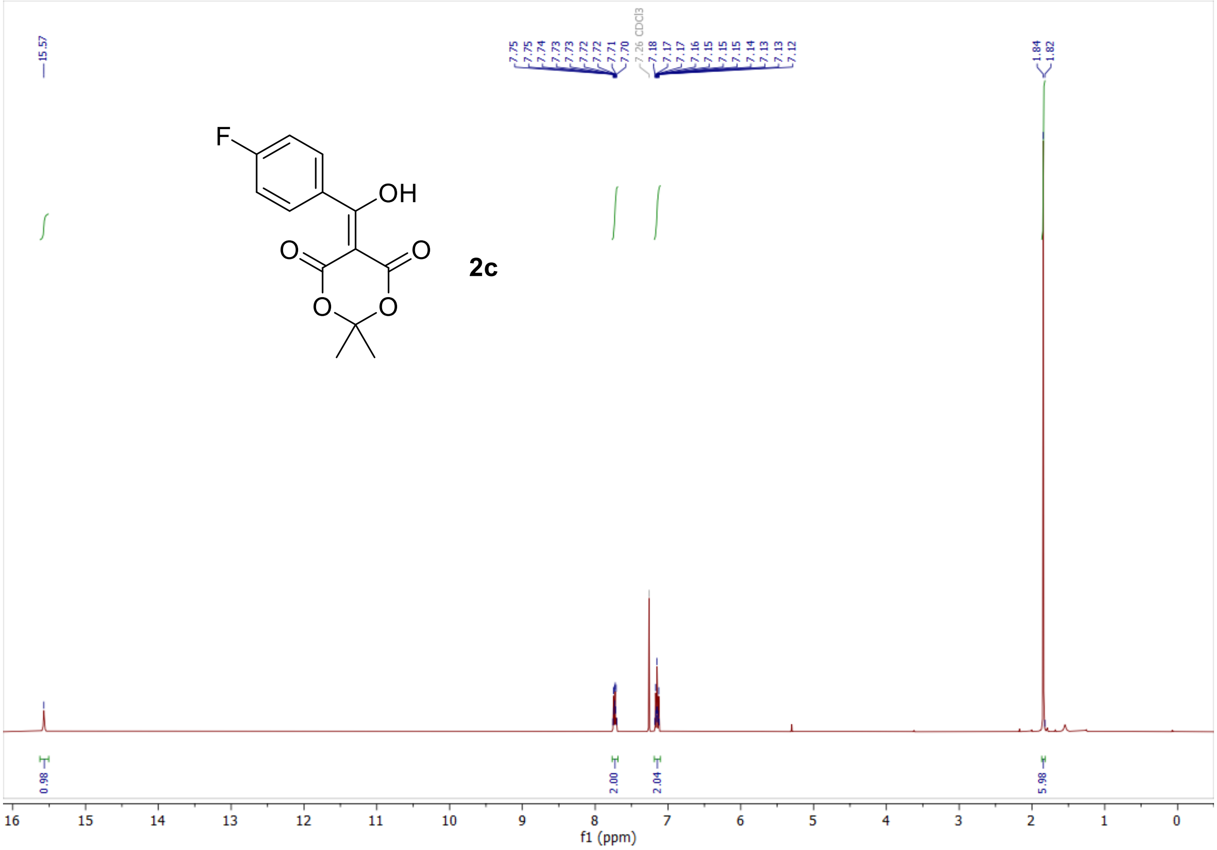

^{13}C NMR (100 MHz, $CDCl_3$) (2c)

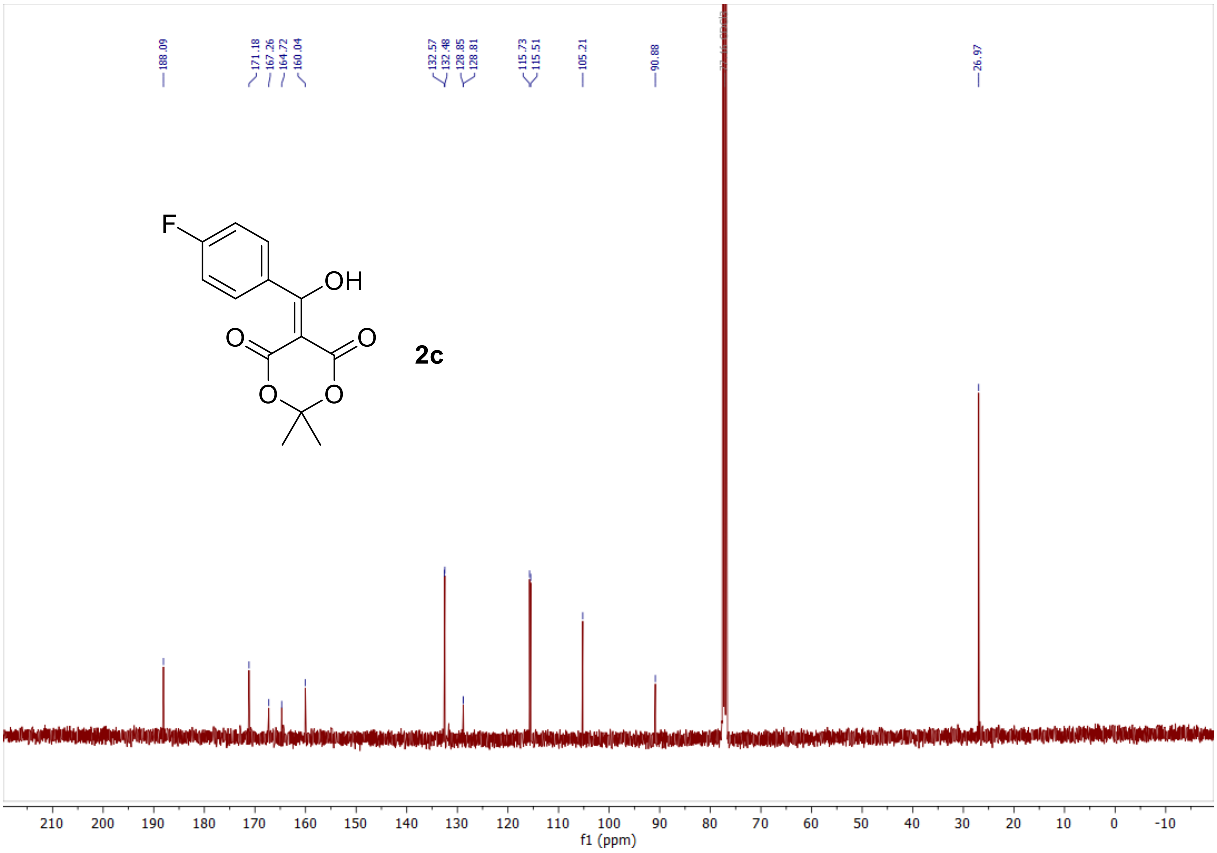

^{19}F NMR (376 MHz, $CDCl_3$) (2c)

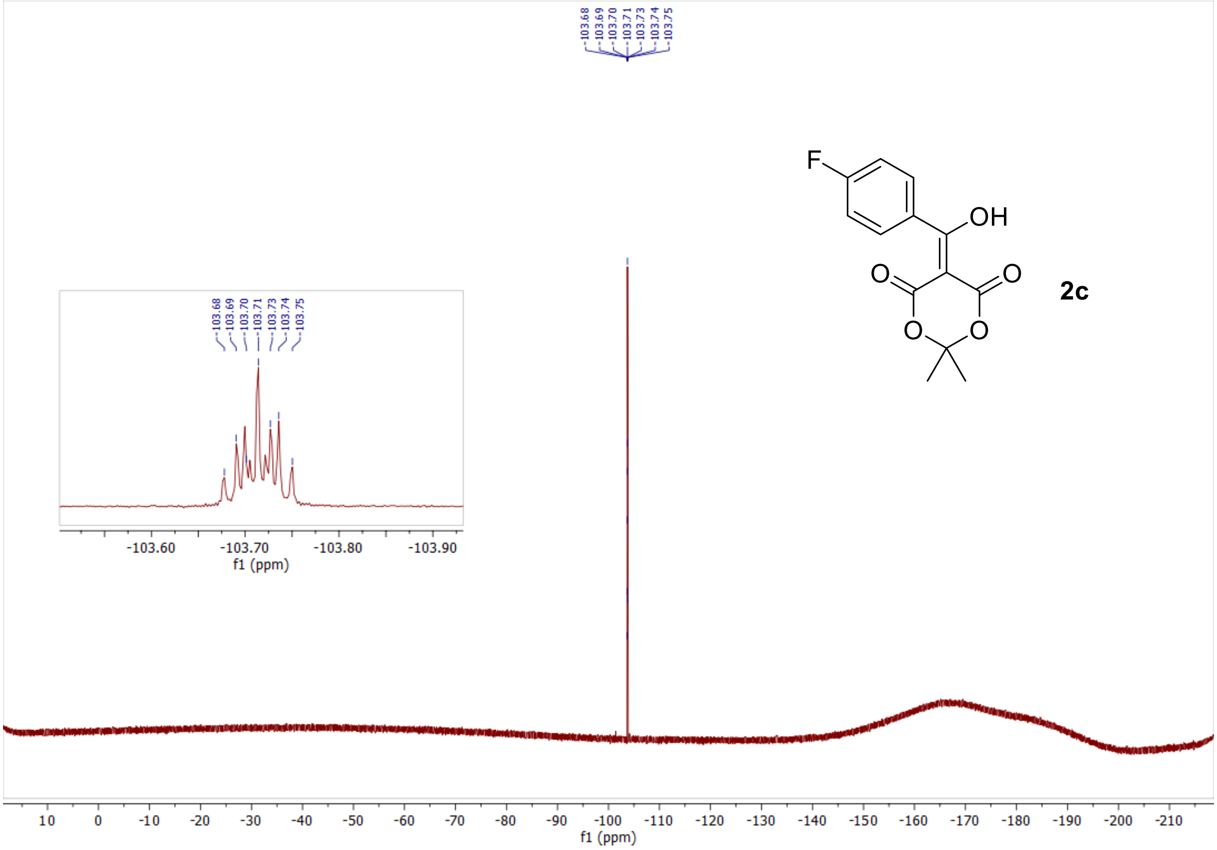

^{1}H NMR (600 MHz, $CDCl_3$) (2d)

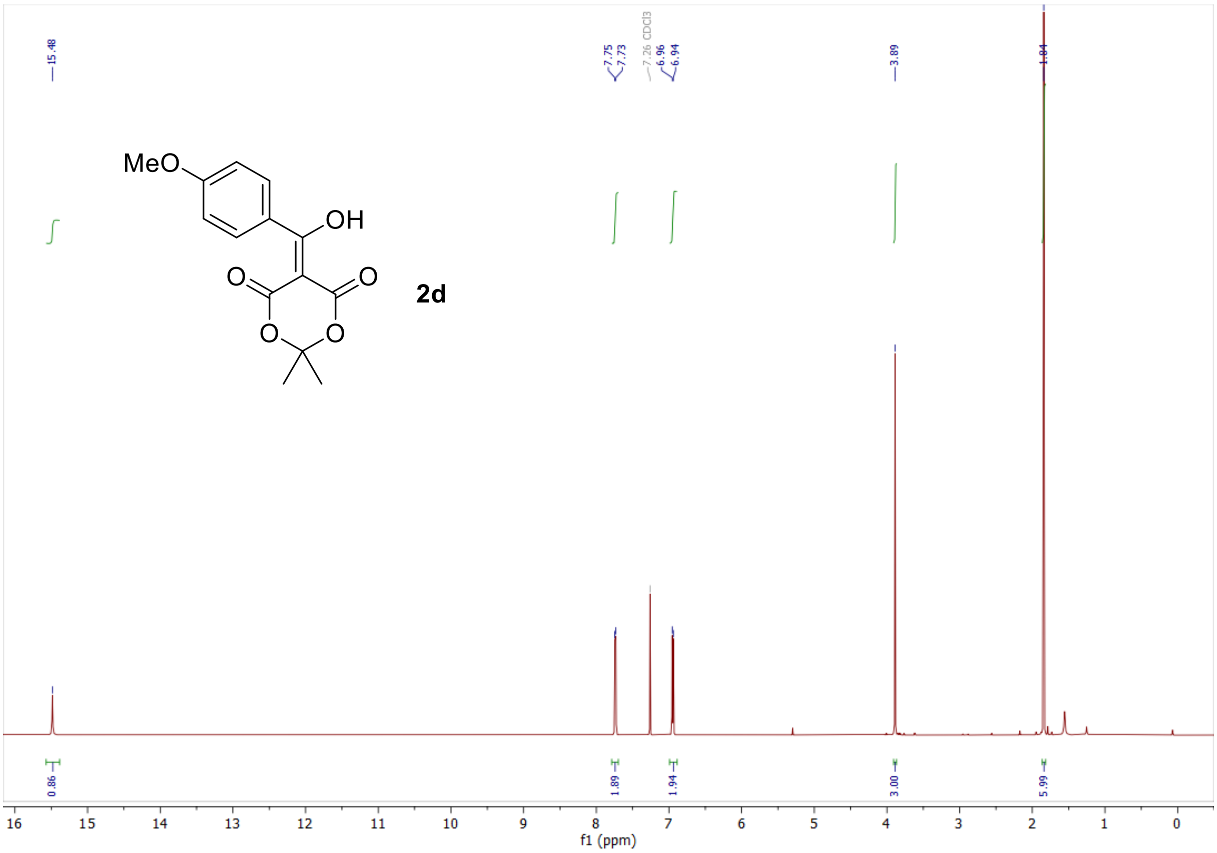

^{13}C NMR (151 MHz, $CDCl_3$) (2d)

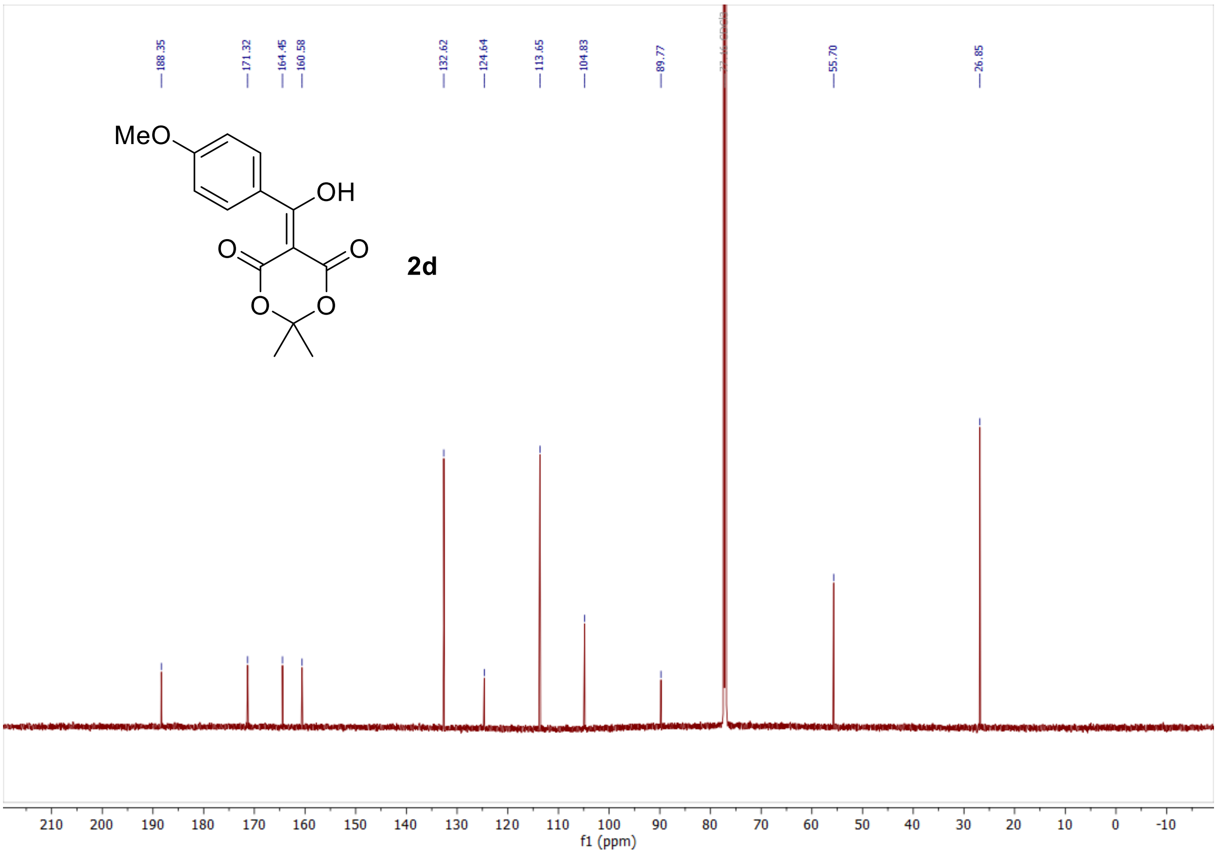

^{1}H NMR (600 MHz, $CDCl_3$) (2e)

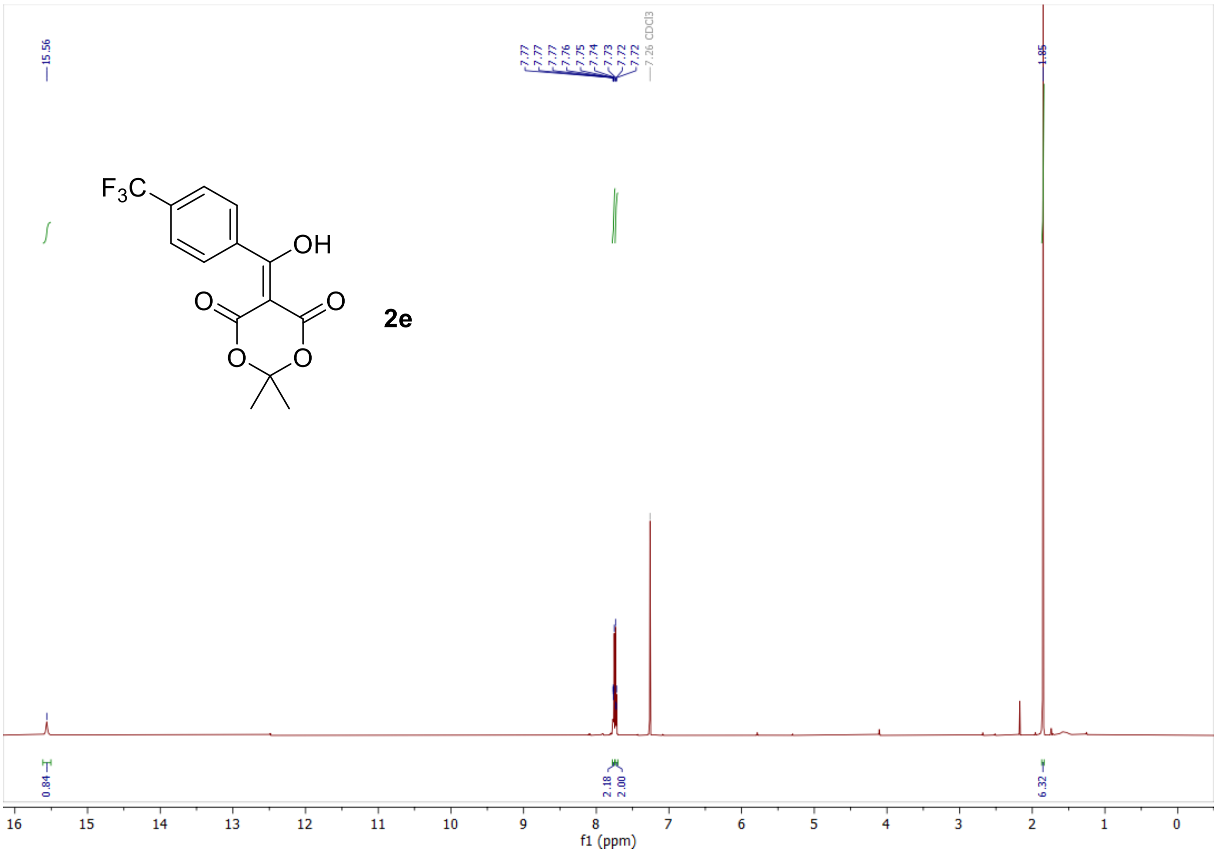

^{13}C NMR (151 MHz, $CDCl_3$) (2e)

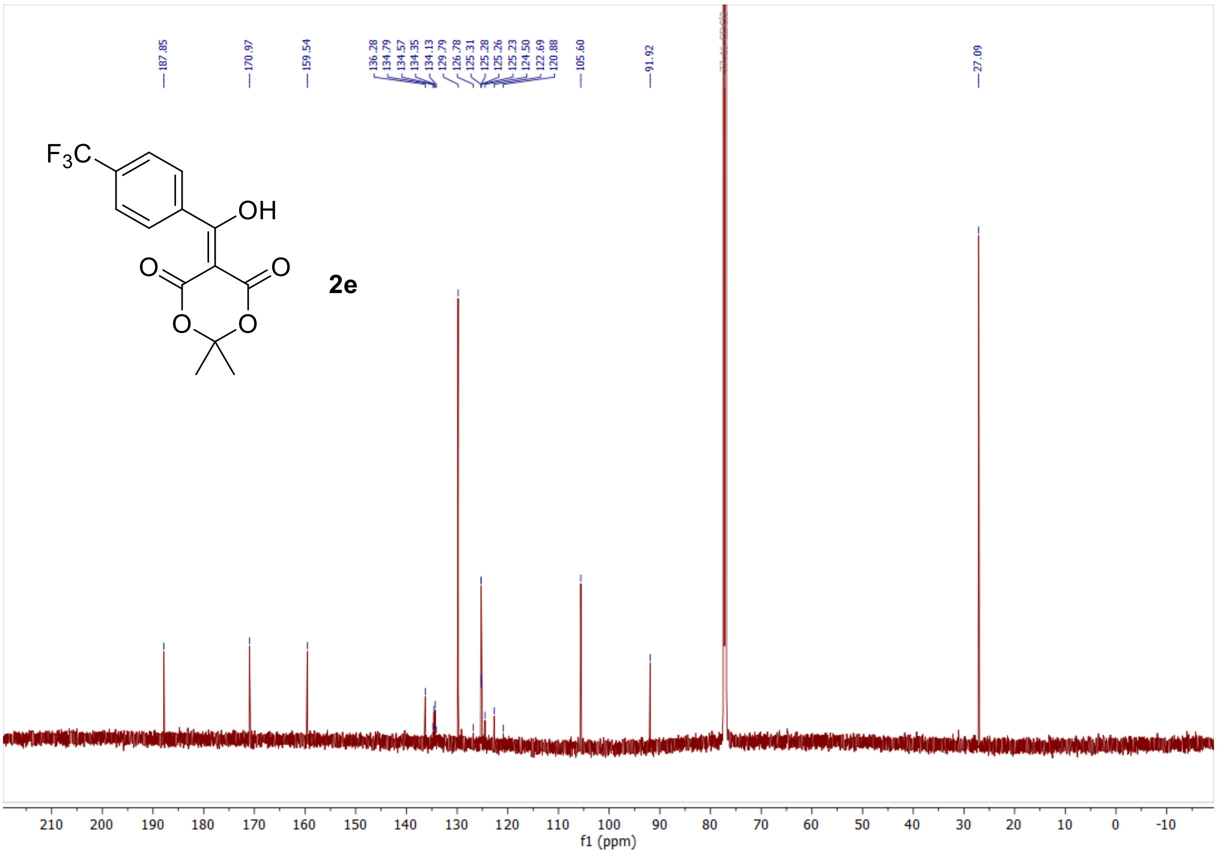

^{19}F NMR (565 MHz, $CDCl_3$) (2e)

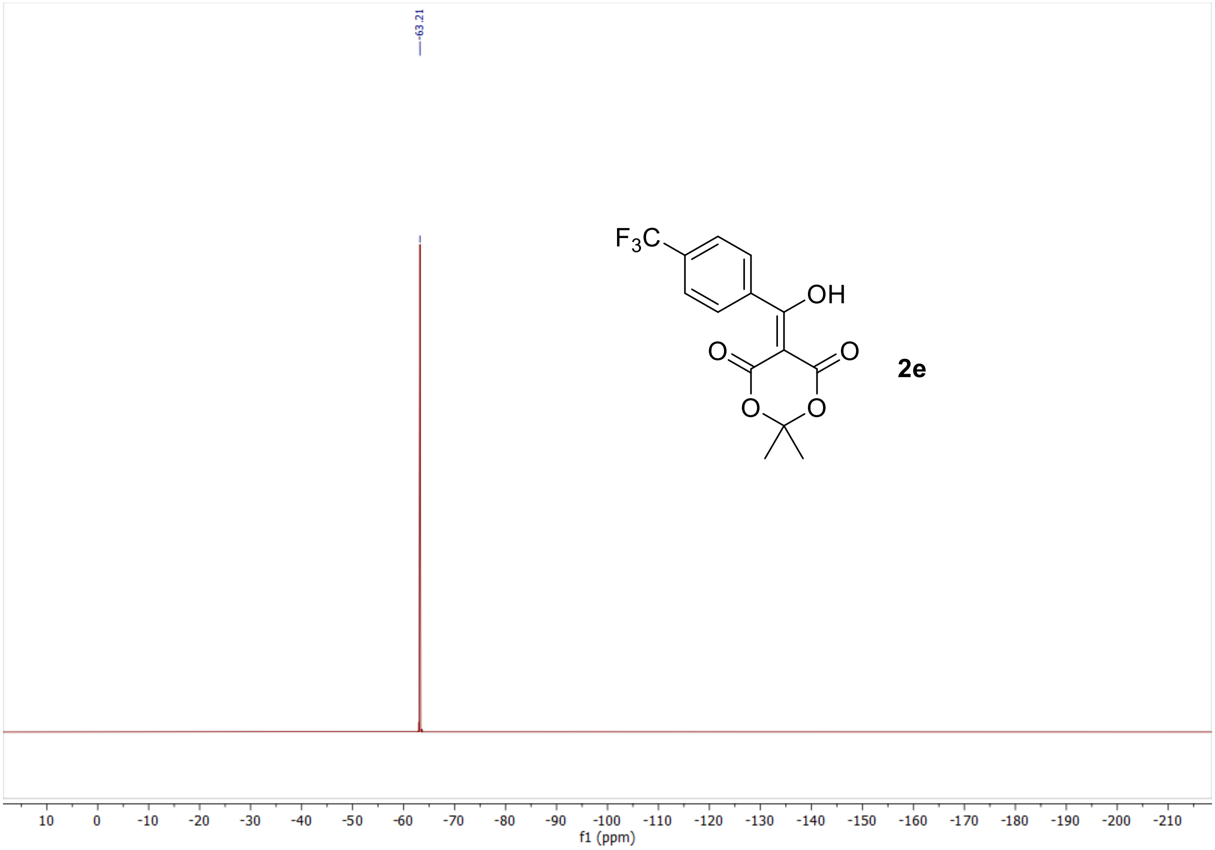

^{1}H NMR (600 MHz, $CDCl_3$) (2f)

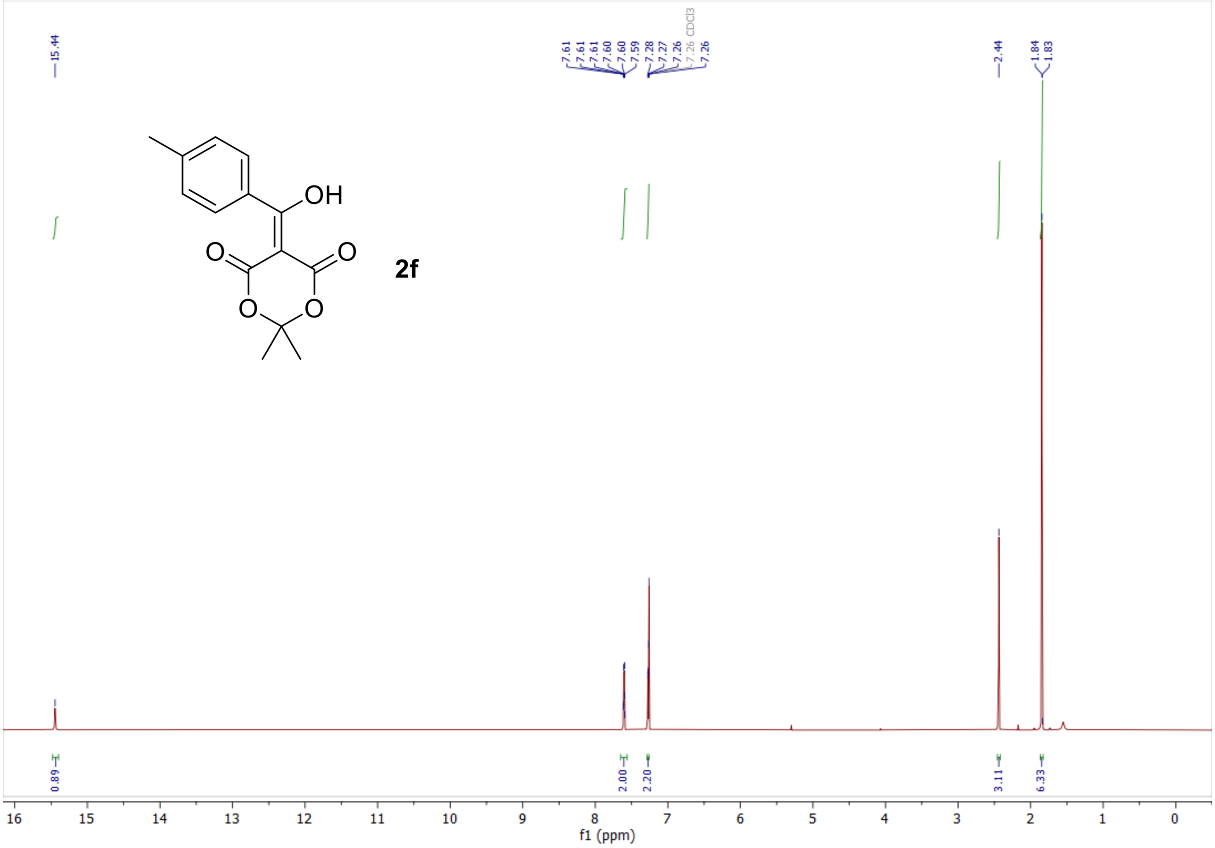

^{13}C NMR (151 MHz, $CDCl_3$) (2f)

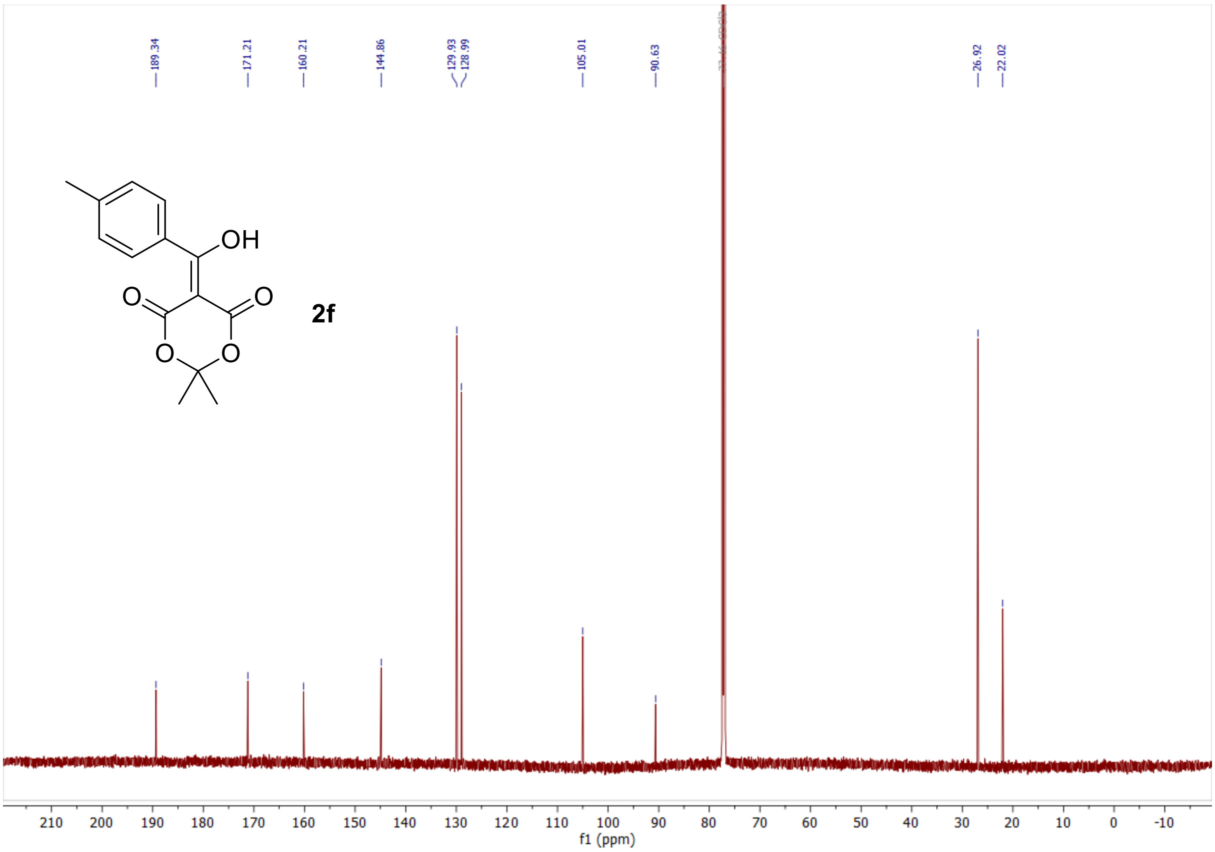

^{1}H NMR (600 MHz, $CDCl_3$) (2g)

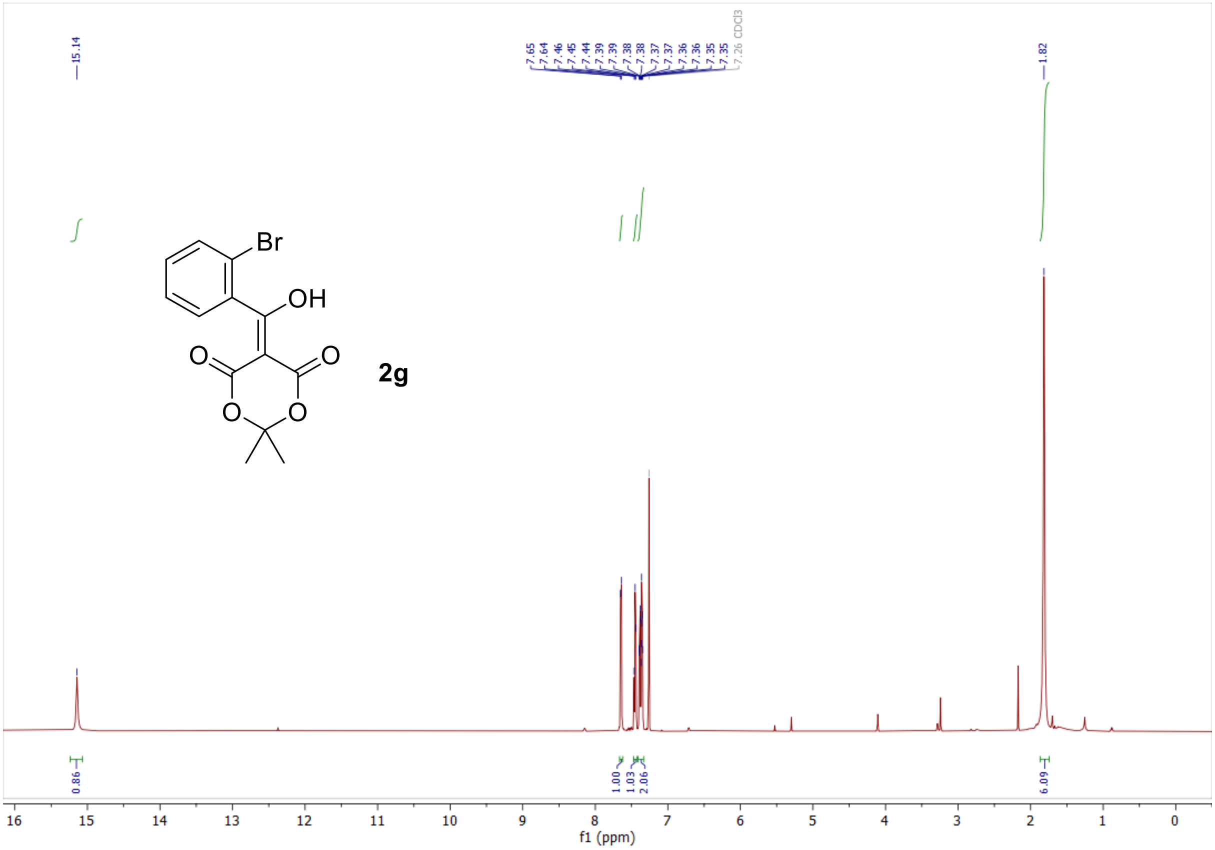

^{13}C NMR (151 MHz, $CDCl_3$) (2g)

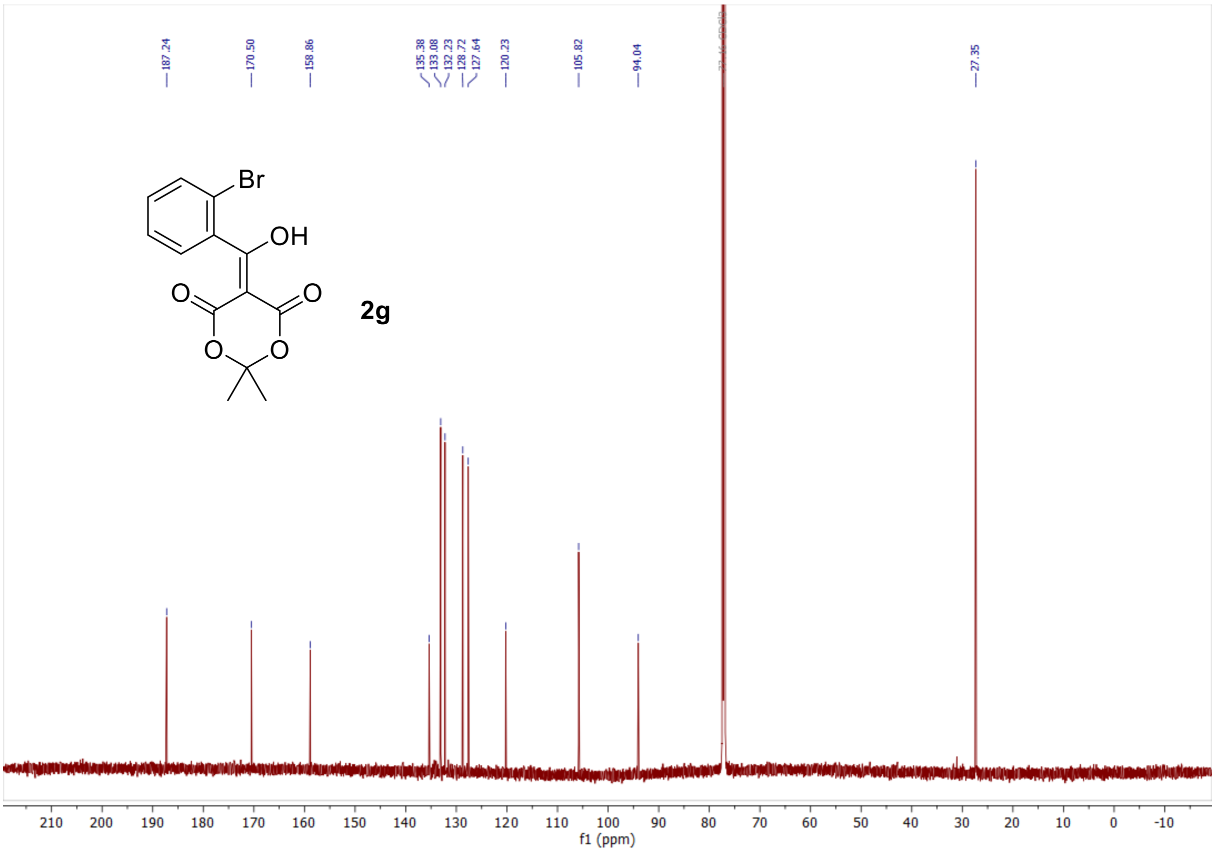

^{1}H NMR (400 MHz, $CDCl_3$) (2h)

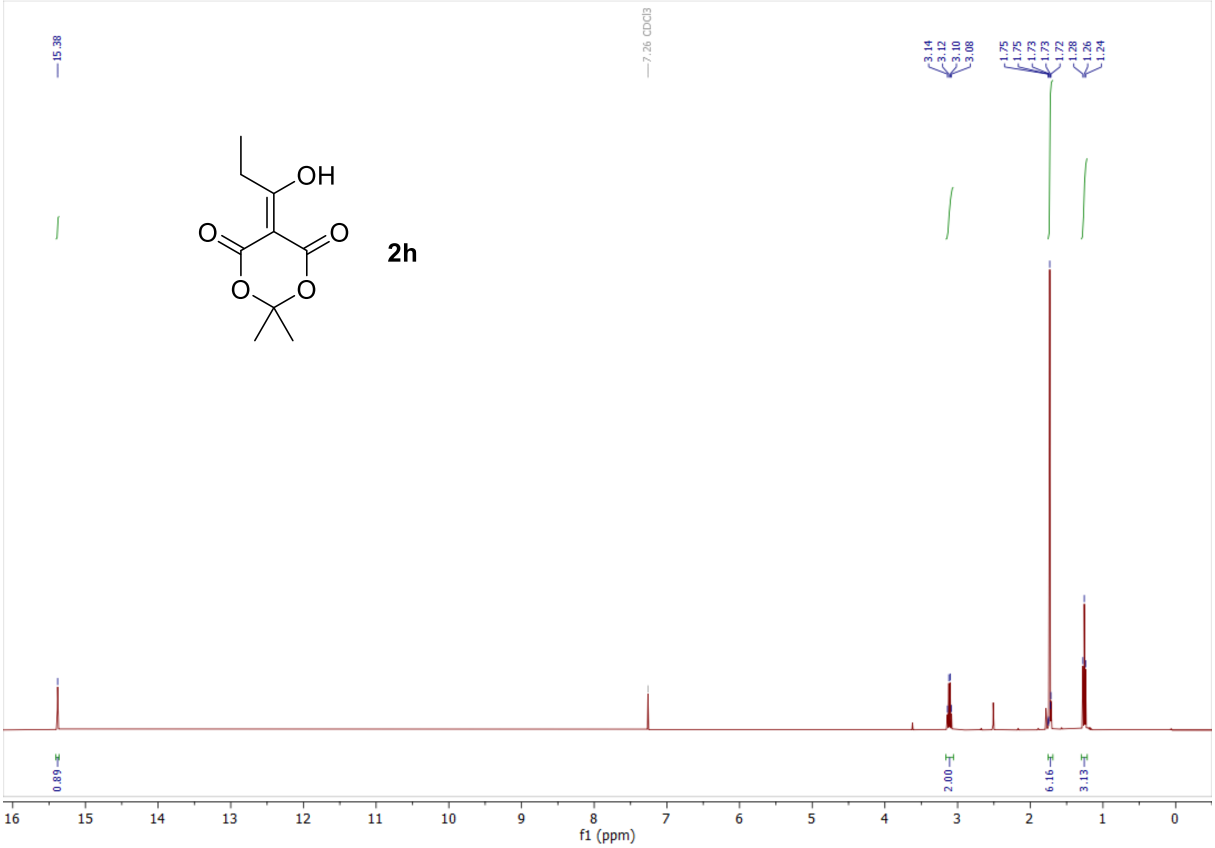

^{13}C NMR (100 MHz, $CDCl_3$) (2h)

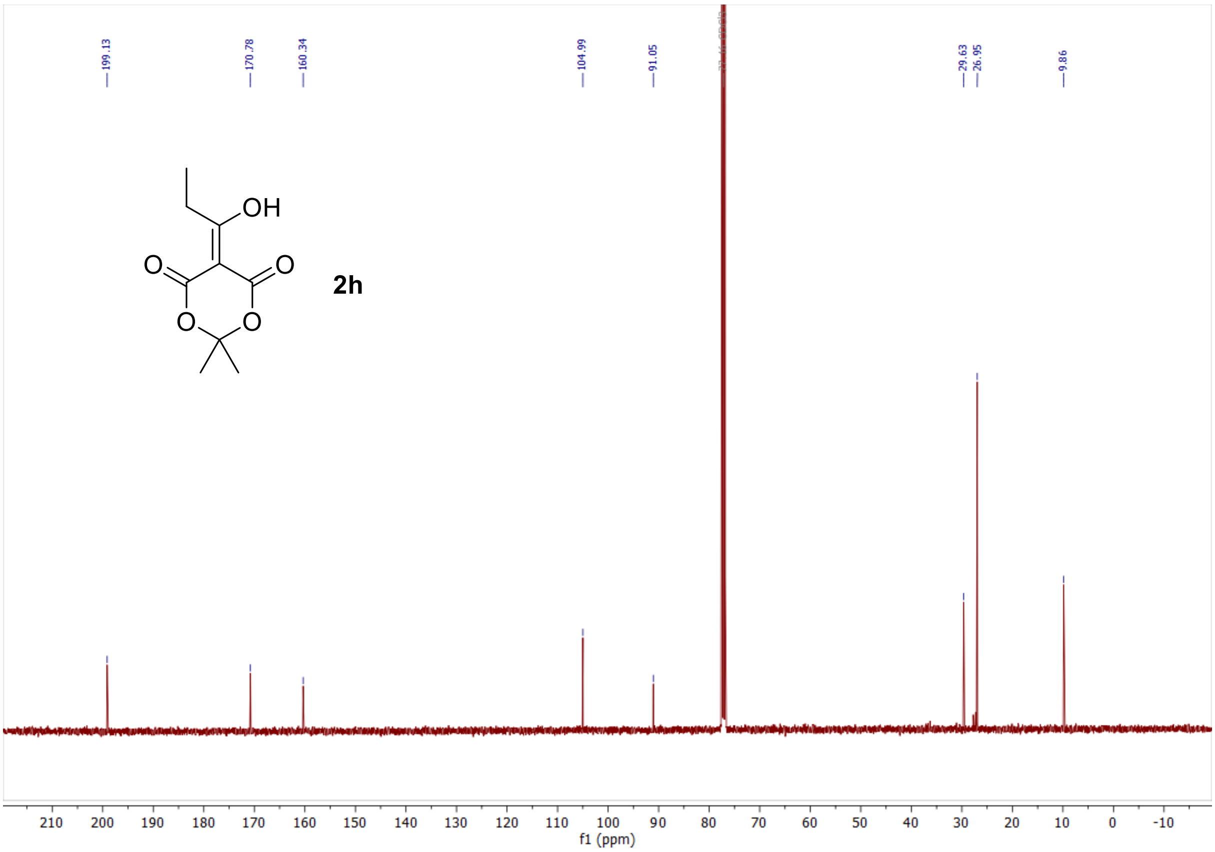

^{1}H NMR (400 MHz, $CDCl_3$) (2i)

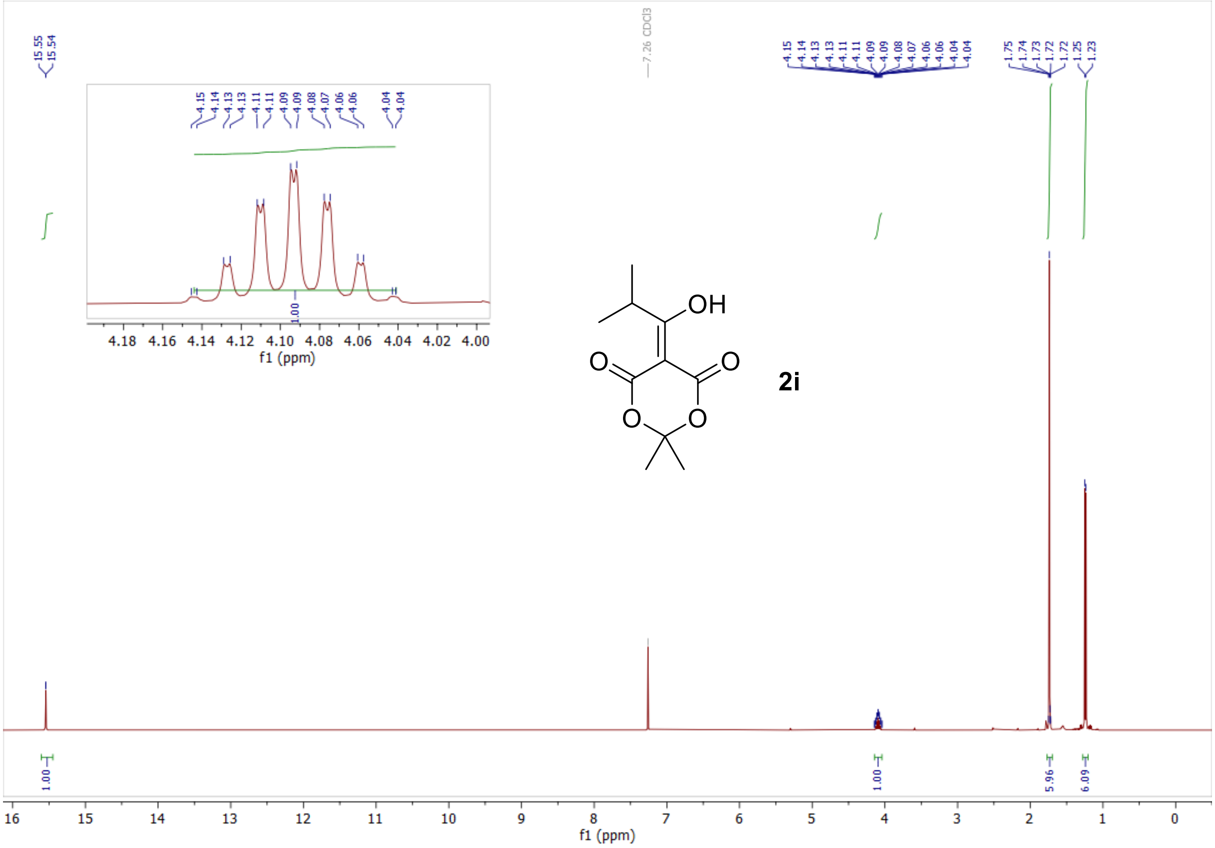

^{13}C NMR (100 MHz, $CDCl_3$) (2i)

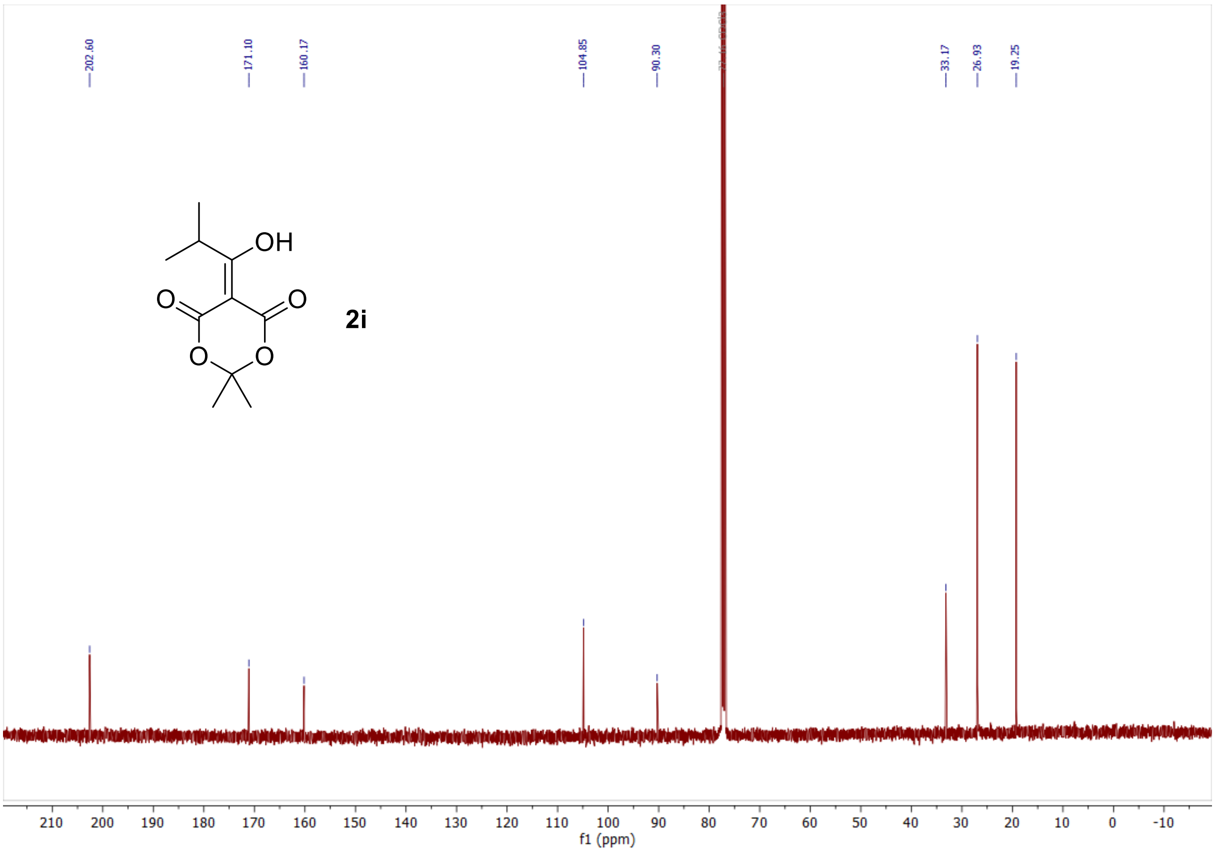

^{1}H NMR (600 MHz, $CDCl_3$) (2j)

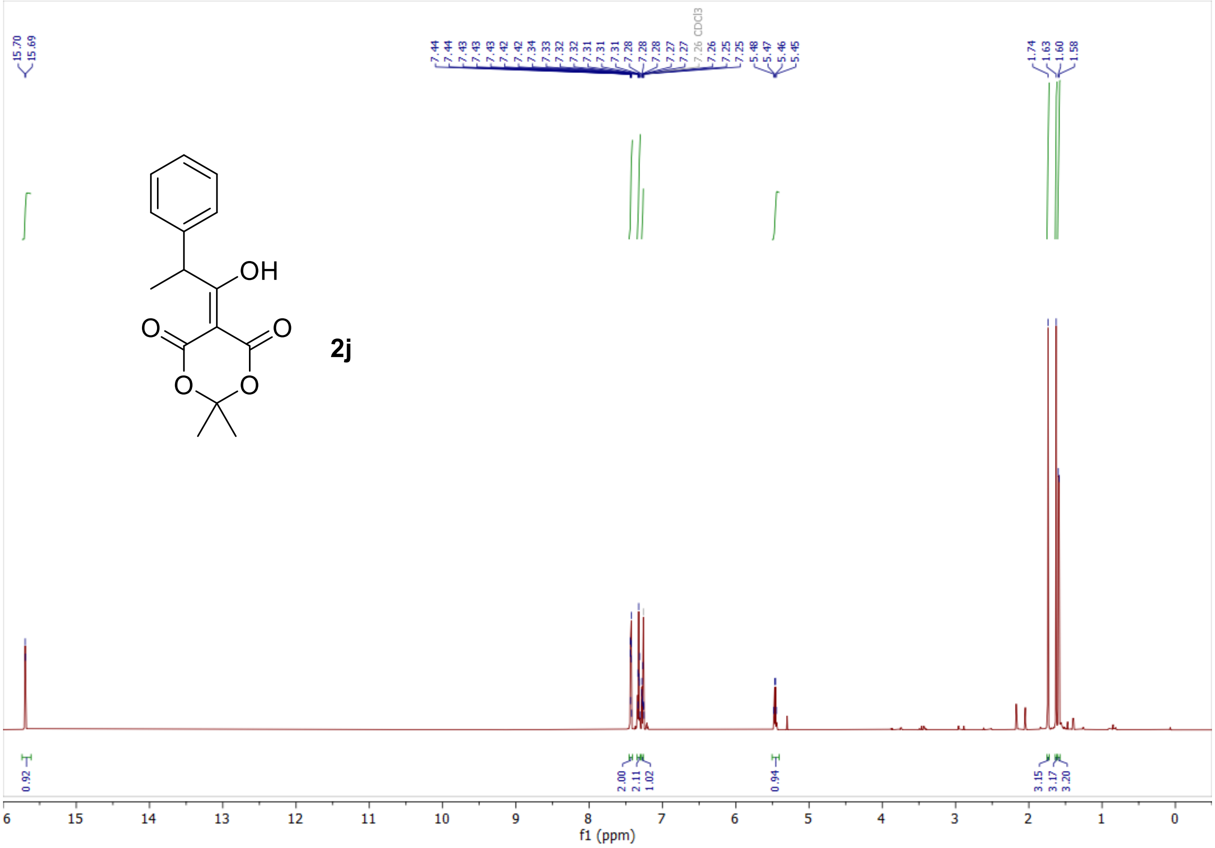

^{13}C NMR (151 MHz, $CDCl_3$) (2j)

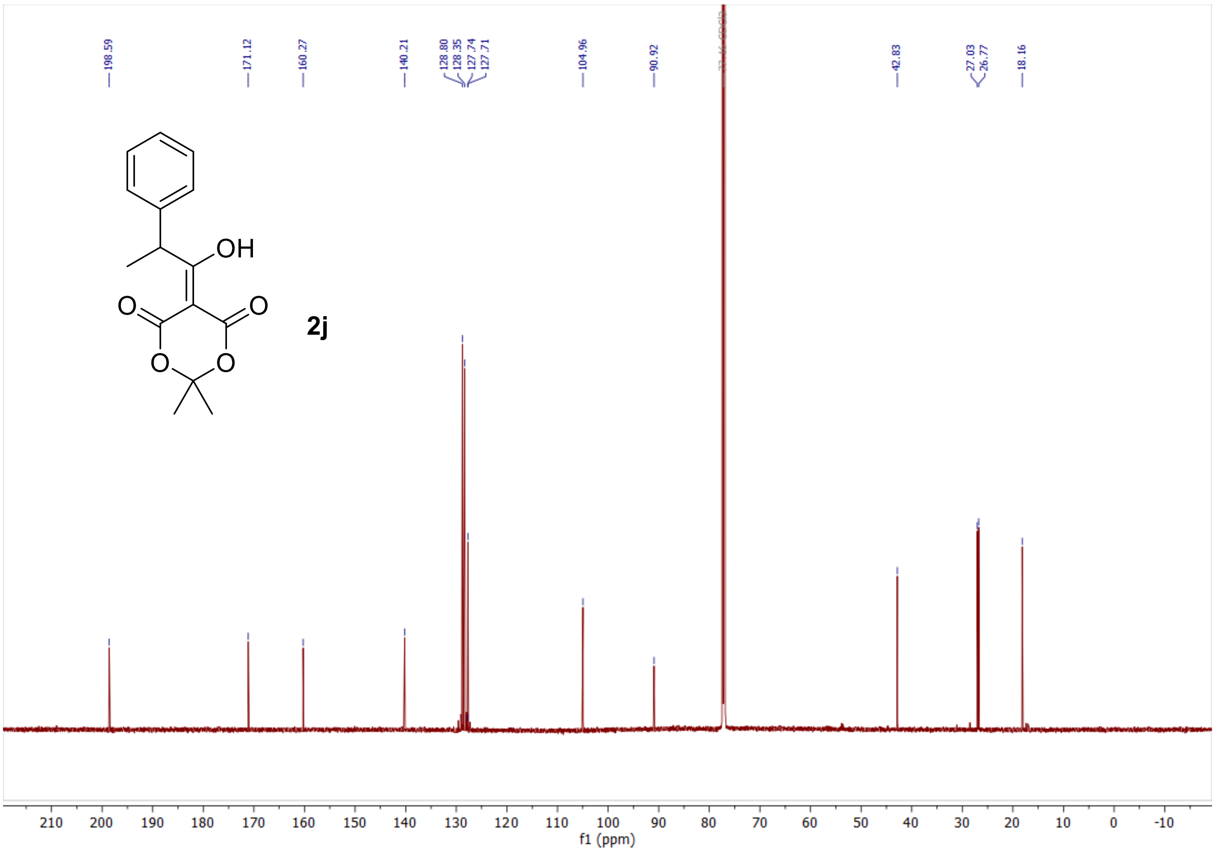

^{1}H NMR (600 MHz, $CDCl_3$) (2l)

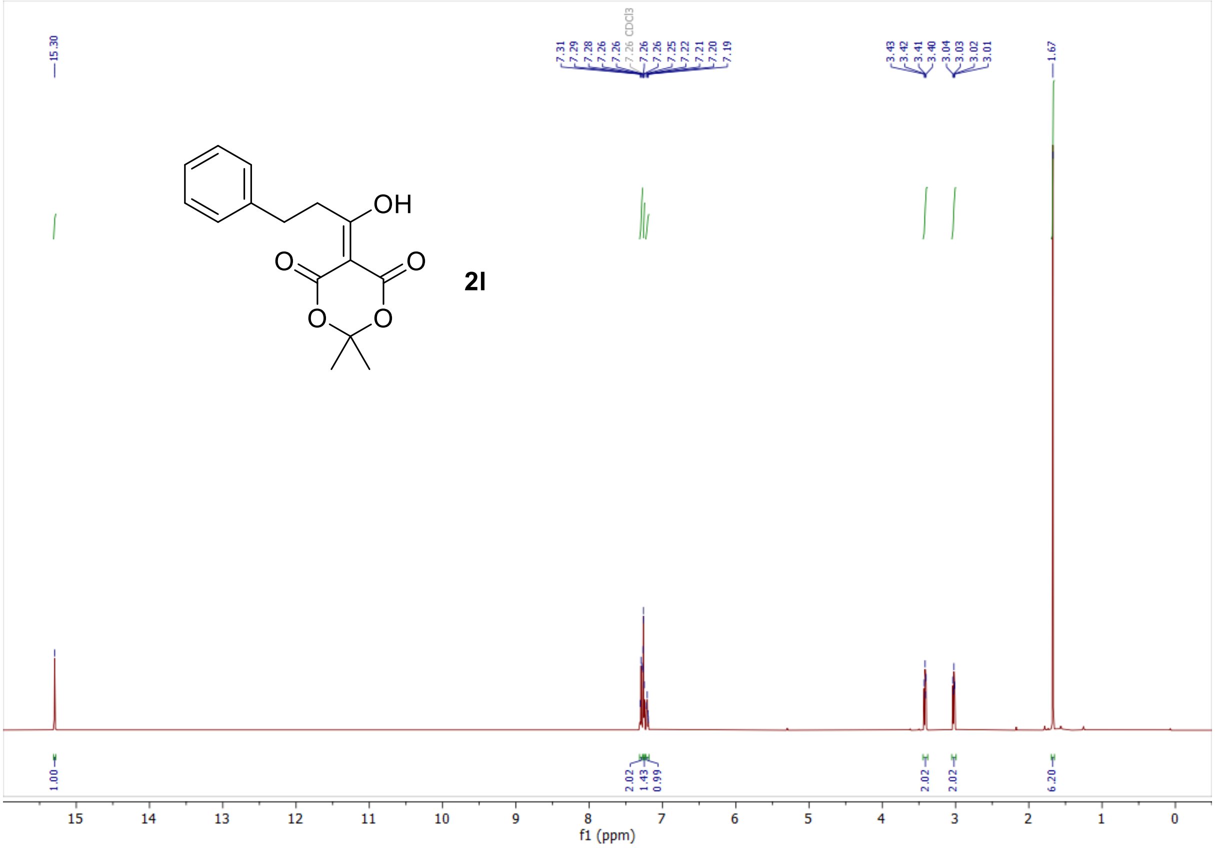

^{13}C NMR (151 MHz, $CDCl_3$) (2l)

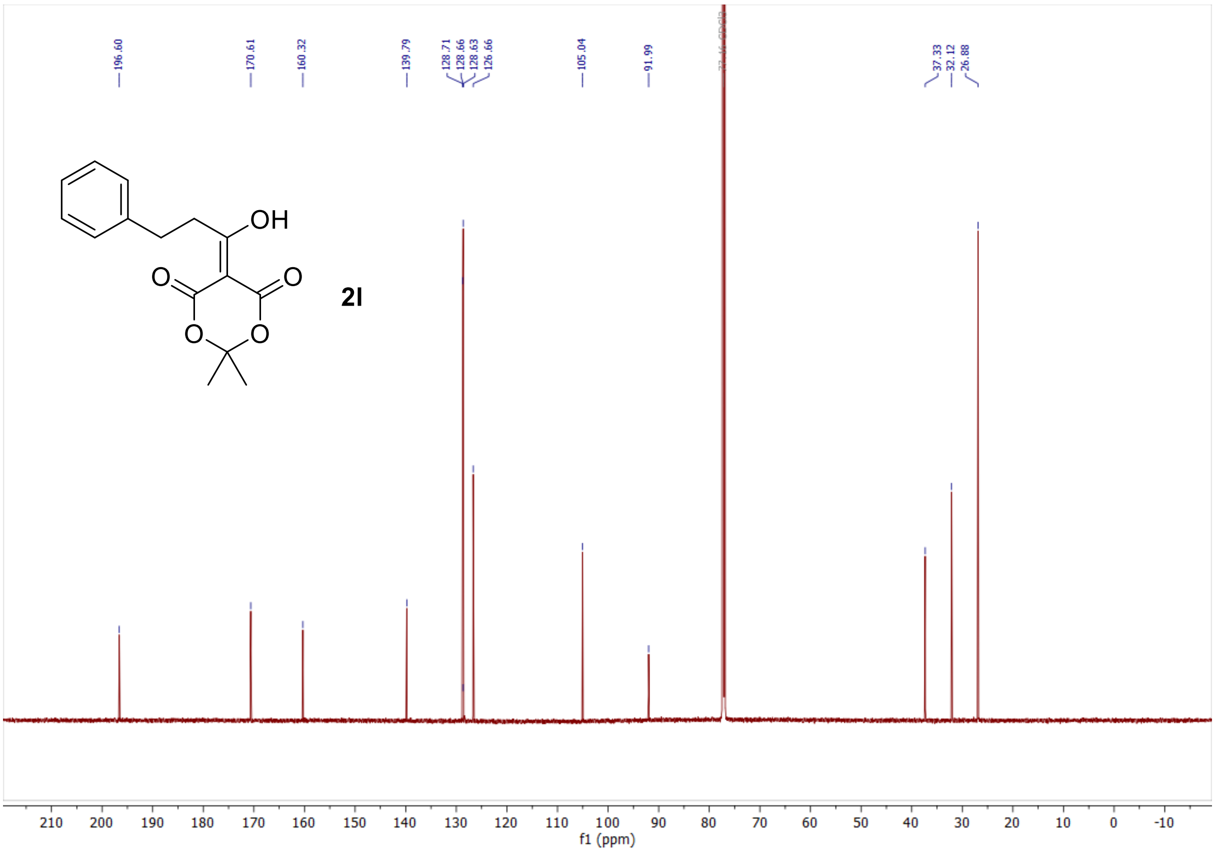

^1H NMR (600 MHz, $CDCl_3$) (2m)

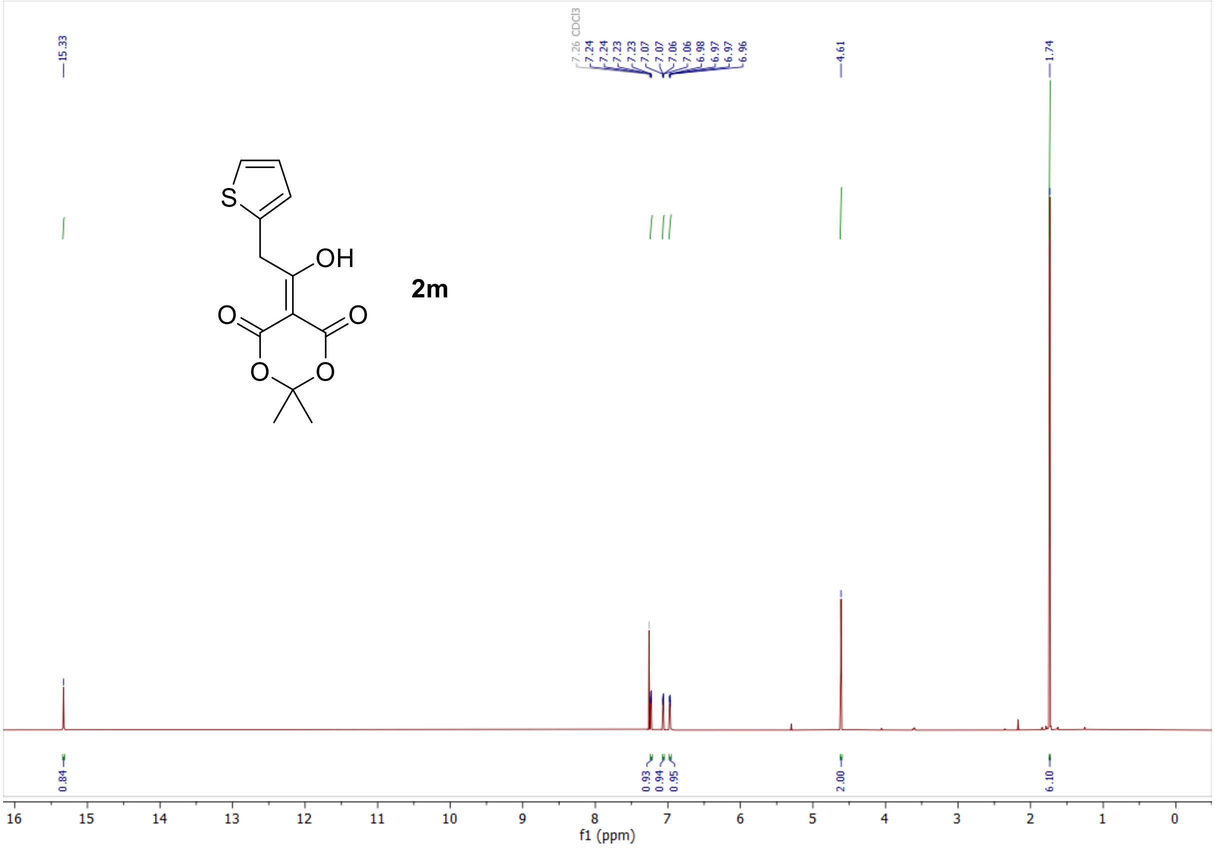

^{13}C NMR (151 MHz, $CDCl_3$) (2m)

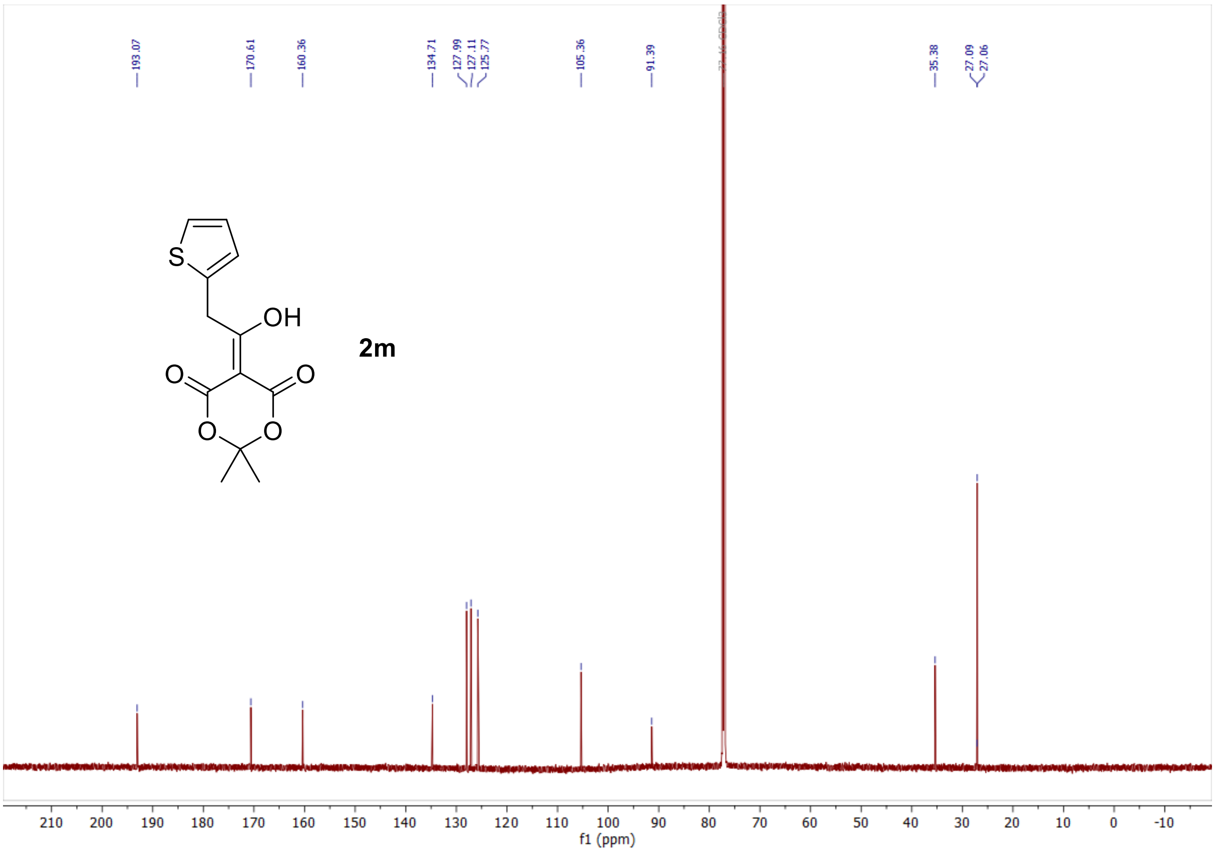

^{1}H NMR (600 MHz, $CDCl_3$) (2n)

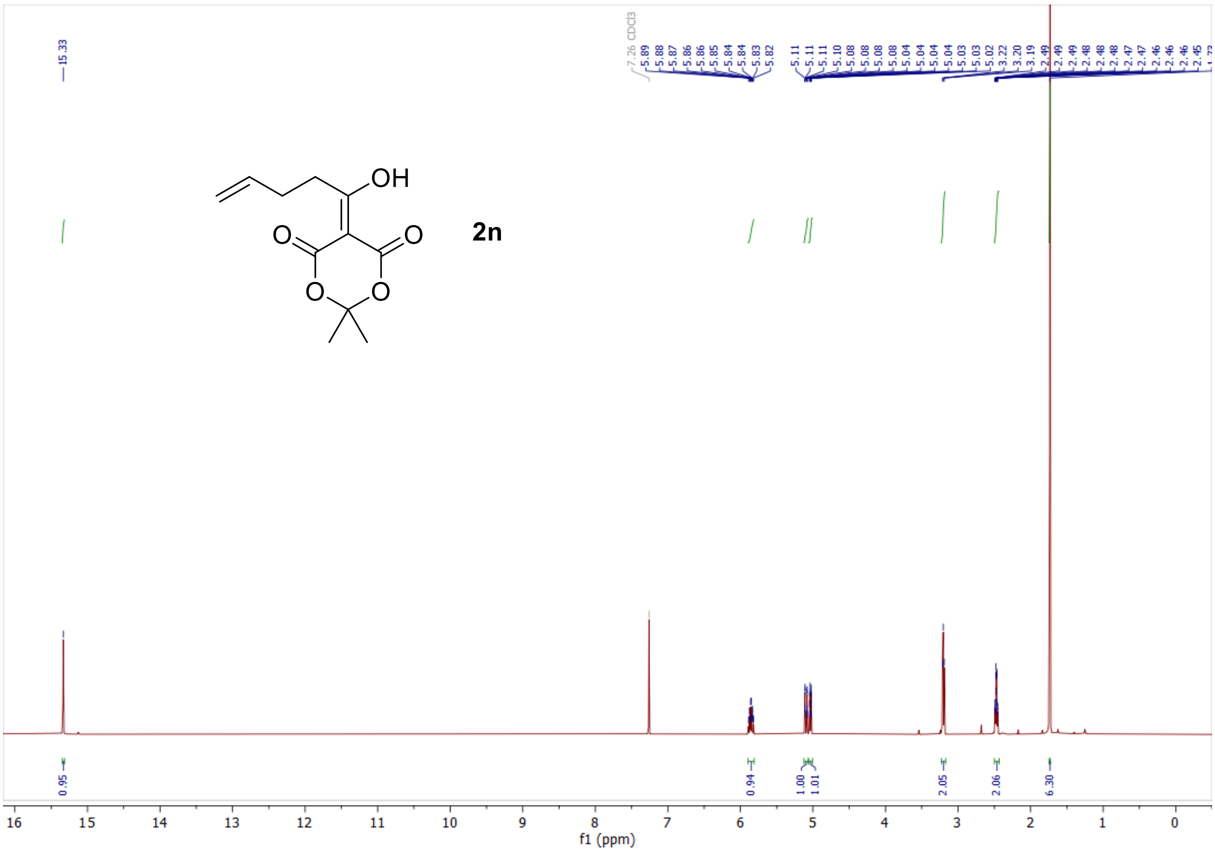

^{13}C NMR (151 MHz, $CDCl_3$) (2n)

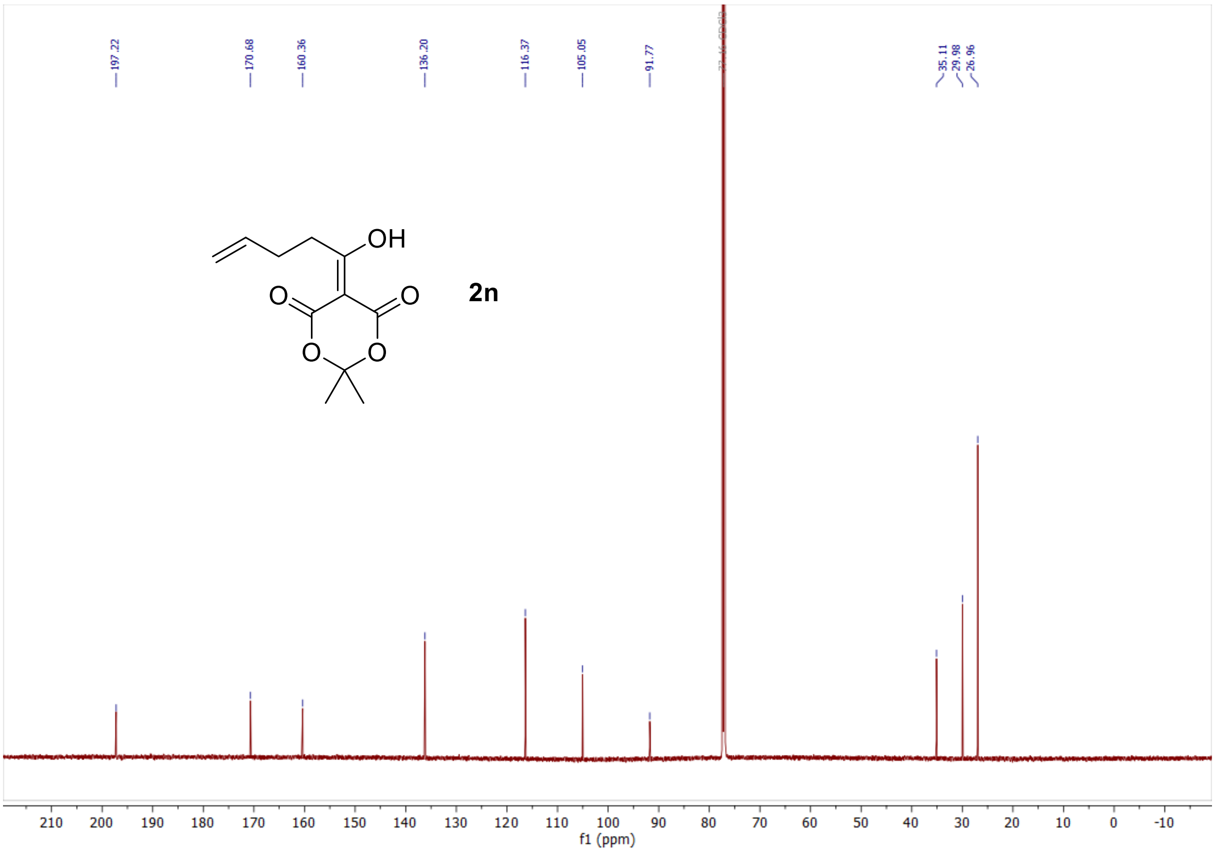

^{1}H NMR (600 MHz, CDCl$_3$) (2o)

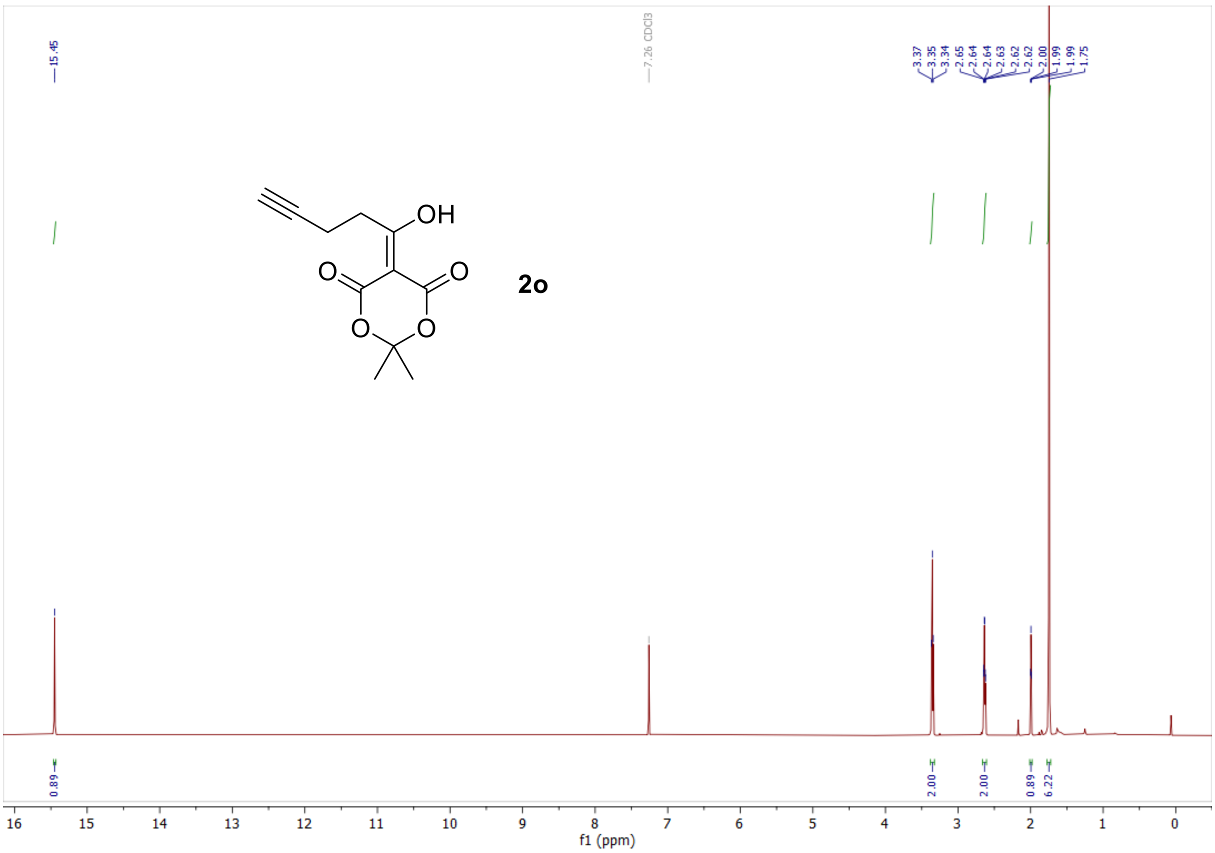

^{13}C NMR (151 MHz, CDCl$_3$) (2o)

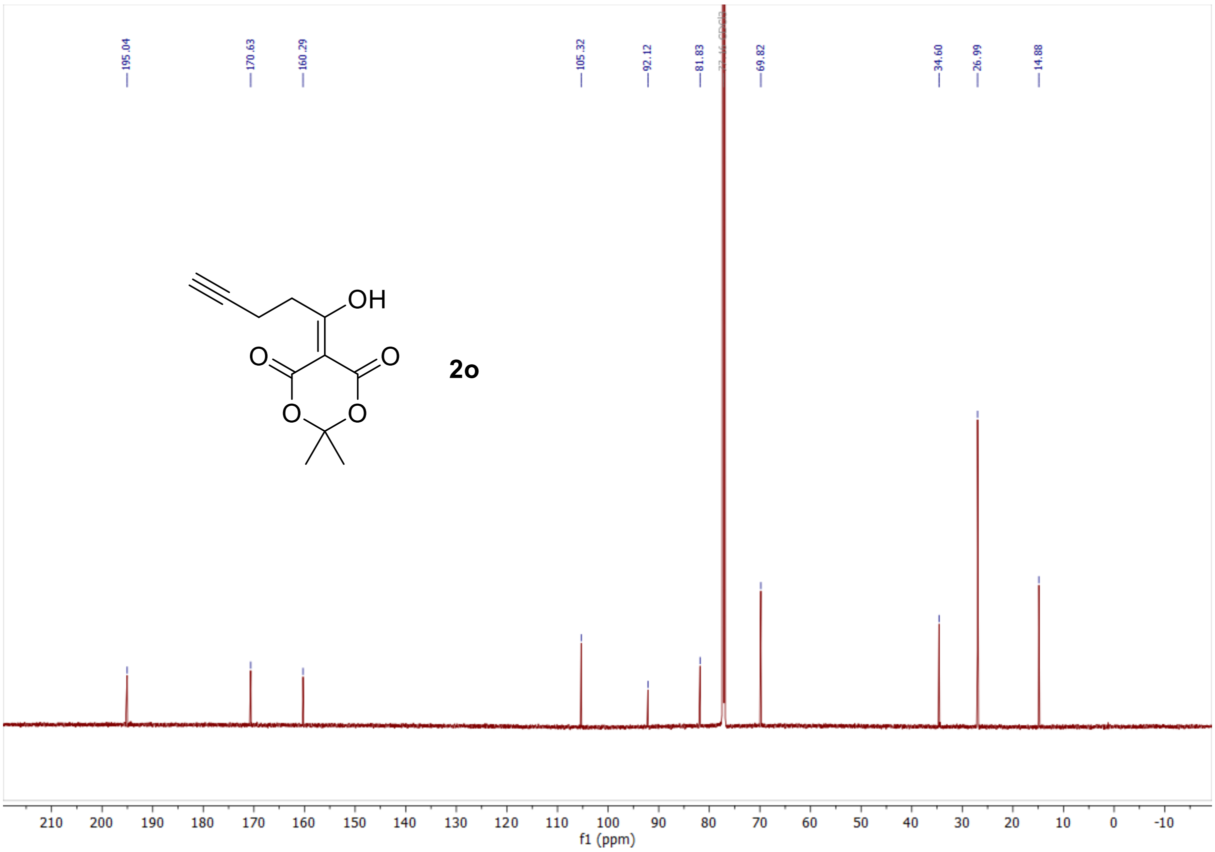

^{1}H NMR (600 MHz, $CDCl_3$) (2q)

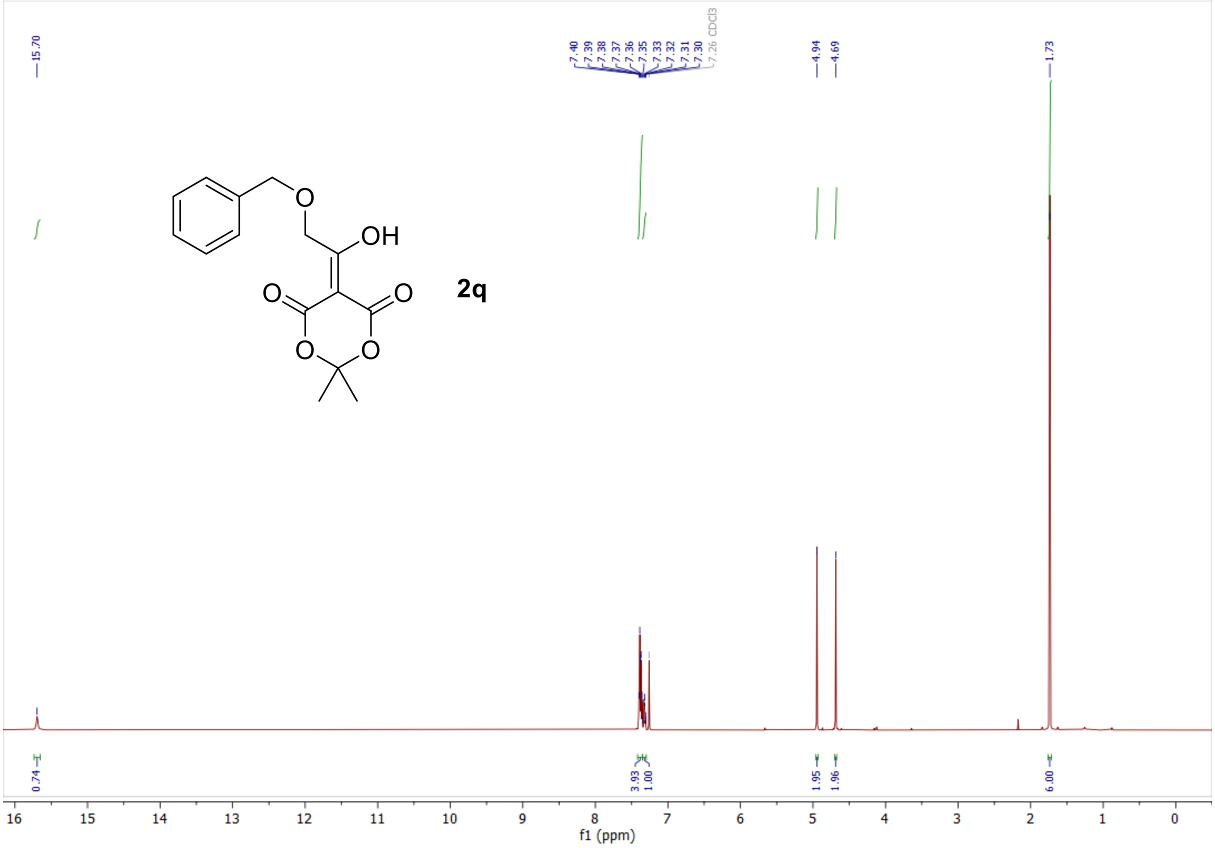

^{13}C NMR (151 MHz, $CDCl_3$) (2q)

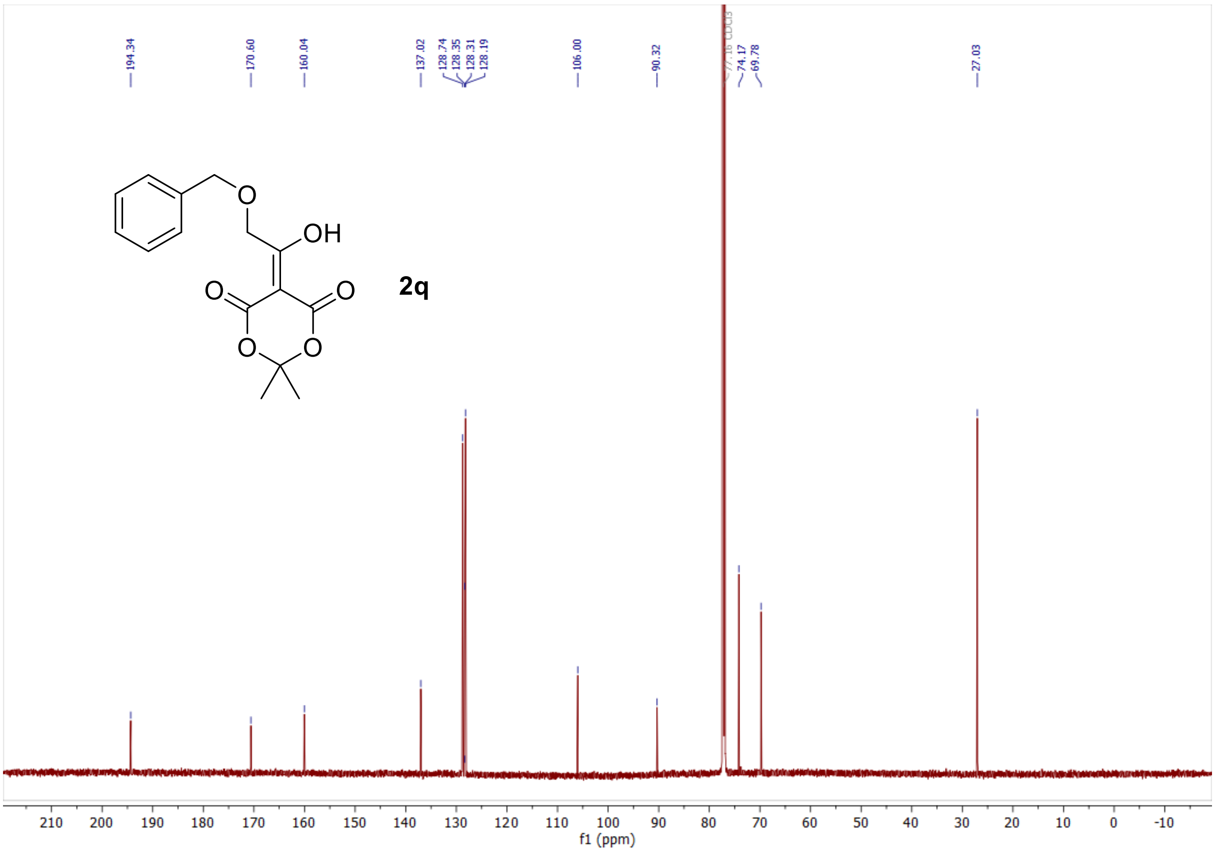

^{1}H NMR (400 MHz, $CDCl_3$) (3a)

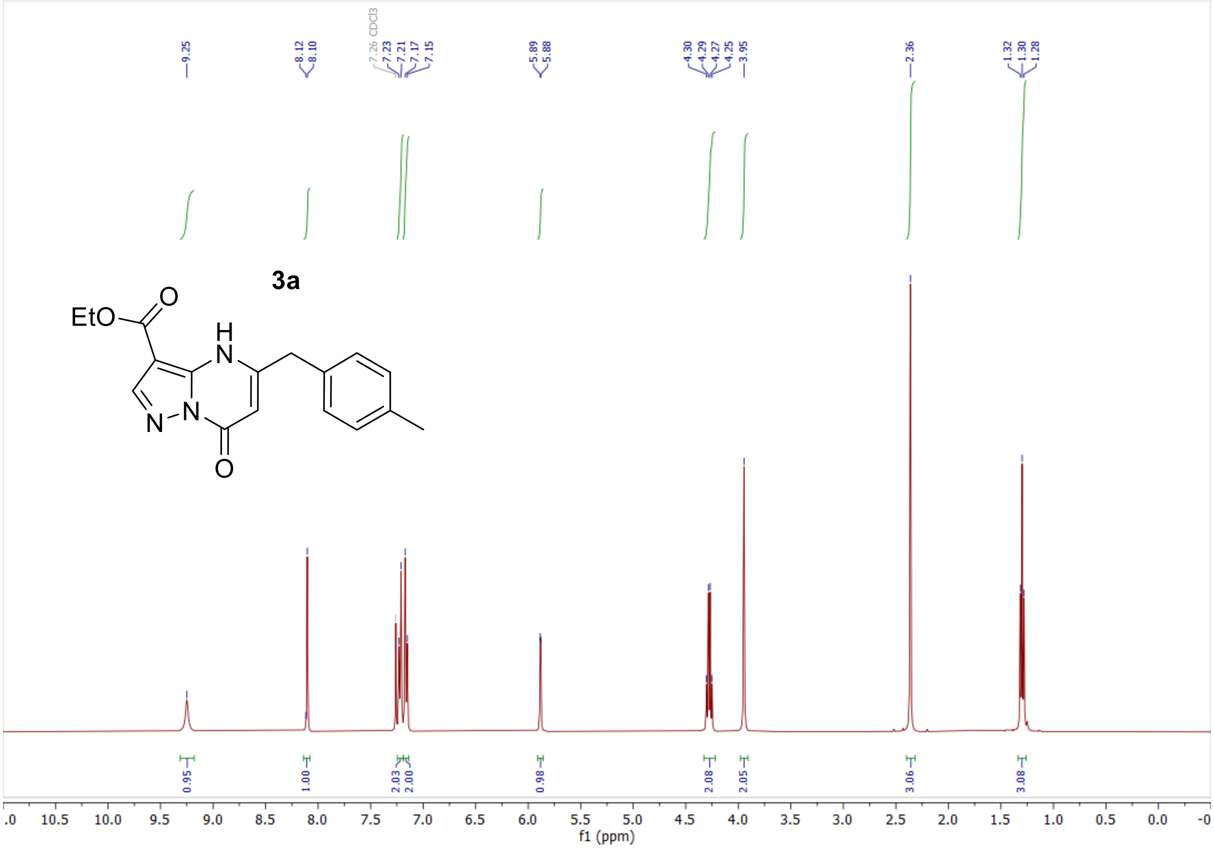

^{13}C NMR (100 MHz, $CDCl_3$) (3a)

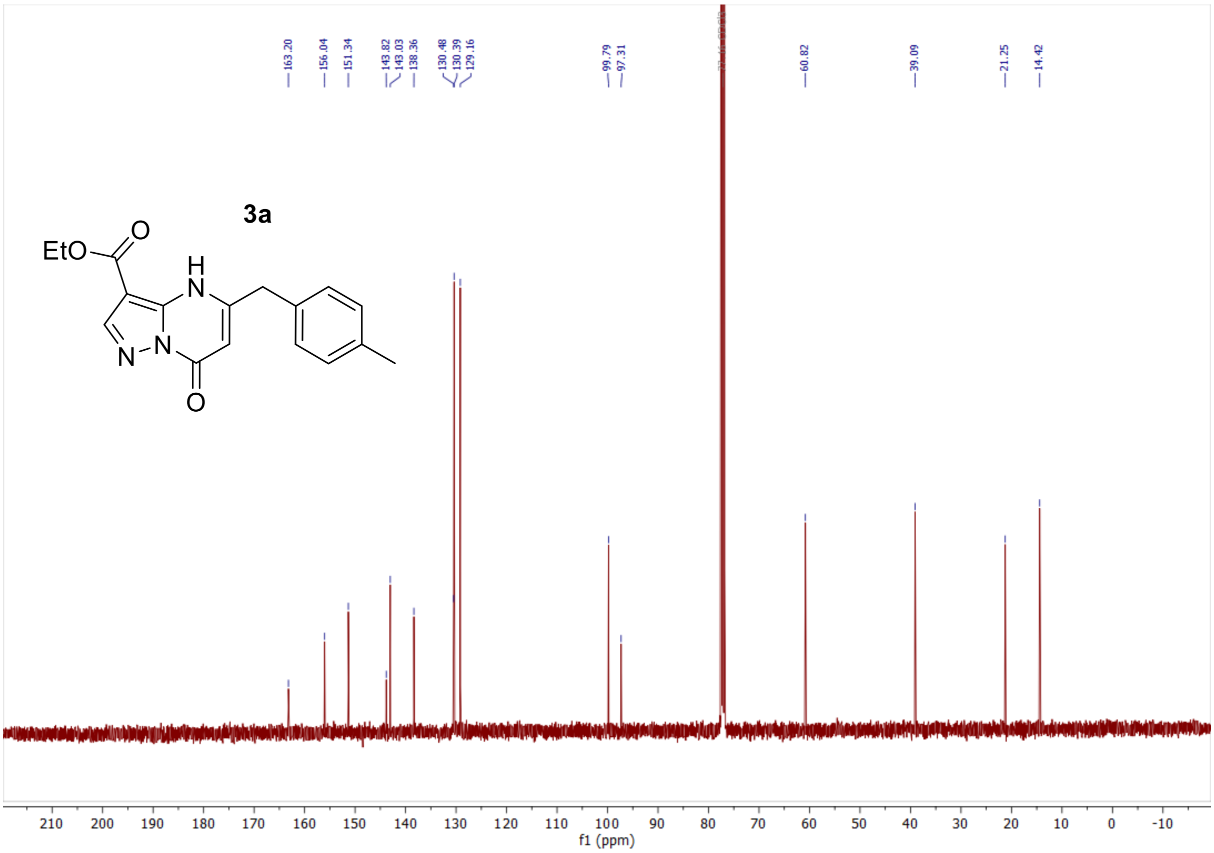

^{1}H NMR (400 MHz, $CDCl_3$) (3b)

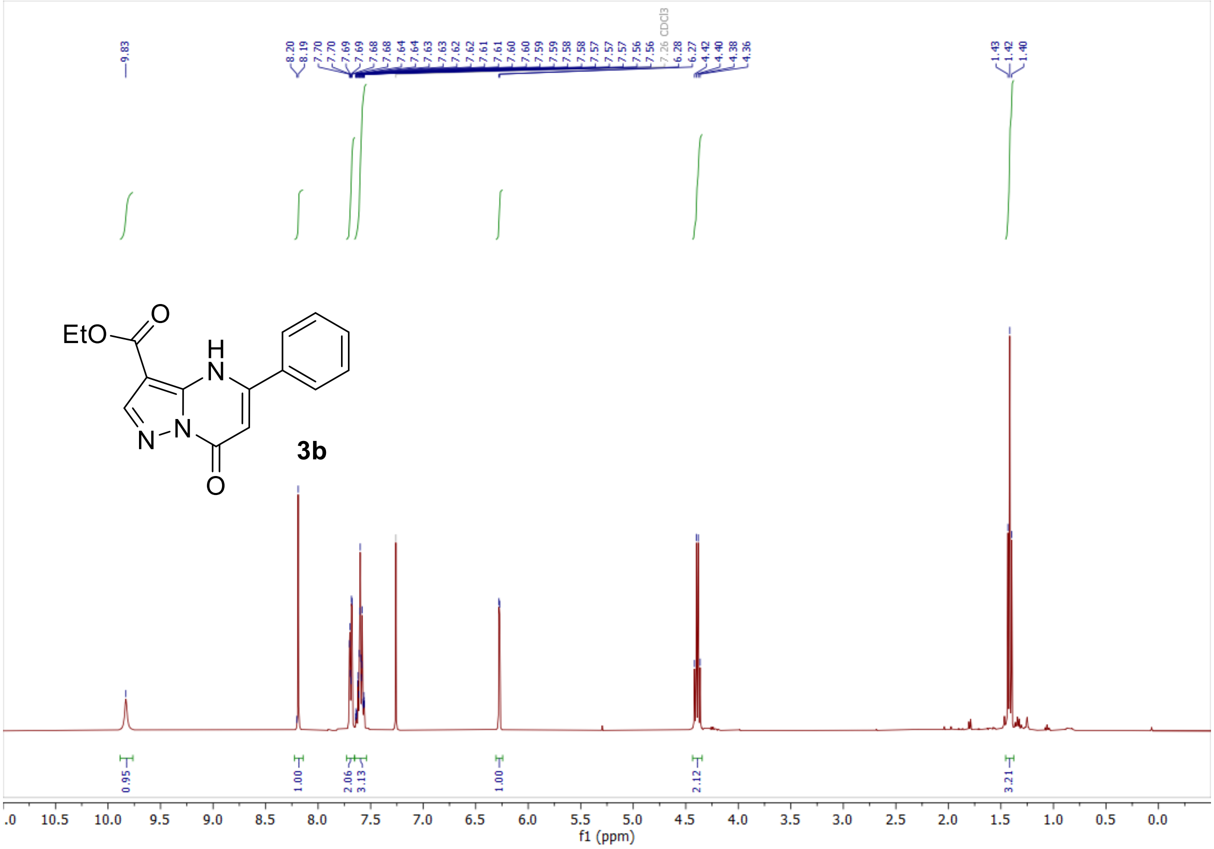

^{13}C NMR (100 MHz, $CDCl_3$) (3b)

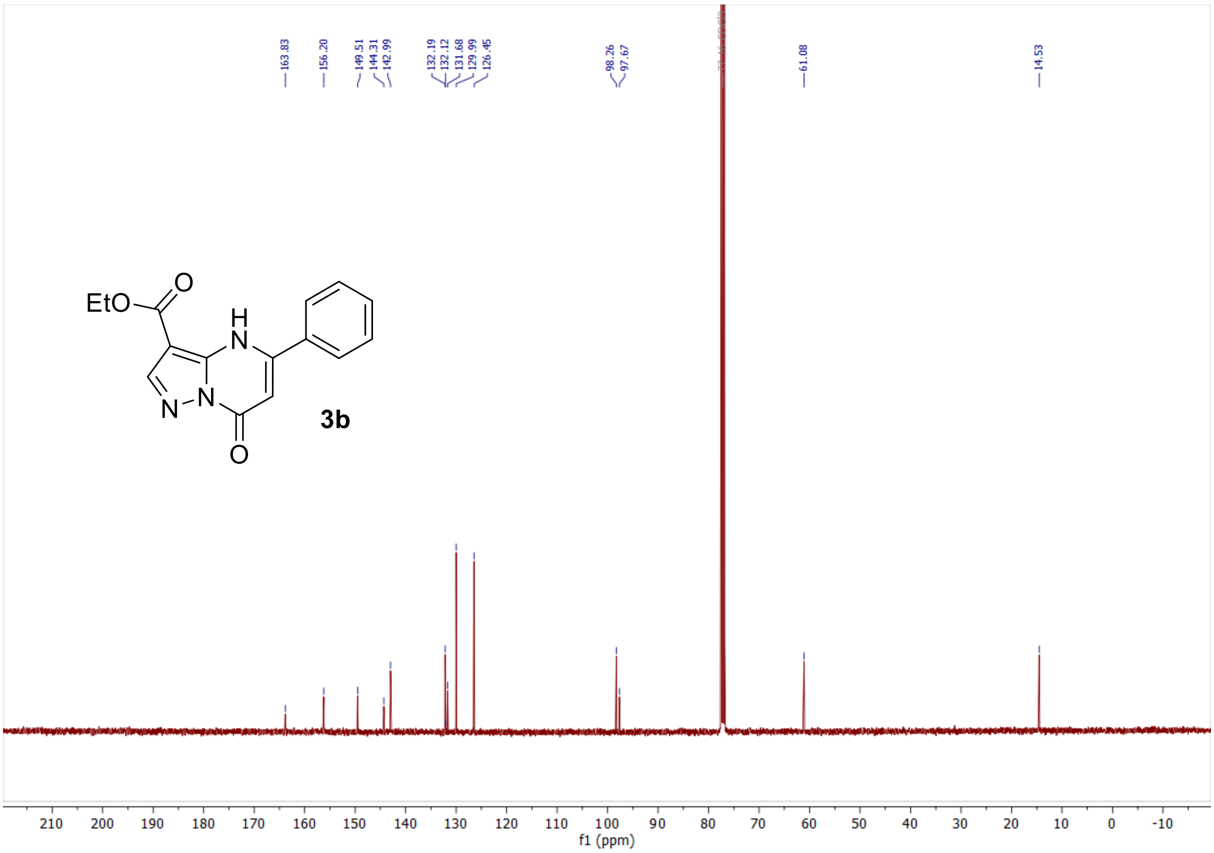

¹H NMR (600 MHz, $CDCl_3$) (3c)

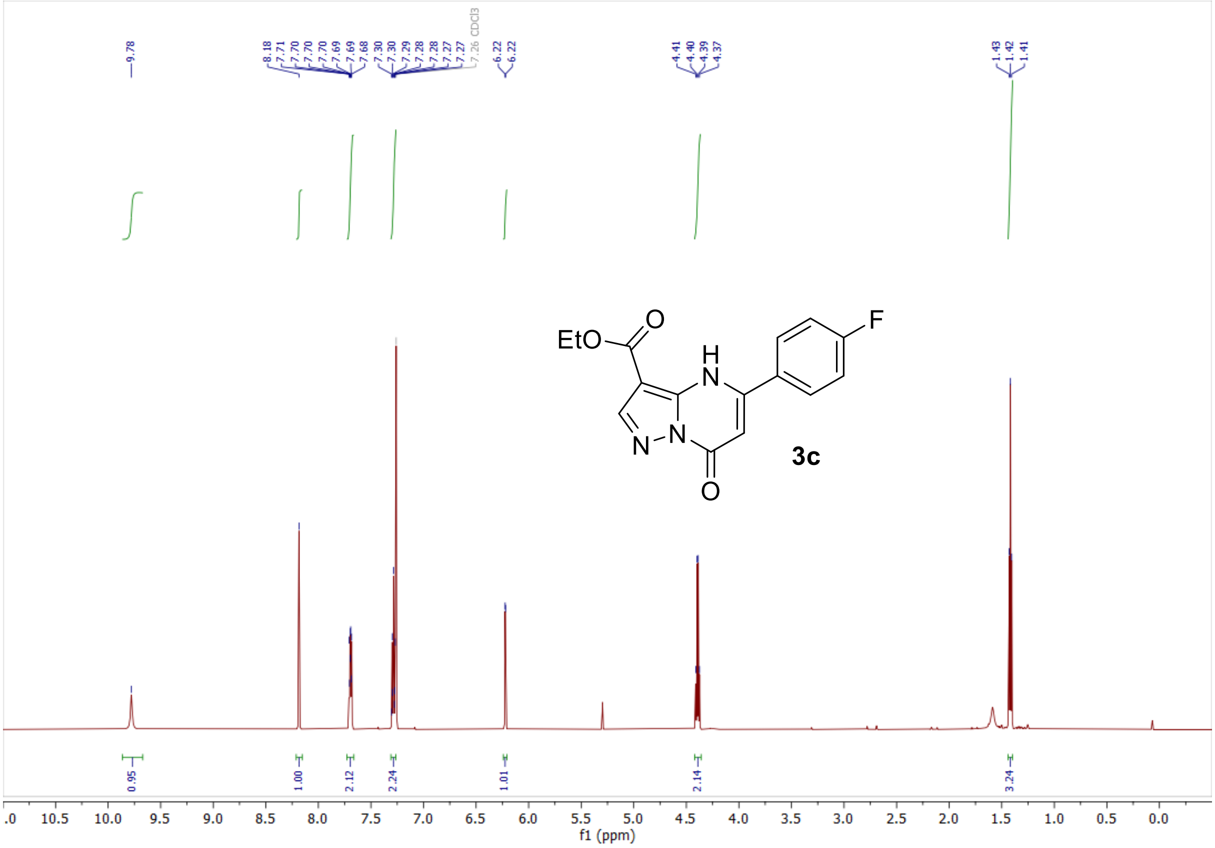

¹³C NMR (151 MHz, $CDCl_3$) (3c)

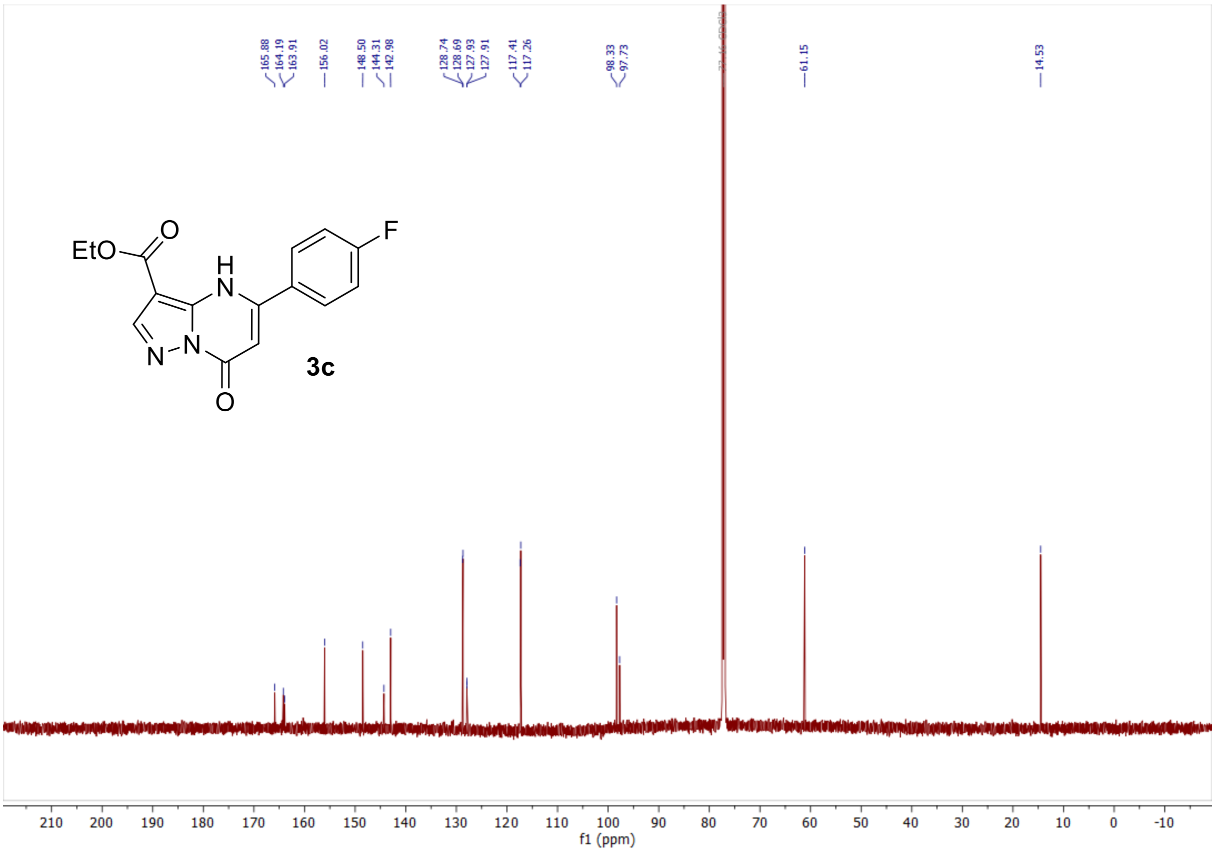

^{19}F NMR (565 MHz, CDCl$_3$) (3c)

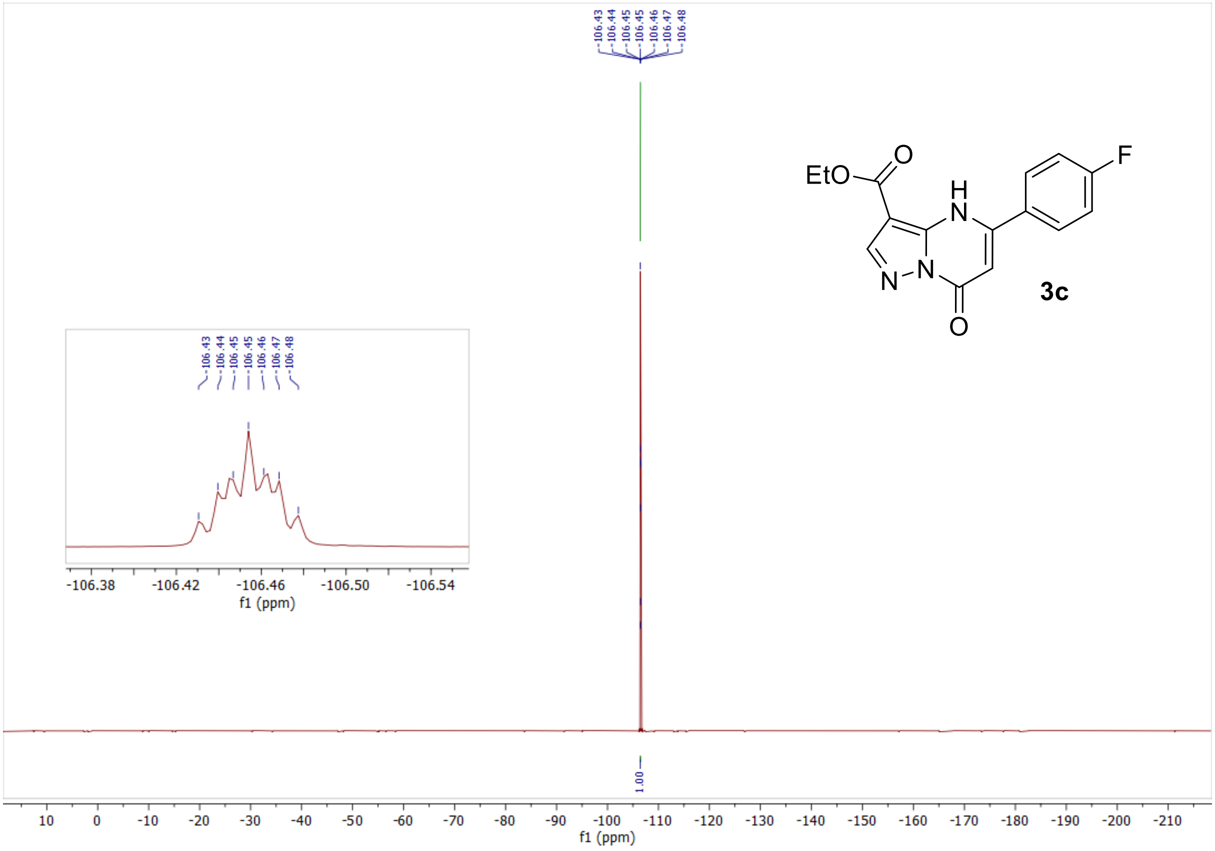

^{1}H NMR (600 MHz, CDCl$_3$) (3d)

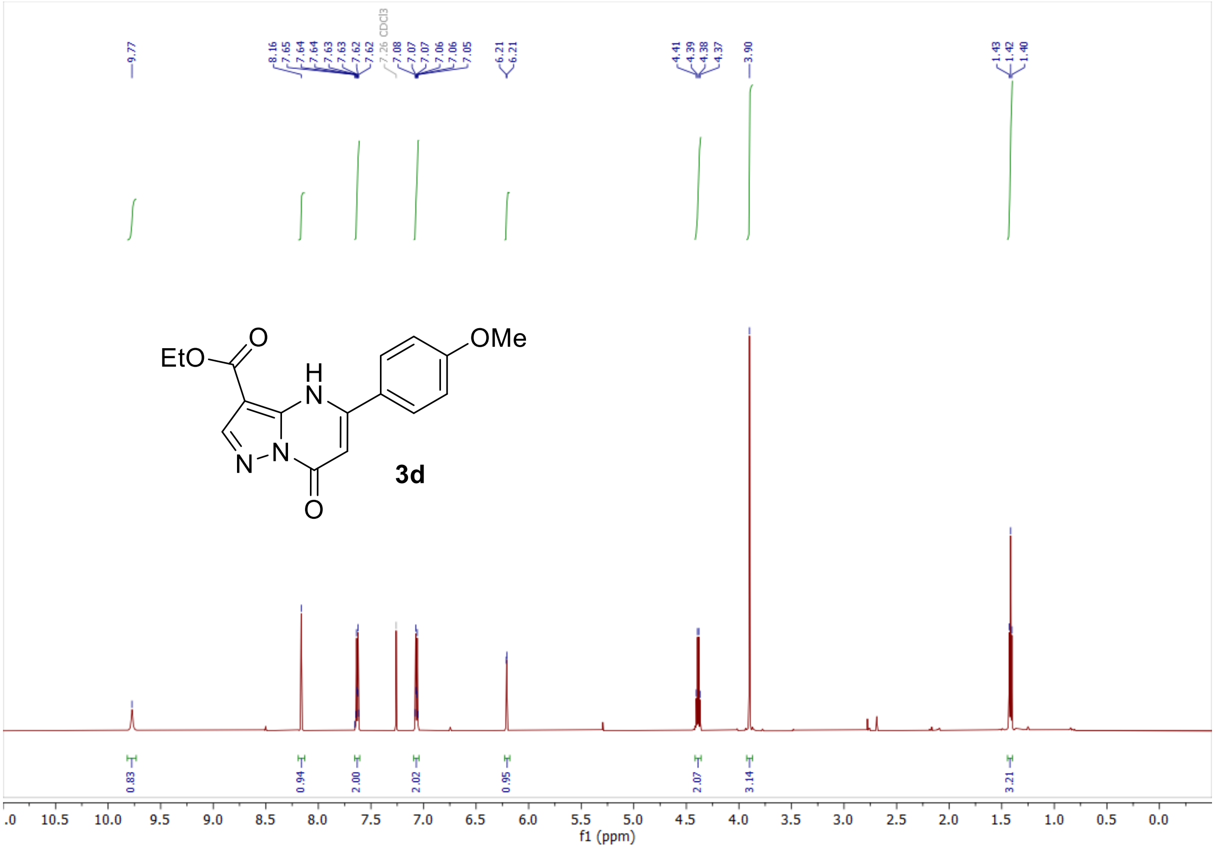

^{13}C NMR (151 MHz, CDCl$_3$) (3d)

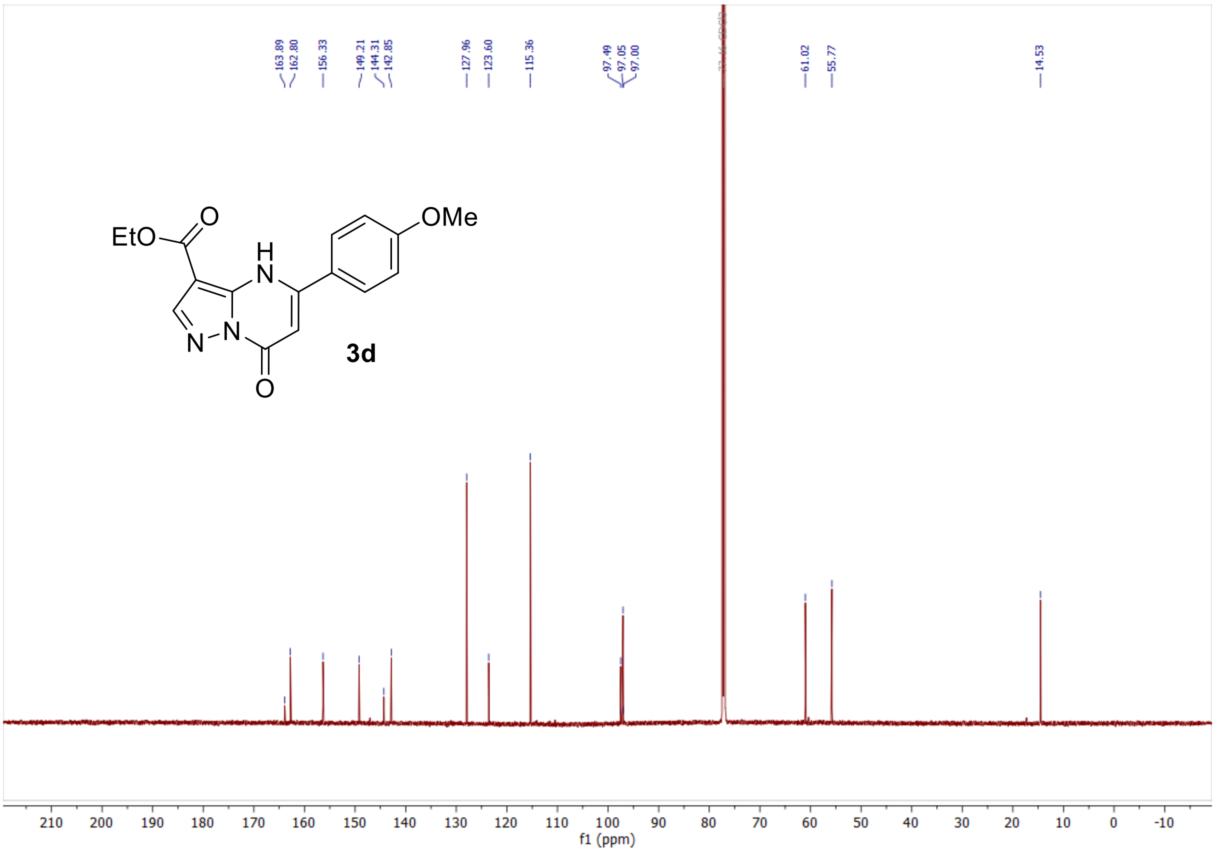

^{1}H NMR (600 MHz, CDCl$_3$) (3e)

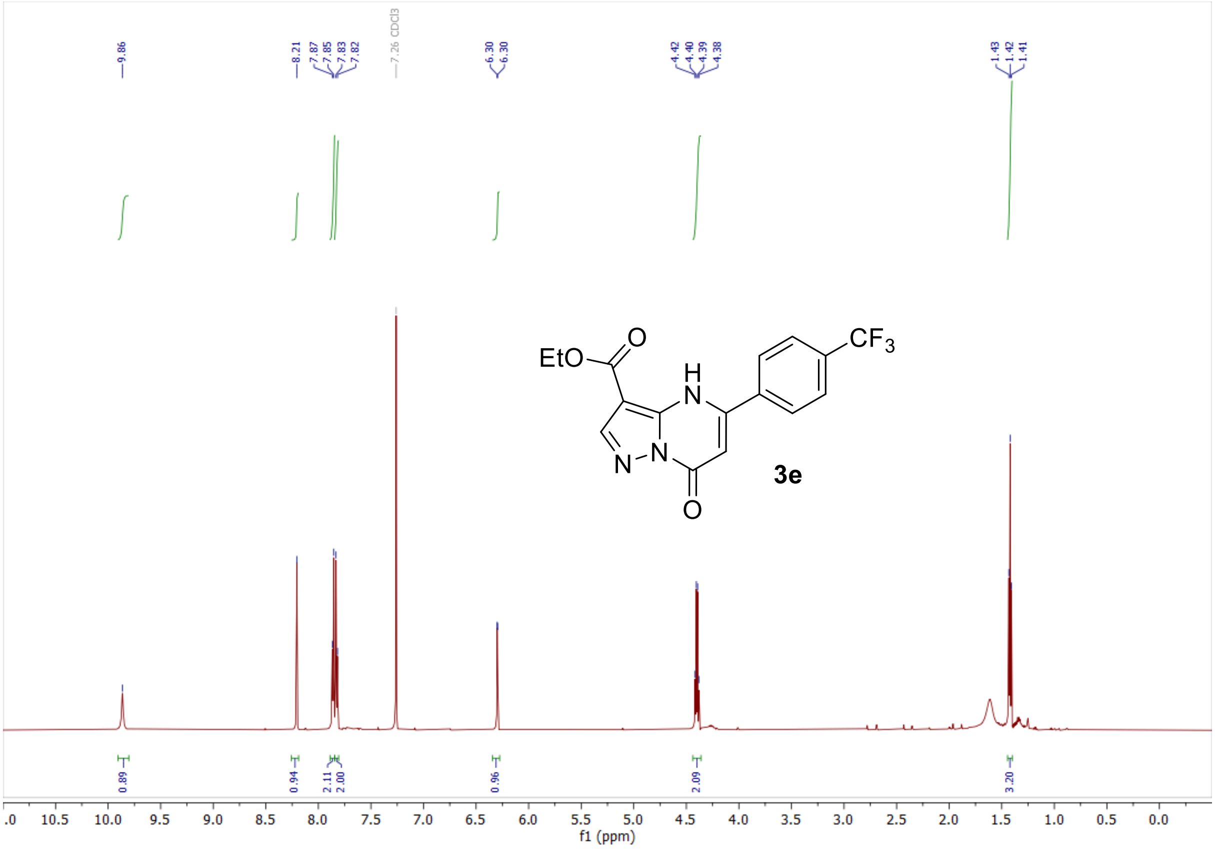

^{13}C NMR (151 MHz, $CDCl_3$) (3e)

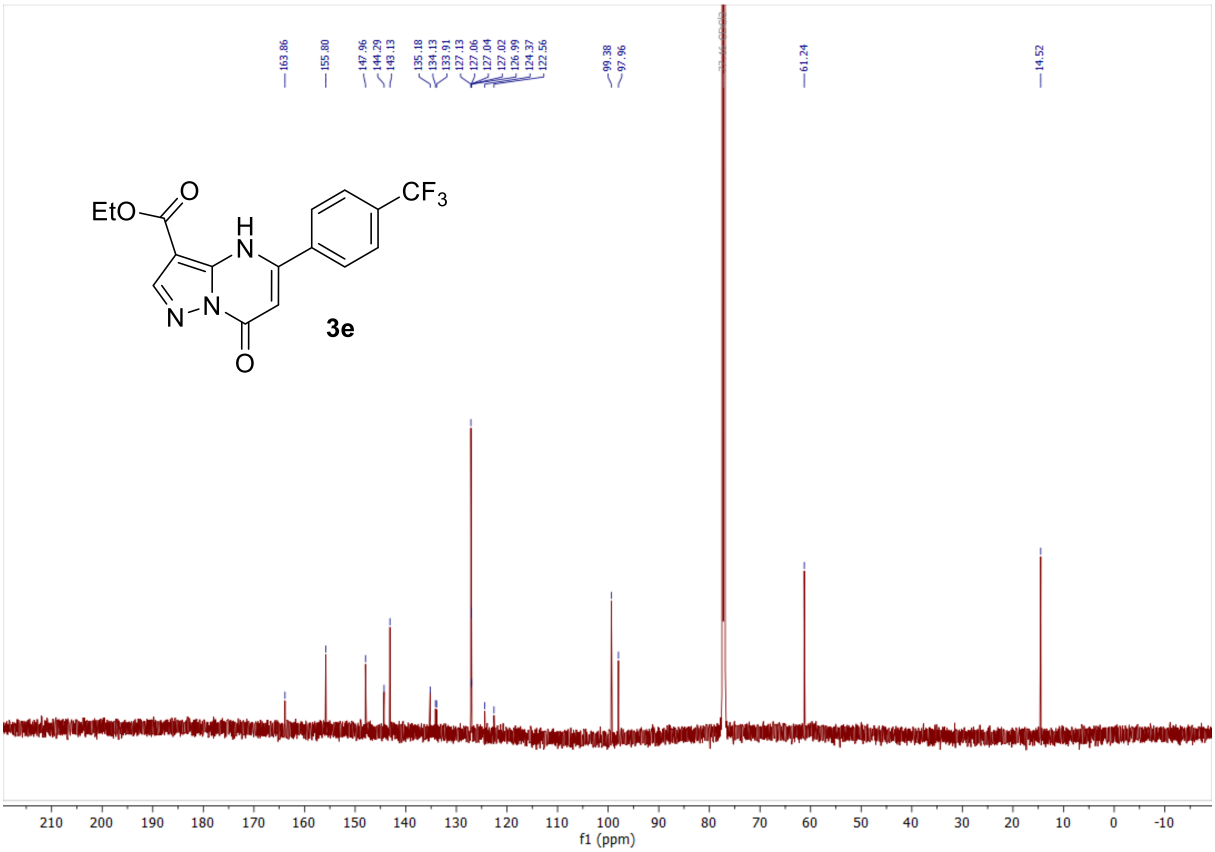

^{19}F NMR (565 MHz, $CDCl_3$) (3e)

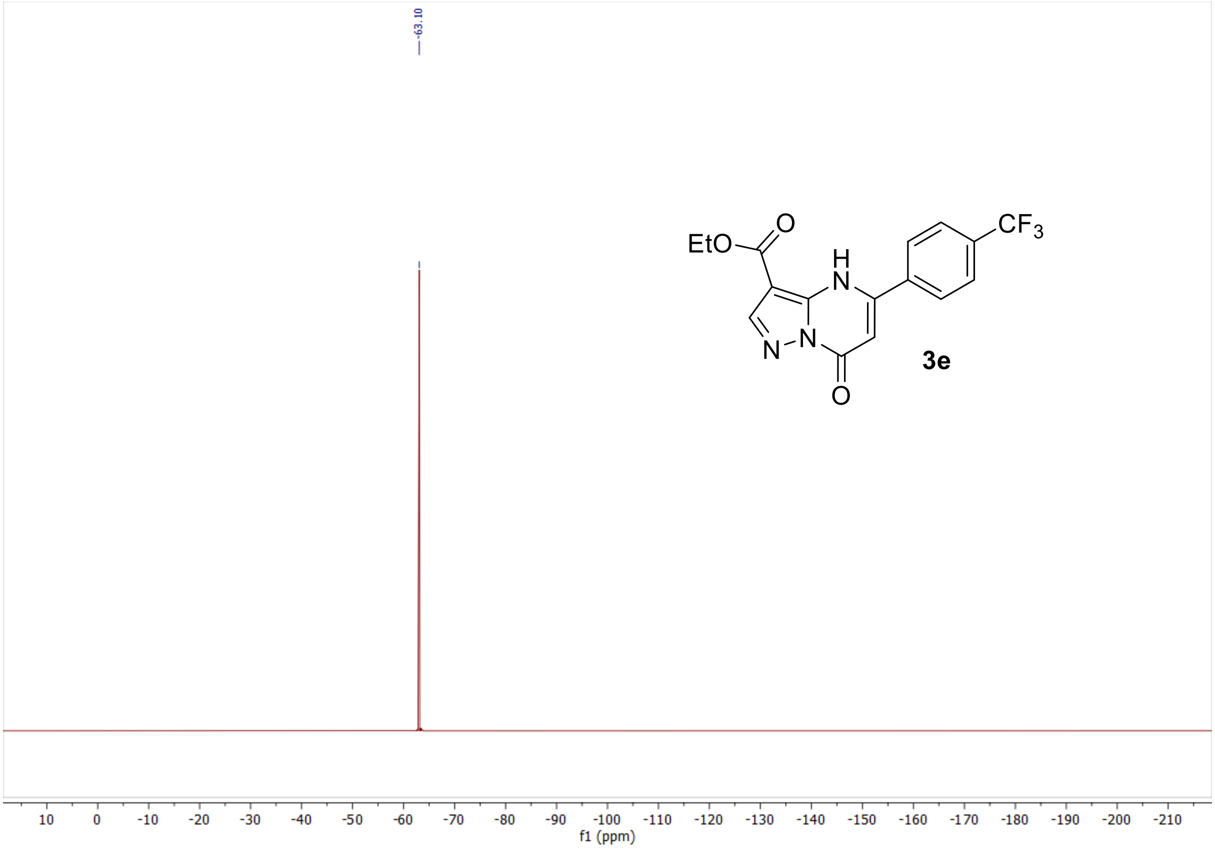

^{1}H NMR (600 MHz, $CDCl_3$) (3f)

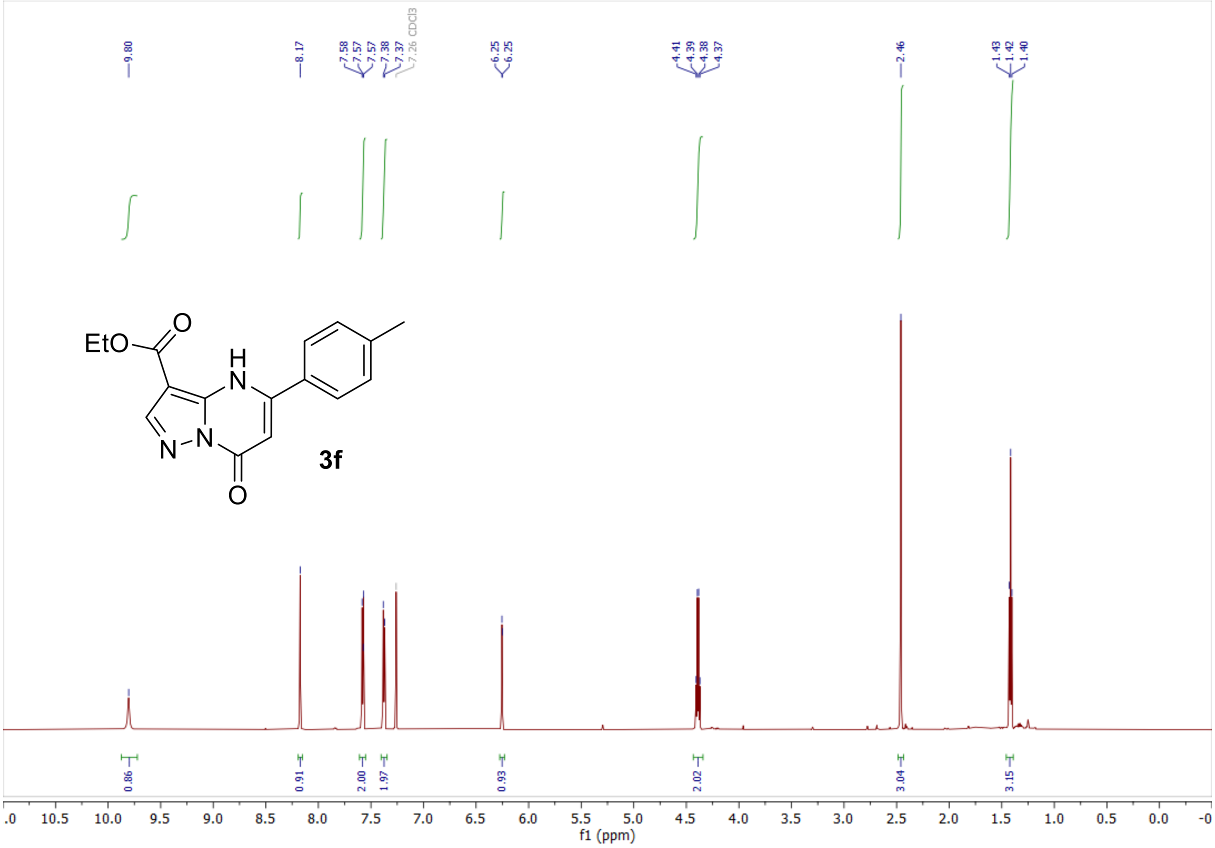

^{13}C NMR (151 MHz, $CDCl_3$) (3f)

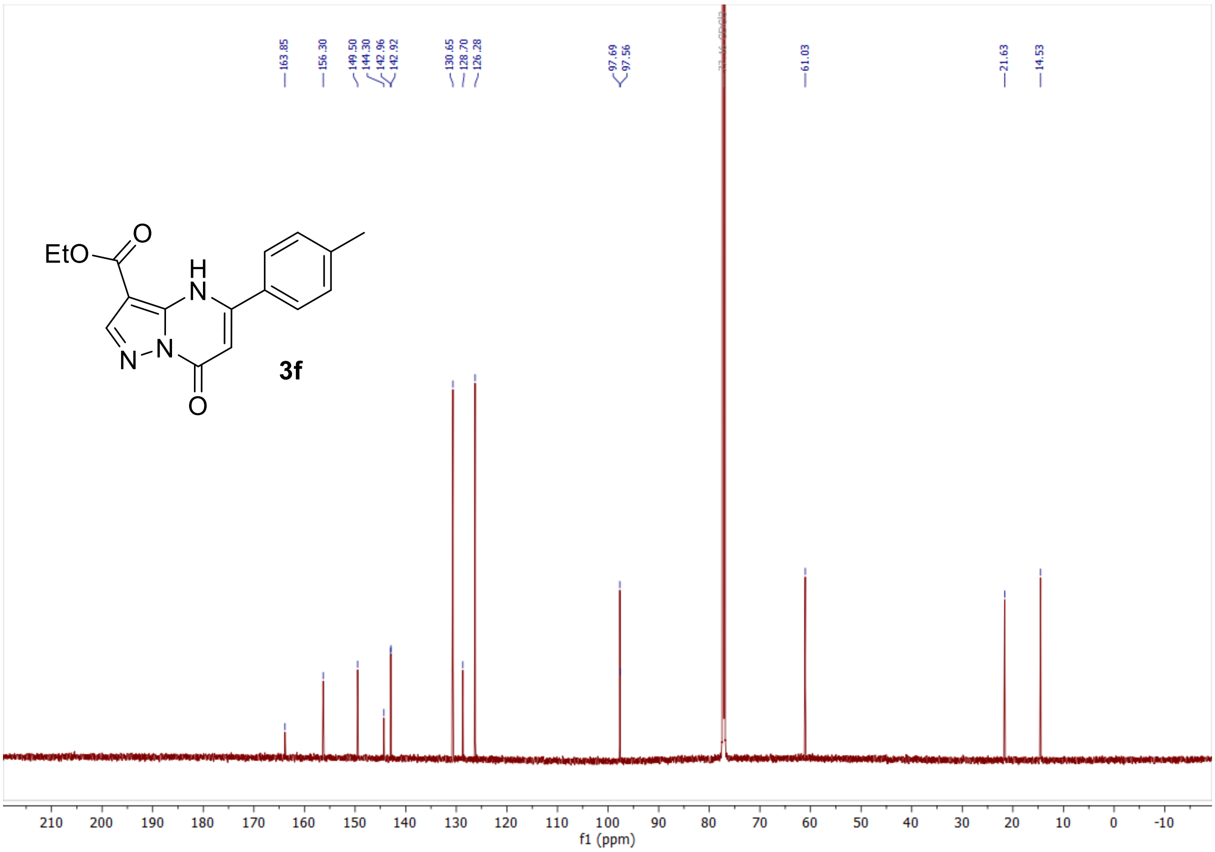

^{1}H NMR (600 MHz, $CDCl_3$) (3g)

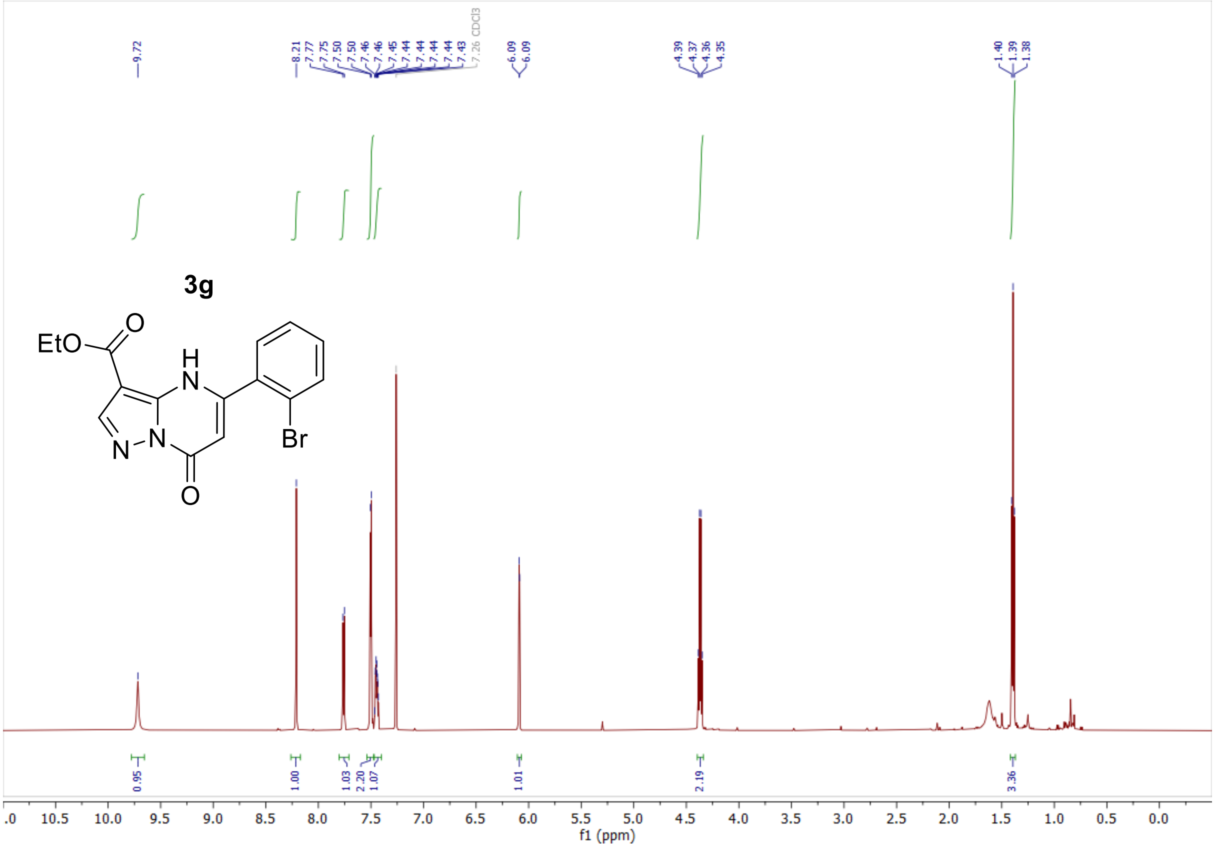

^{13}C NMR (151 MHz, $CDCl_3$) (3g)

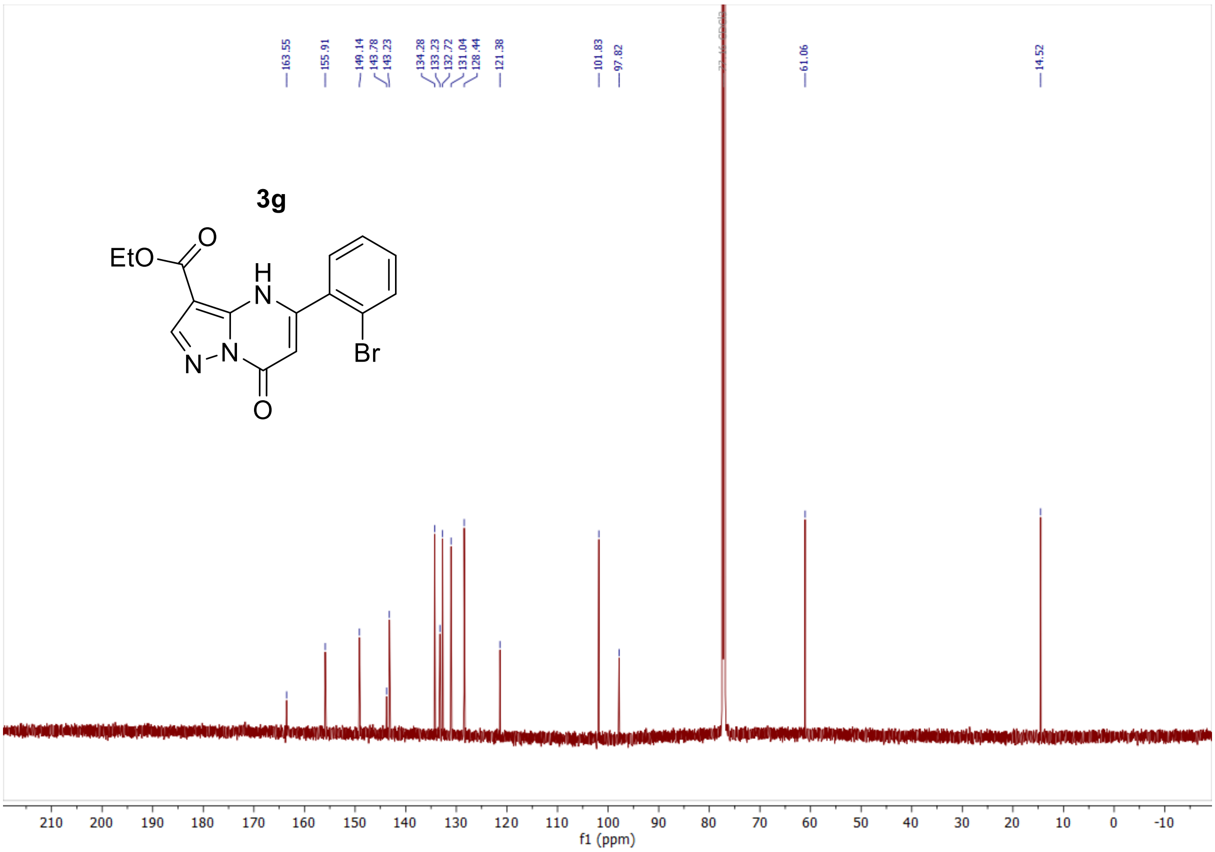

^{1}H NMR (400 MHz, $CDCl_3$) (3h)

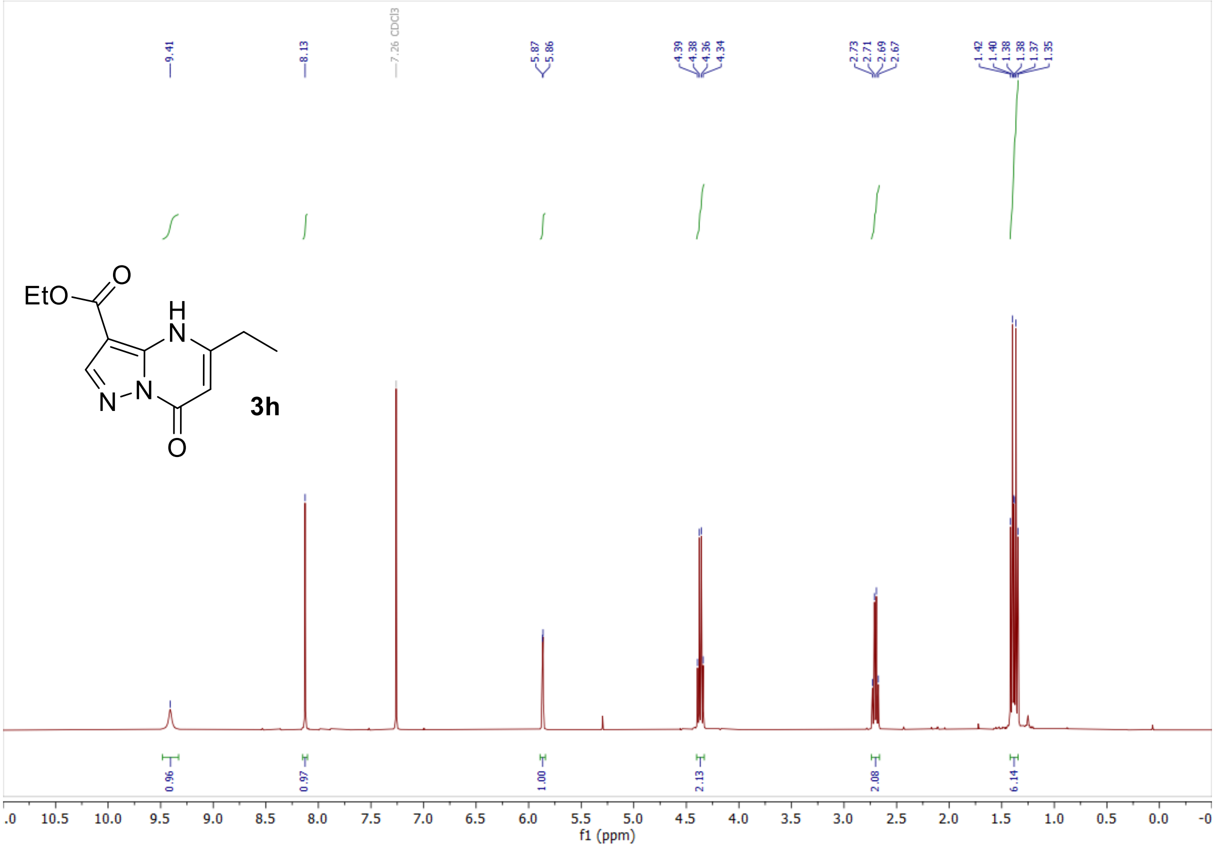

^{13}C NMR (100 MHz, $CDCl_3$) (3h)

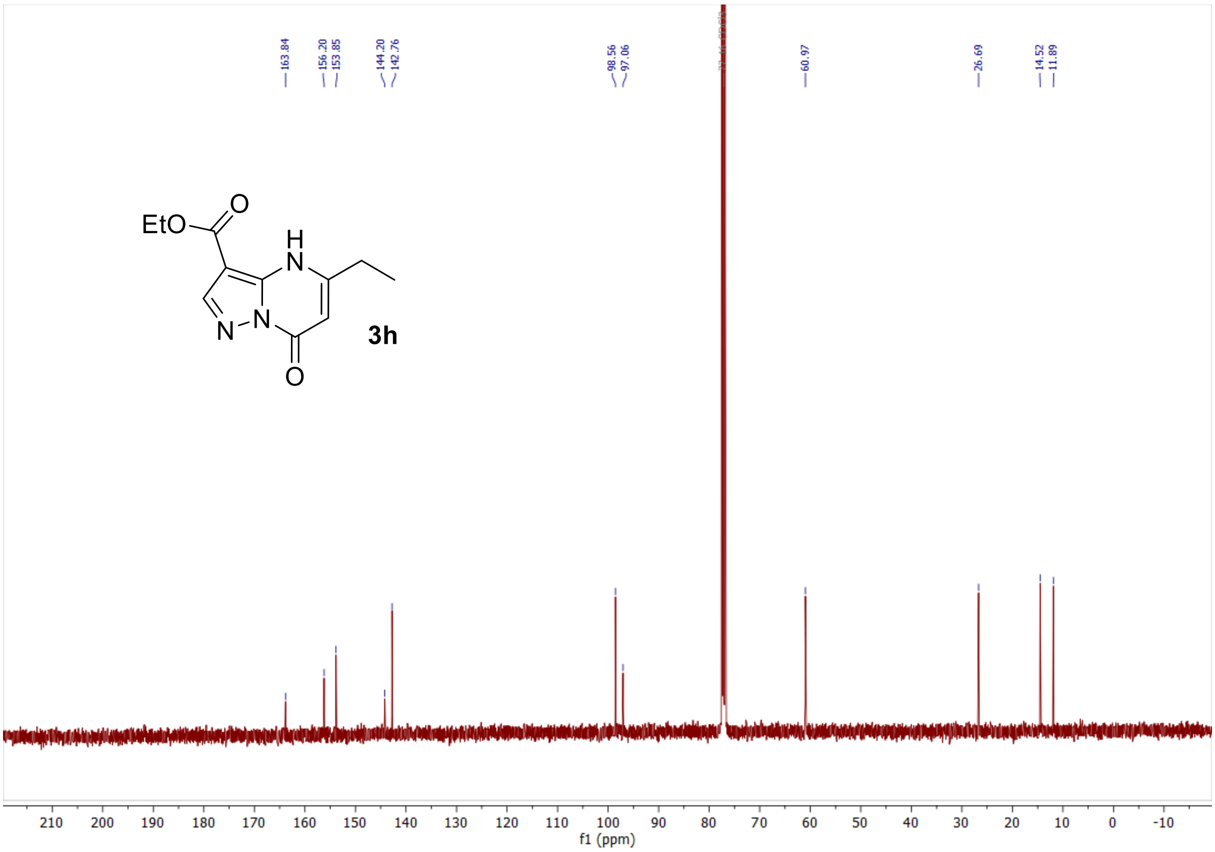

^{1}H NMR (600 MHz, $CDCl_3$) (3i)

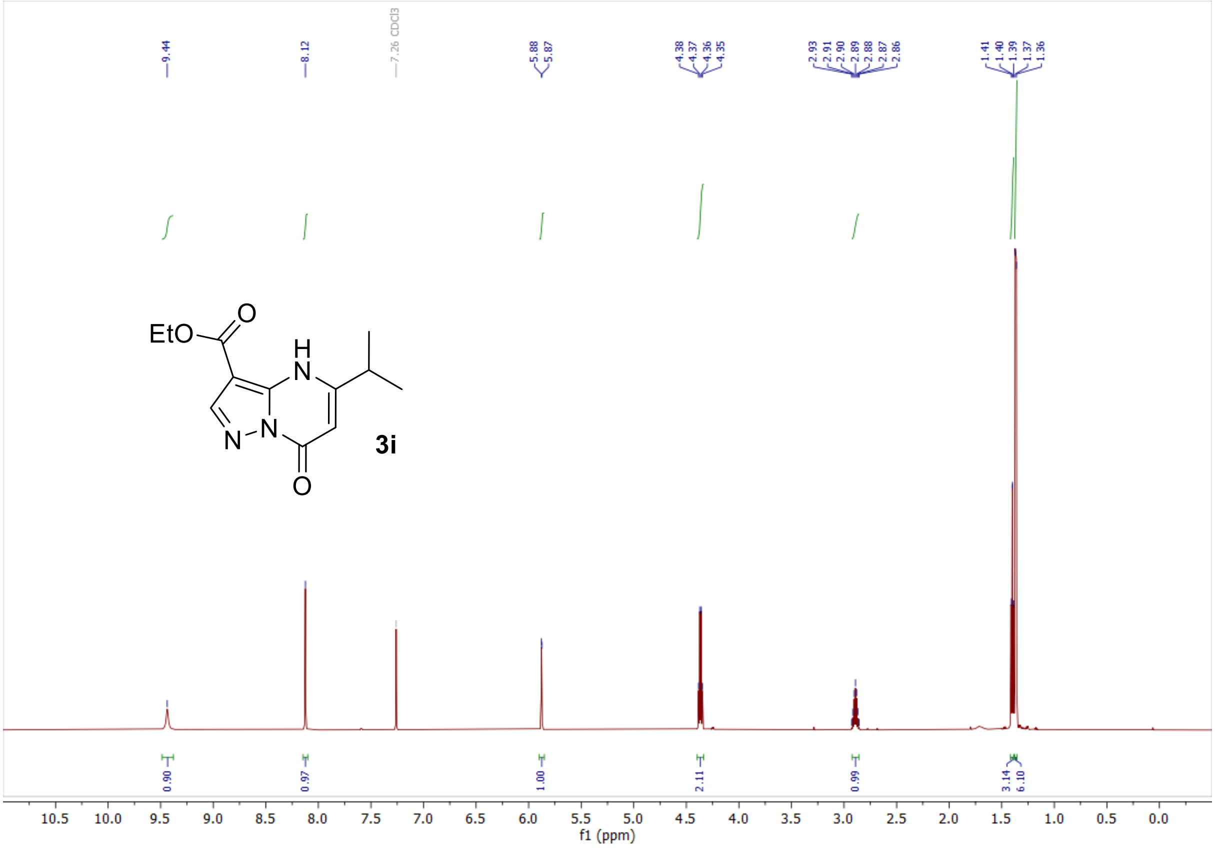

^{13}C NMR (151 MHz, $CDCl_3$) (3i)

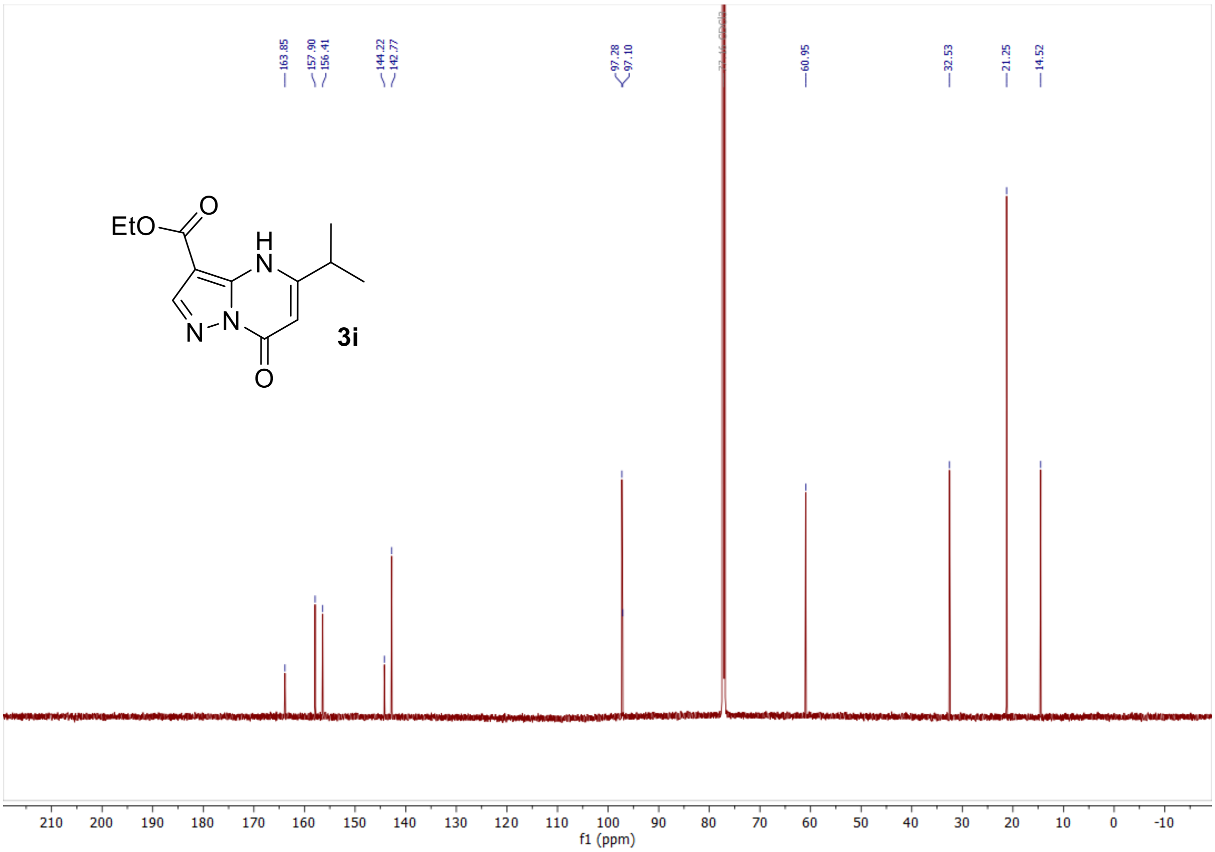

^{1}H NMR (600 MHz, $CDCl_3$) (3j)

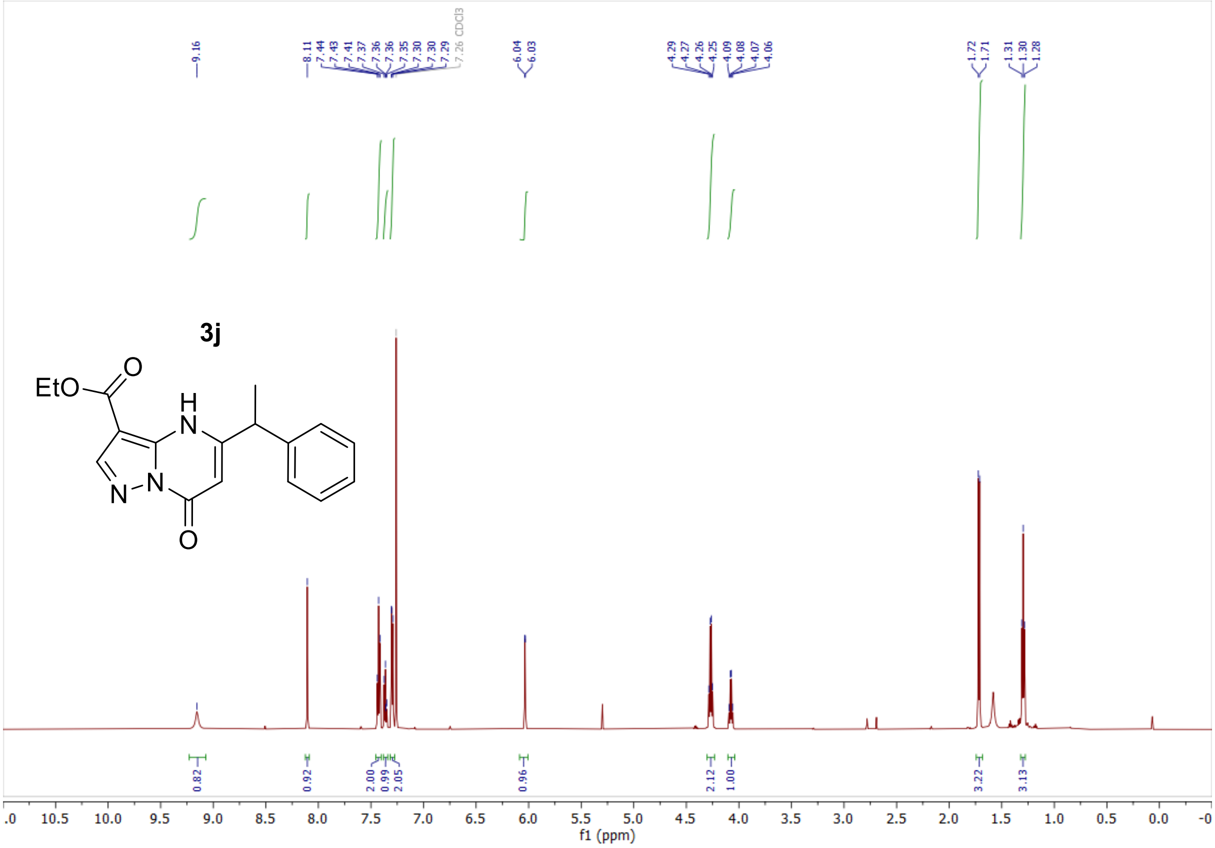

^{13}C NMR (151 MHz, $CDCl_3$) (3j)

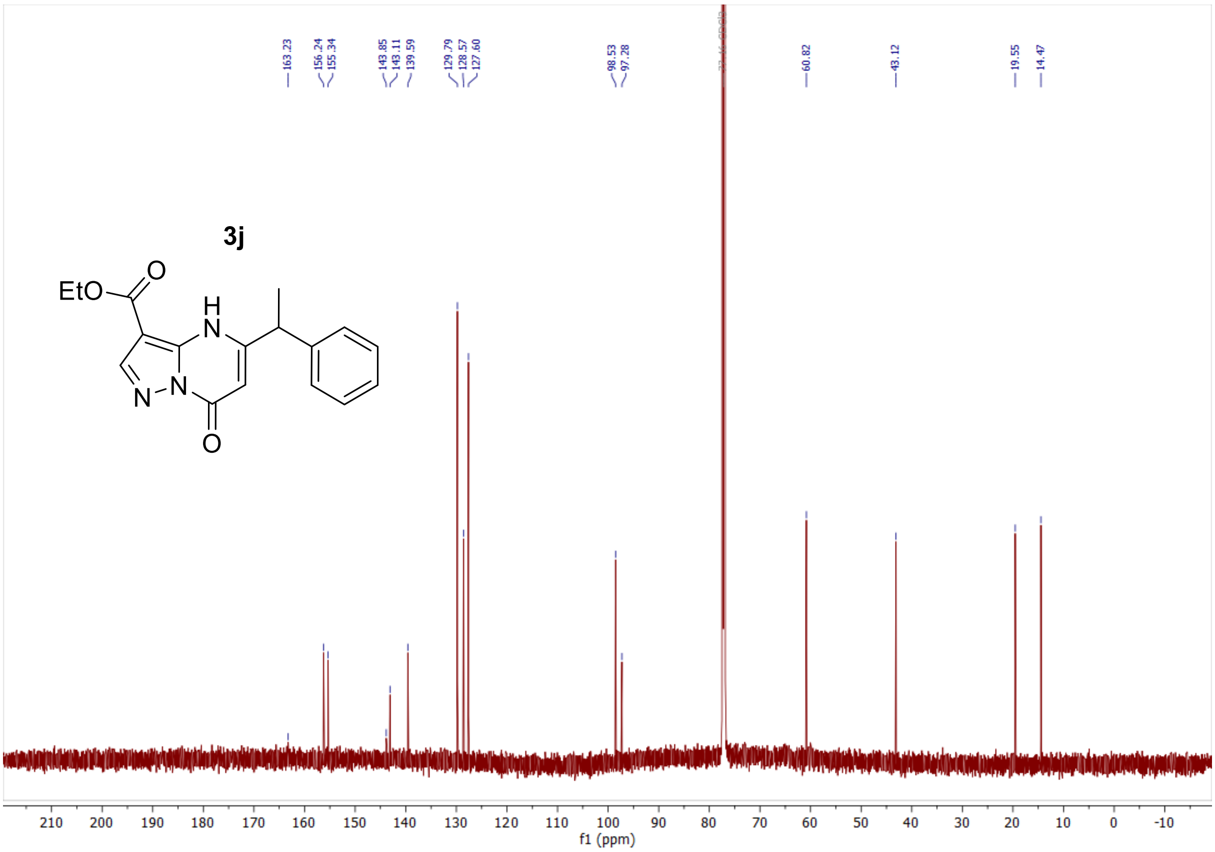

^{1}H NMR (400 MHz, $CDCl_3$) (3k)

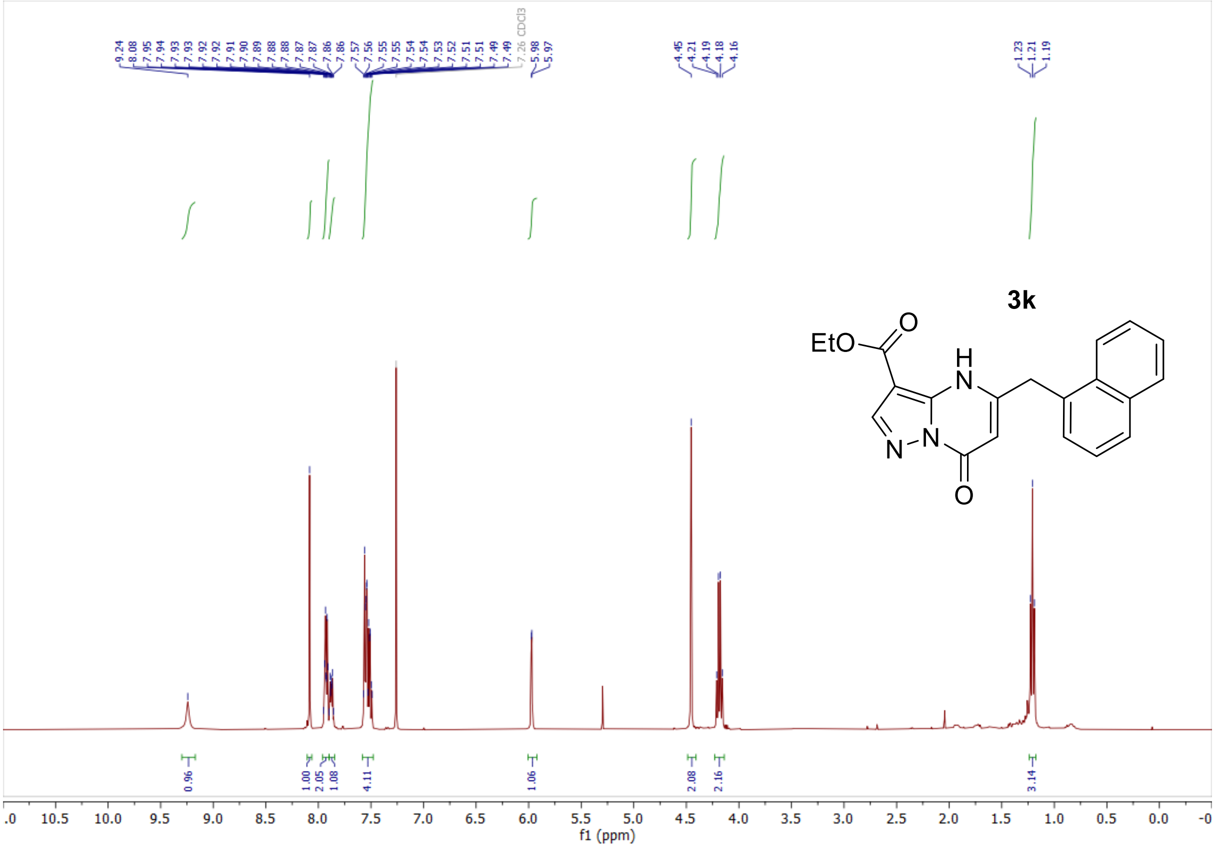

^{13}C NMR (100 MHz, $CDCl_3$) (3k)

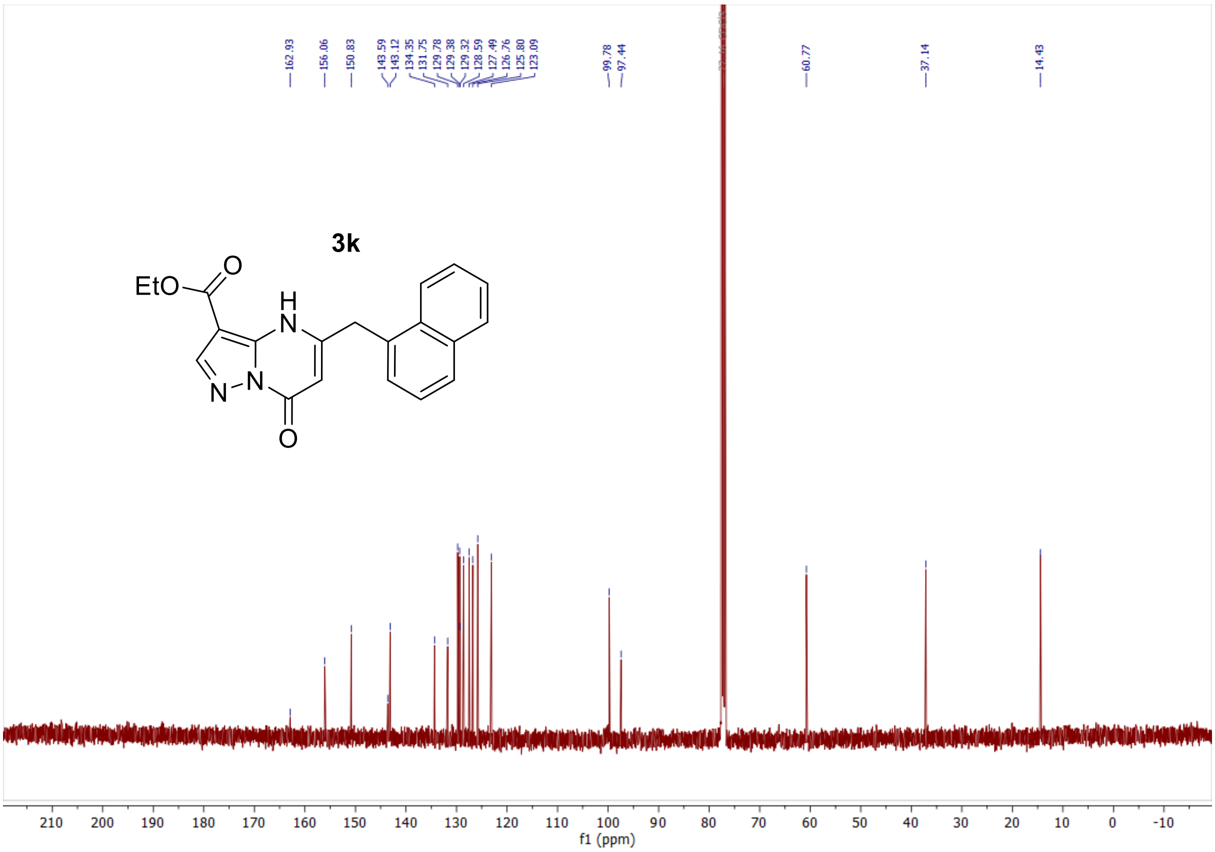

^{1}H NMR (600 MHz, $CDCl_3$) (3l)

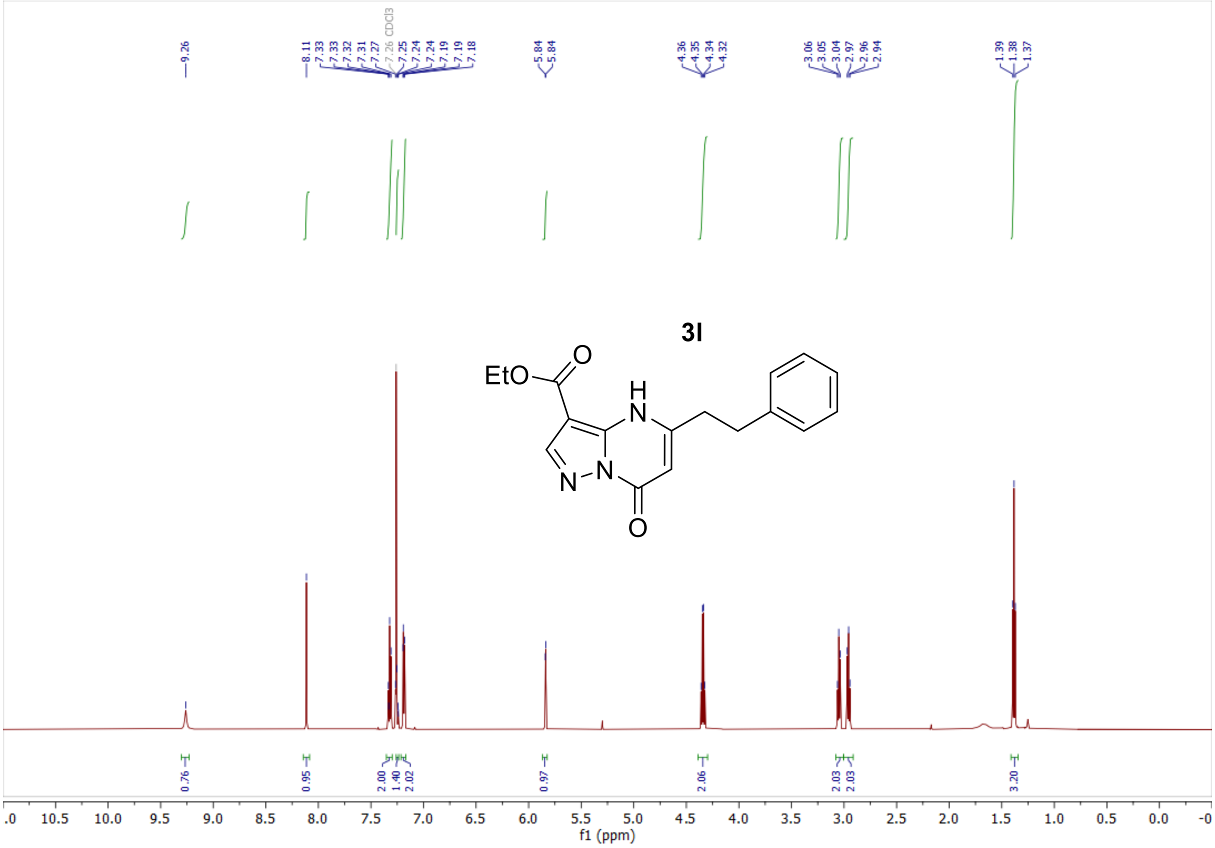

^{13}C NMR (151 MHz, $CDCl_3$) (3l)

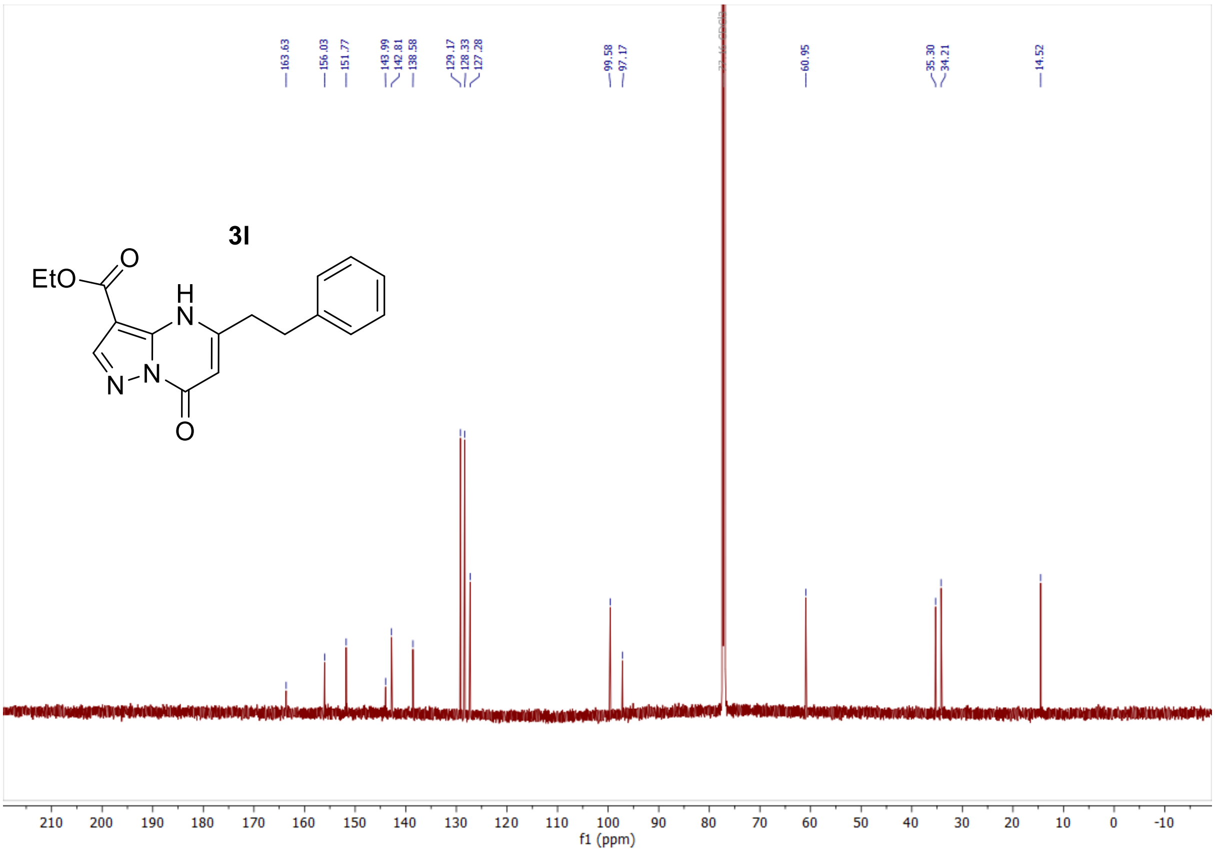

^{1}H NMR (400 MHz, $CDCl_3$) (3m)

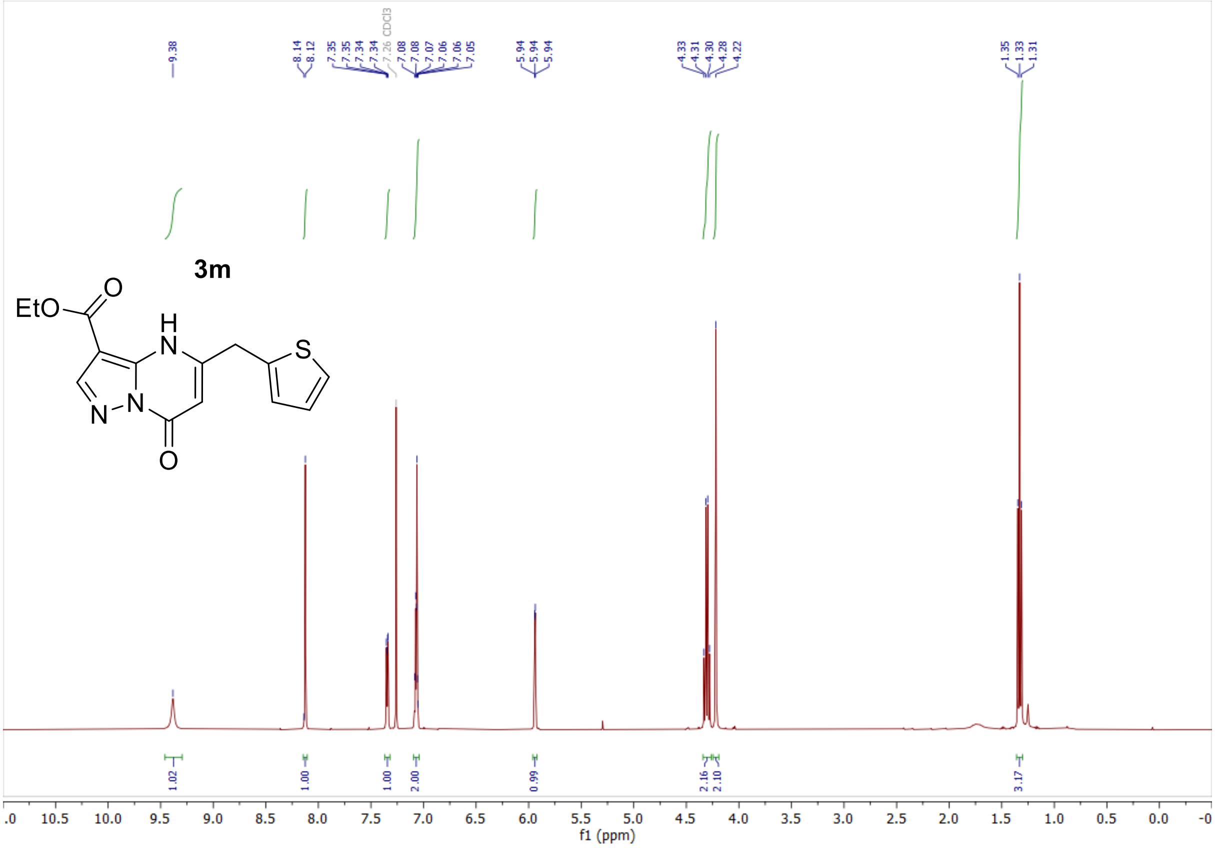

^{13}C NMR (100 MHz, $CDCl_3$) (3m)

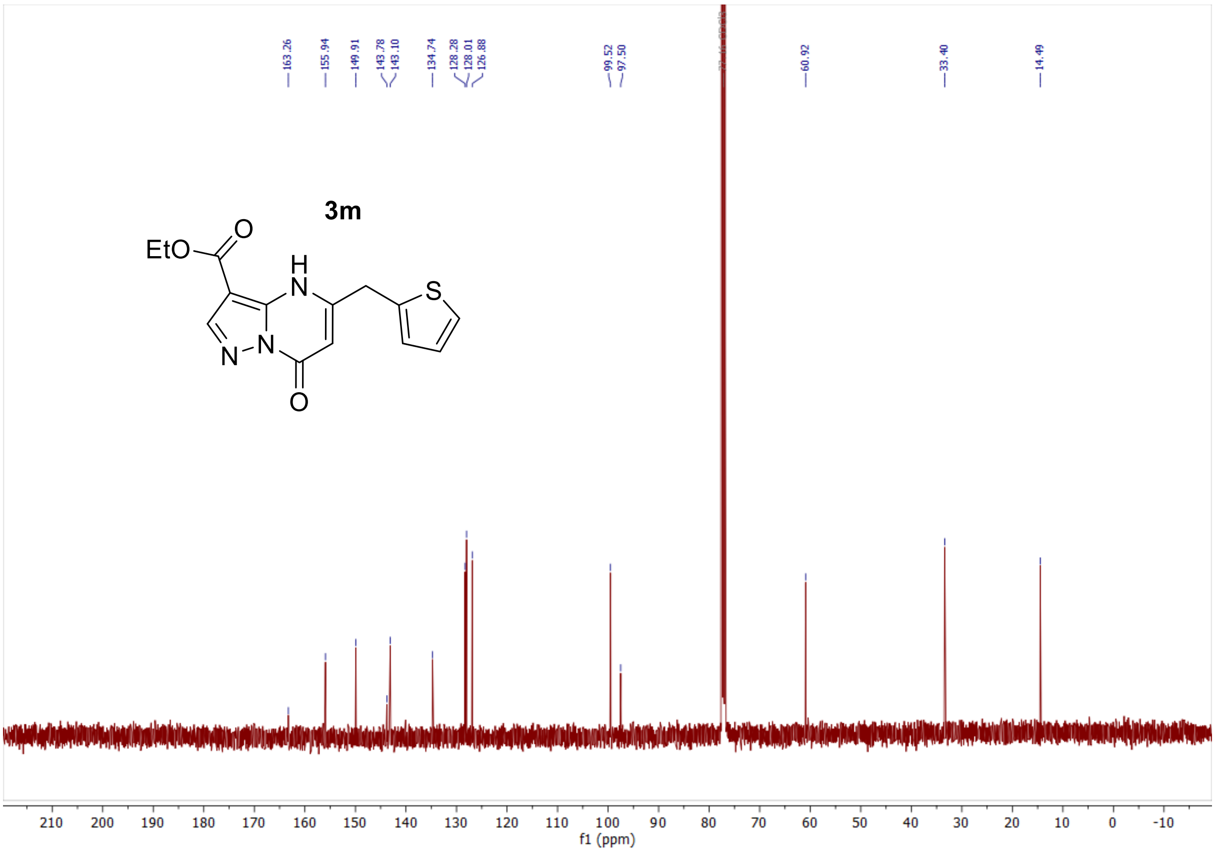

^{1}H NMR (600 MHz, $CDCl_3$) (3n)

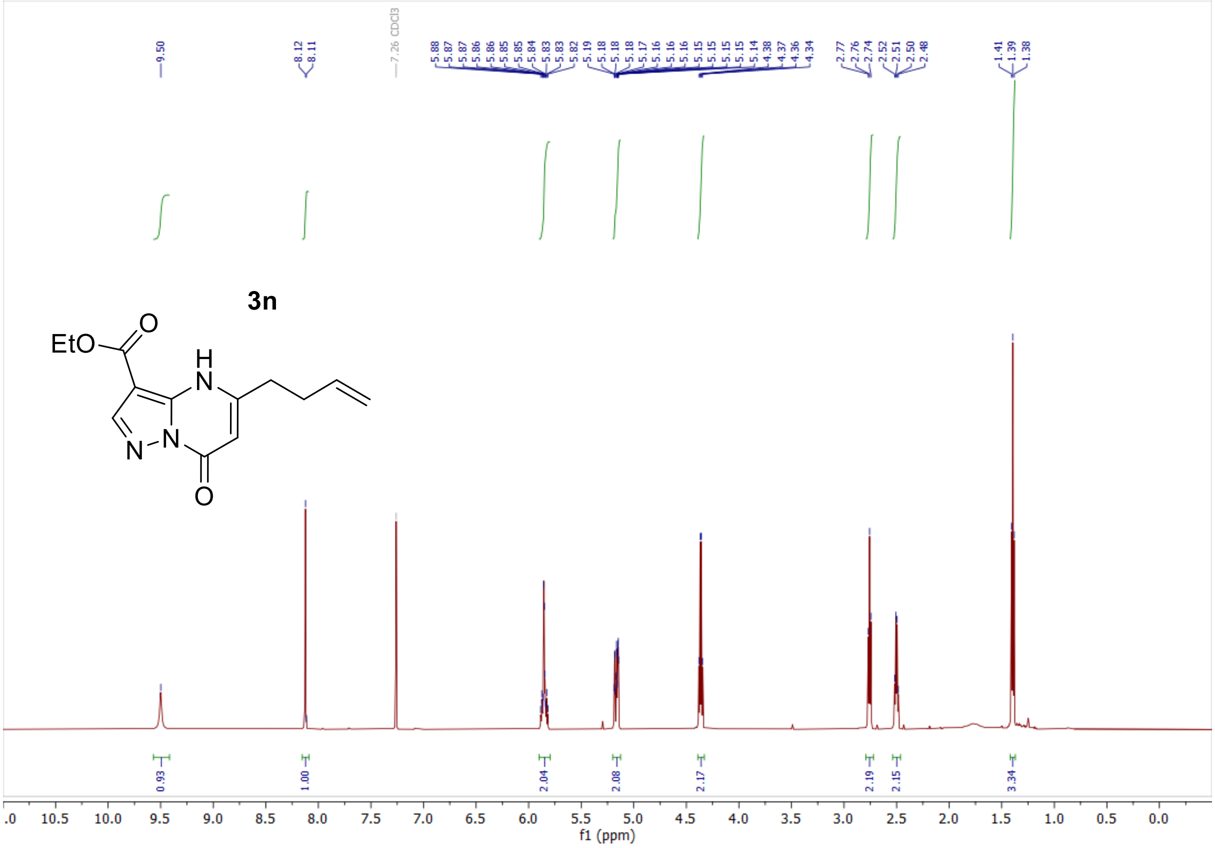

^{13}C NMR (151 MHz, $CDCl_3$) (3n)

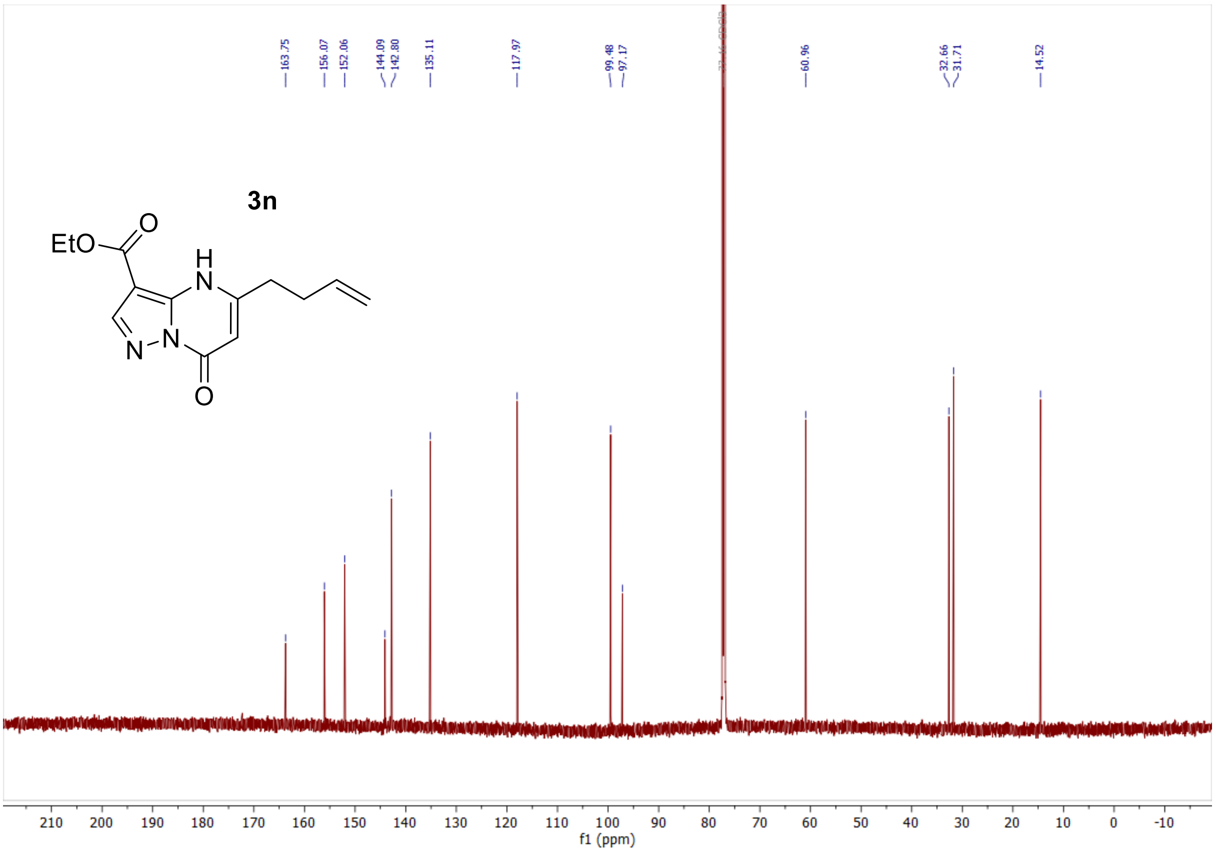

¹H NMR (600 MHz, $CDCl_3$) (3o)

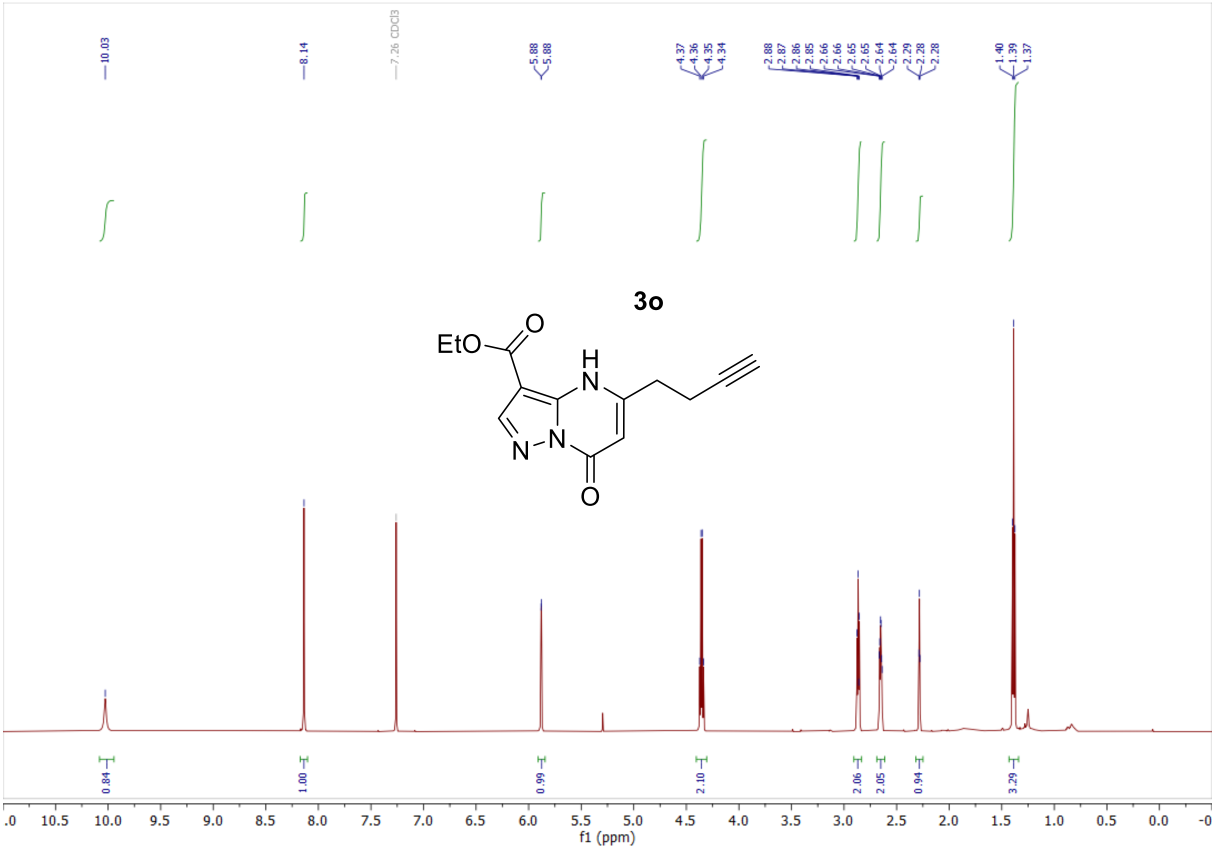

¹³C NMR (151 MHz, $CDCl_3$) (3o)

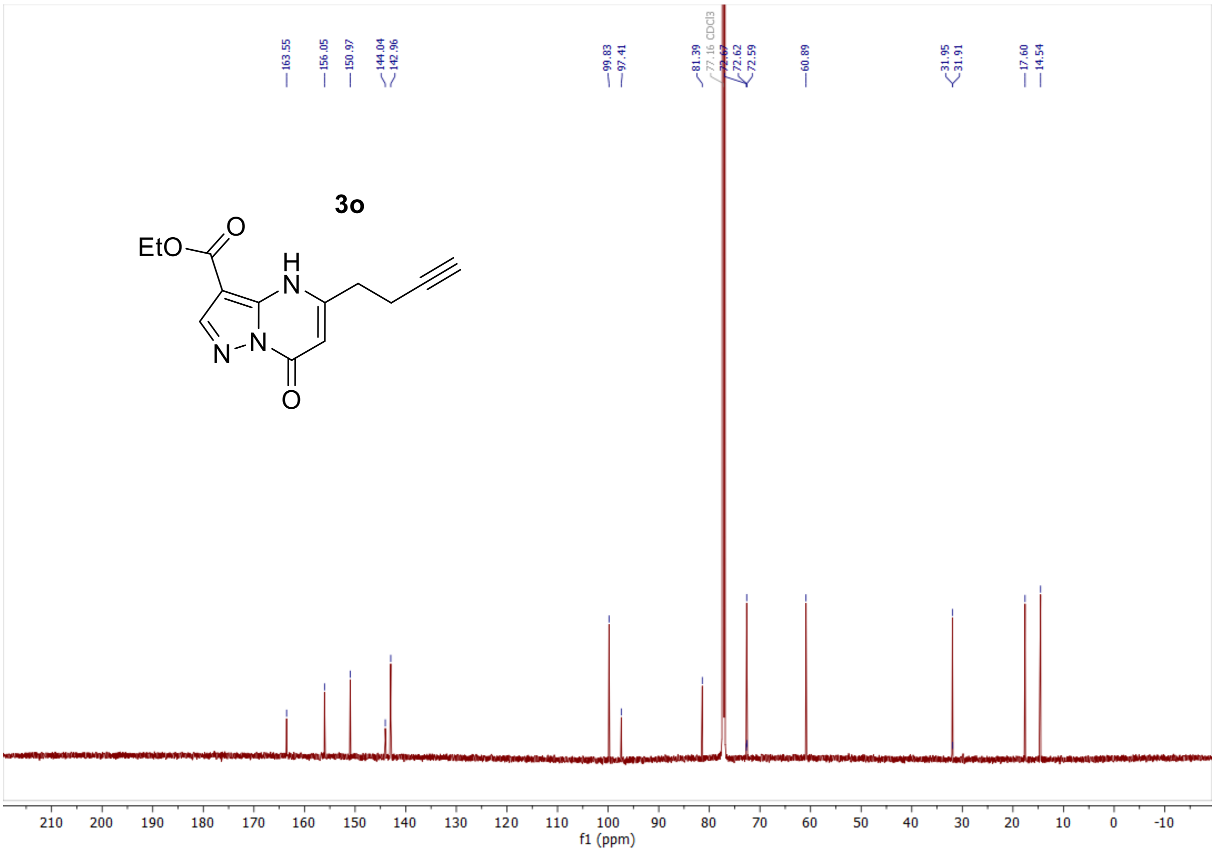

^{1}H NMR (400 MHz, $CDCl_3$) (3p)

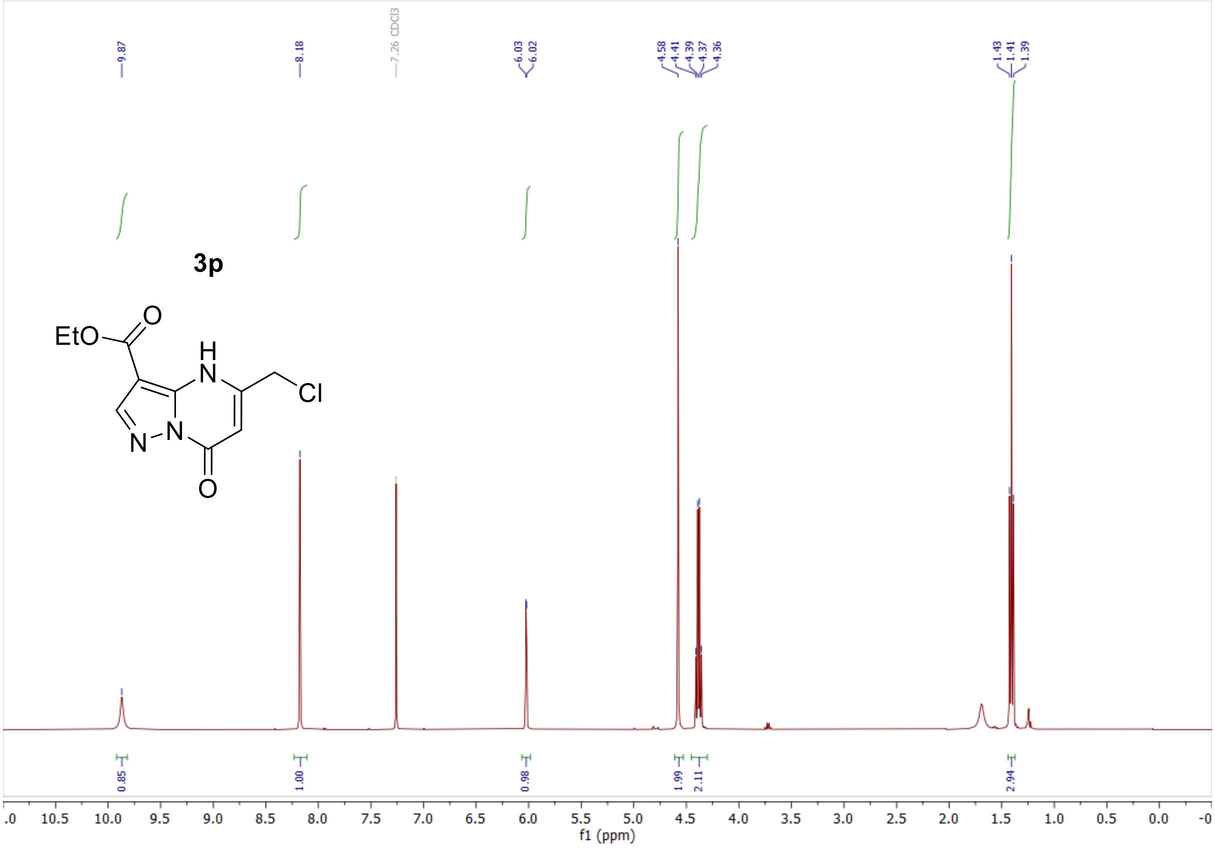

^{13}C NMR (100 MHz, $CDCl_3$) (3p)

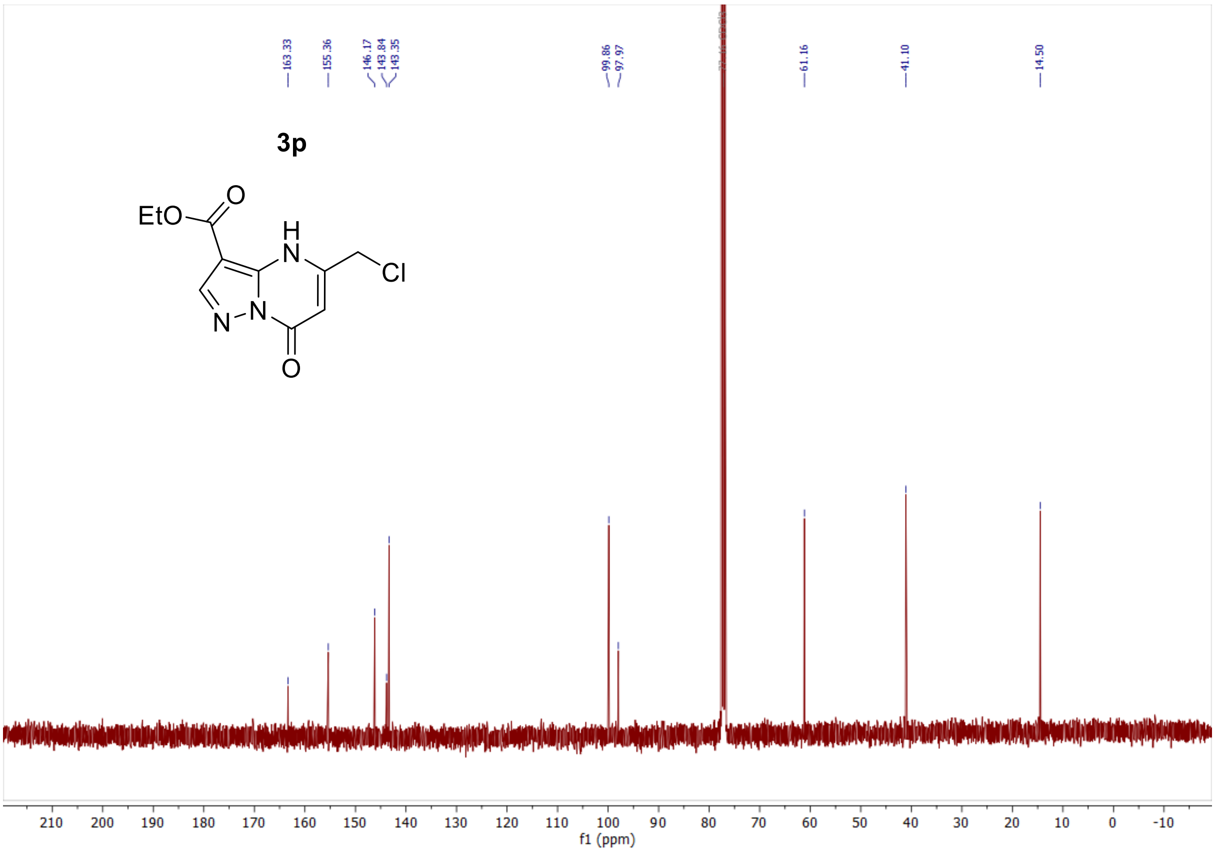

^{1}H NMR (600 MHz, $CDCl_3$) (3q)

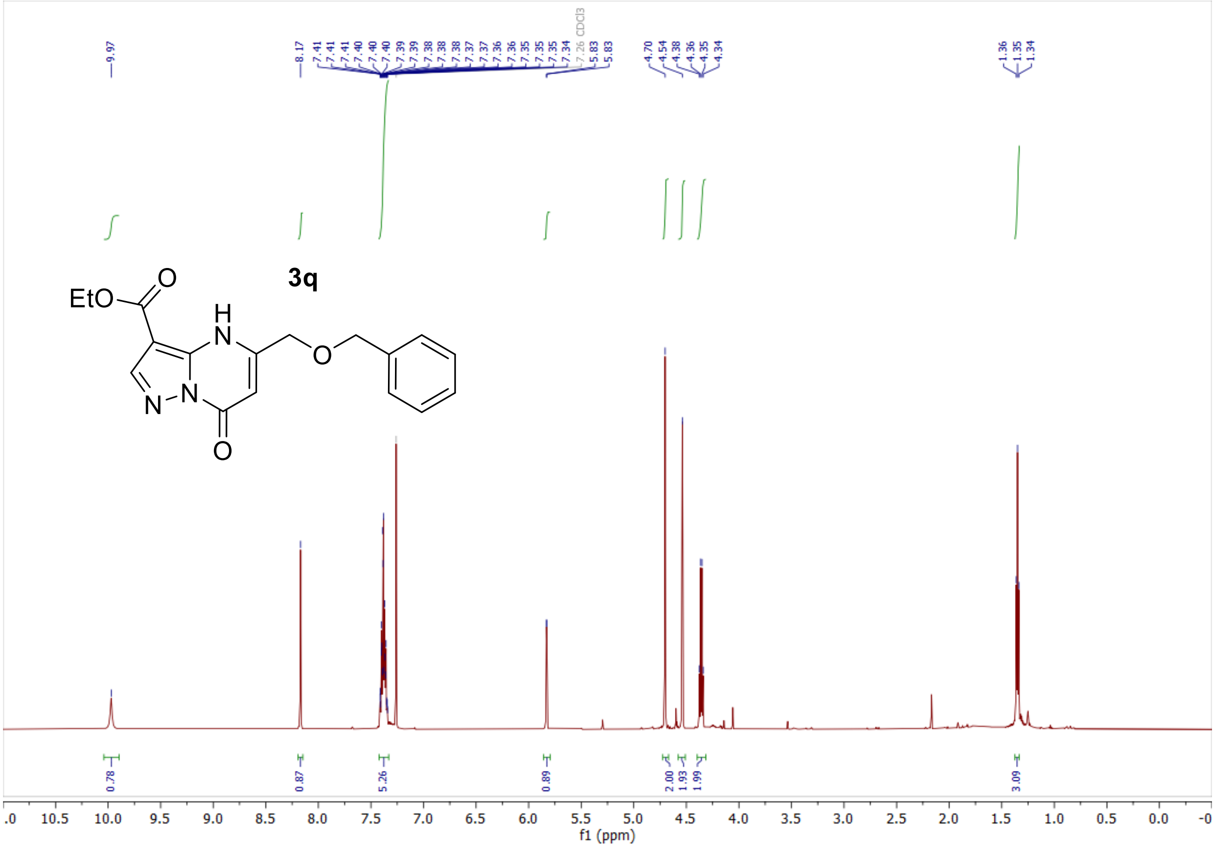

^{13}C NMR (151 MHz, $CDCl_3$) (3q)

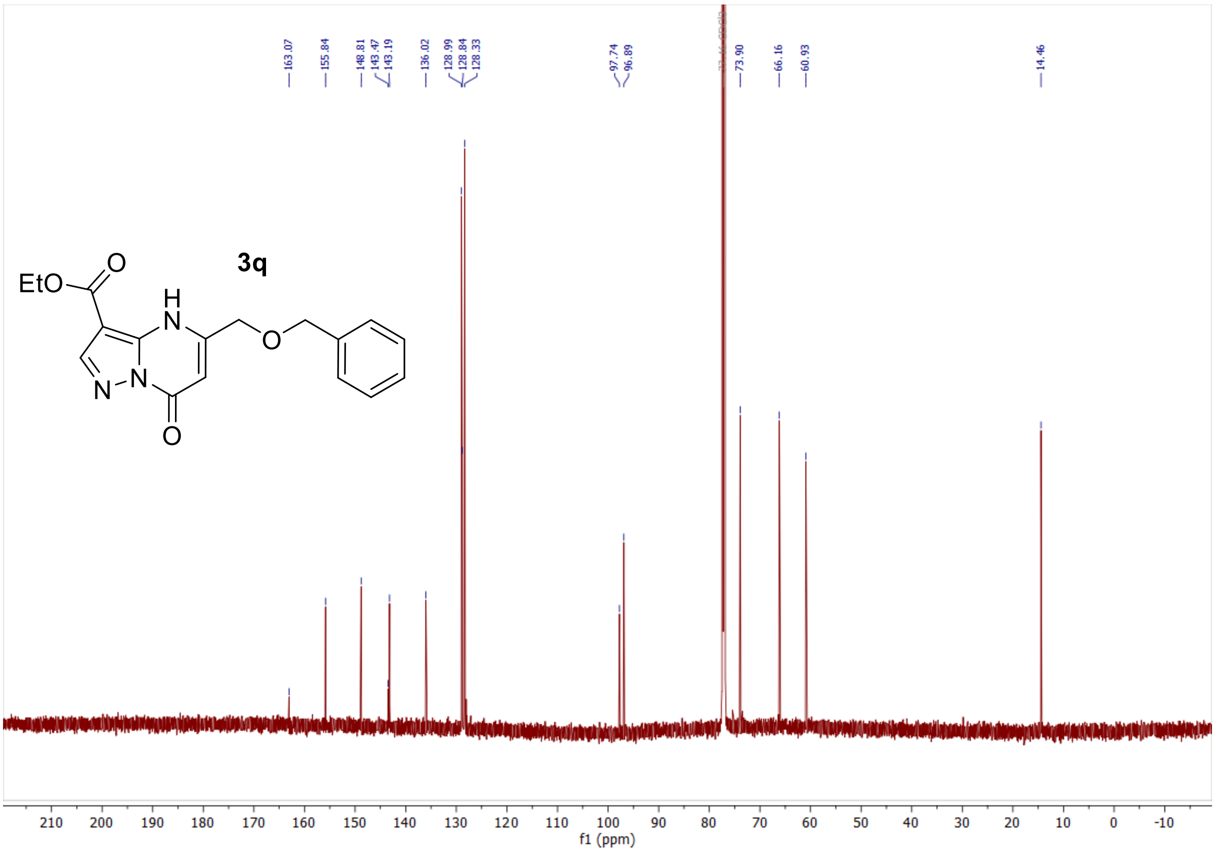

^{1}H NMR (400 MHz, MeOD) (3r)

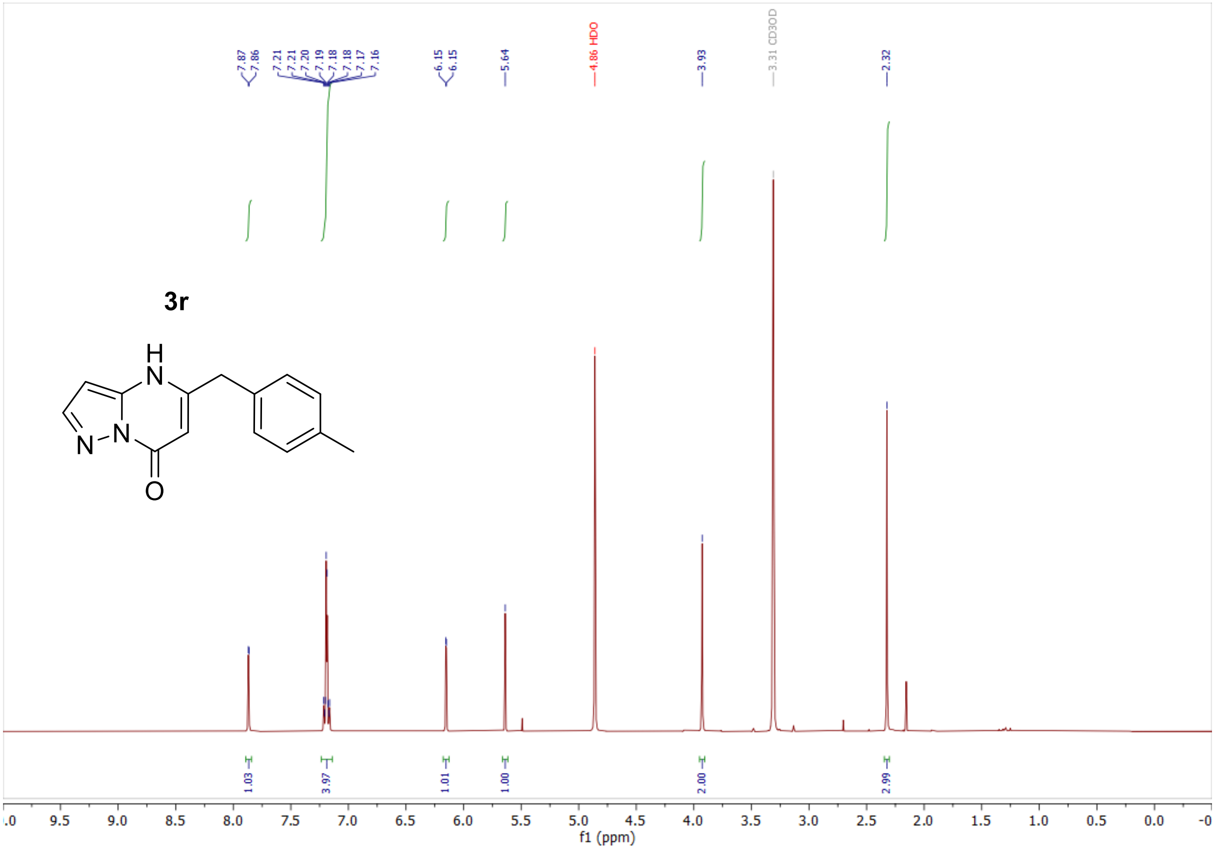

^{13}C NMR (100 MHz, MeOD) (3r)

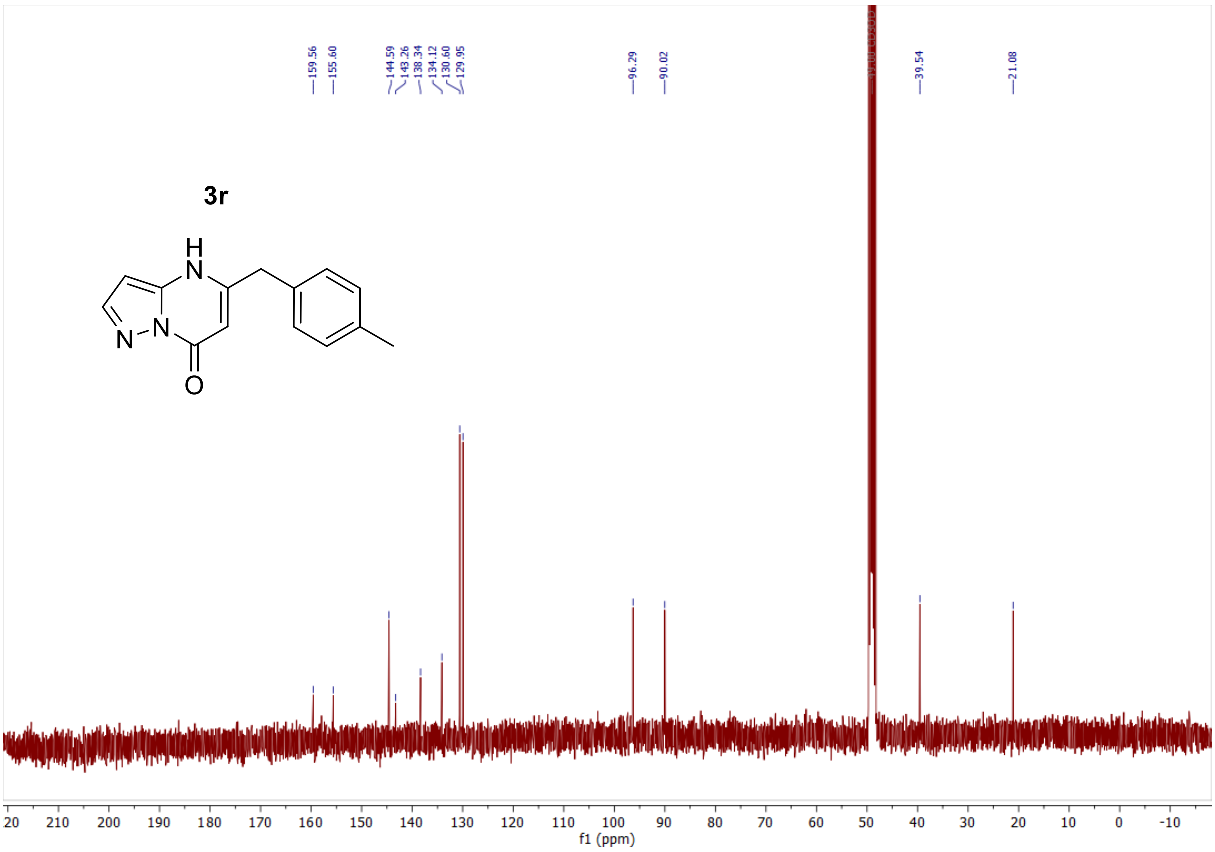

^{1}H NMR (400 MHz, $CDCl_3$) (3t)

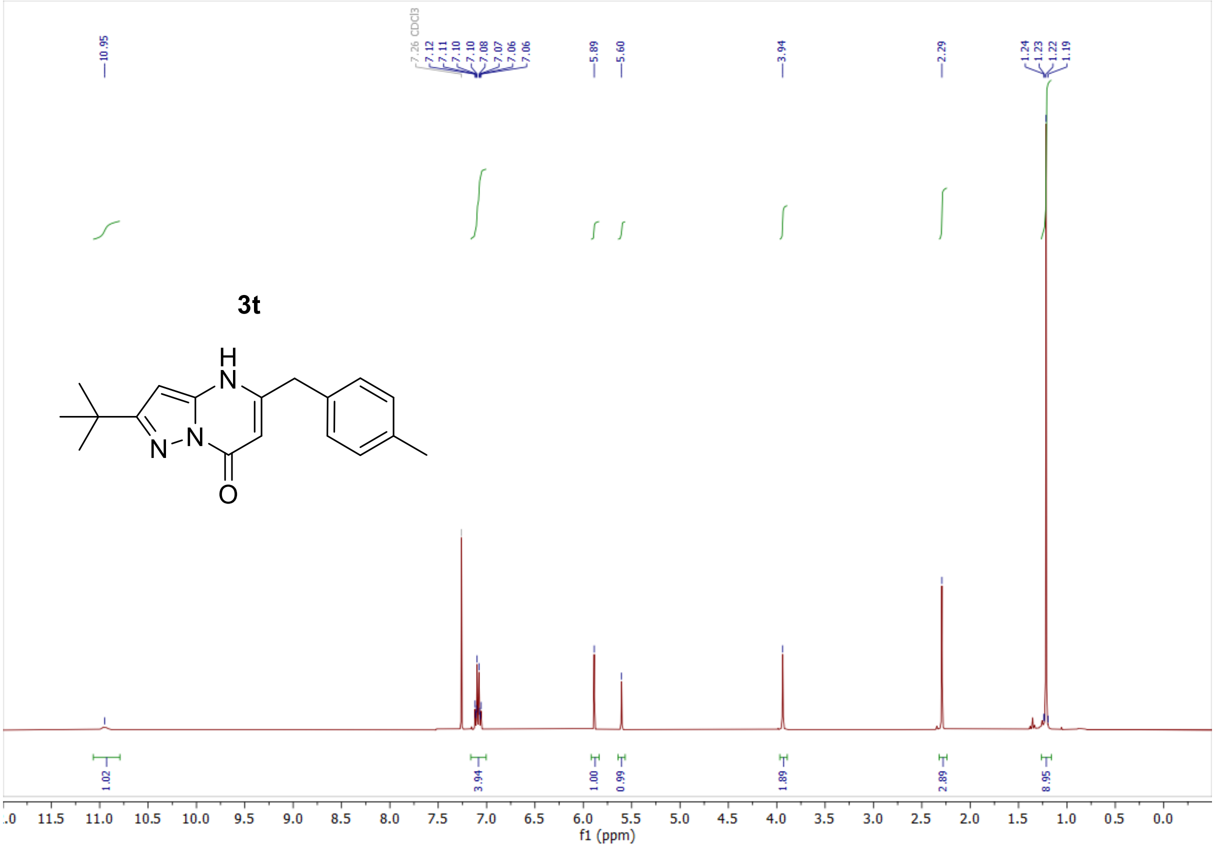

^{13}C NMR (100 MHz, $CDCl_3$) (3t)

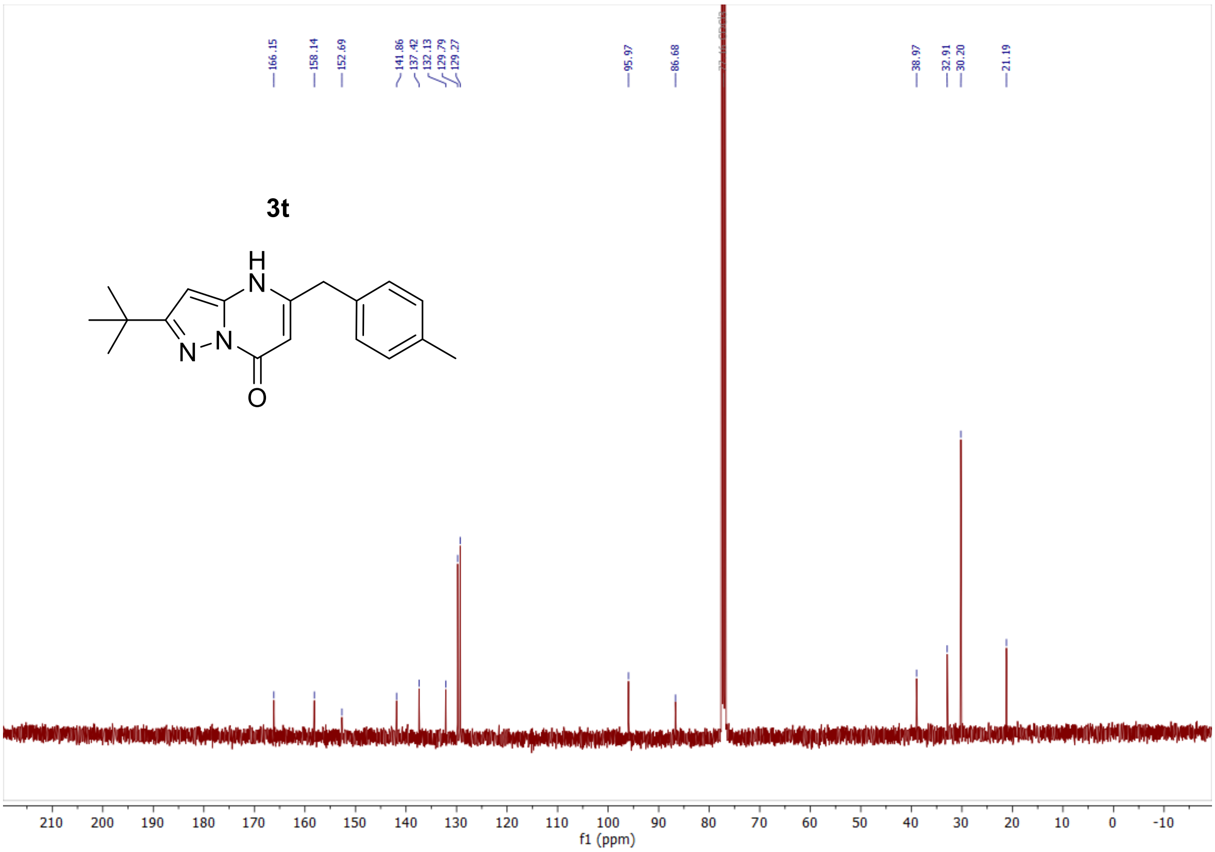

^{1}H NMR (400 MHz, DMSO) (3u)

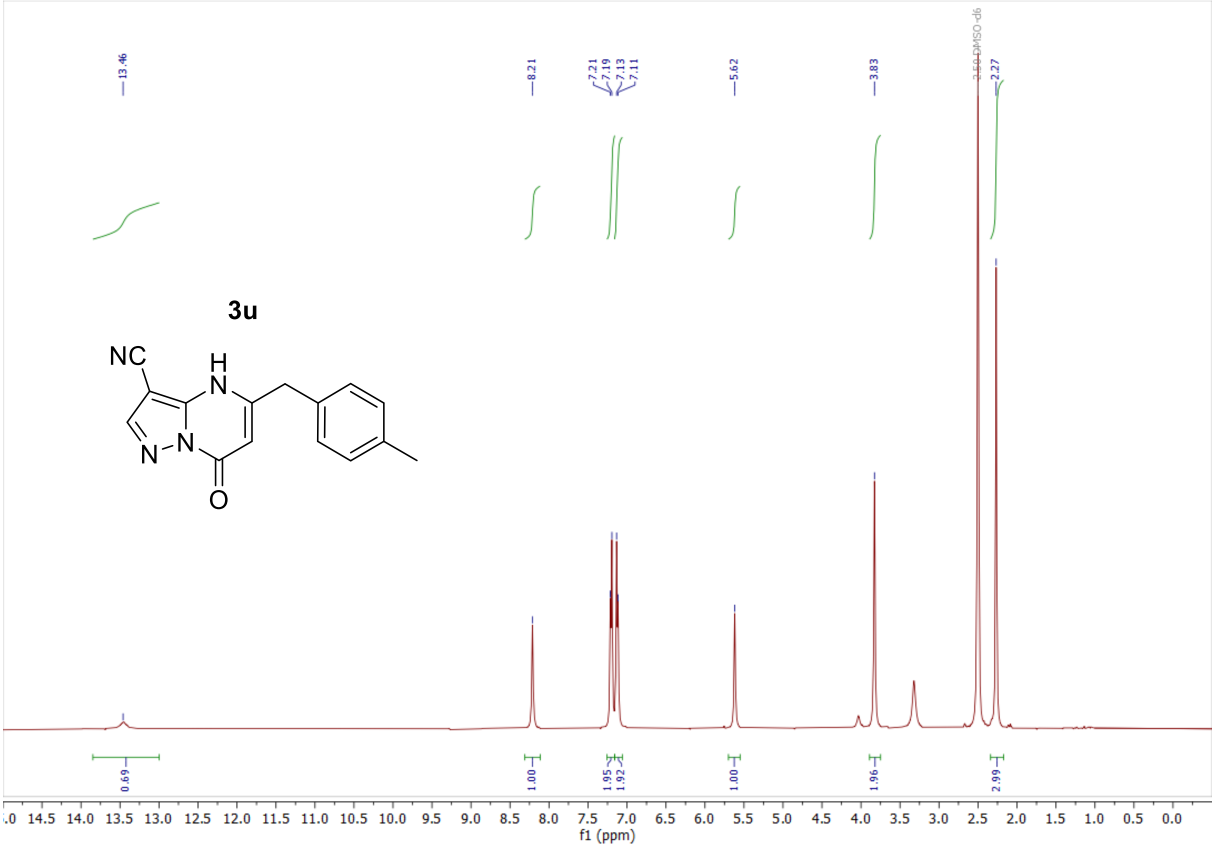

^{13}C NMR (100 MHz, DMSO) (3u)

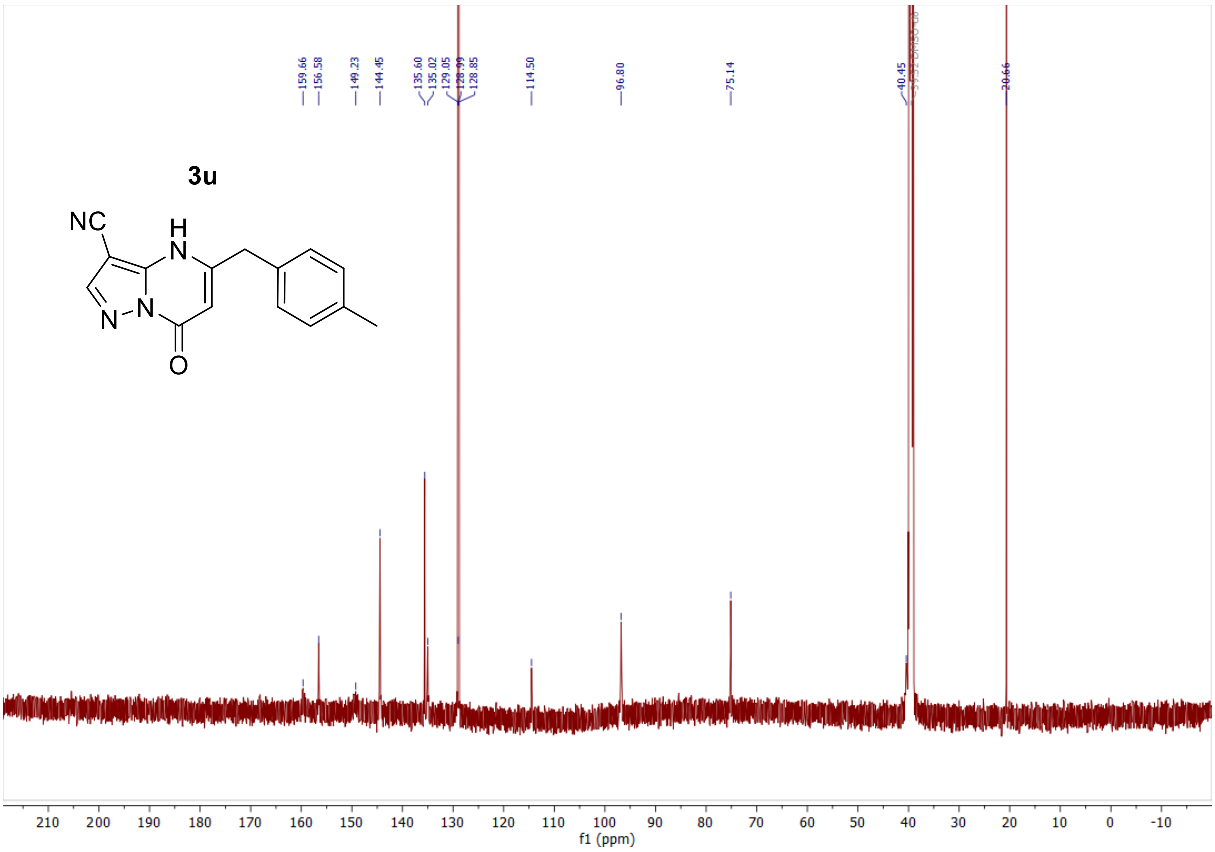

^{1}H NMR (600 MHz, DMSO) (3v)

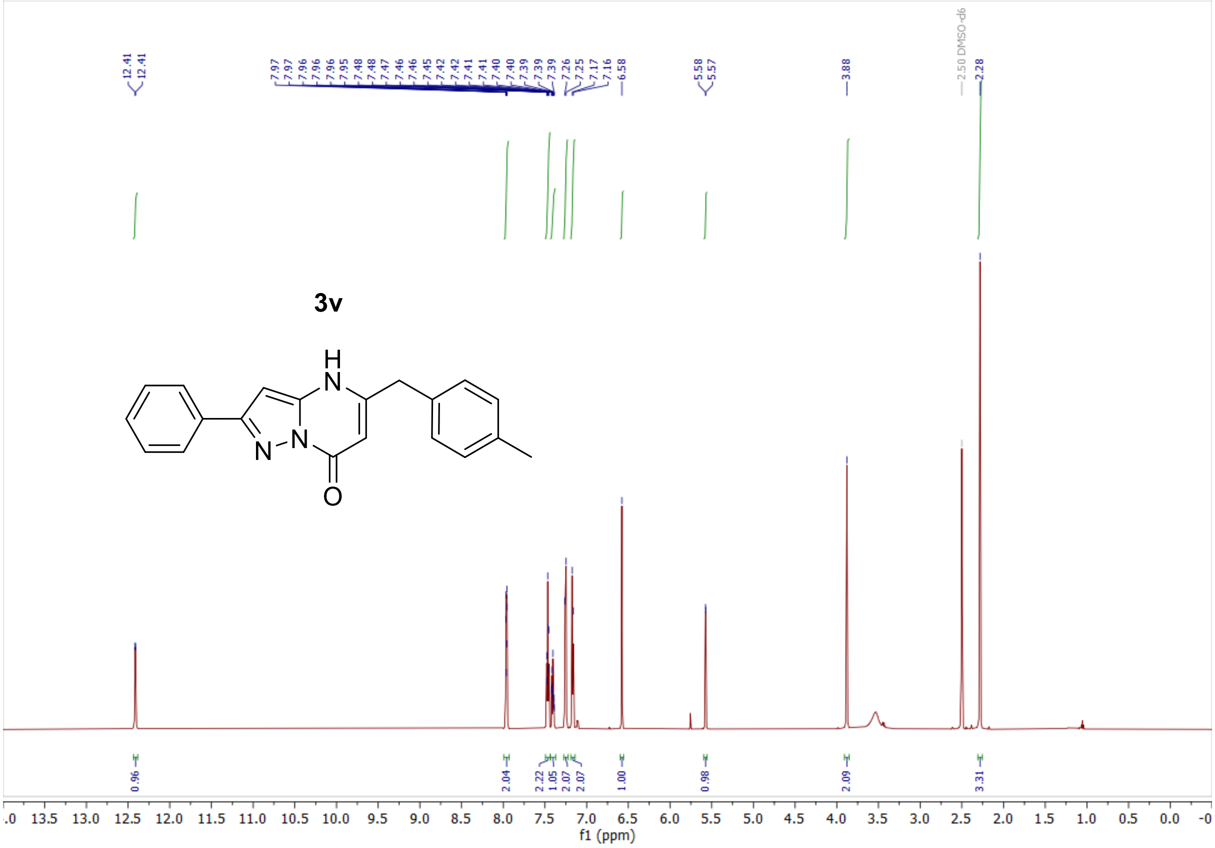

^{13}C NMR (151 MHz, DMSO) (3v)

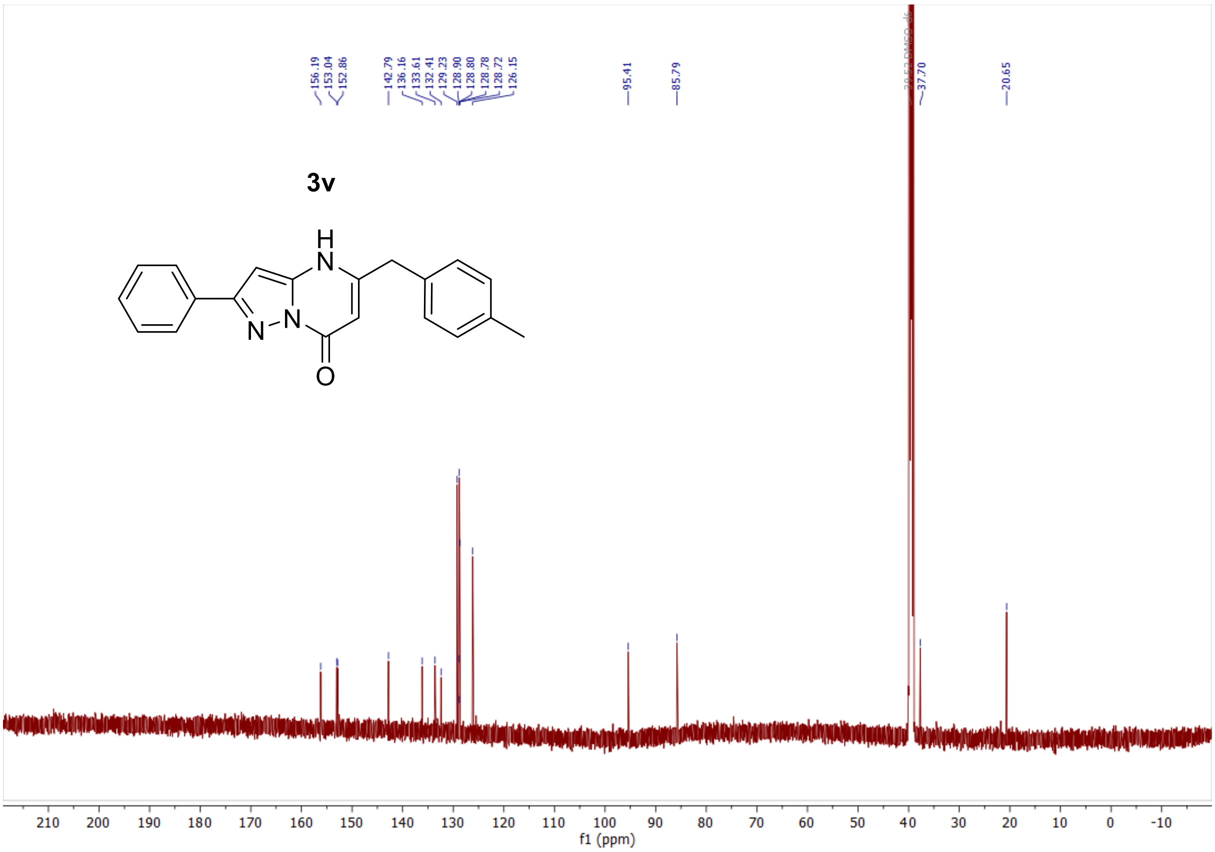

^{1}H NMR (600 MHz, MeOD) (3w)

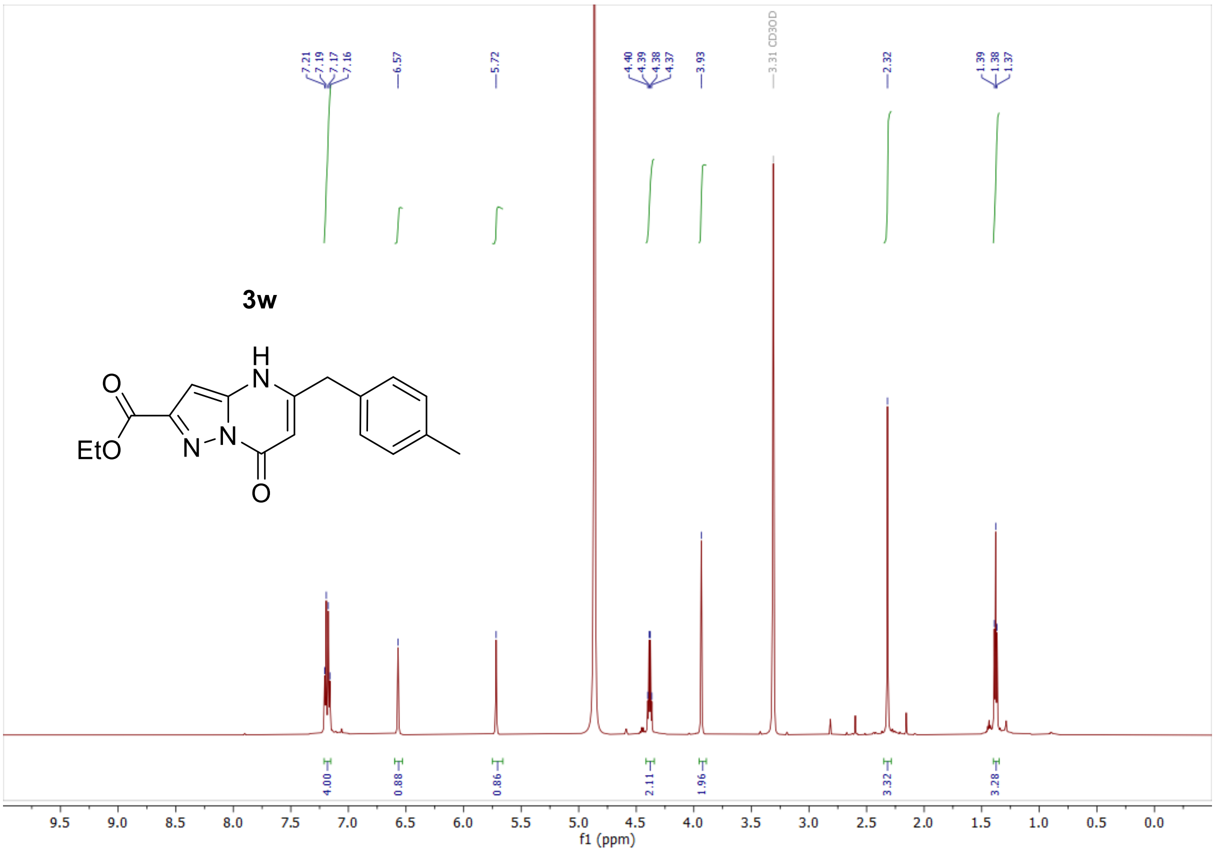

^{13}C NMR (151 MHz, MeOD) (3w)

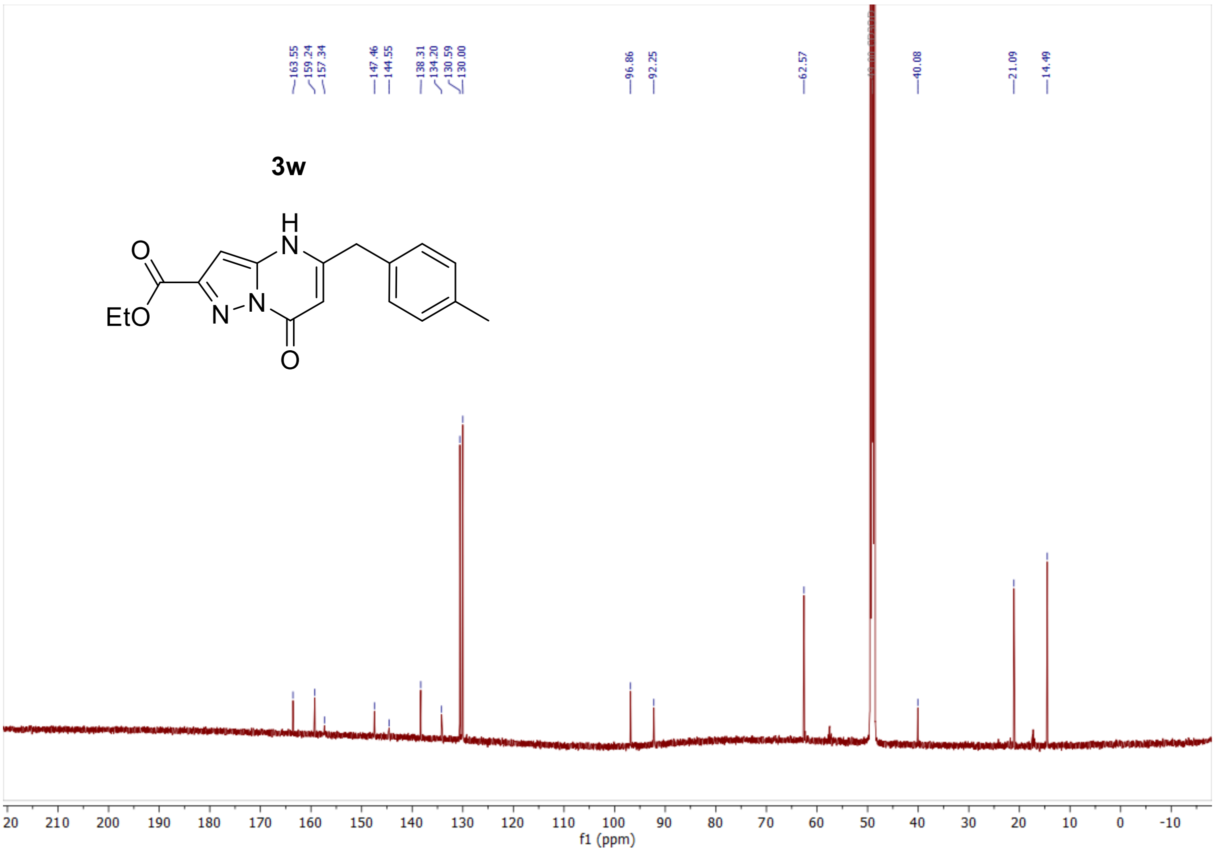

^{1}H NMR (400 MHz, CDCl₃) (5a)

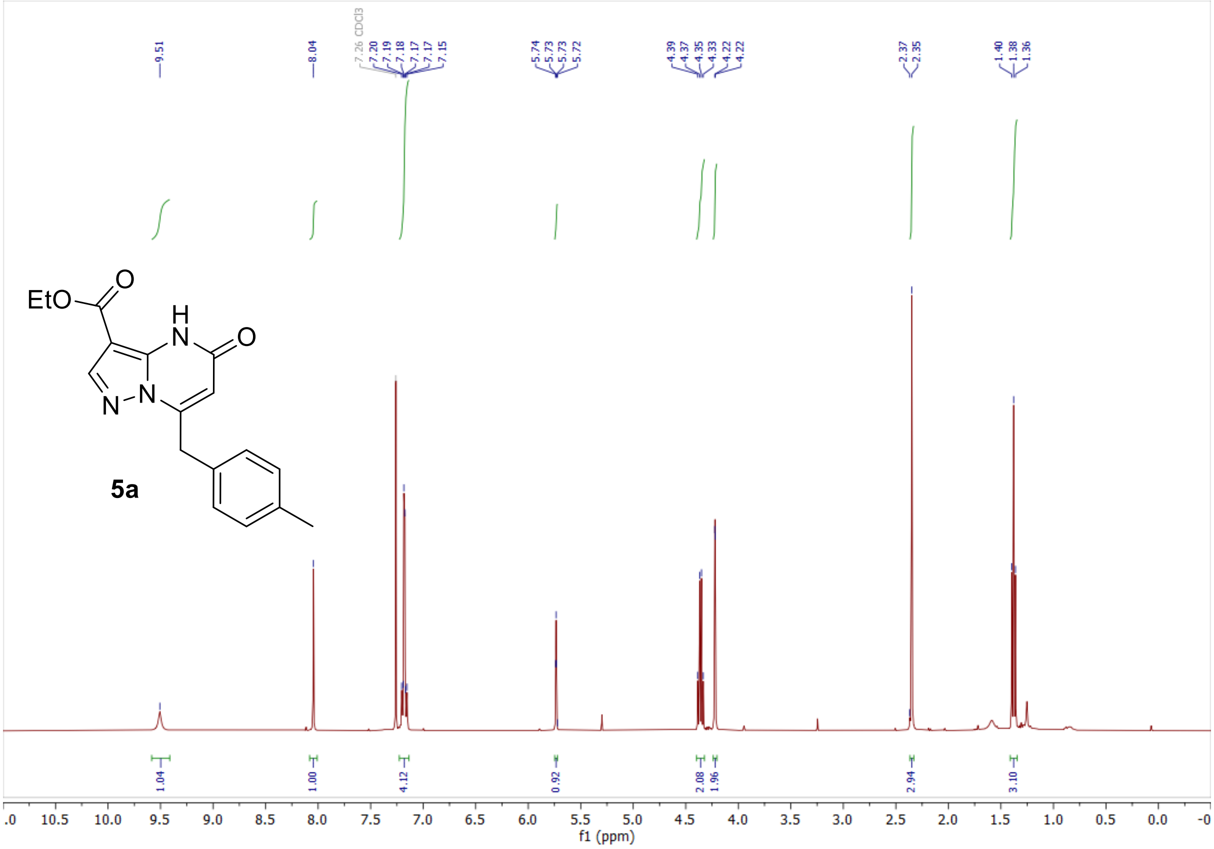

^{13}C NMR (100 MHz, CDCl₃) (5a)

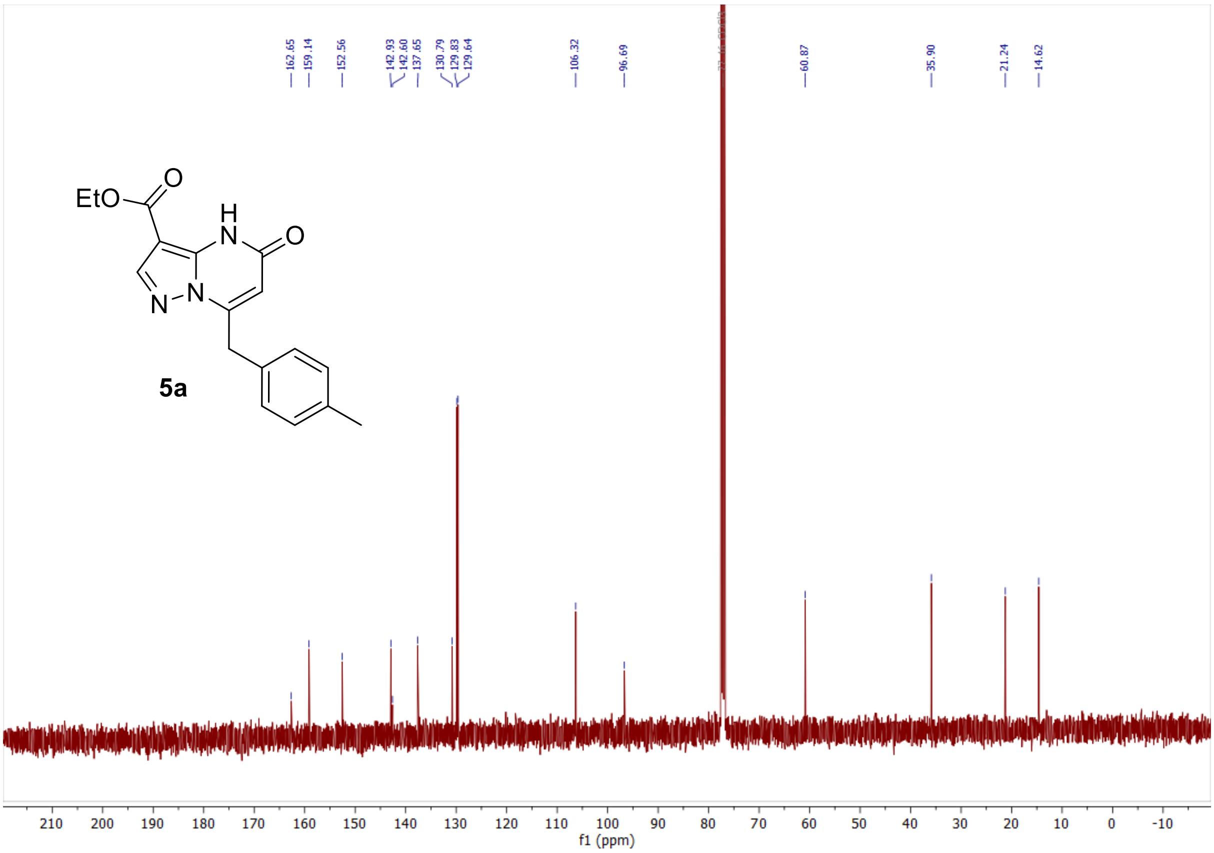

^{1}H NMR (400 MHz, $CDCl_3$) (5b)

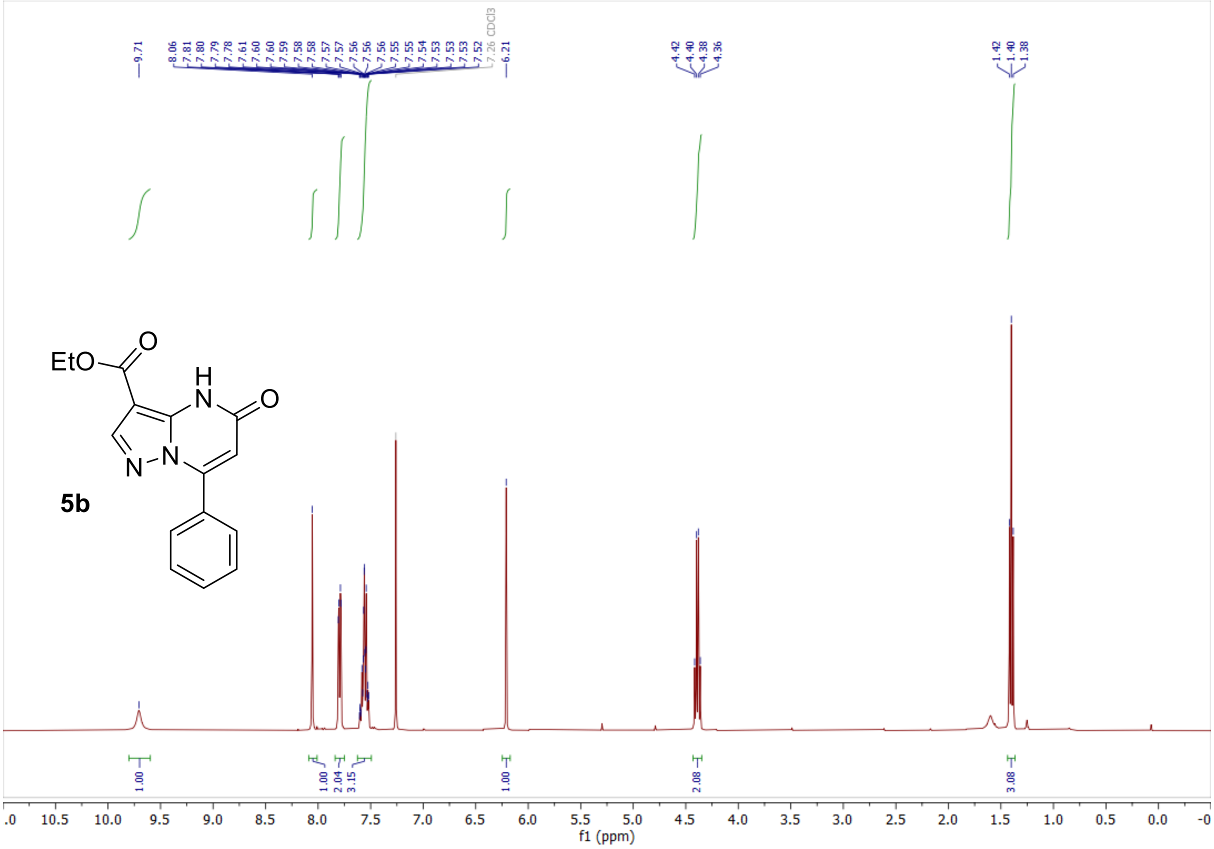

^{13}C NMR (100 MHz, $CDCl_3$) (5b)

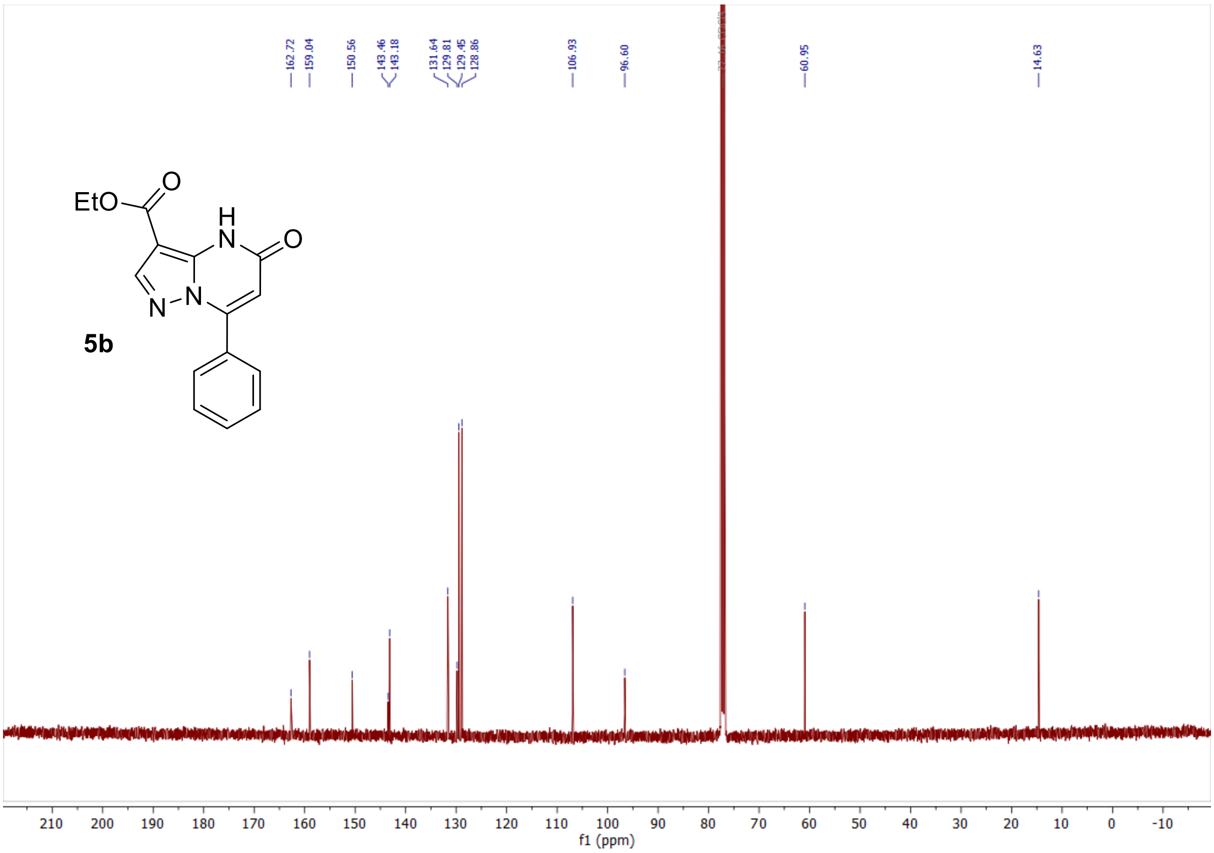

^{1}H NMR (600 MHz, $CDCl_3$) (5c)

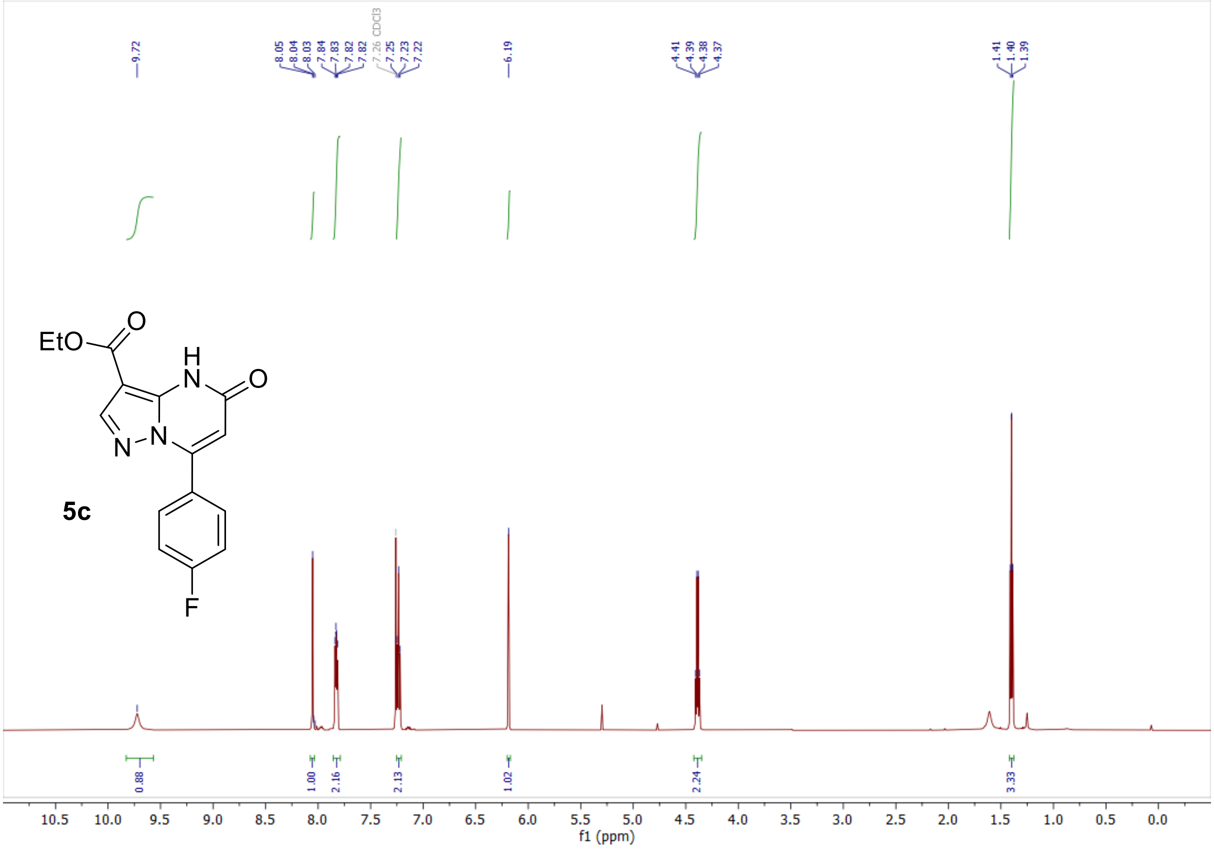

^{13}C NMR (151 MHz, $CDCl_3$) (5c)

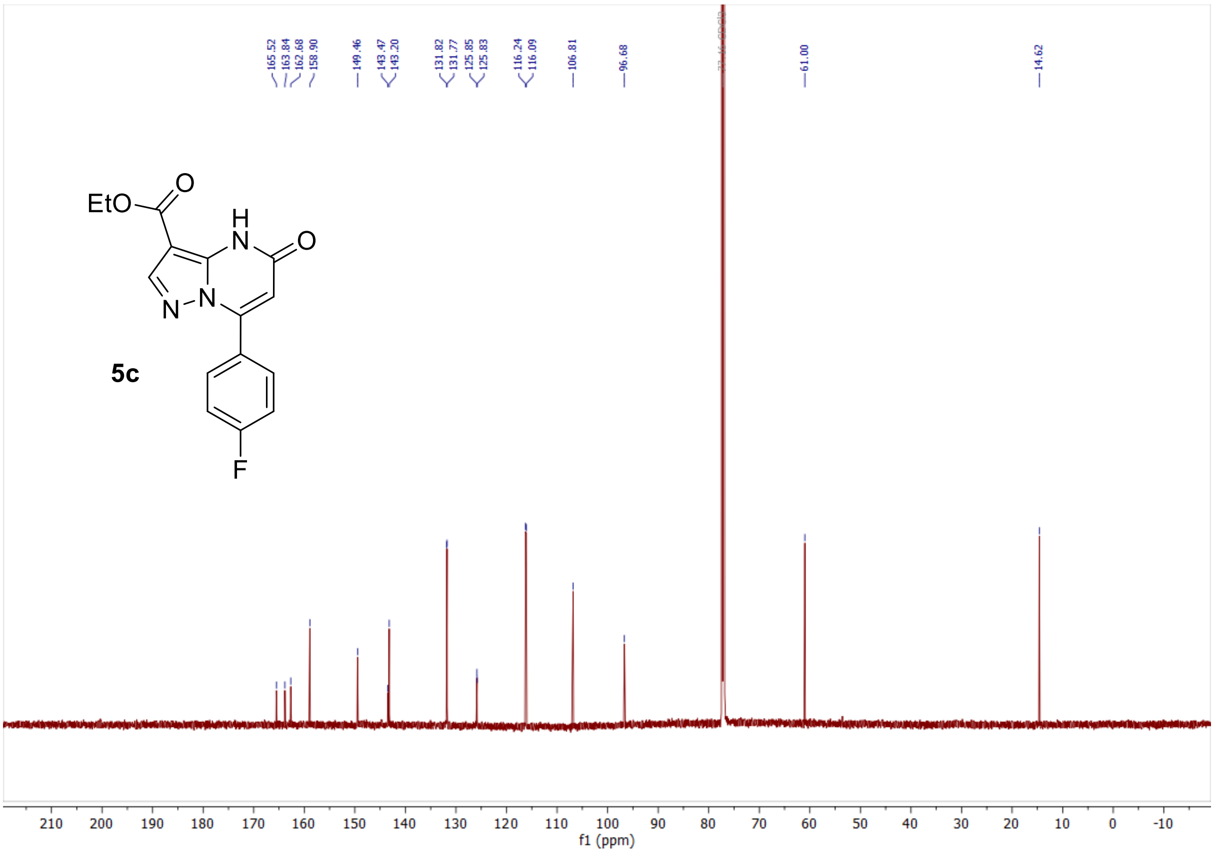

^{19}F NMR (565 MHz, $CDCl_3$) (5c)

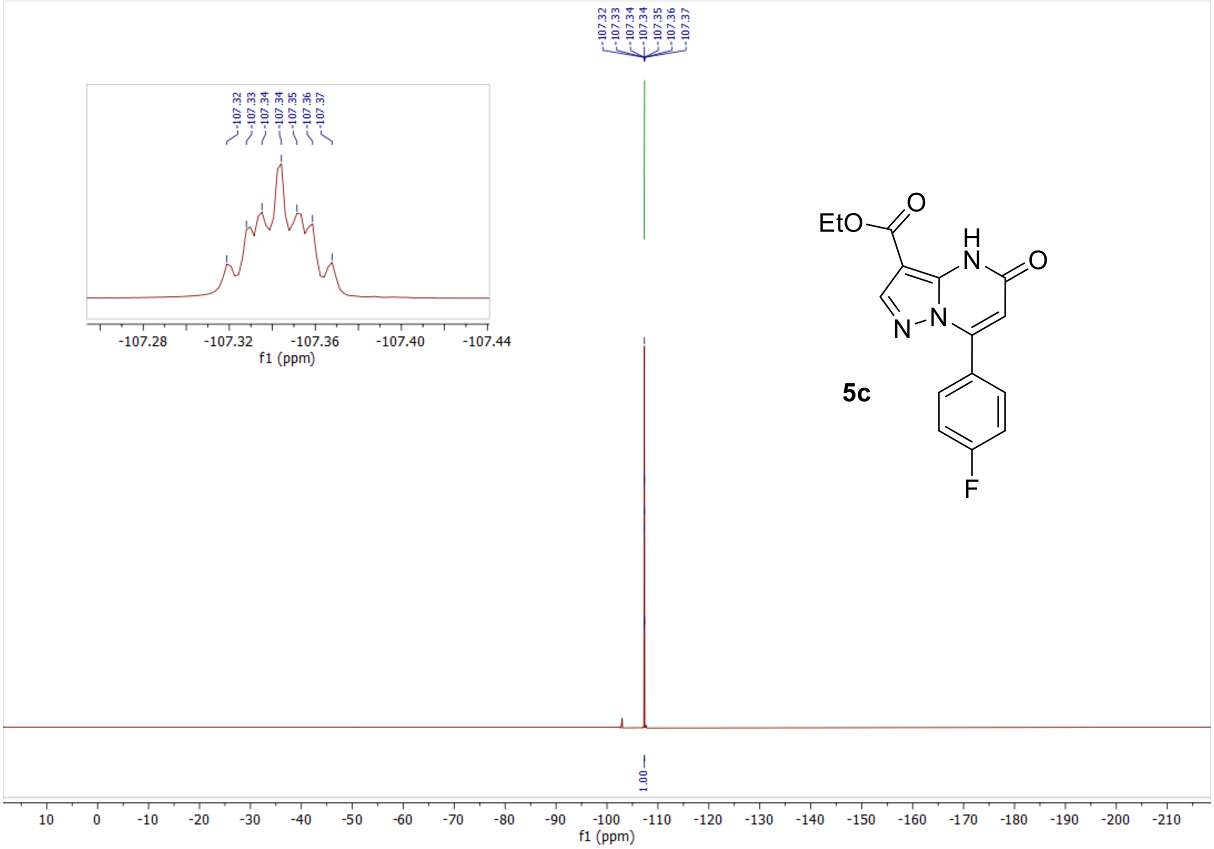

^{1}H NMR (600 MHz, $CDCl_3$) (5d)

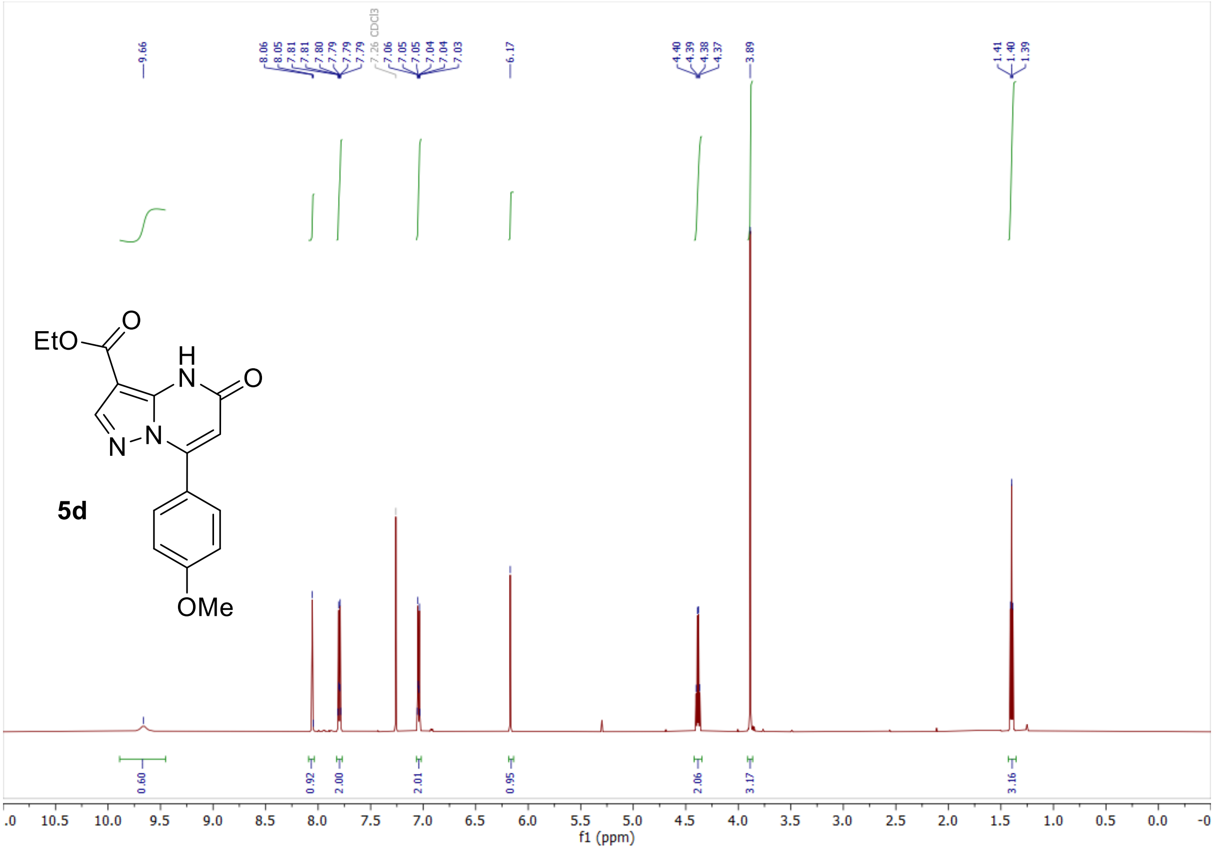

^{13}C NMR (151 MHz, CDCl₃) (5d)

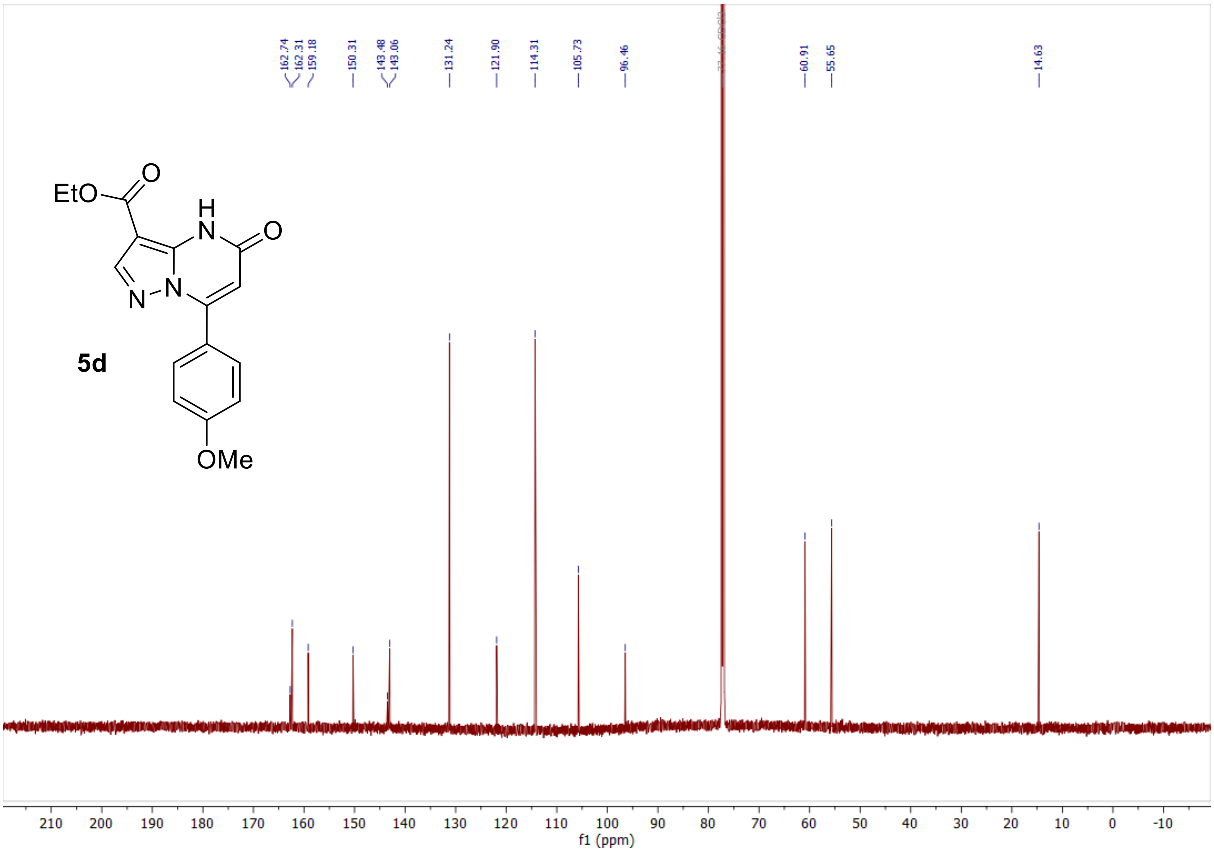

^{1}H NMR (400 MHz, CDCl₃) (5e)

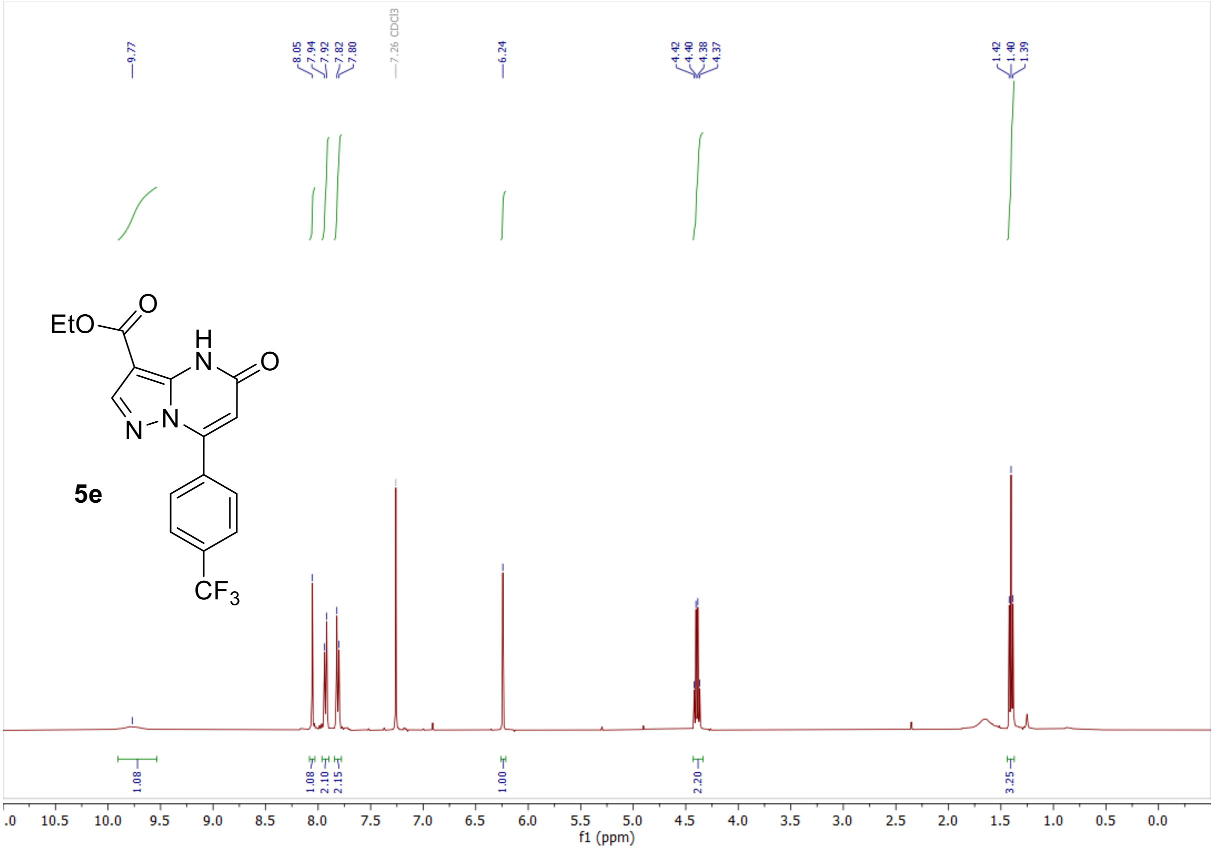

^{13}C NMR (100 MHz, $CDCl_3$) (5e)

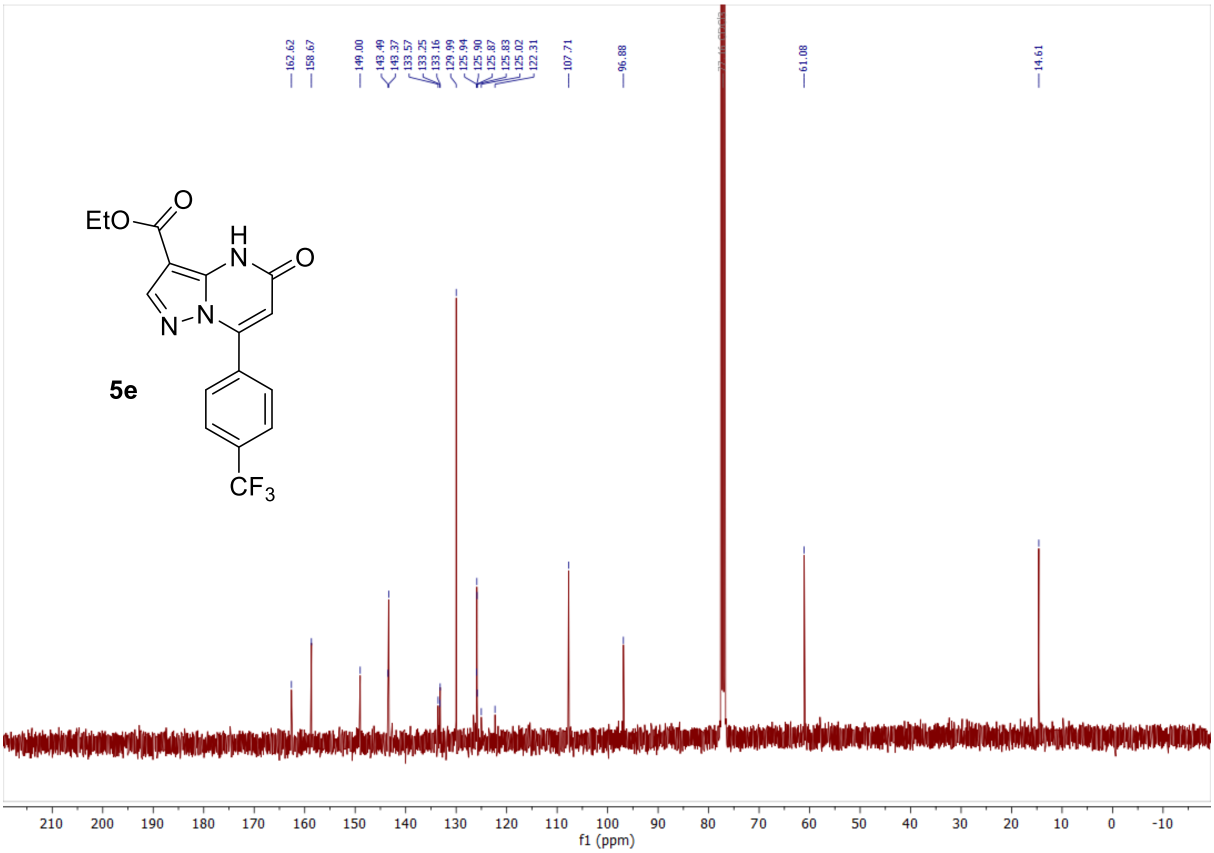

^{19}F NMR (376 MHz, $CDCl_3$) (5e)

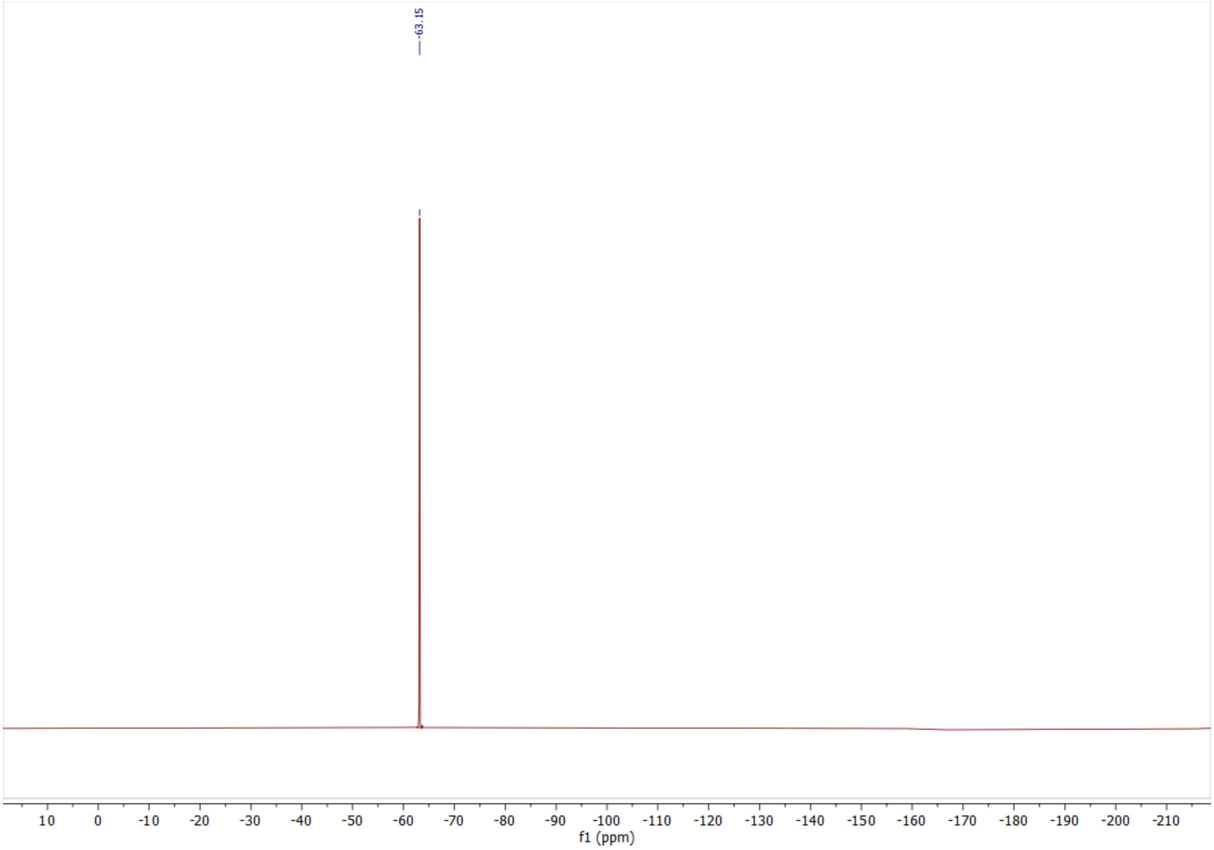

^{1}H NMR (400 MHz, CDCl₃) (5f)

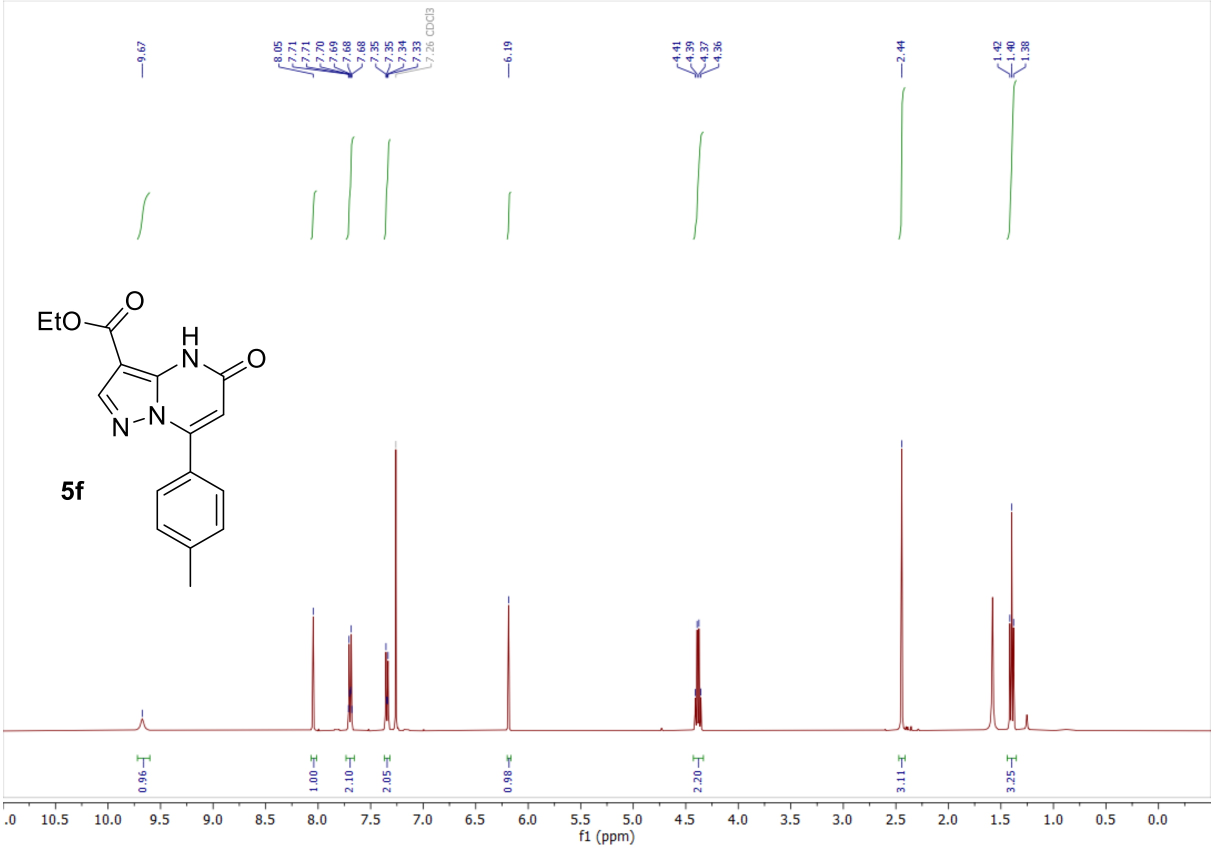

^{13}C NMR (100 MHz, CDCl₃) (5f)

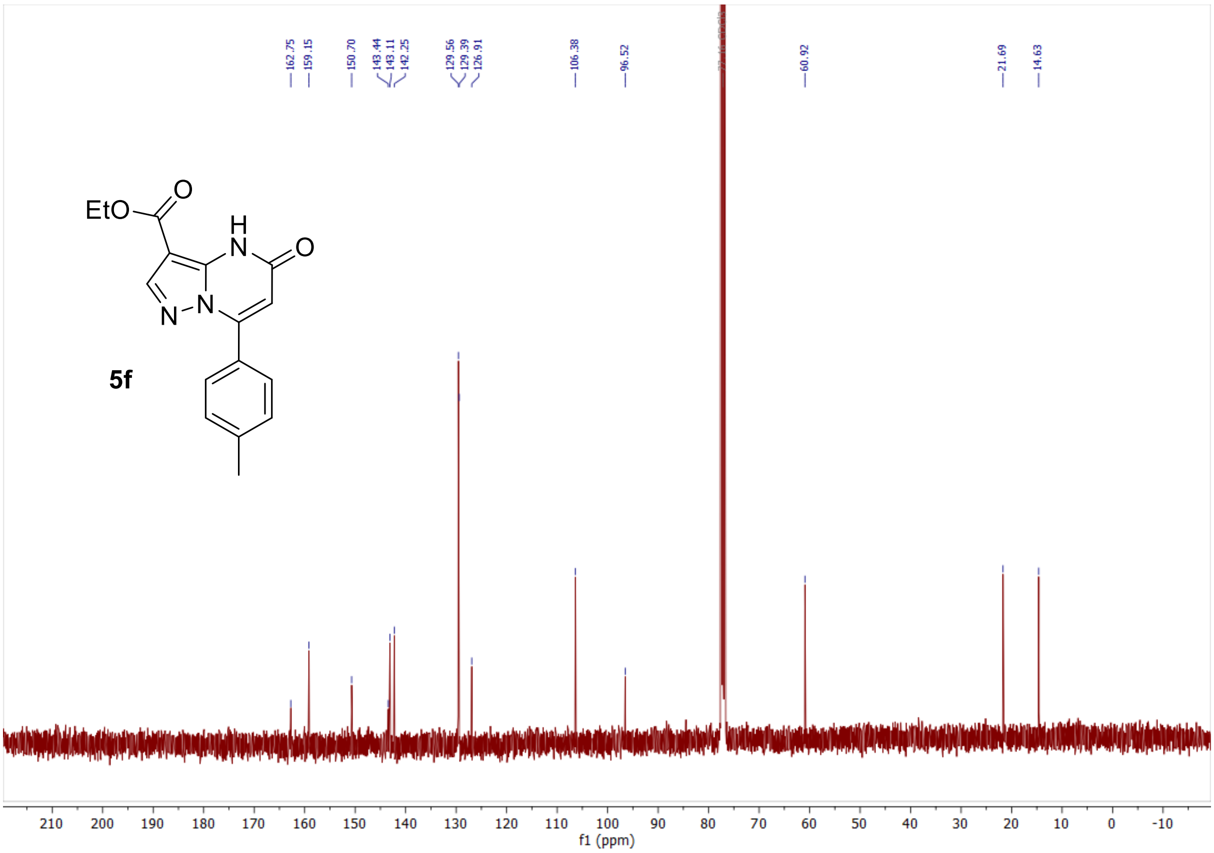

^{1}H NMR (400 MHz, $CDCl_3$) (5h)

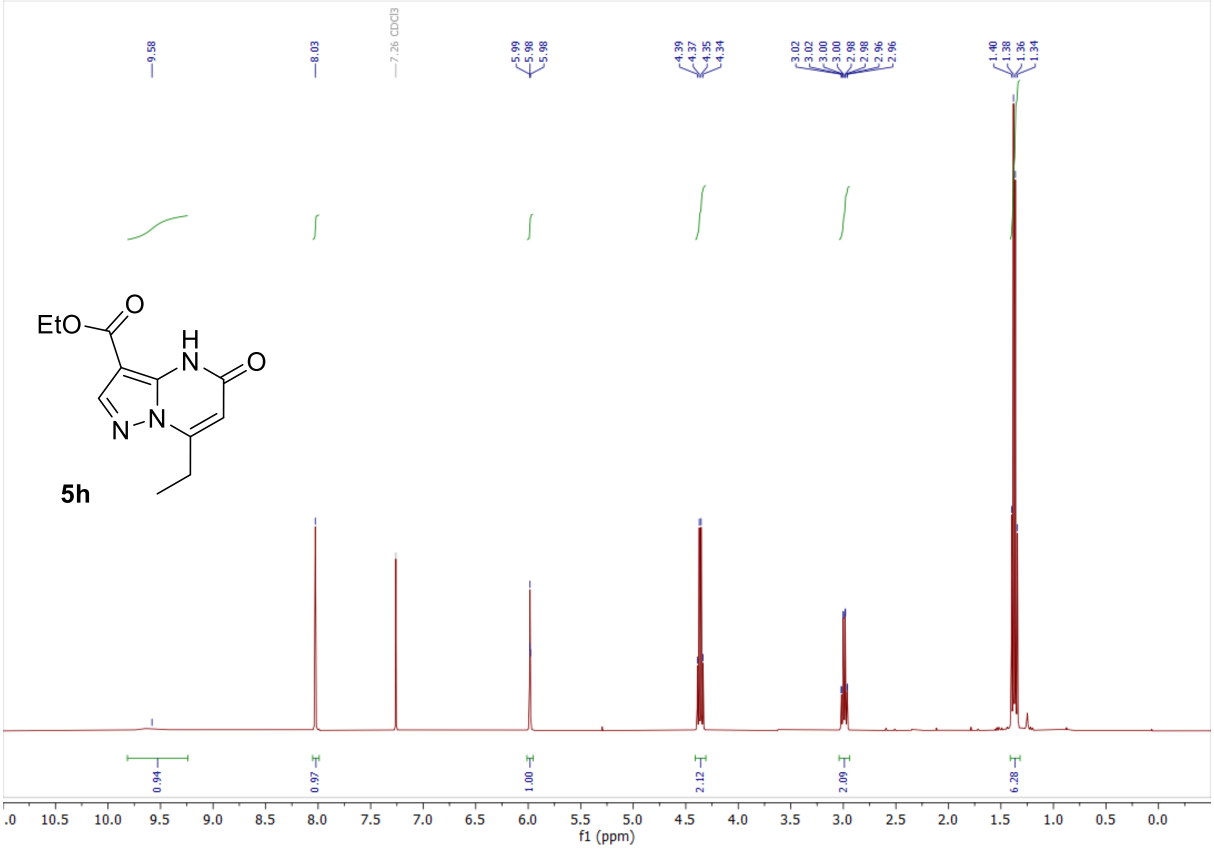

^{13}C NMR (100 MHz, $CDCl_3$) (5h)

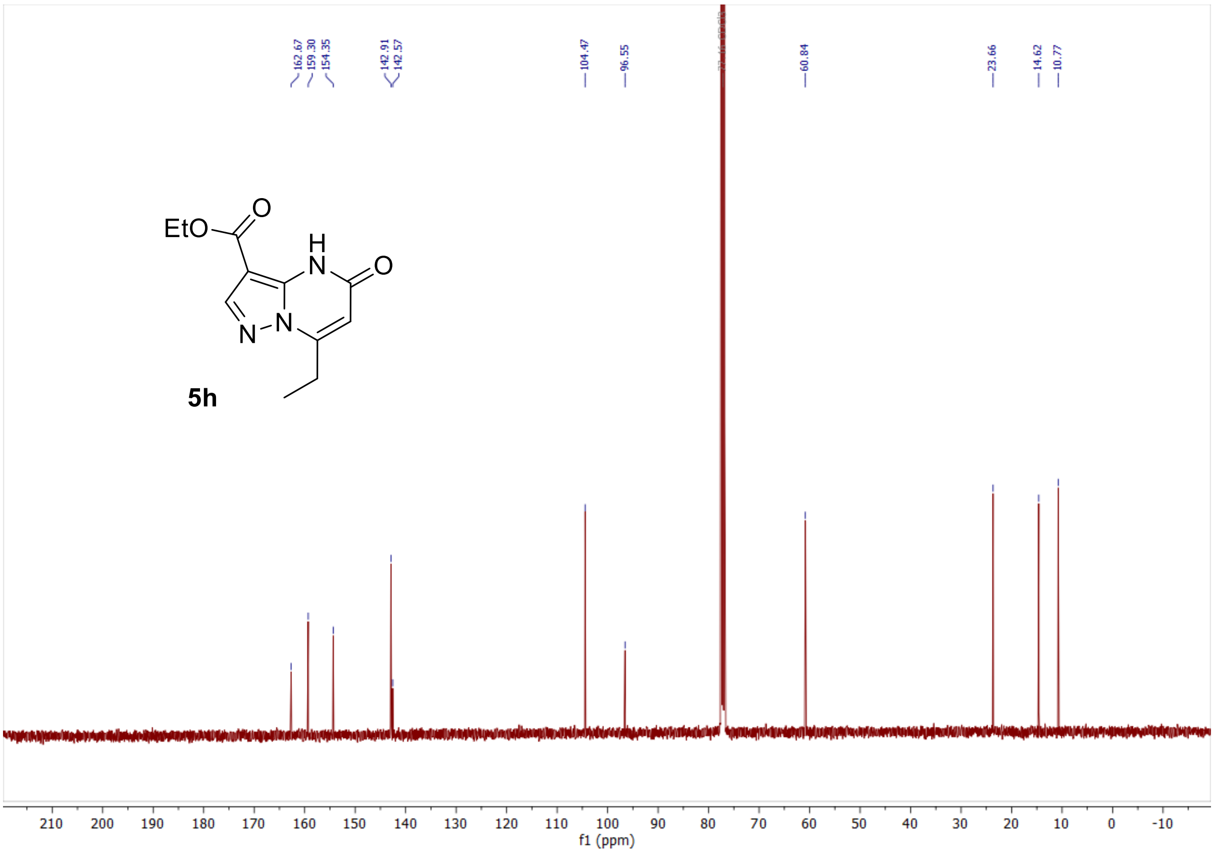

^{1}H NMR (600 MHz, CDCl₃) (5i)

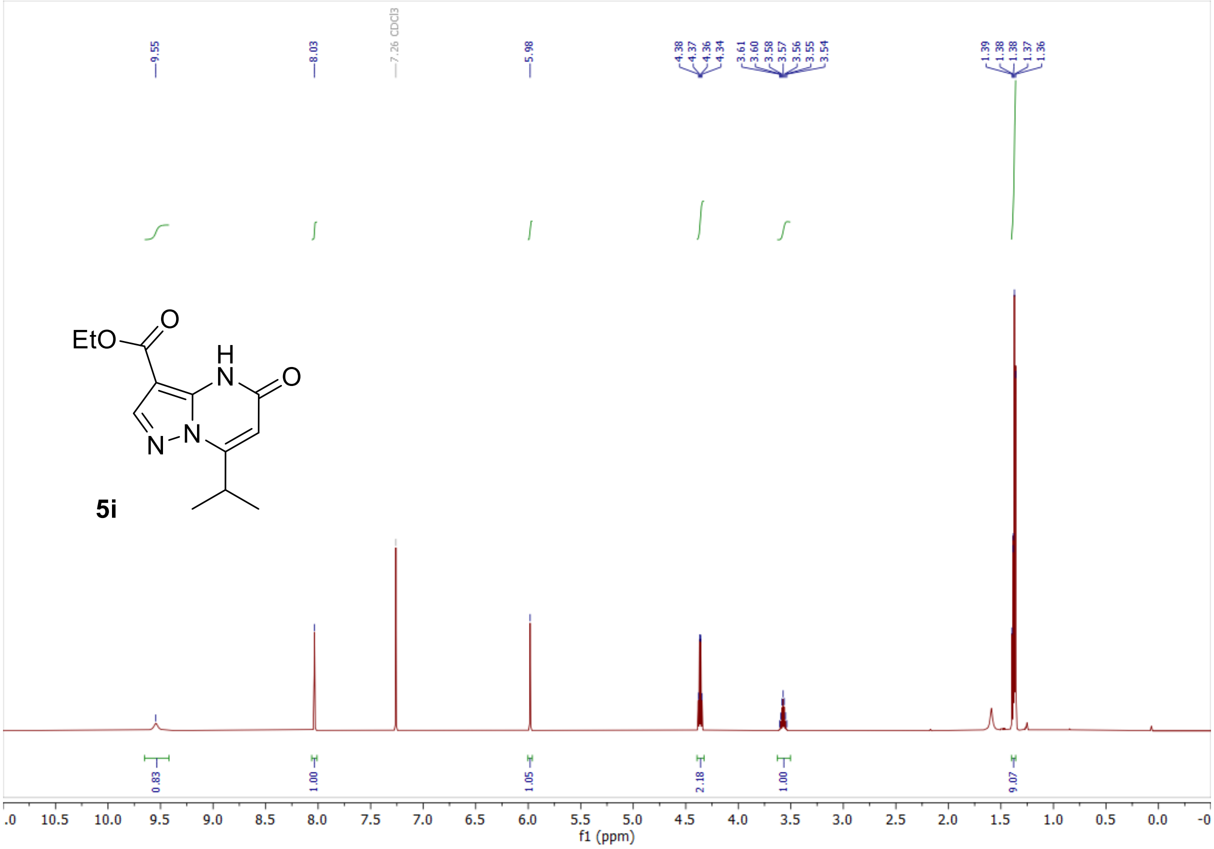

^{13}C NMR (151 MHz, CDCl₃) (5i)

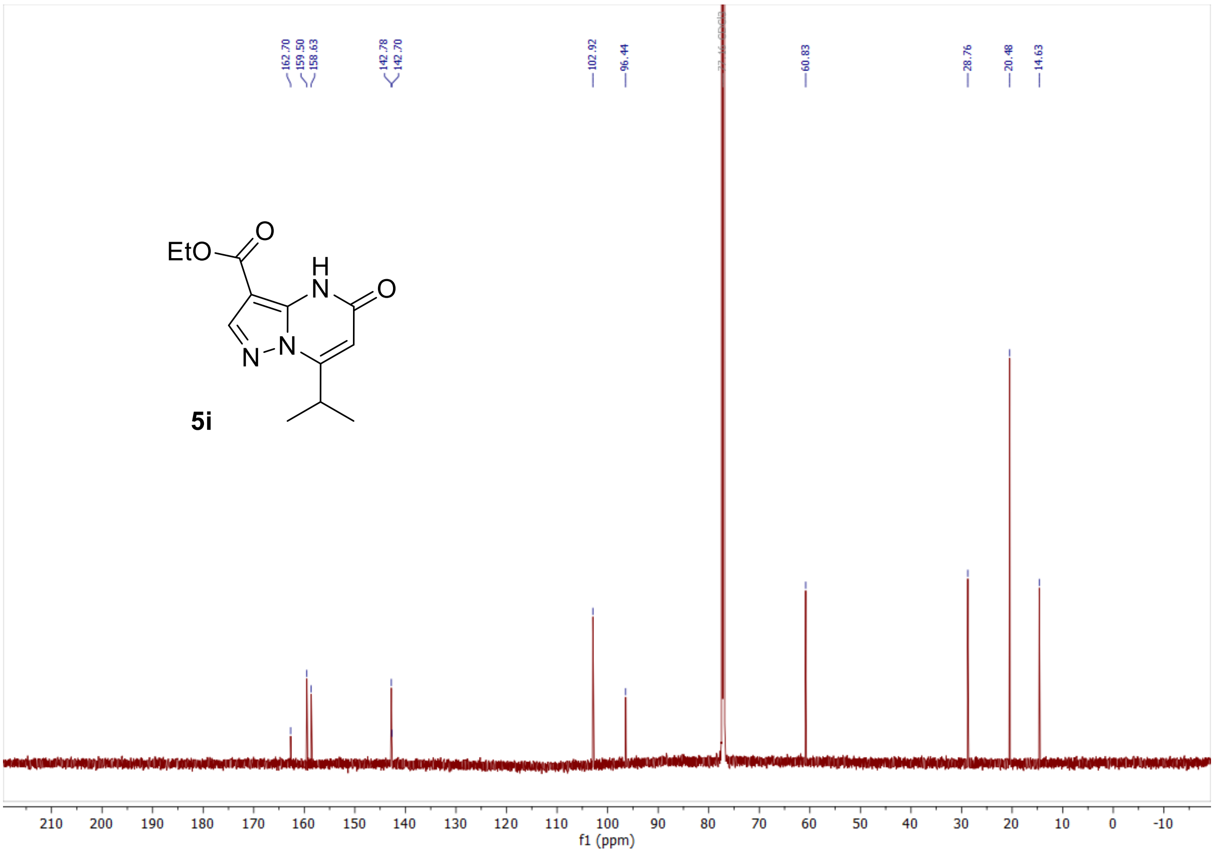

^{1}H NMR (600 MHz, CDCl₃) (5j)

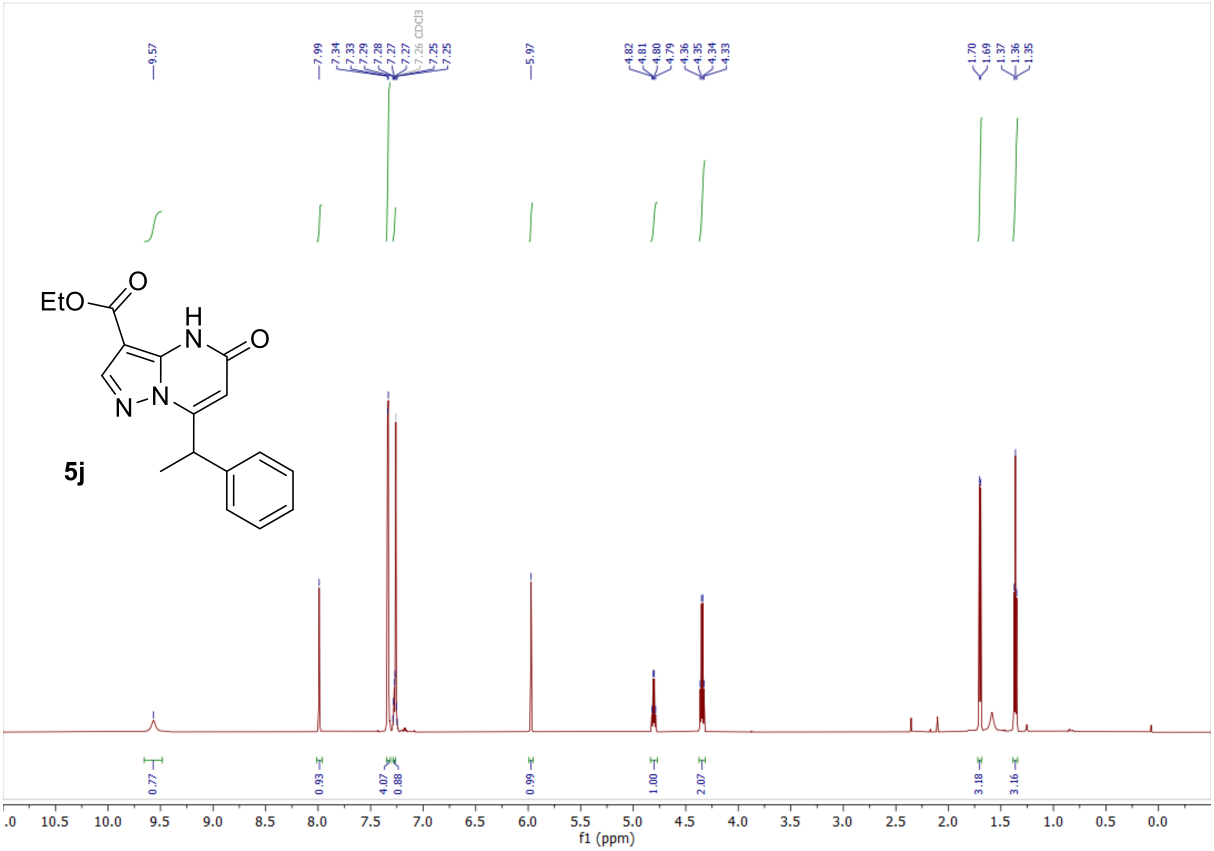

^{13}C NMR (151 MHz, CDCl₃) (5j)

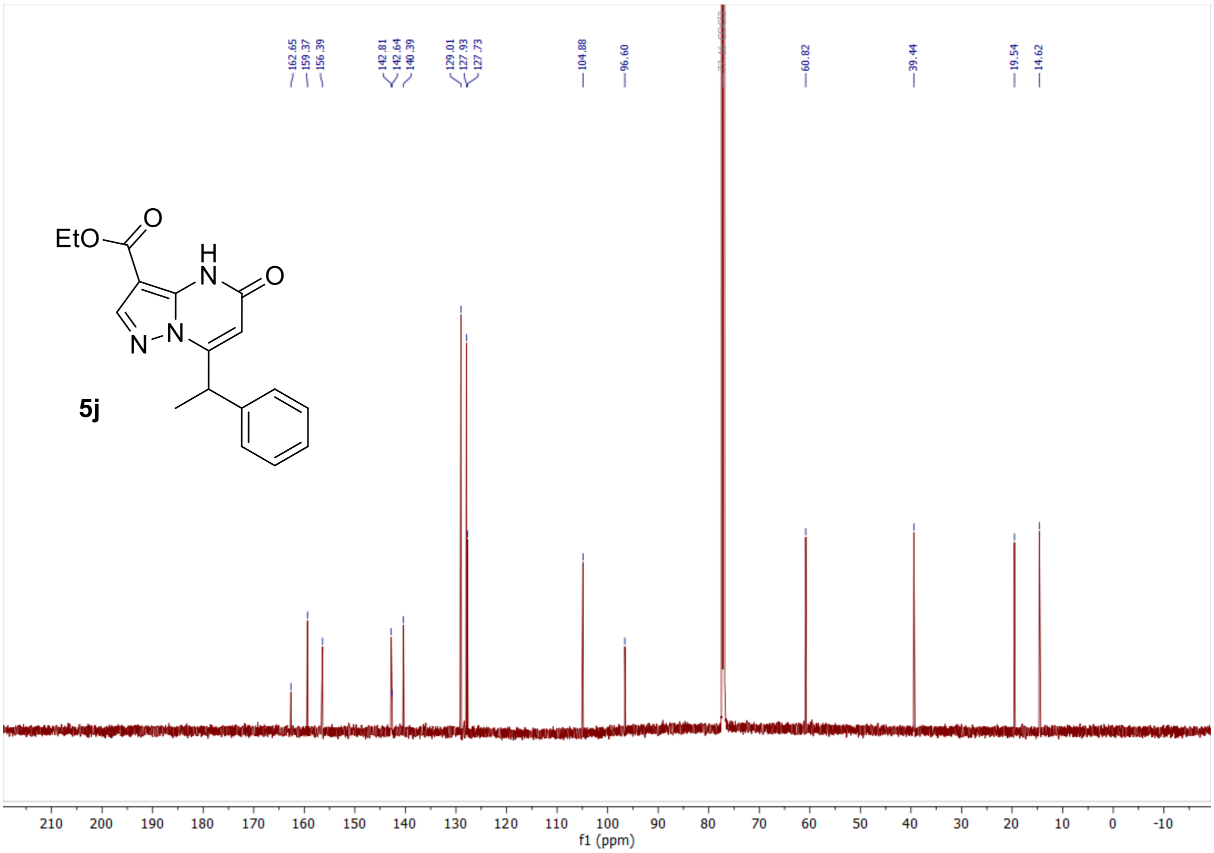

^{1}H NMR (400 MHz, $CDCl_3$) (5k)

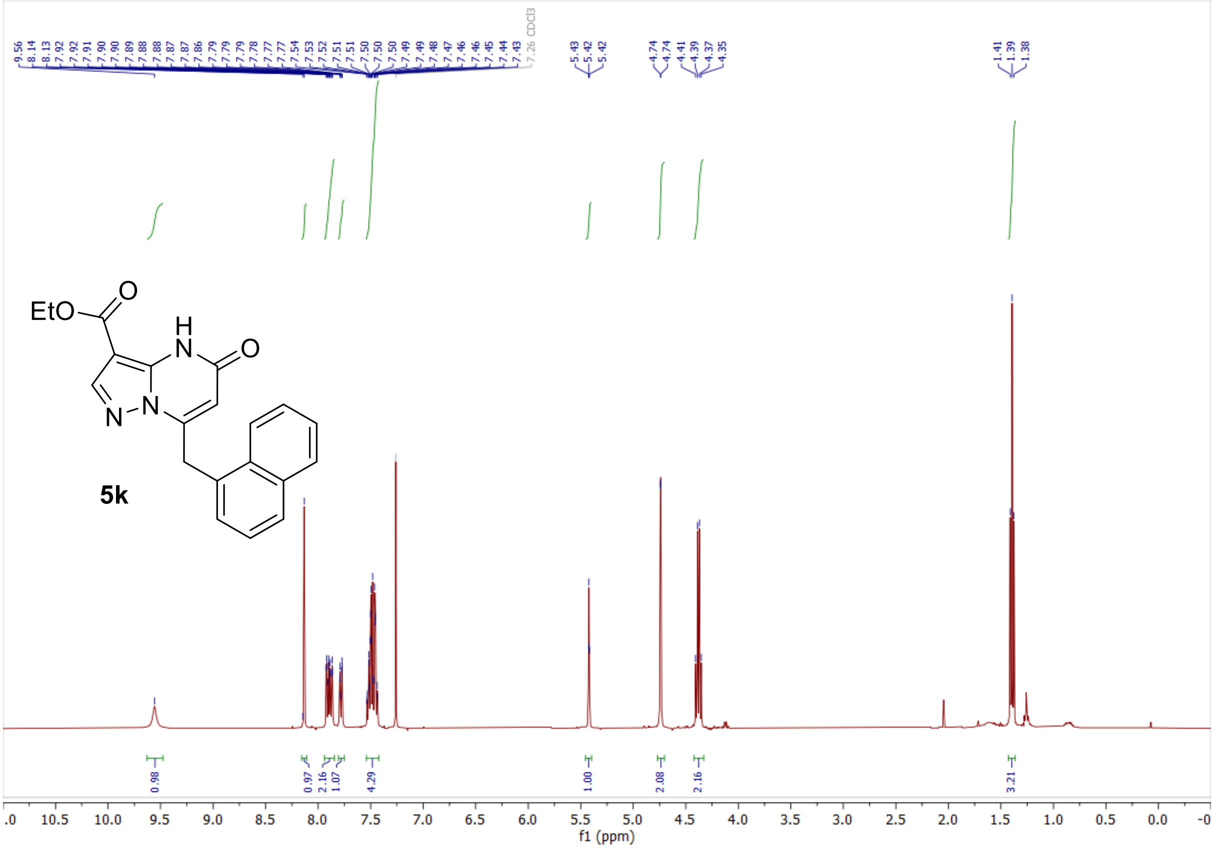

^{13}C NMR (100 MHz, $CDCl_3$) (5k)

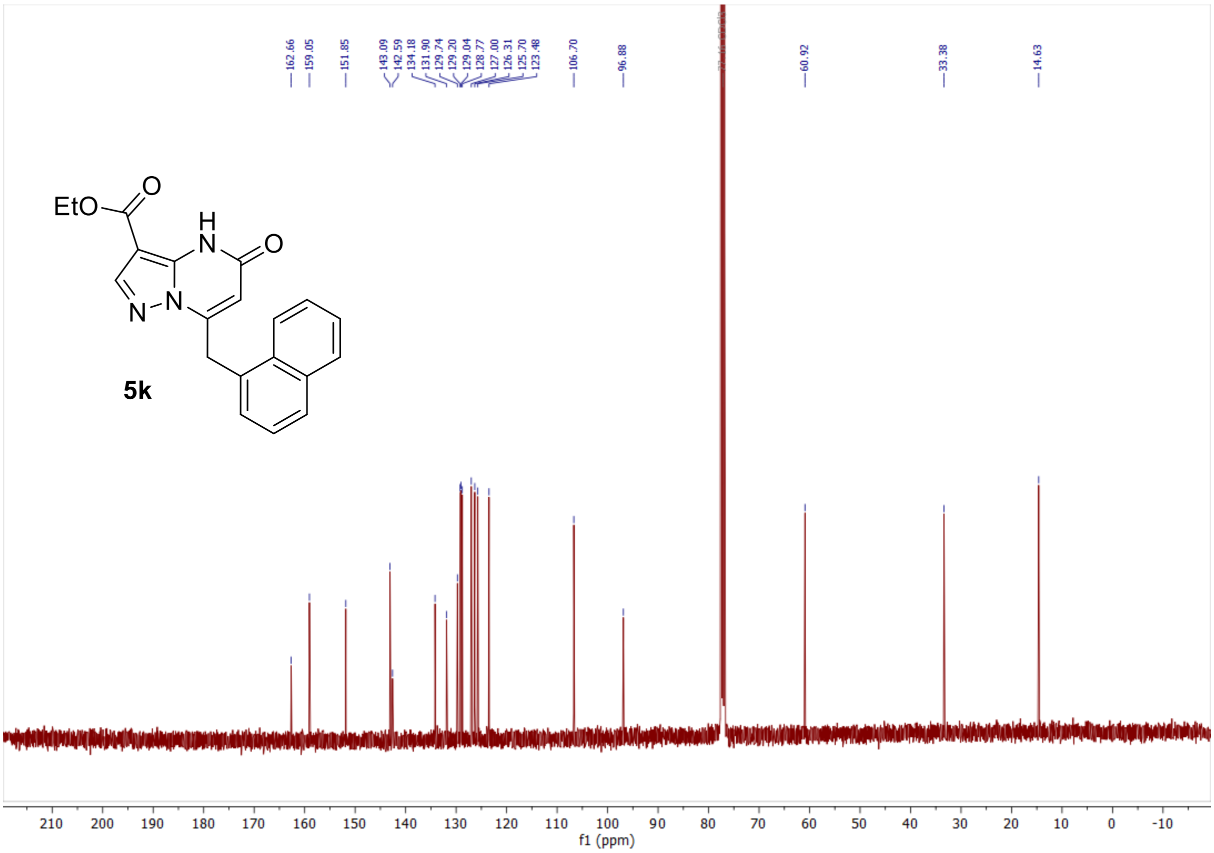

^{1}H NMR (600 MHz, $CDCl_3$) (5l)

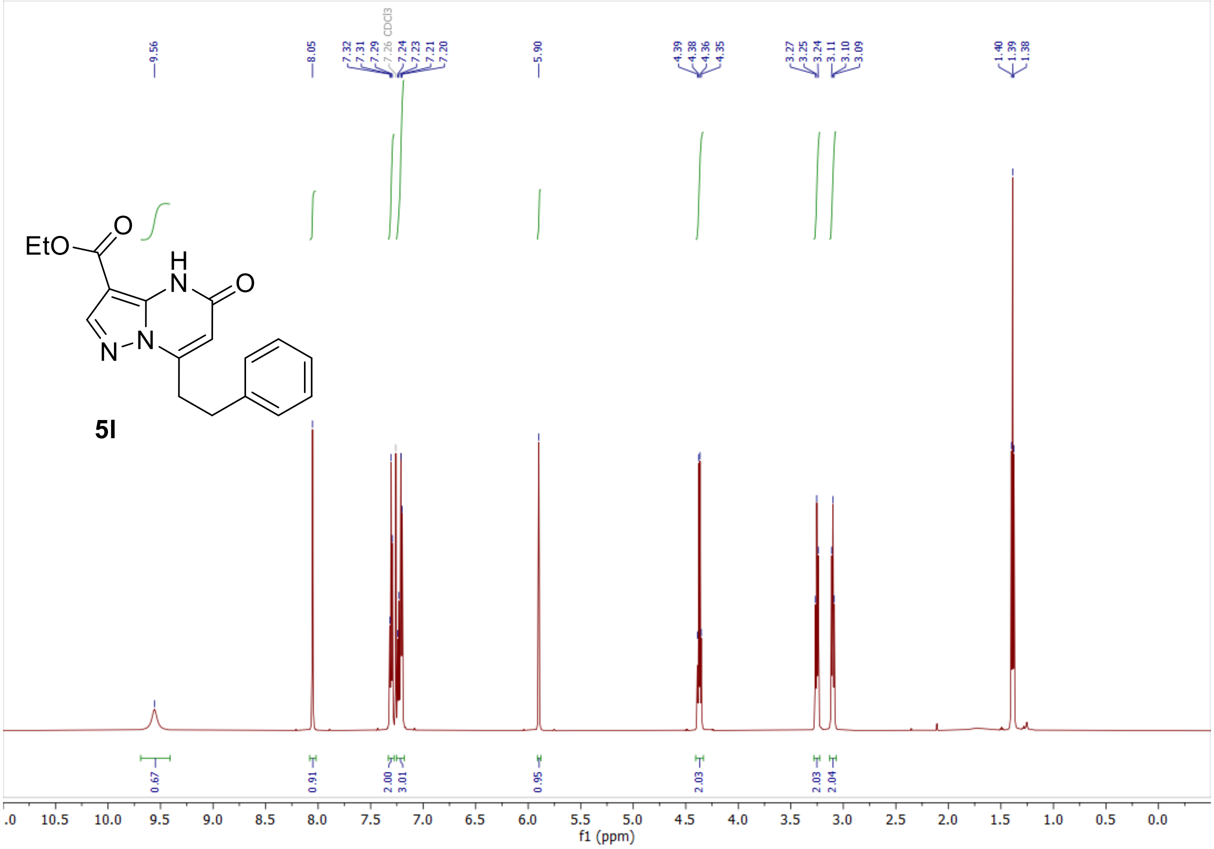

^{13}C NMR (151 MHz, $CDCl_3$) (5l)

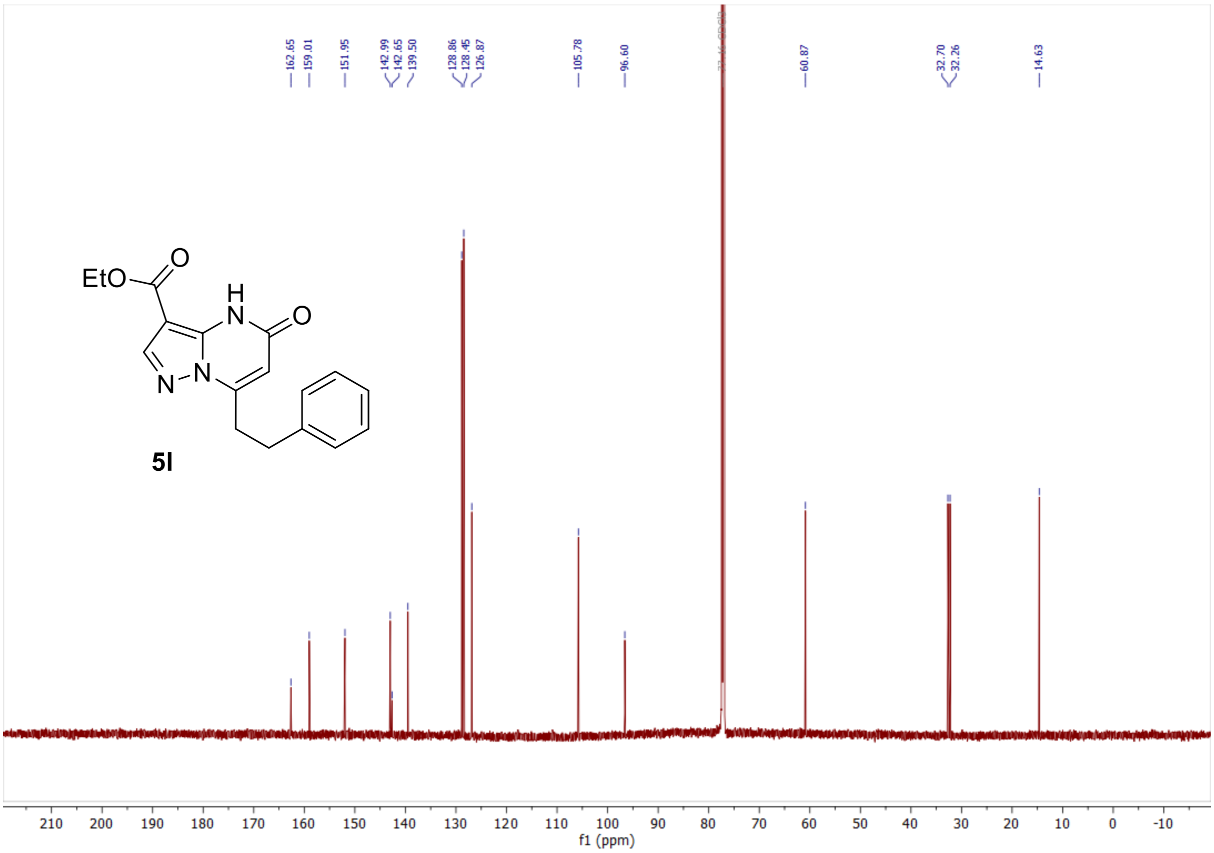

^1H NMR (600 MHz, $CDCl_3$) (5m)

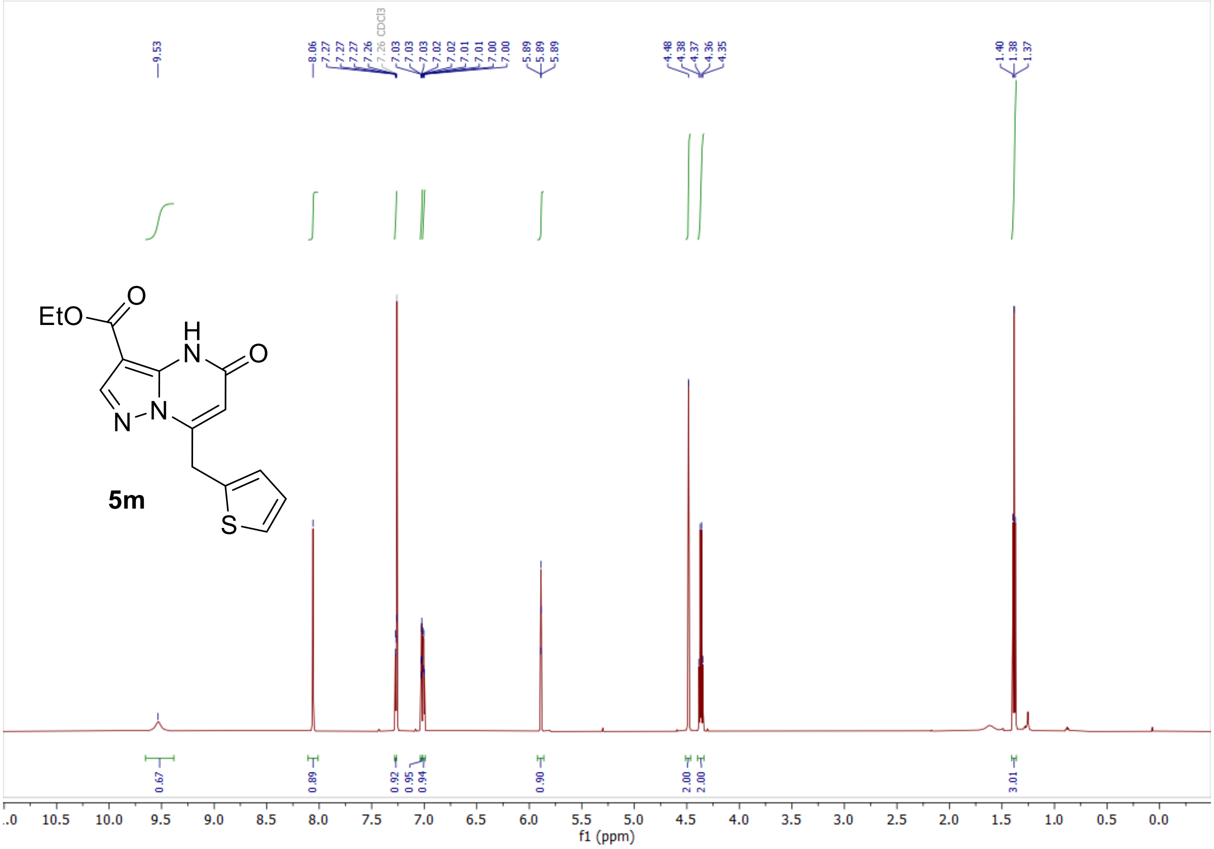

^{13}C NMR (151 MHz, $CDCl_3$) (5m)

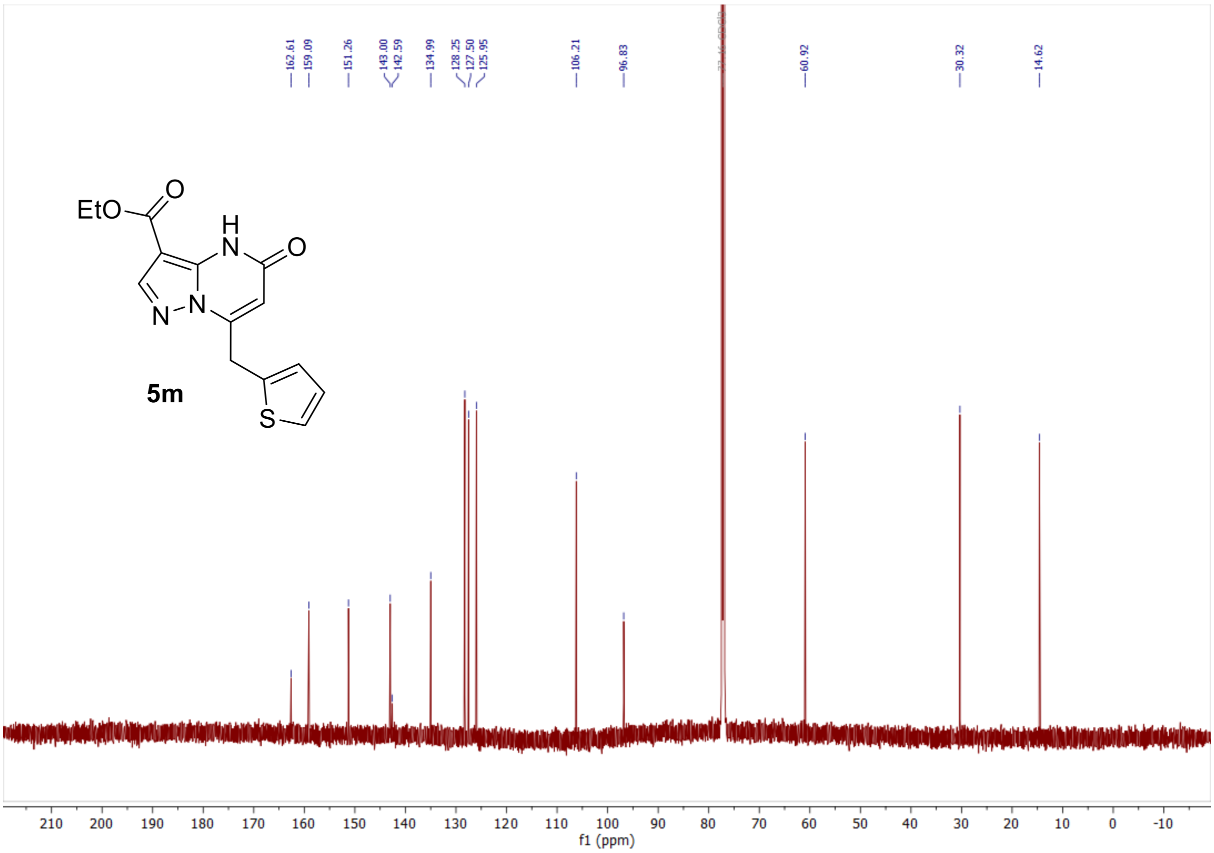

^{1}H NMR (400 MHz, $CDCl_3$) (5n)

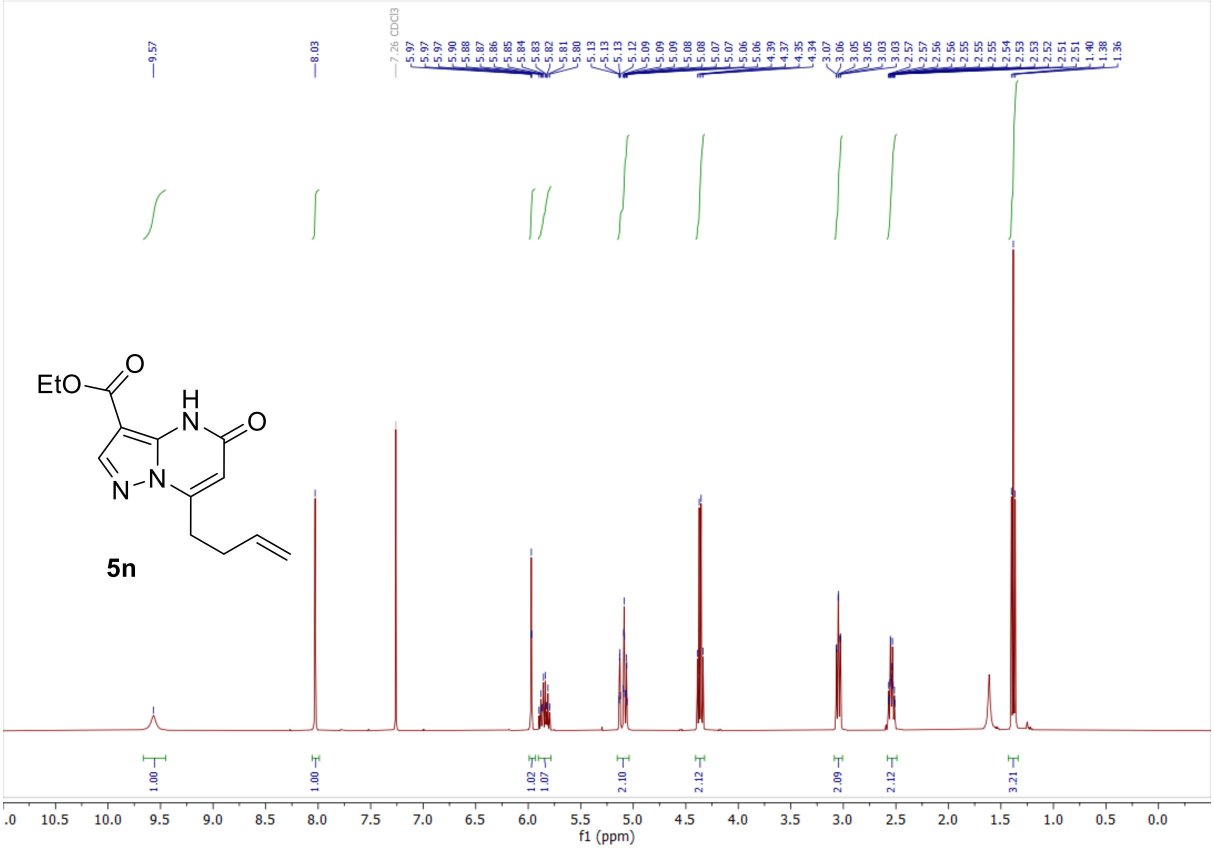

^{13}C NMR (100 MHz, $CDCl_3$) (5n)

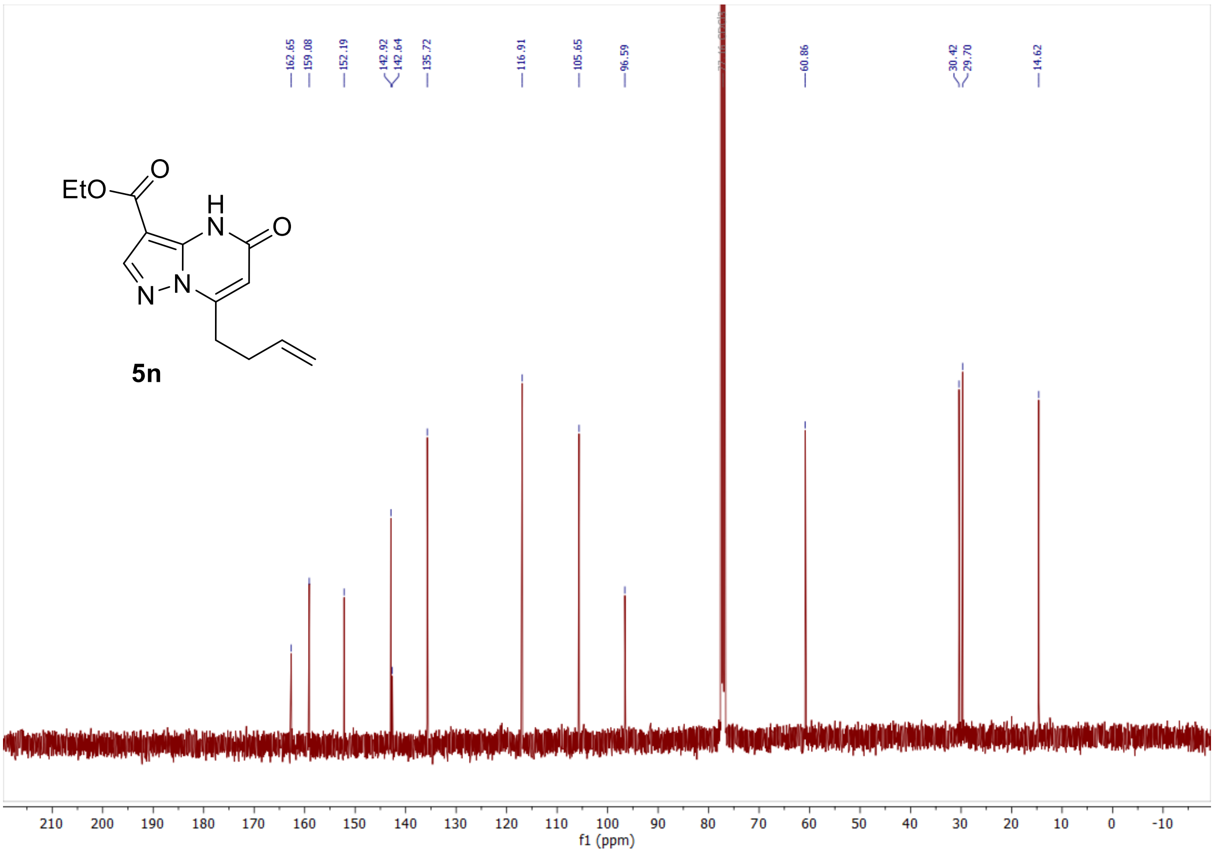

^{1}H NMR (600 MHz, $CDCl_3$) (5o)

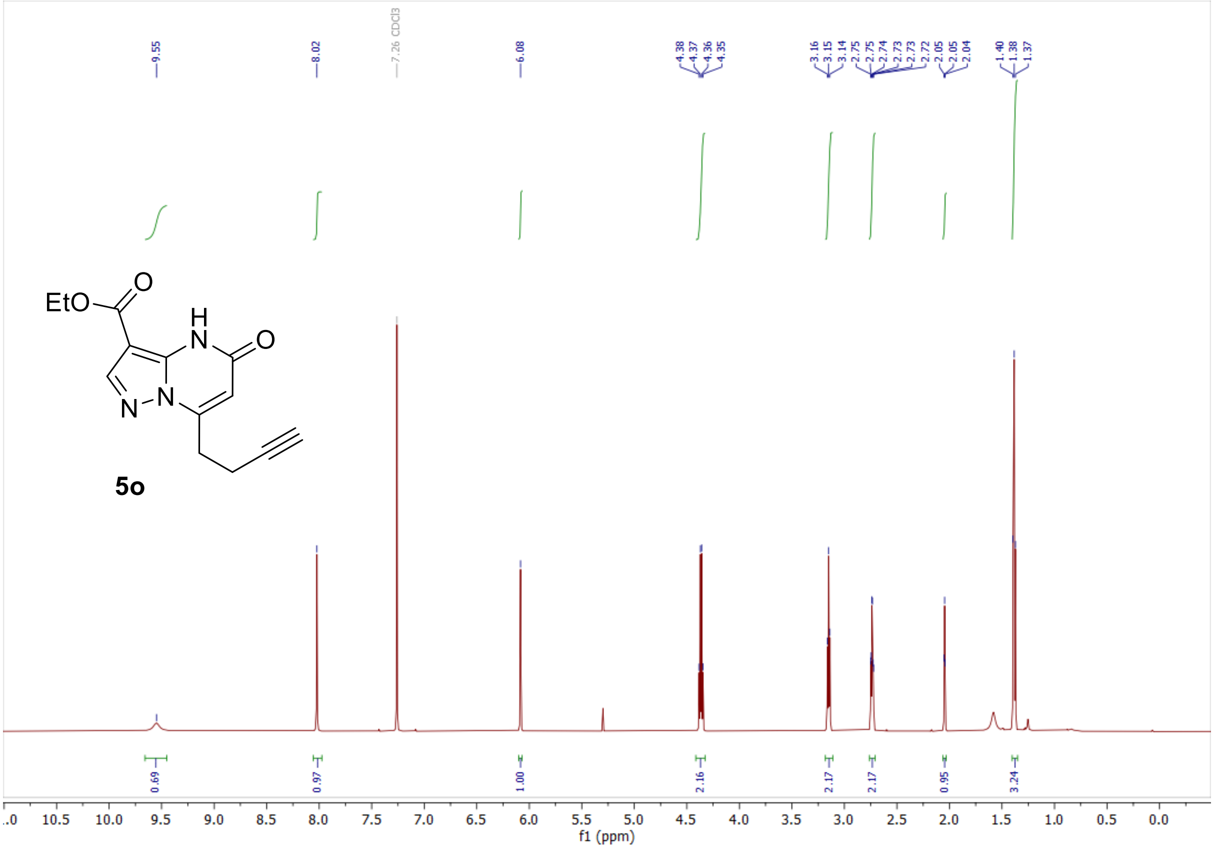

^{13}C NMR (151 MHz, $CDCl_3$) (5o)

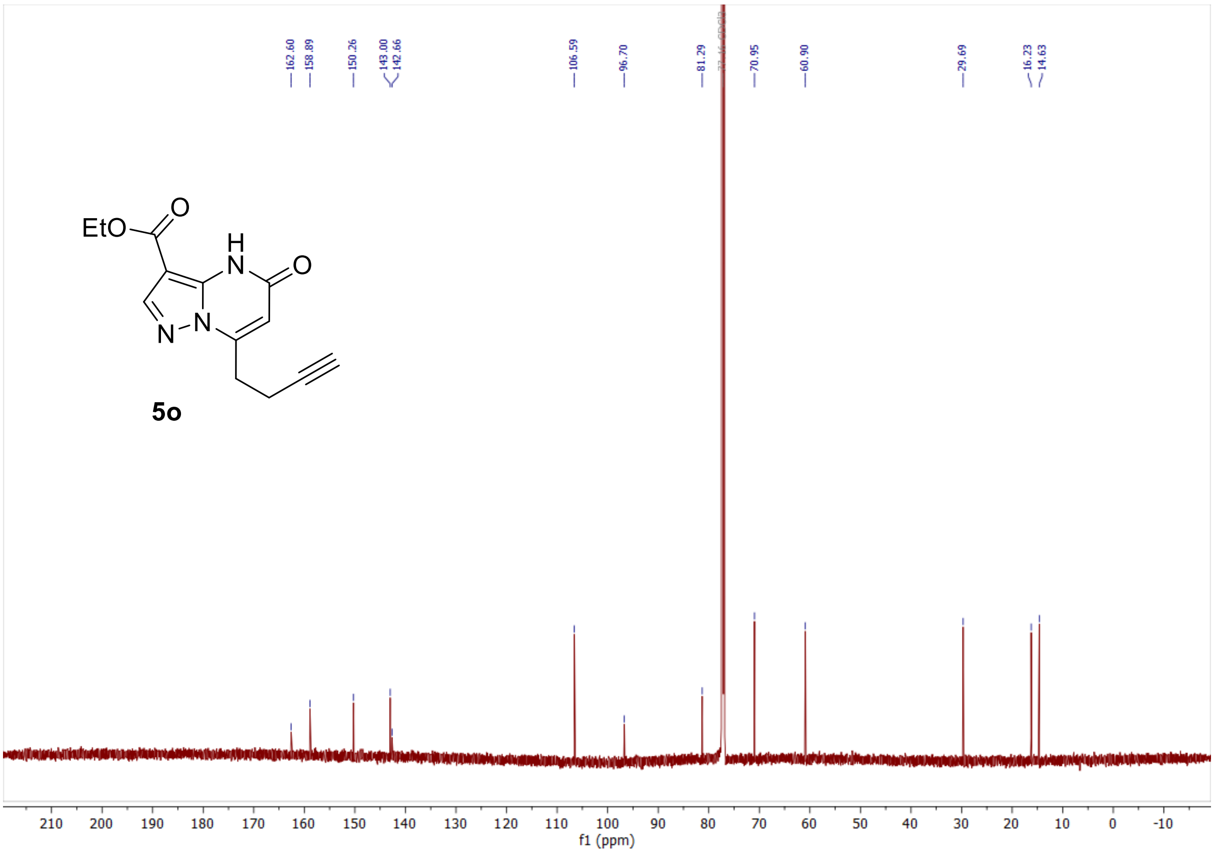

^{1}H NMR (600 MHz, $CDCl_3$) (5p)

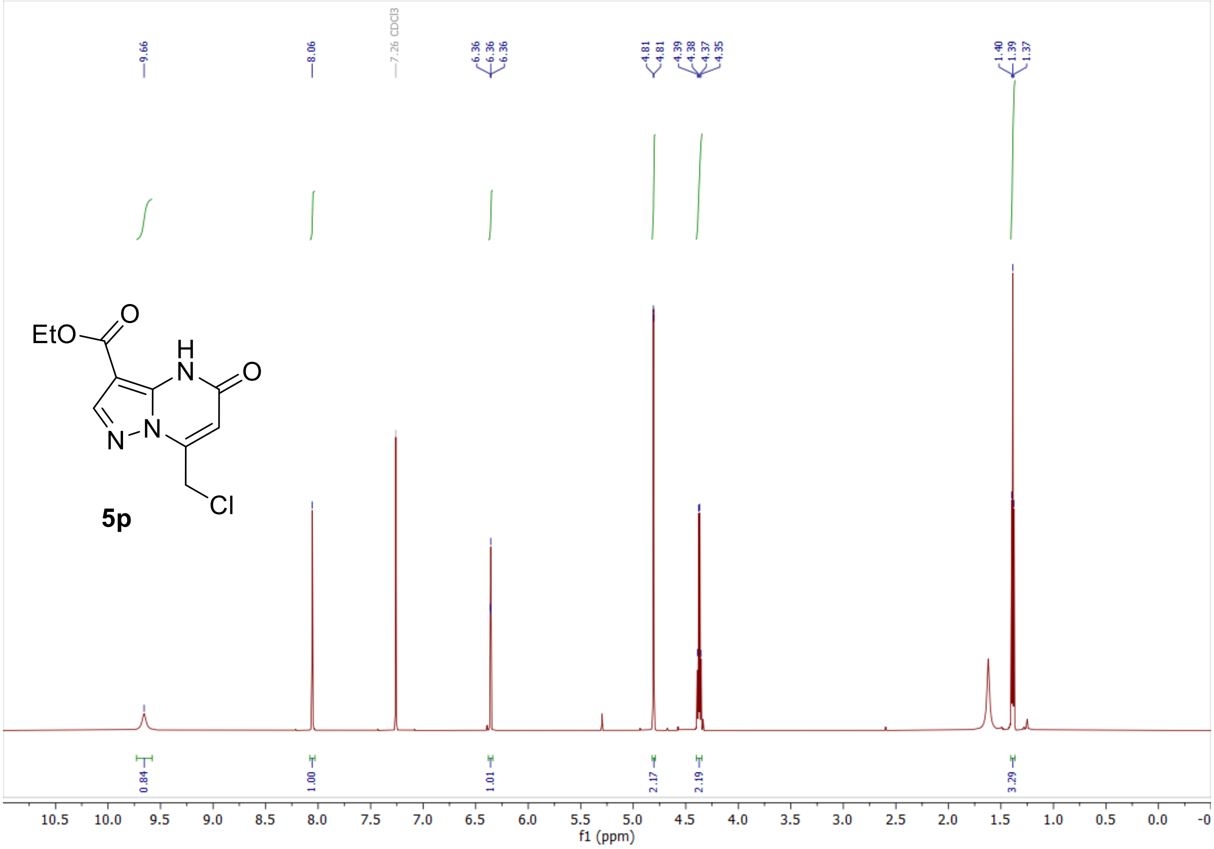

^{13}C NMR (151 MHz, $CDCl_3$) (5p)

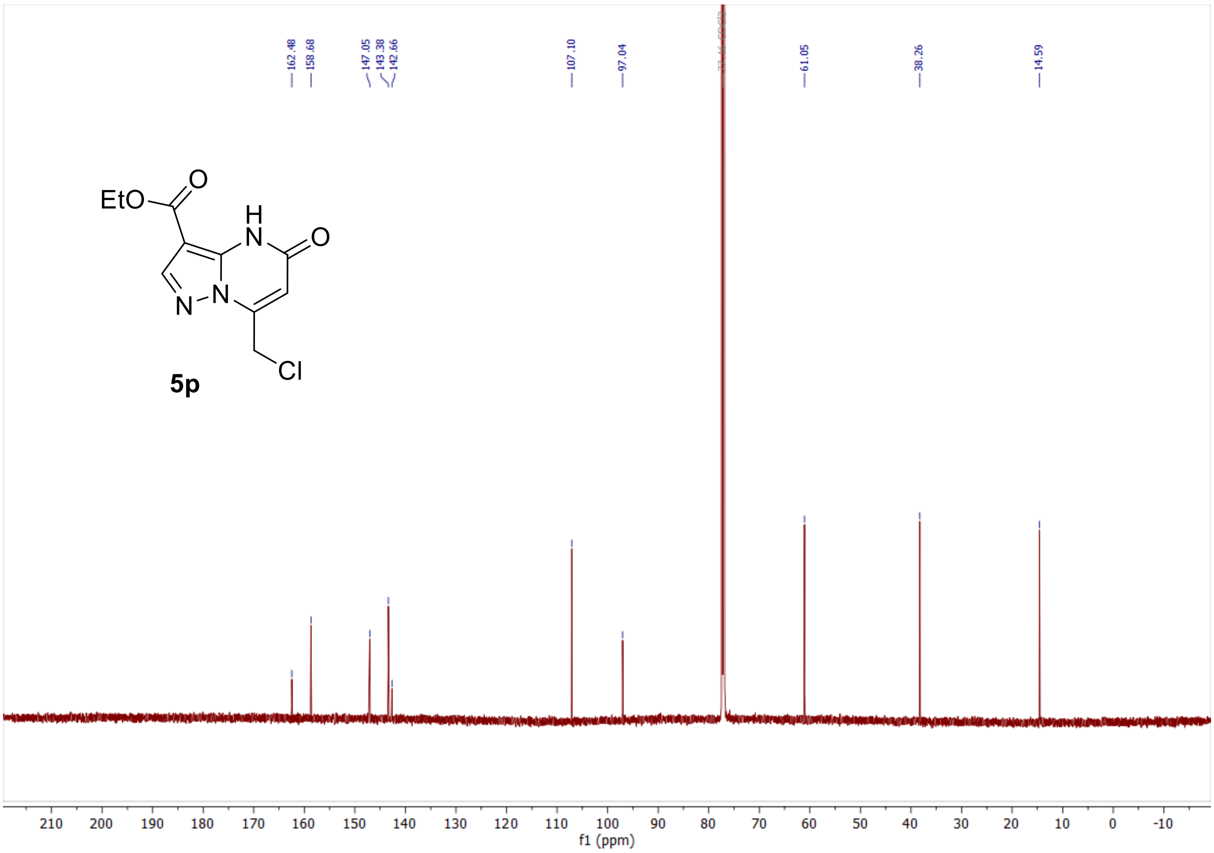

^{1}H NMR (600 MHz, $CDCl_3$) (5q)

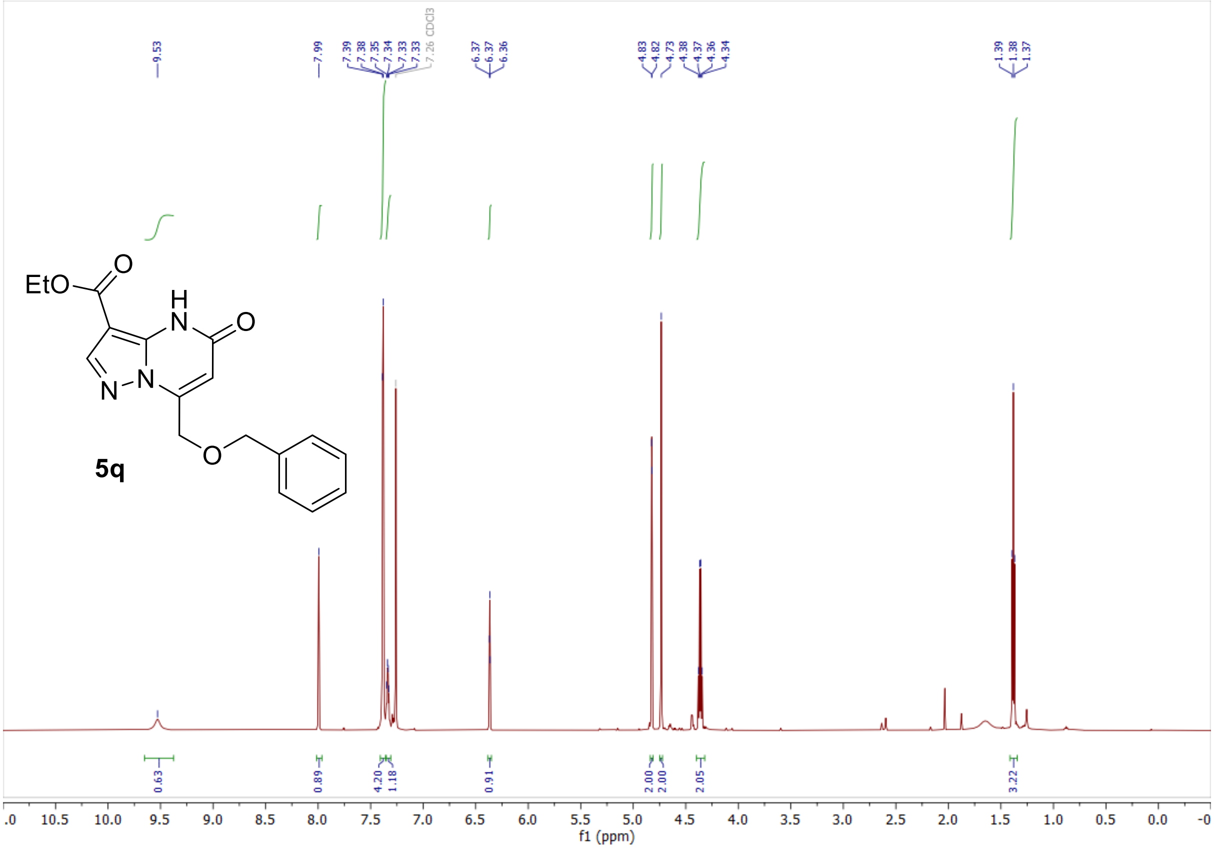

^{13}C NMR (151 MHz, $CDCl_3$) (5q)

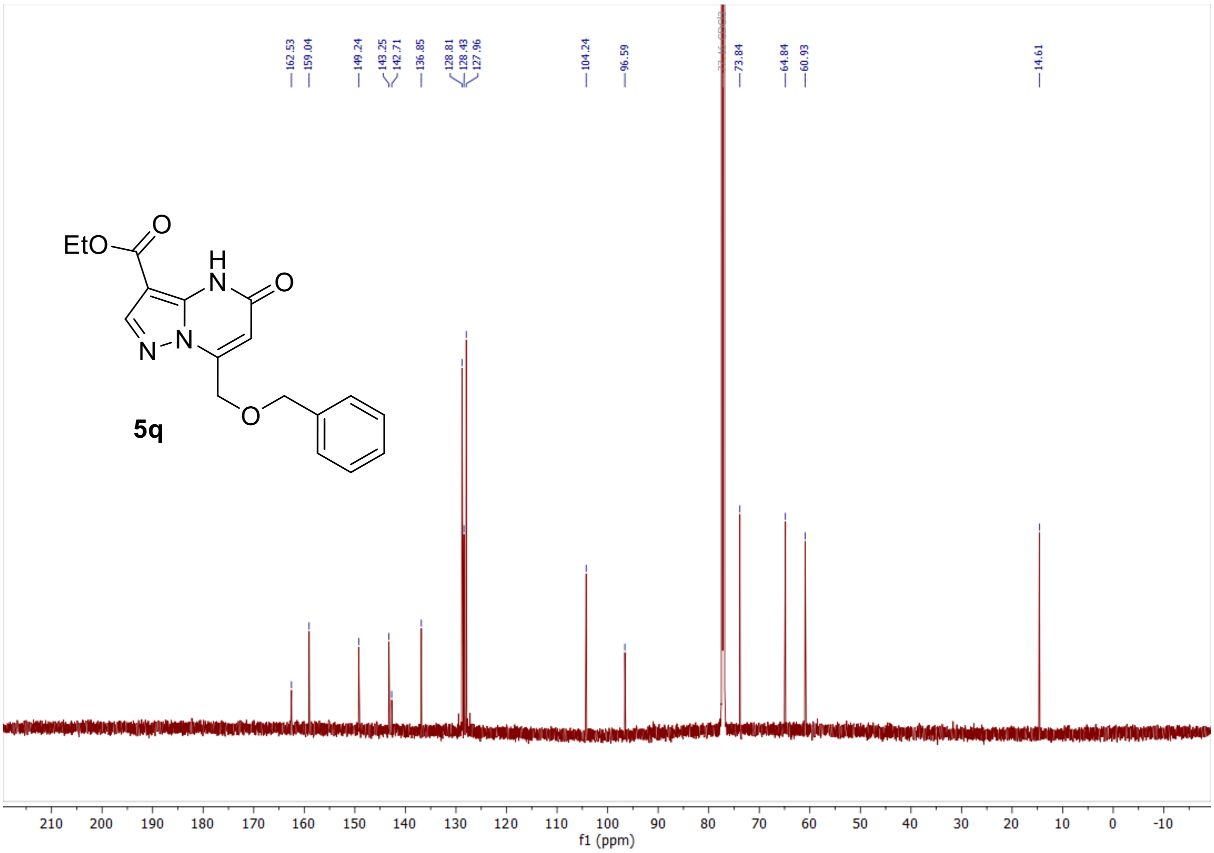

^{1}H NMR (400 MHz, $CDCl_3$) (5r)

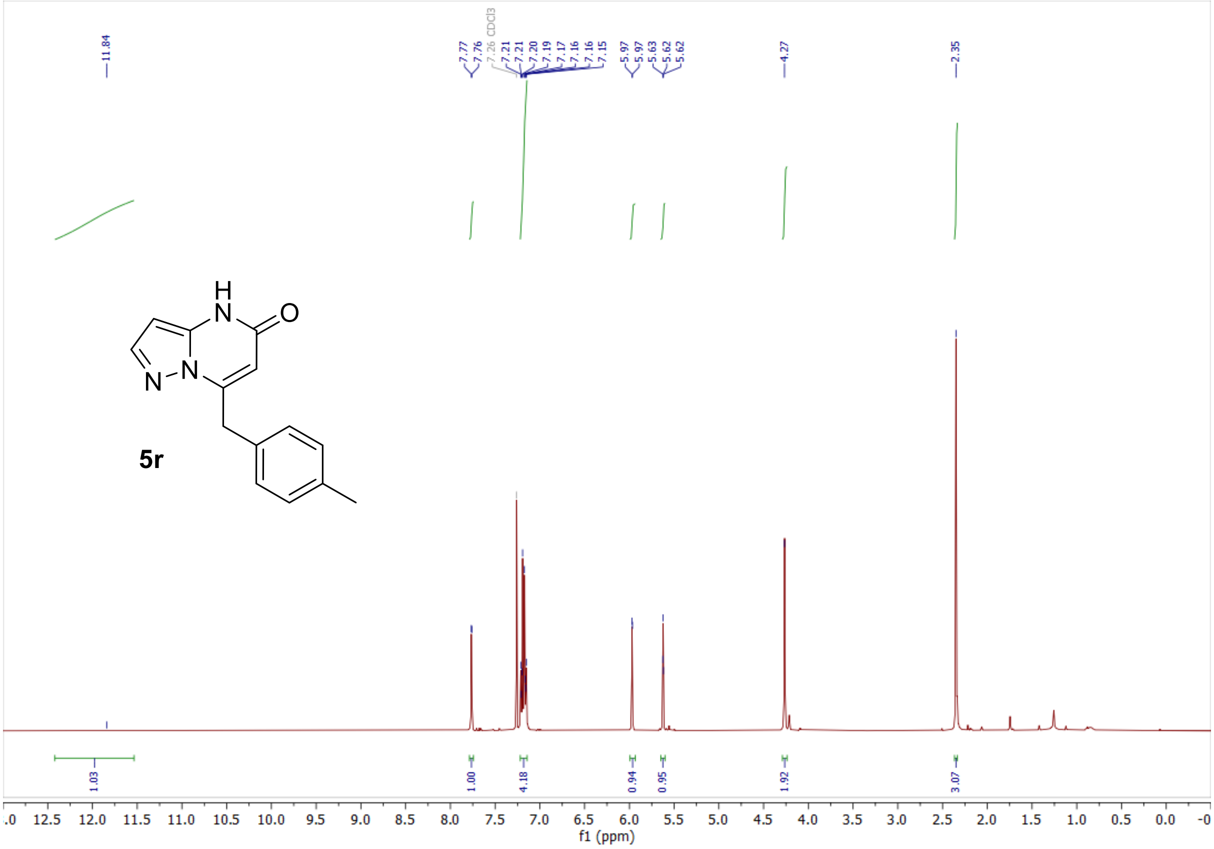

^{13}C NMR (100 MHz, $CDCl_3$) (5r)

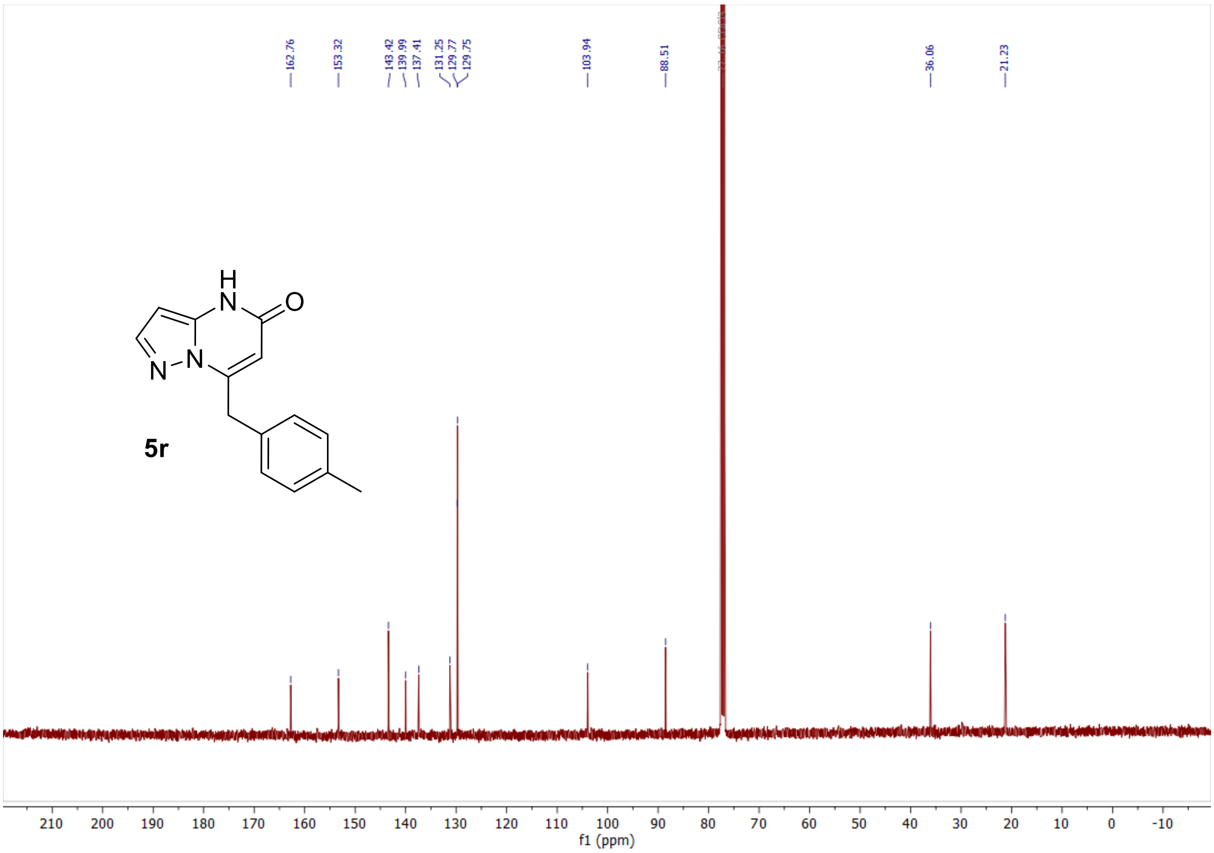

^{1}H NMR (600 MHz, $CDCl_3$) (5s)

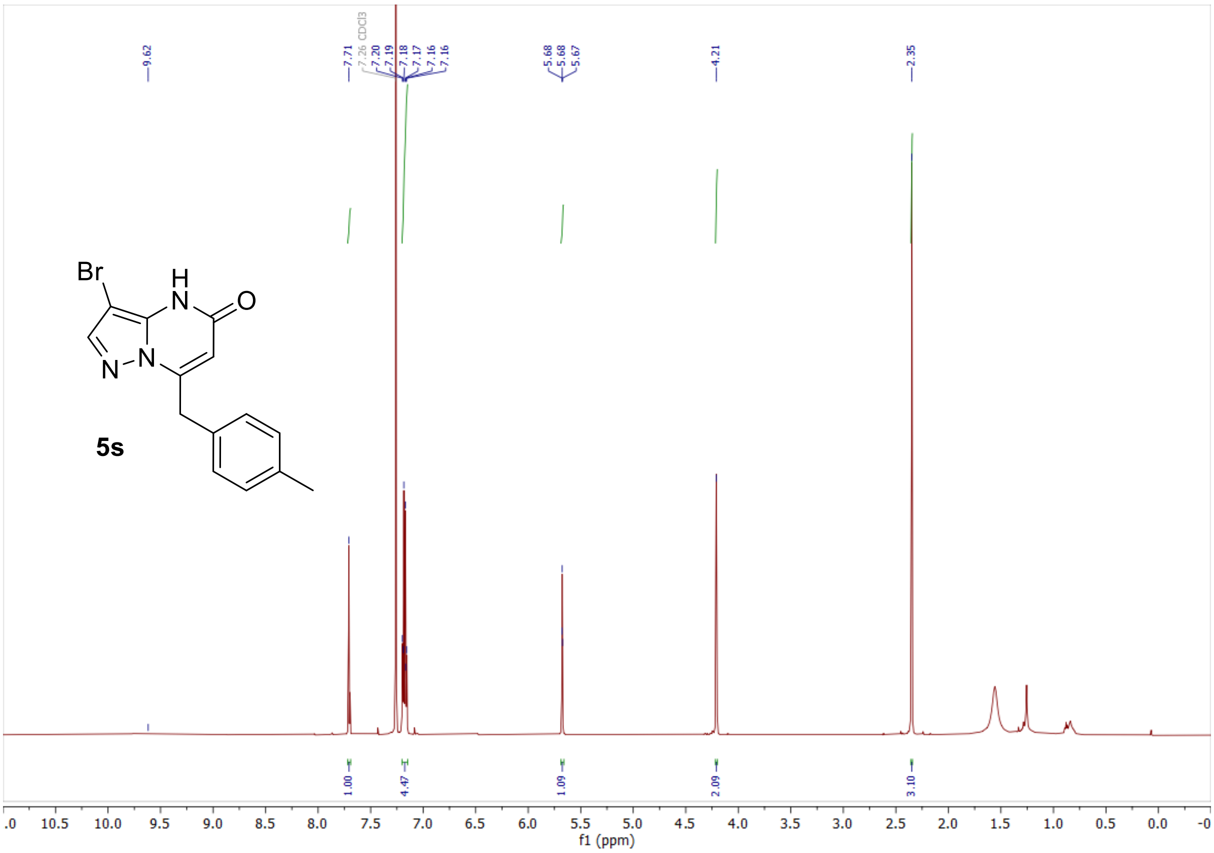

^{13}C NMR (151 MHz, $CDCl_3$) (5s)

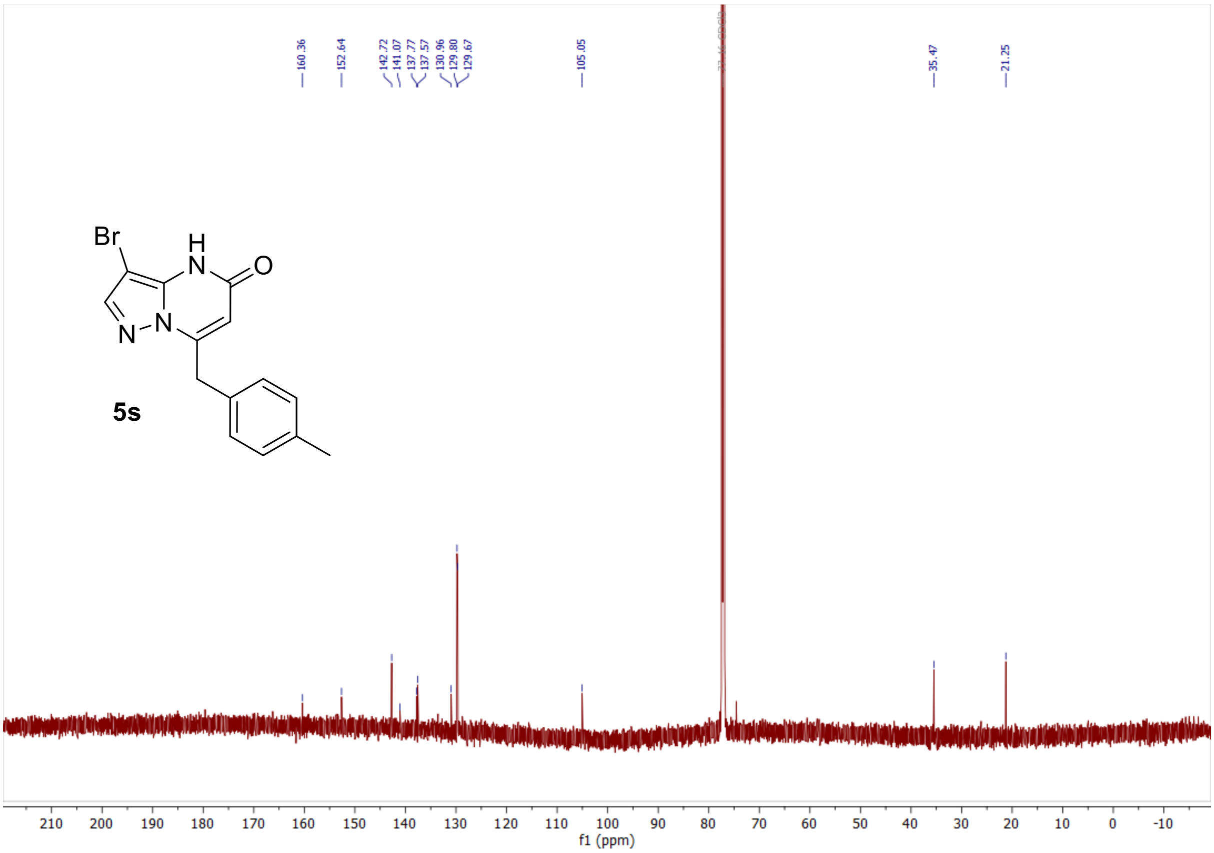

^{1}H NMR (400 MHz, $CDCl_3$) (5t)

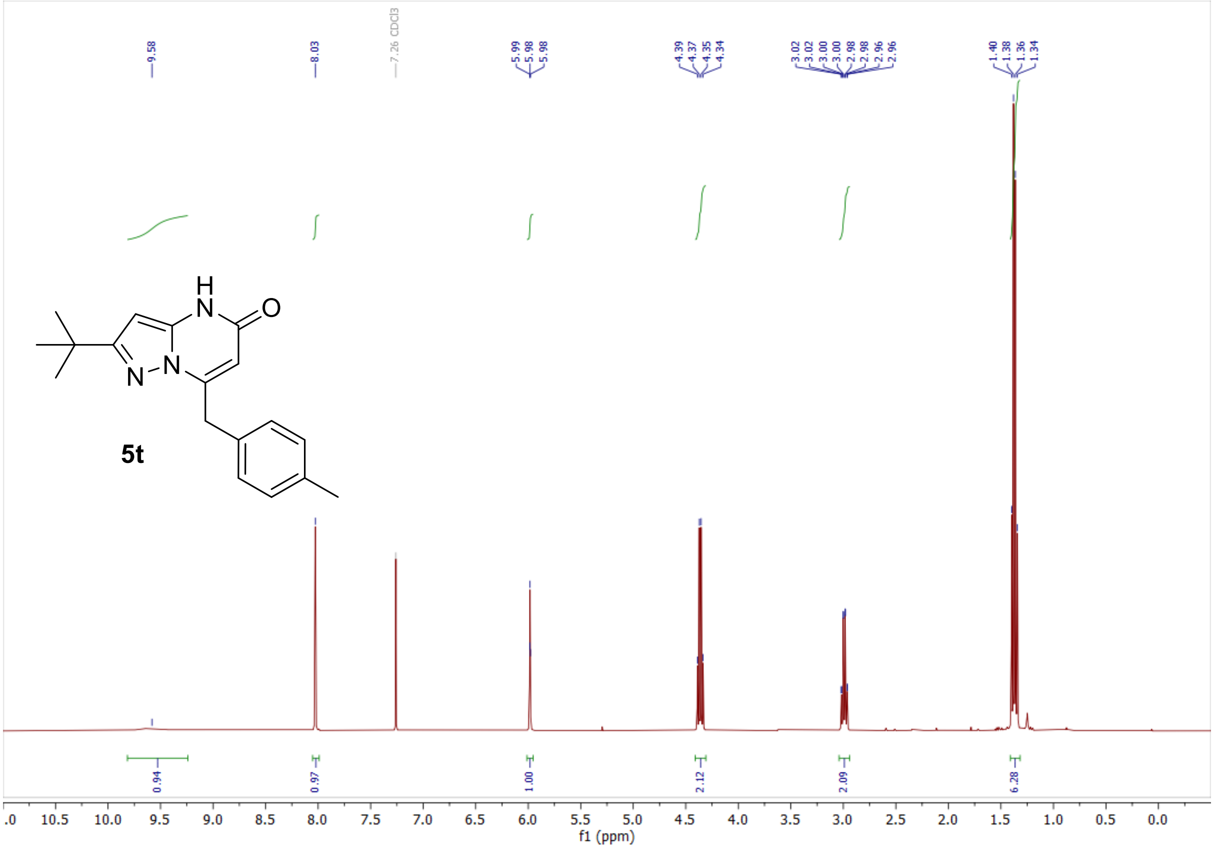

^{13}C NMR (100 MHz, $CDCl_3$) (5t)

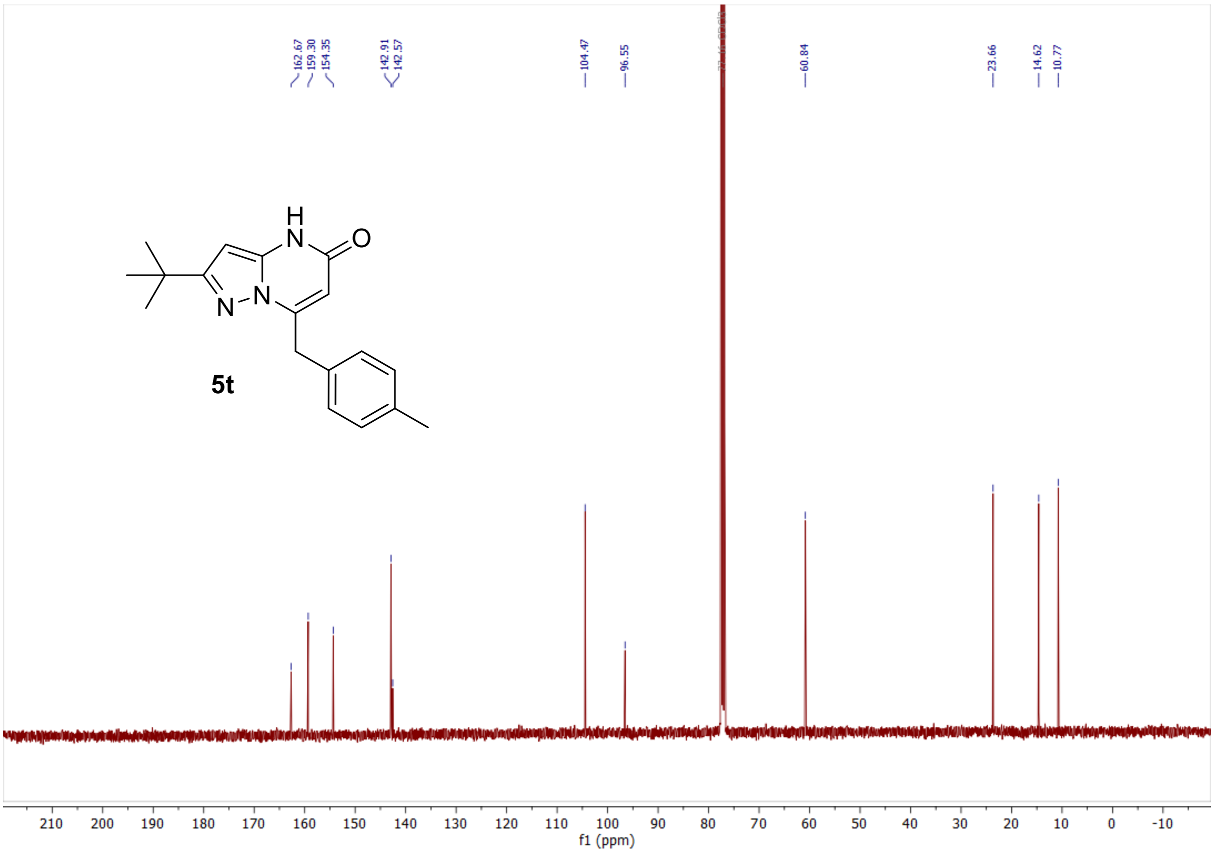

^{1}H NMR (400 MHz, $CDCl_3$) (5u)

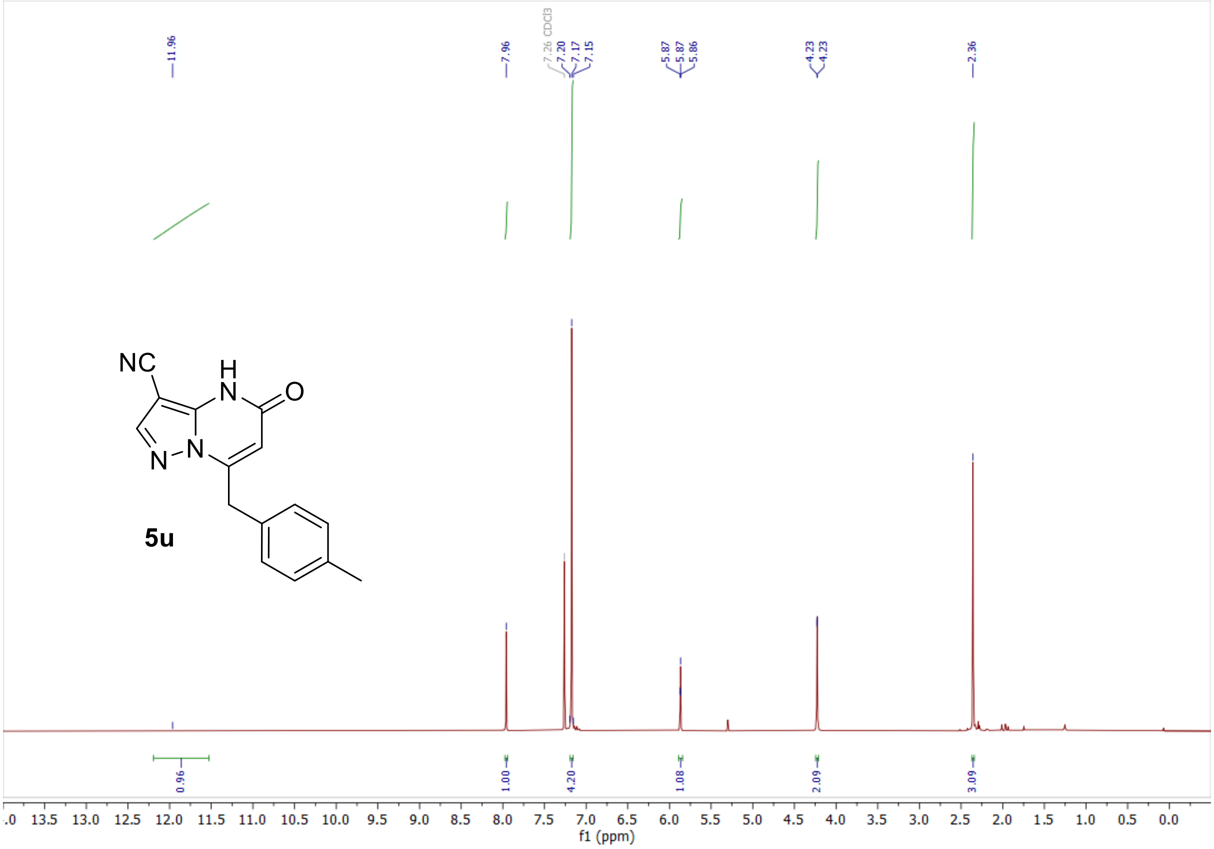

^{13}C NMR (100 MHz, $CDCl_3$) (5u)

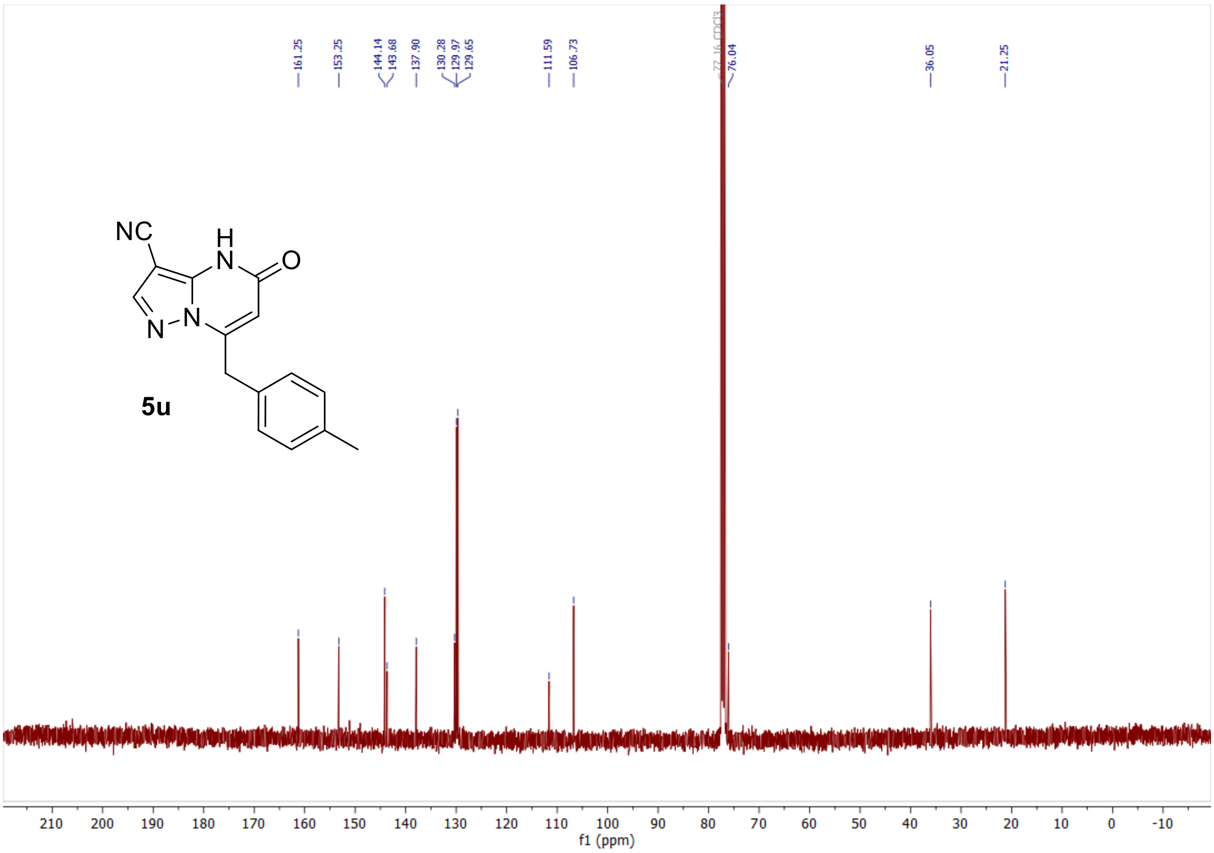

^{1}H NMR (400 MHz, DMSO) (5v)

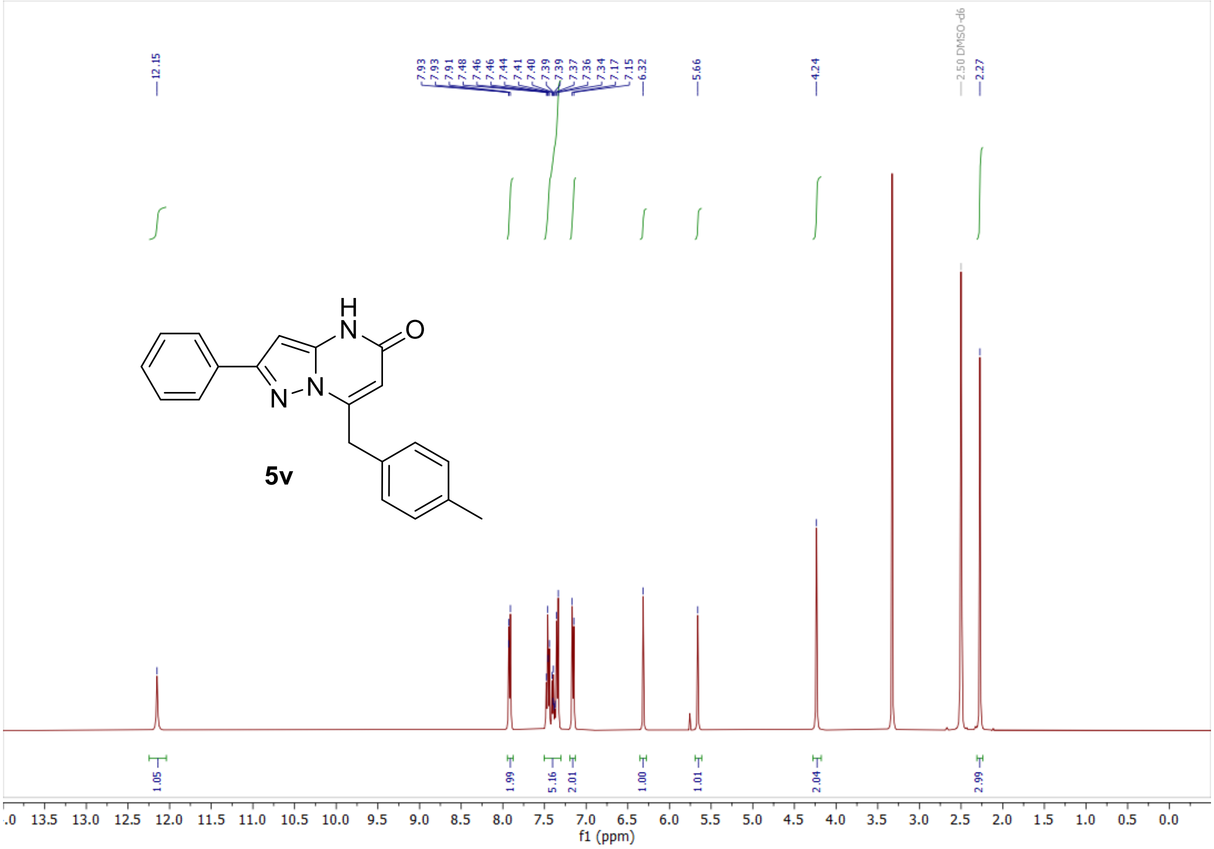

5v

^{13}C NMR (151 MHz, DMSO) (5v)

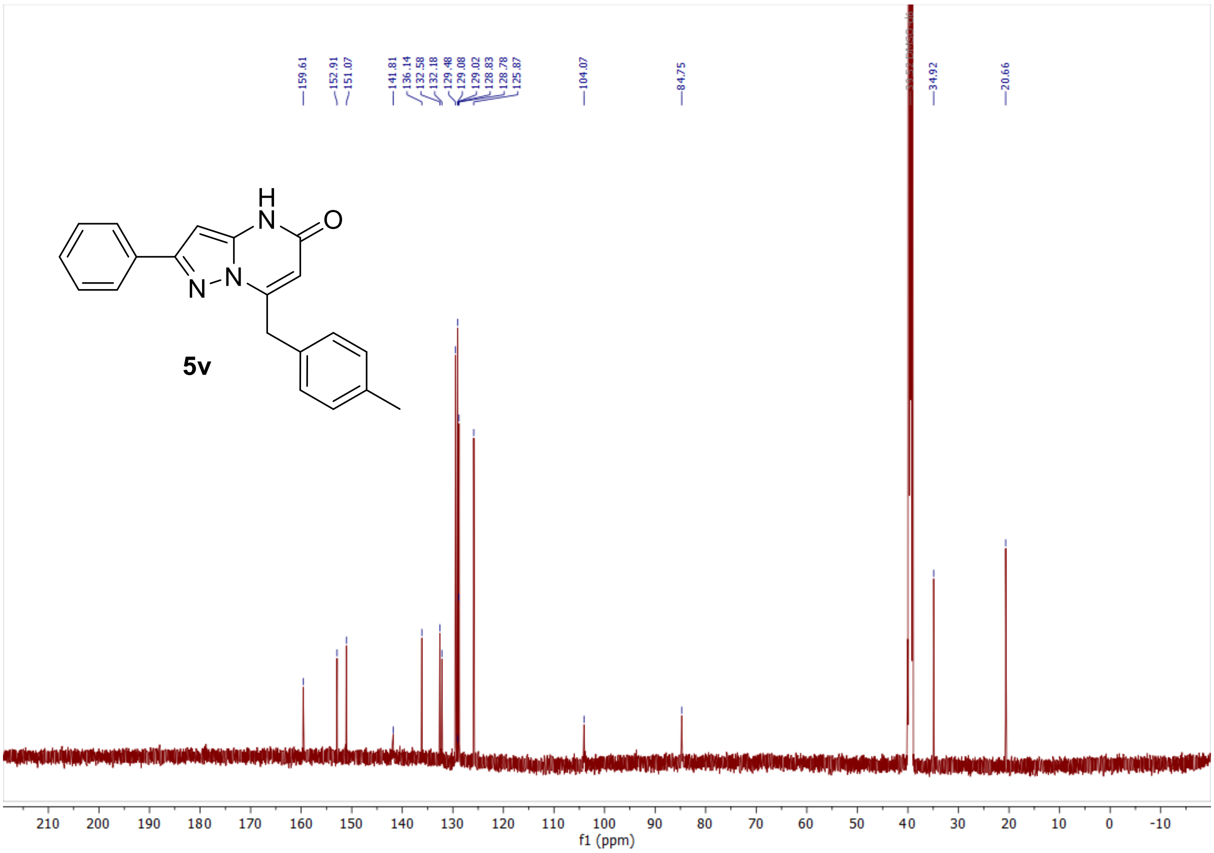

5v

^{1}H NMR (600 MHz, $CDCl_3$) (5w)

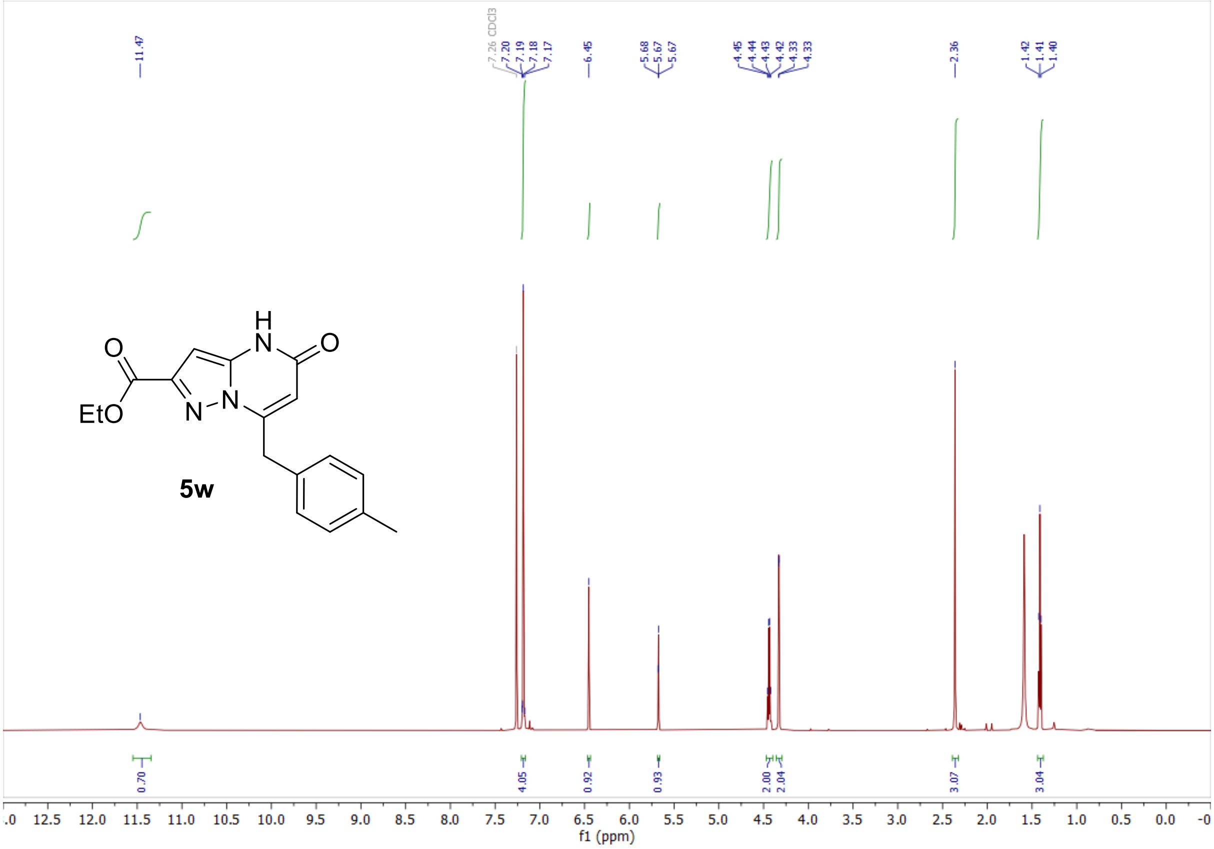

^{13}C NMR (151 MHz, $CDCl_3$) (5w)

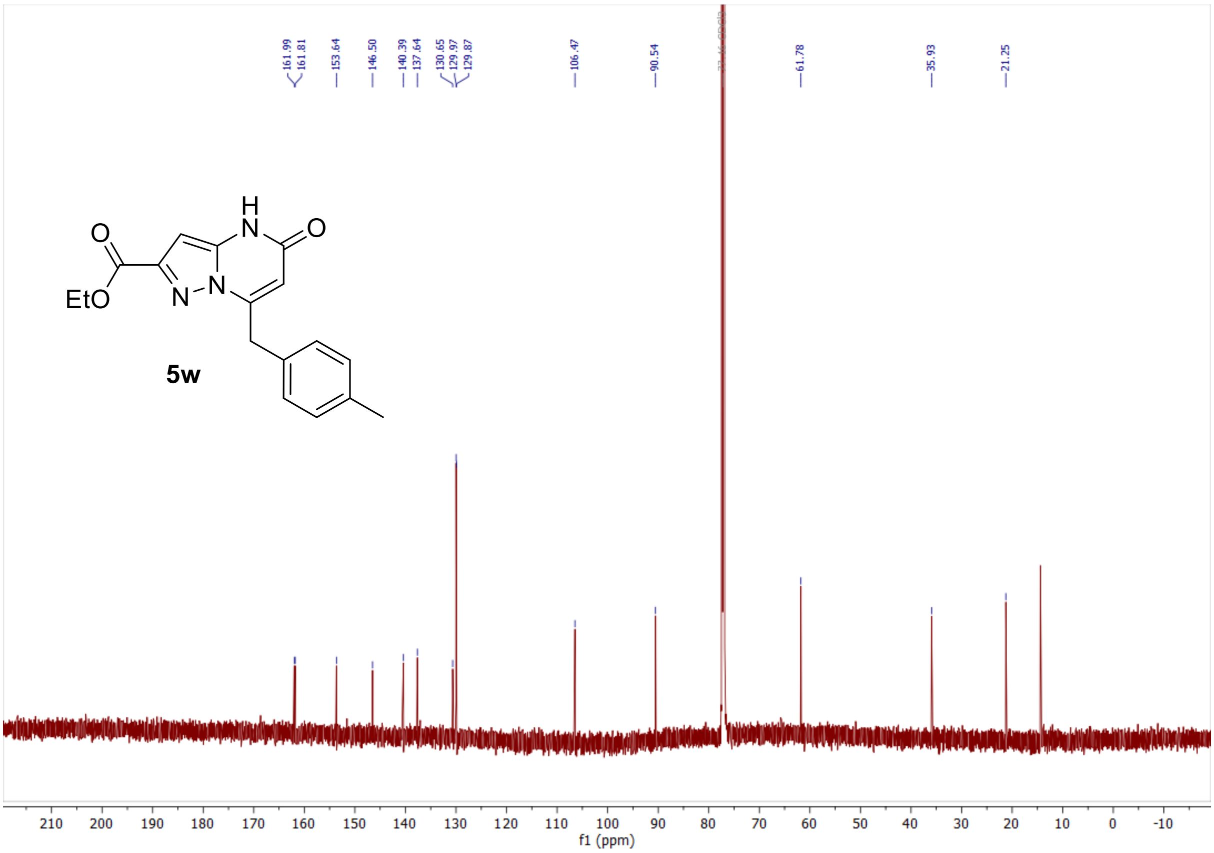

^{1}H NMR (600 MHz, $CDCl_3$) (5x)

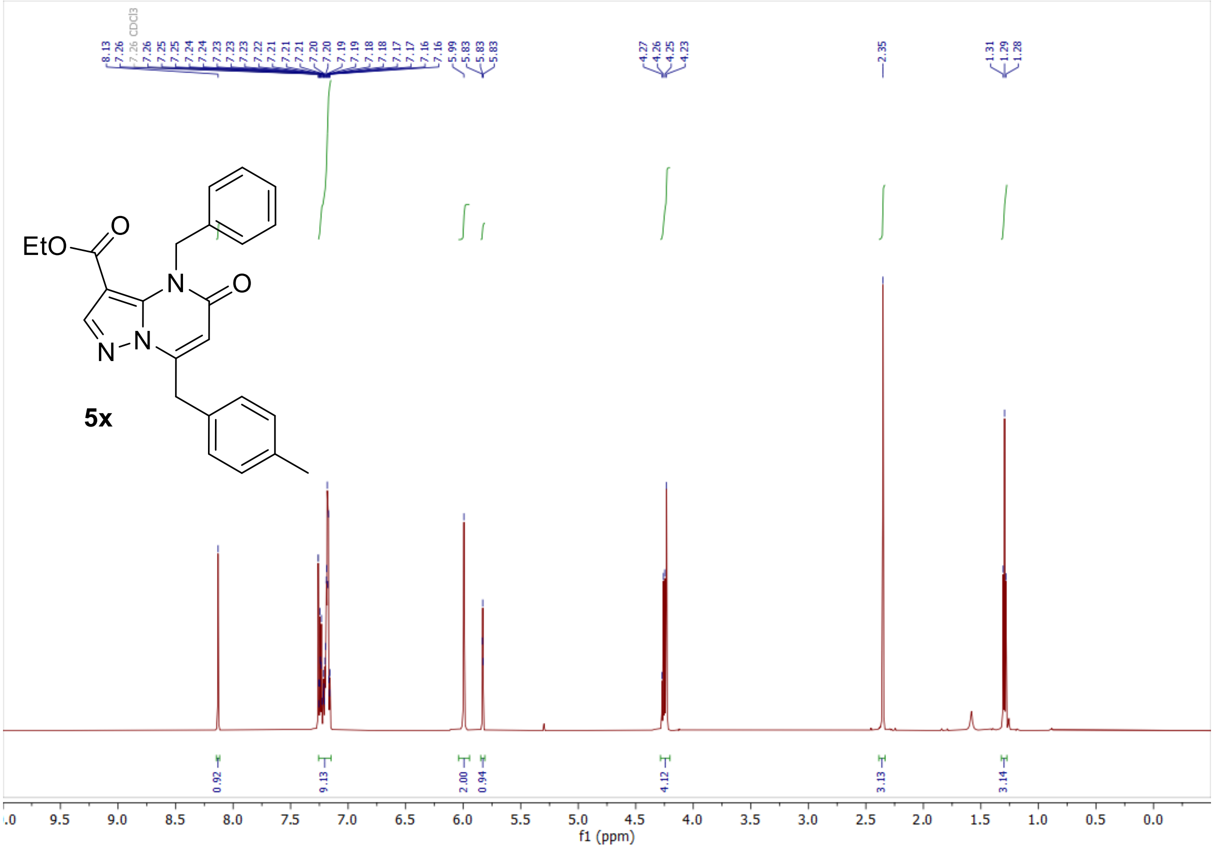

^{13}C NMR (151 MHz, $CDCl_3$) (5x)

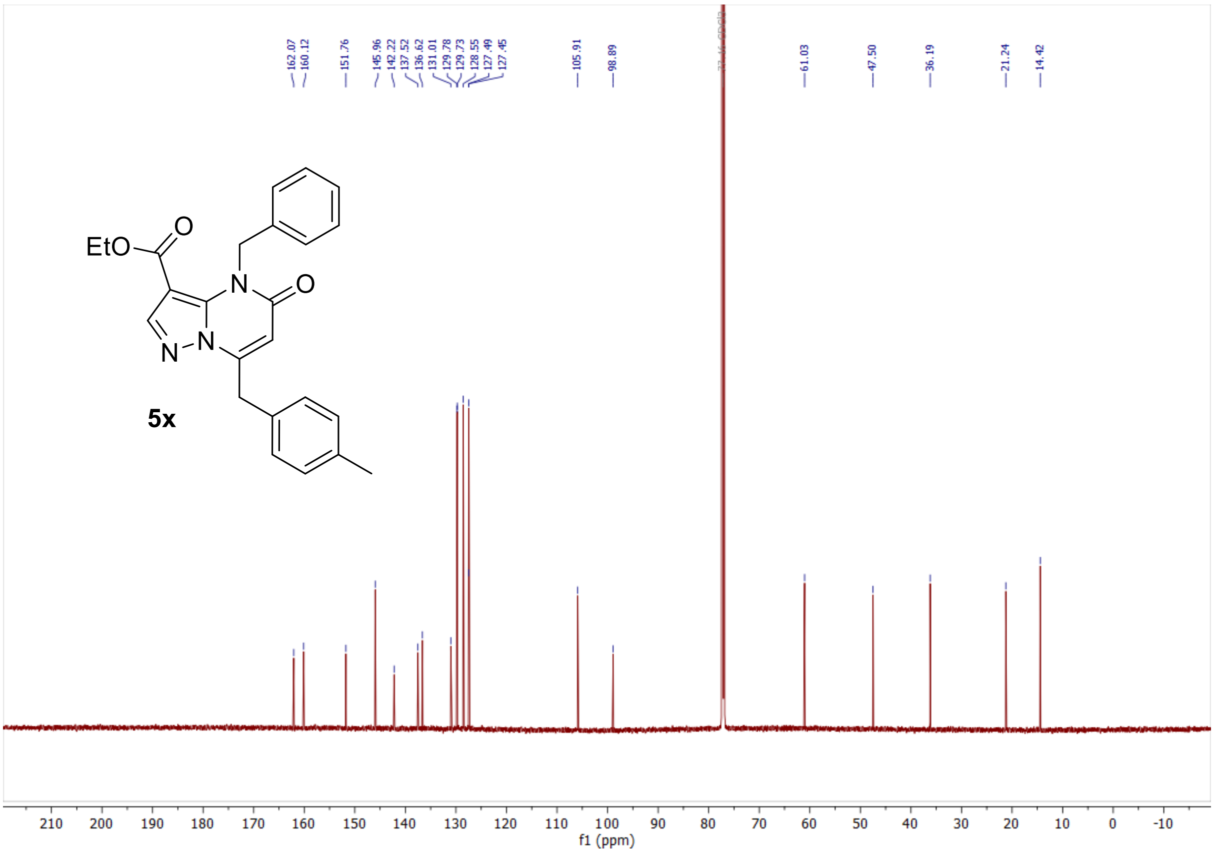

^{1}H NMR (600 MHz, $CDCl_3$) (6)

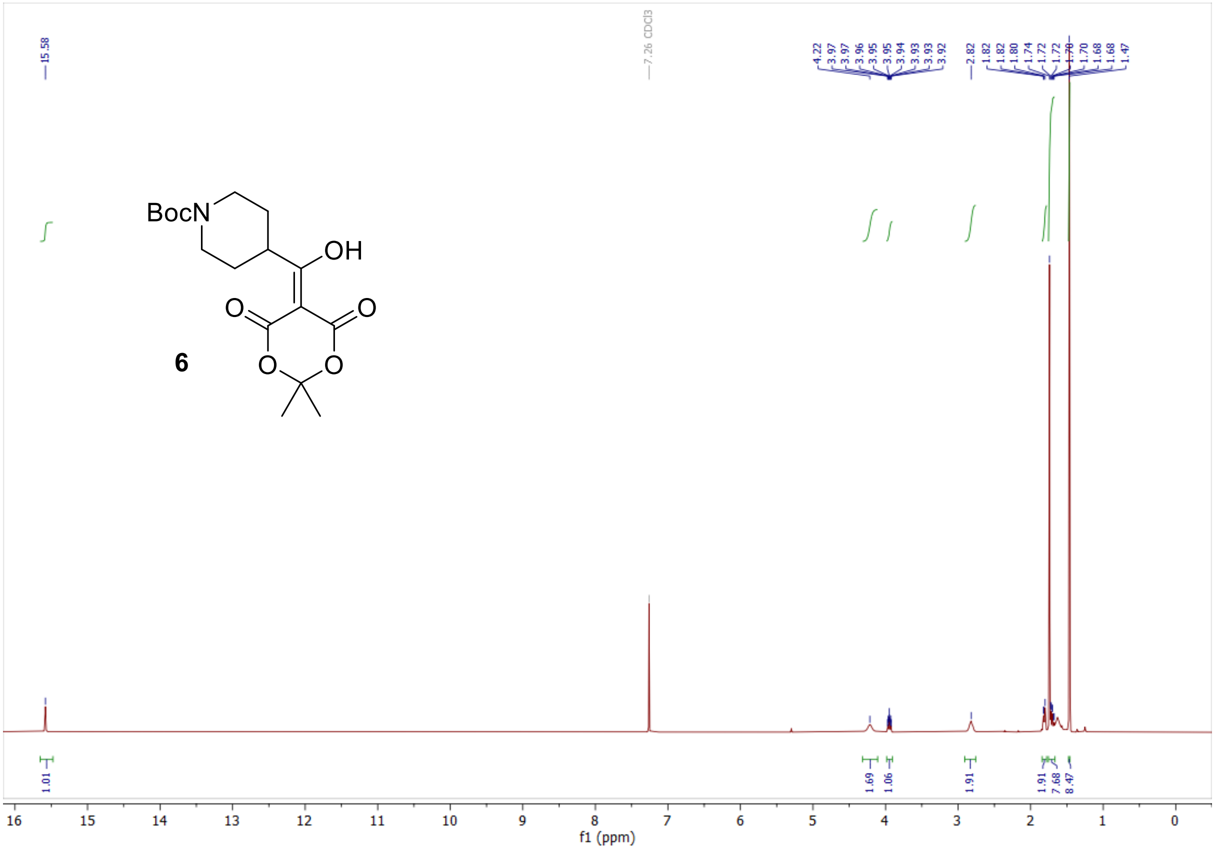

^{13}C NMR (151 MHz, $CDCl_3$) (6)

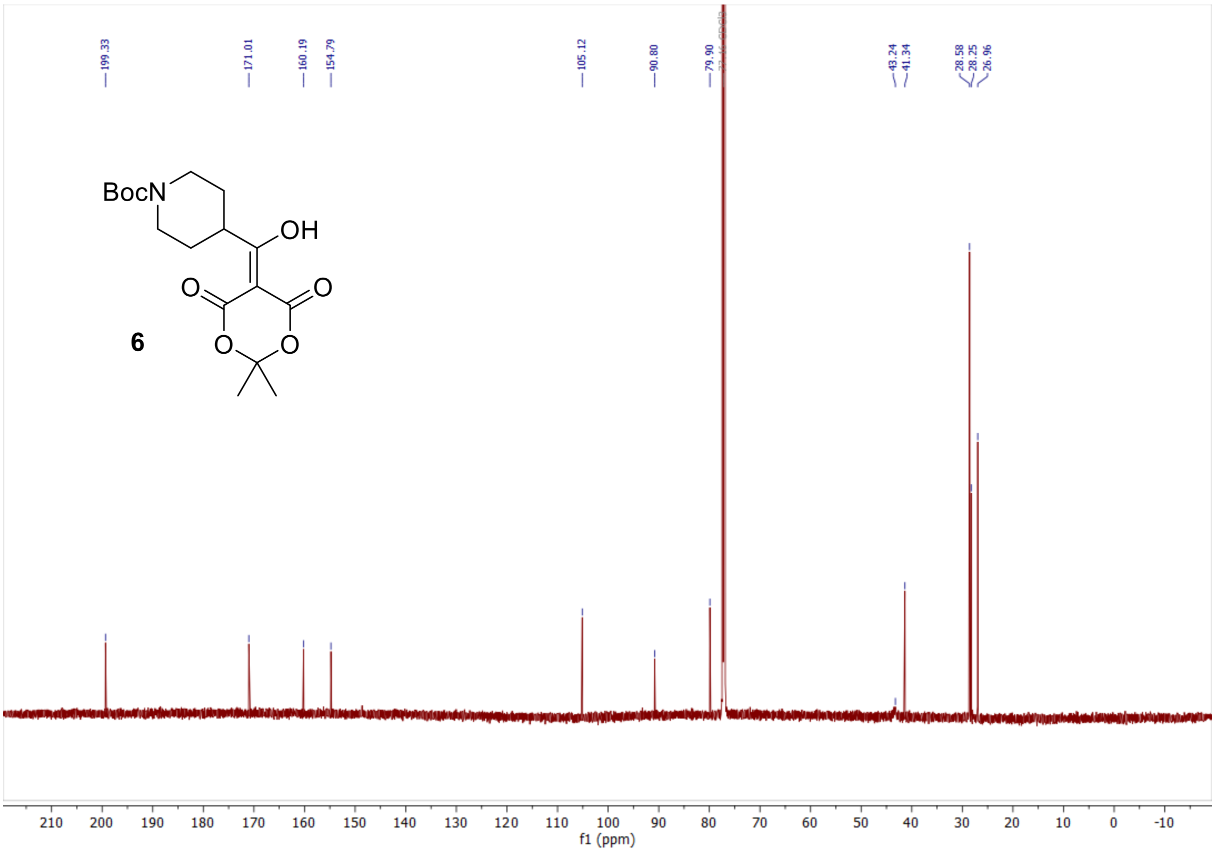

^{1}H NMR (600 MHz, D_2O) (7)

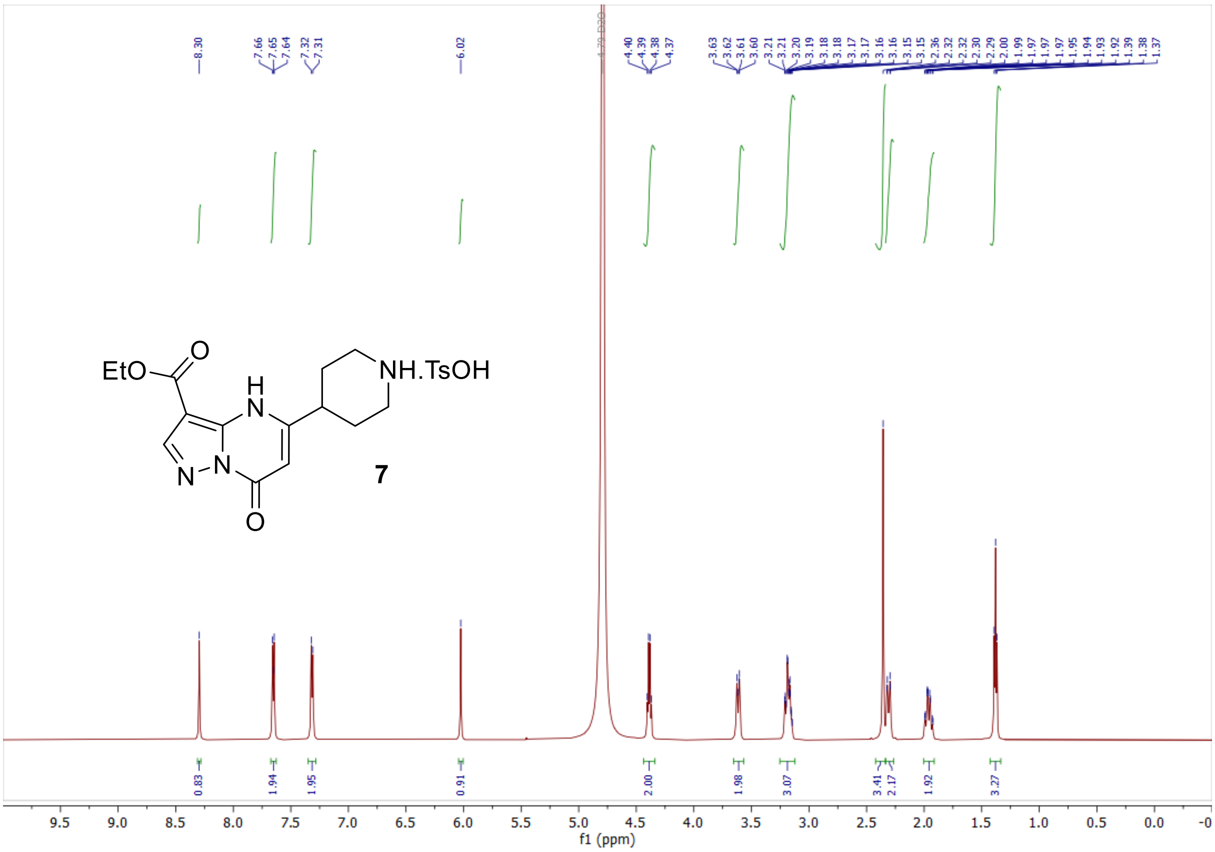

^{13}C NMR (151 MHz, D_2O) (7)

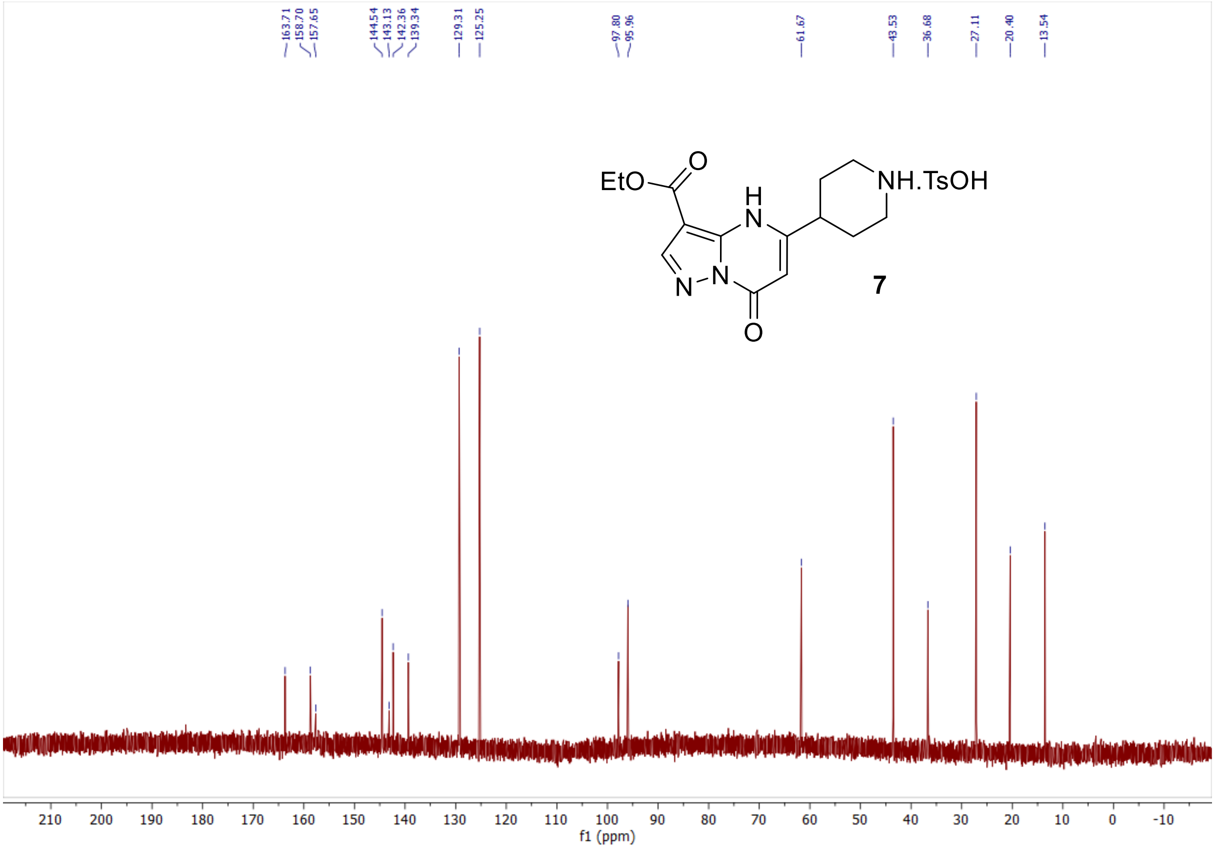

^{1}H NMR (600 MHz, D_2O) (9)

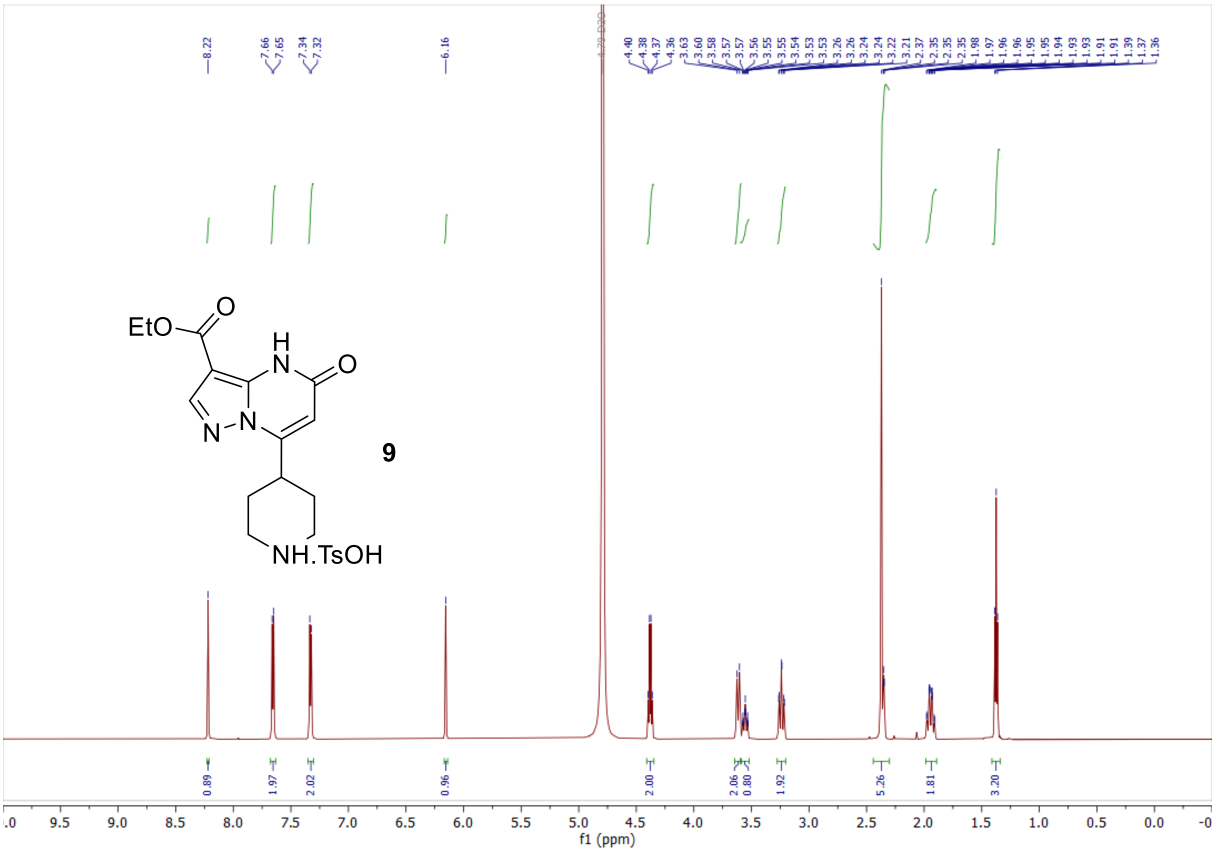

^{13}C NMR (151 MHz, D_2O) (9)

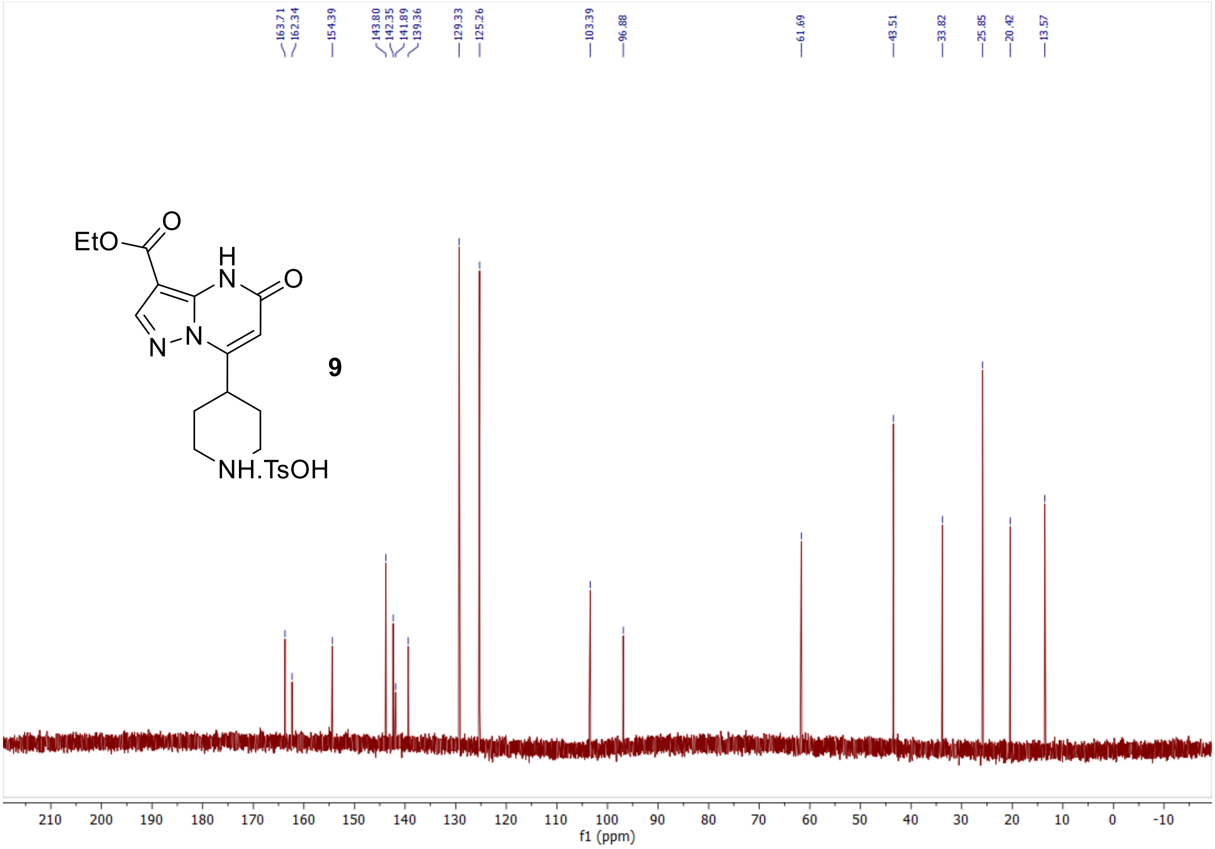

^{1}H NMR (400 MHz, $CDCl_3$) (10)

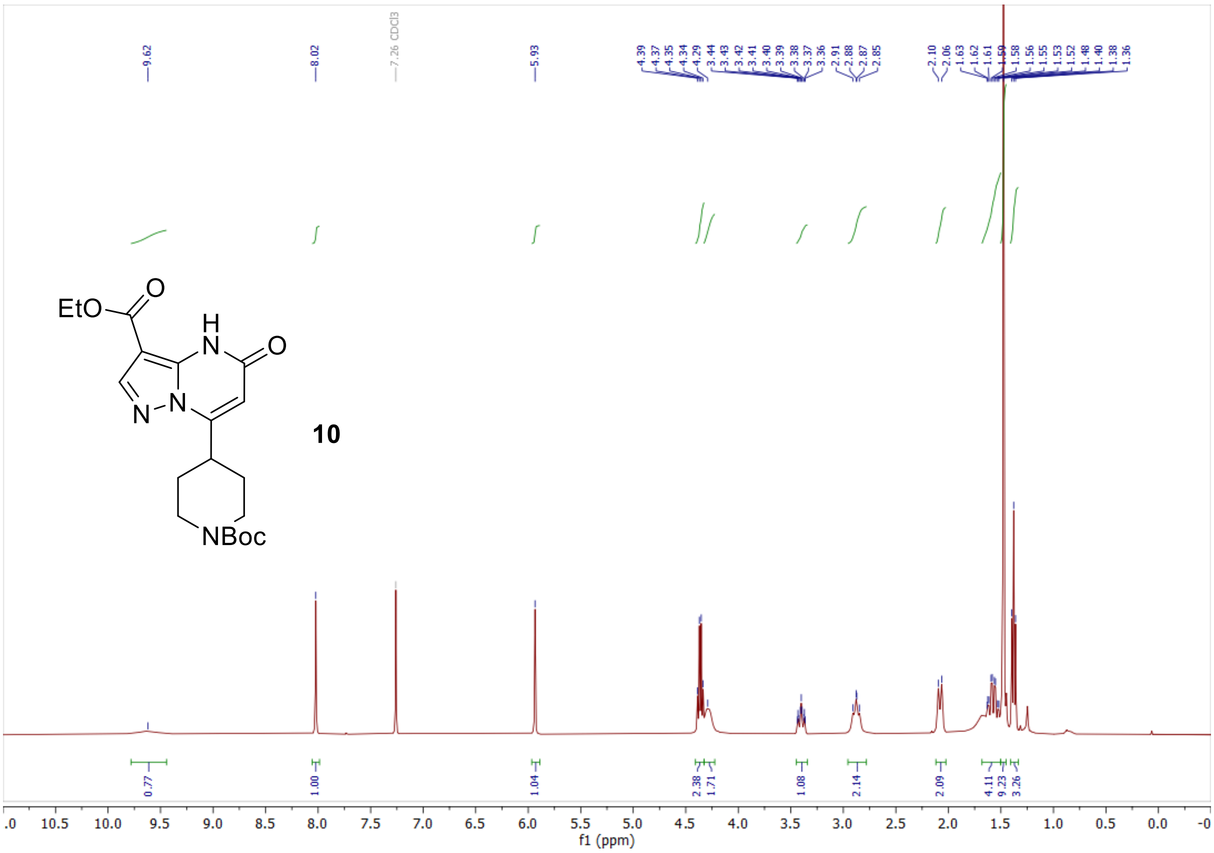

^{13}C NMR (151 MHz, $CDCl_3$) (10)

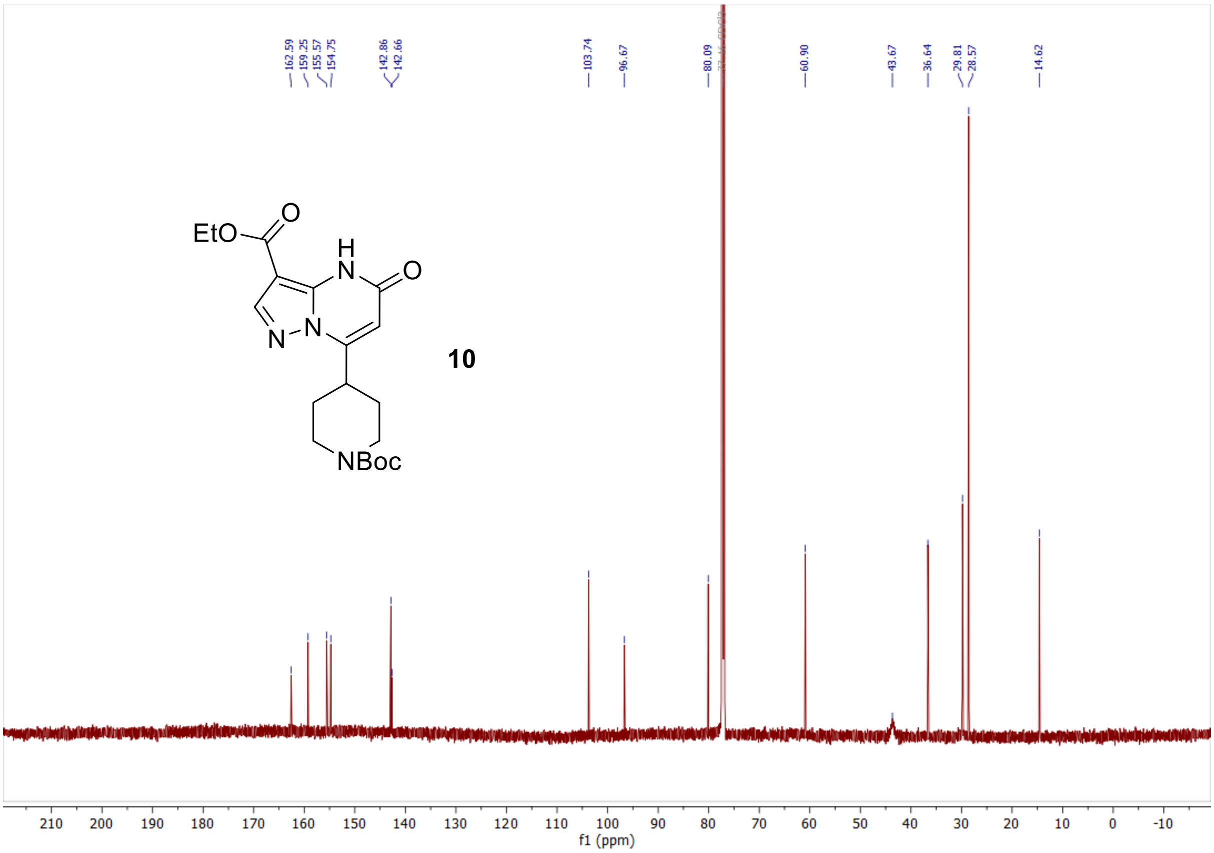

^{1}H NMR (400 MHz, $CDCl_3$) (11)

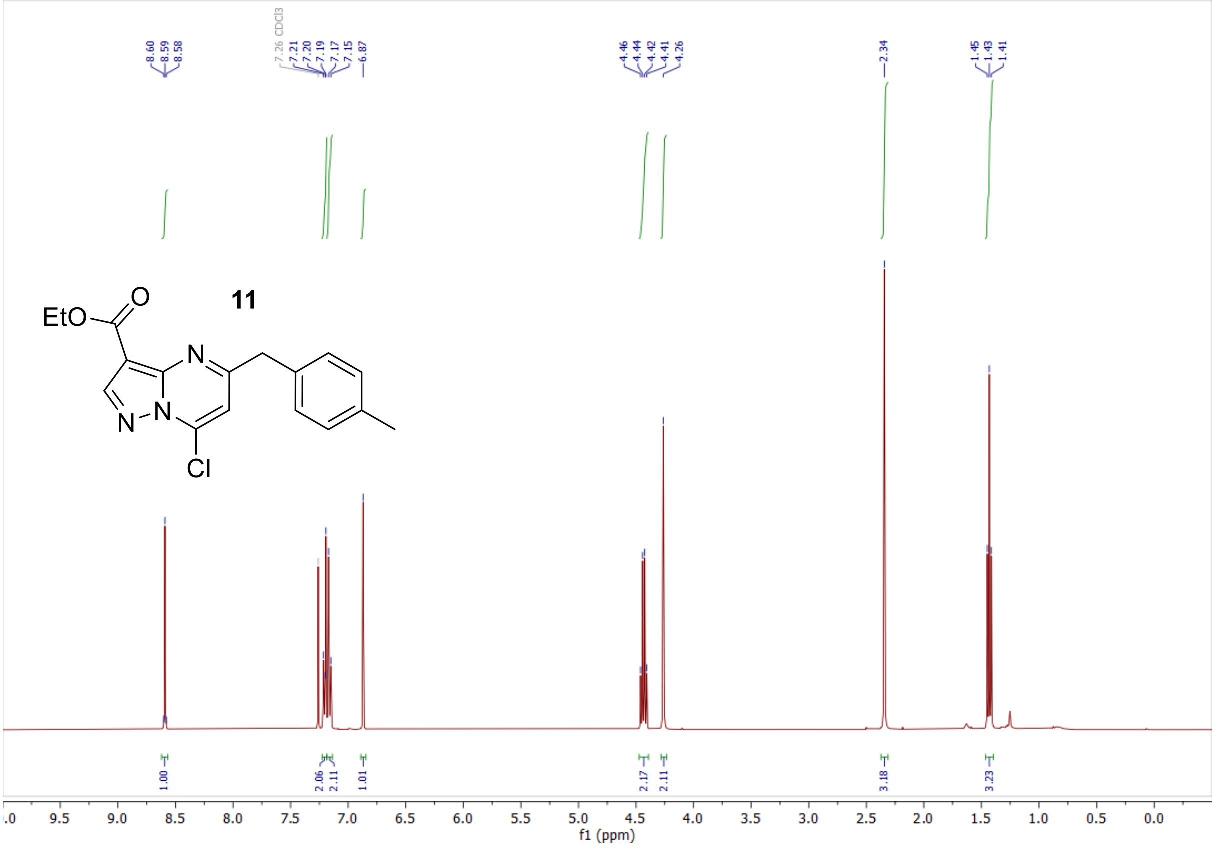

^{13}C NMR (100 MHz, $CDCl_3$) (11)

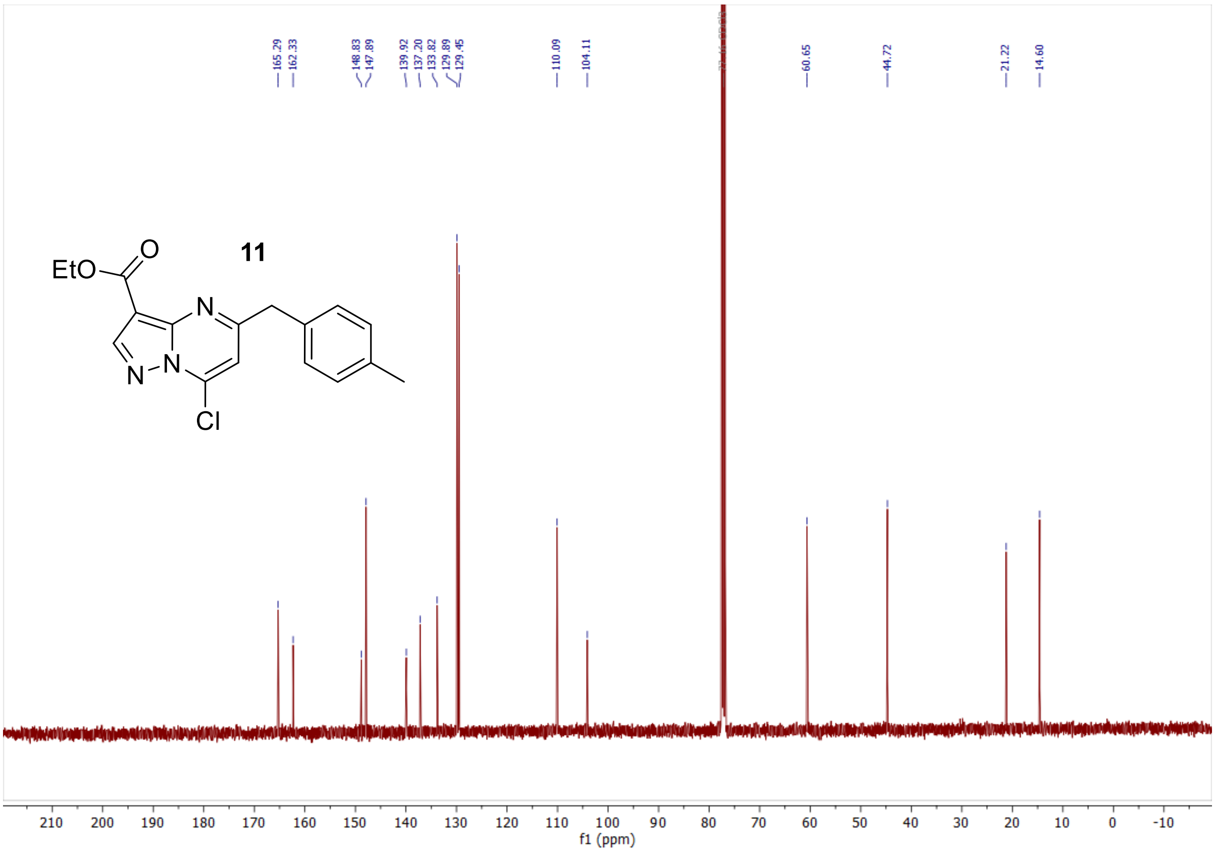

^{1}H NMR (400 MHz, $CDCl_3$) (12)

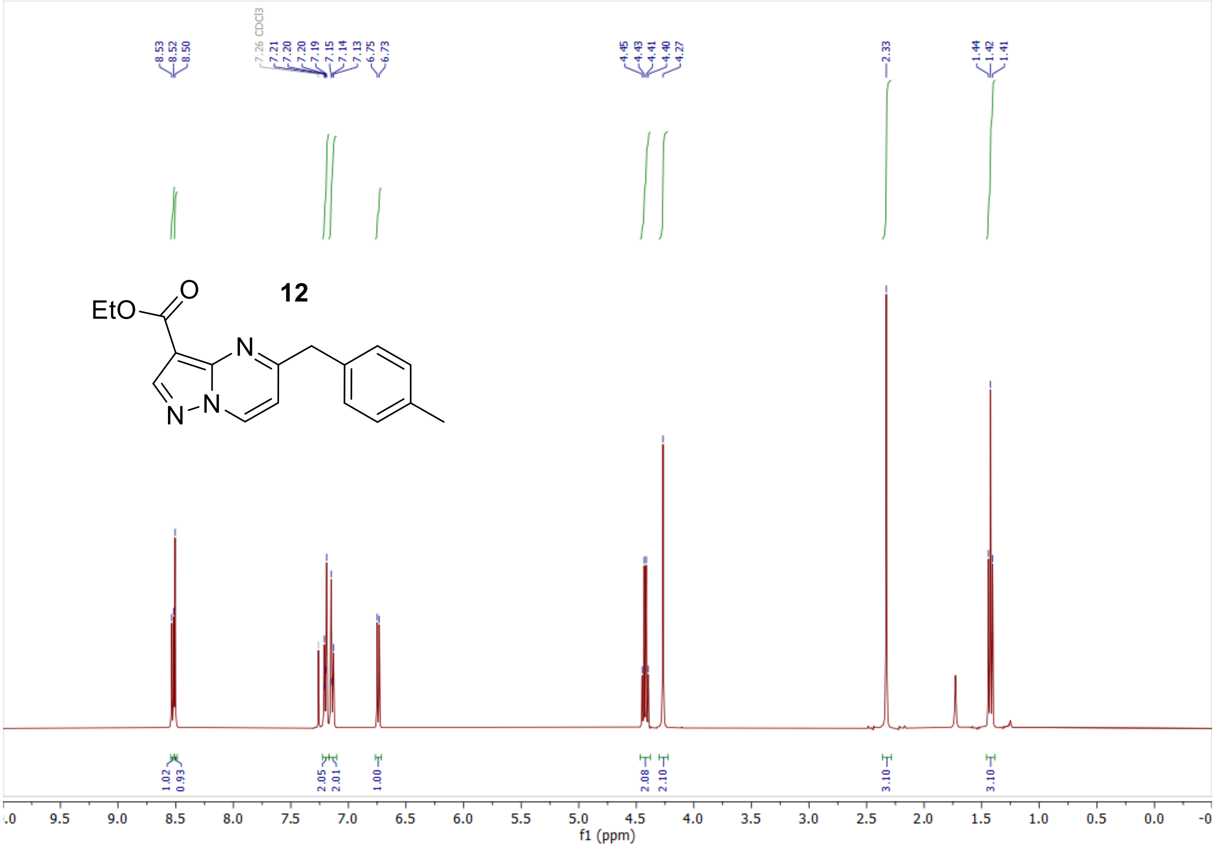

^{13}C NMR (100 MHz, $CDCl_3$) (12)

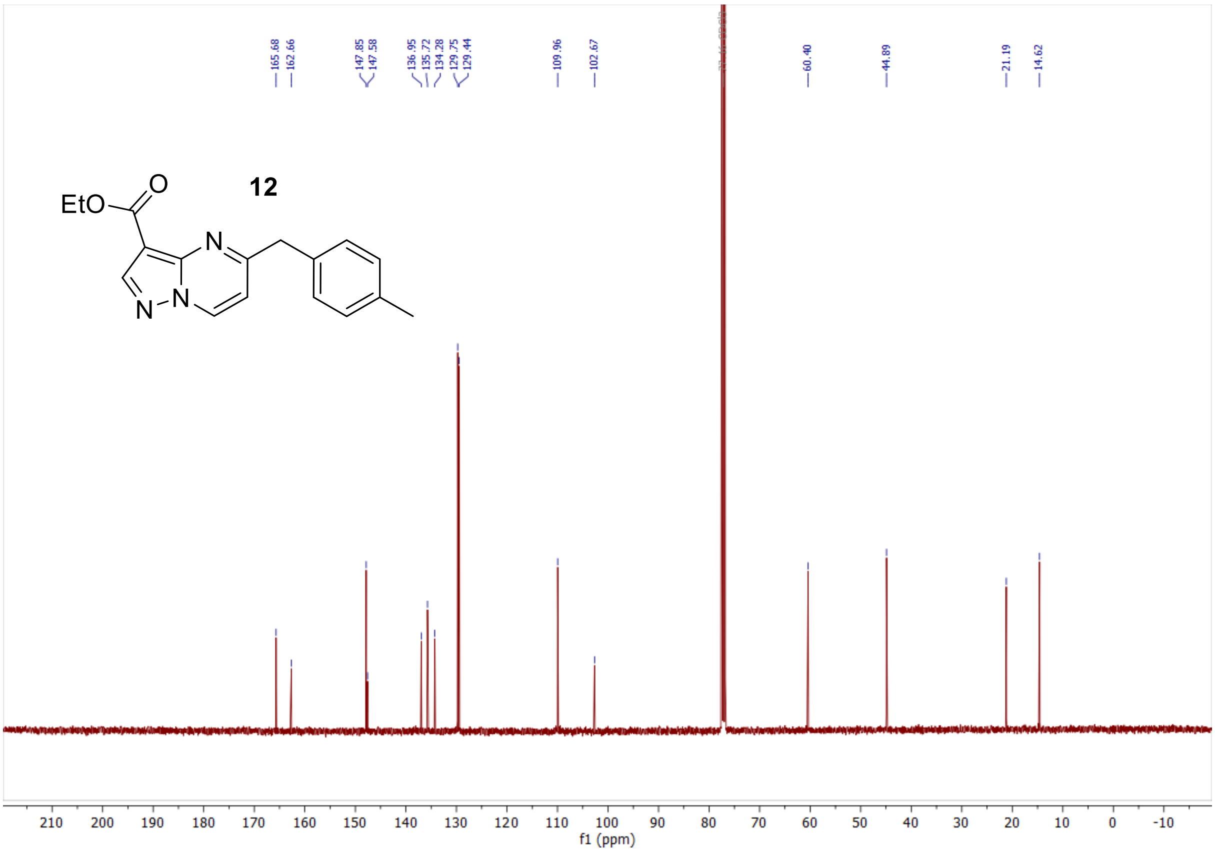

^{1}H NMR (400 MHz, $CDCl_3$) (13)

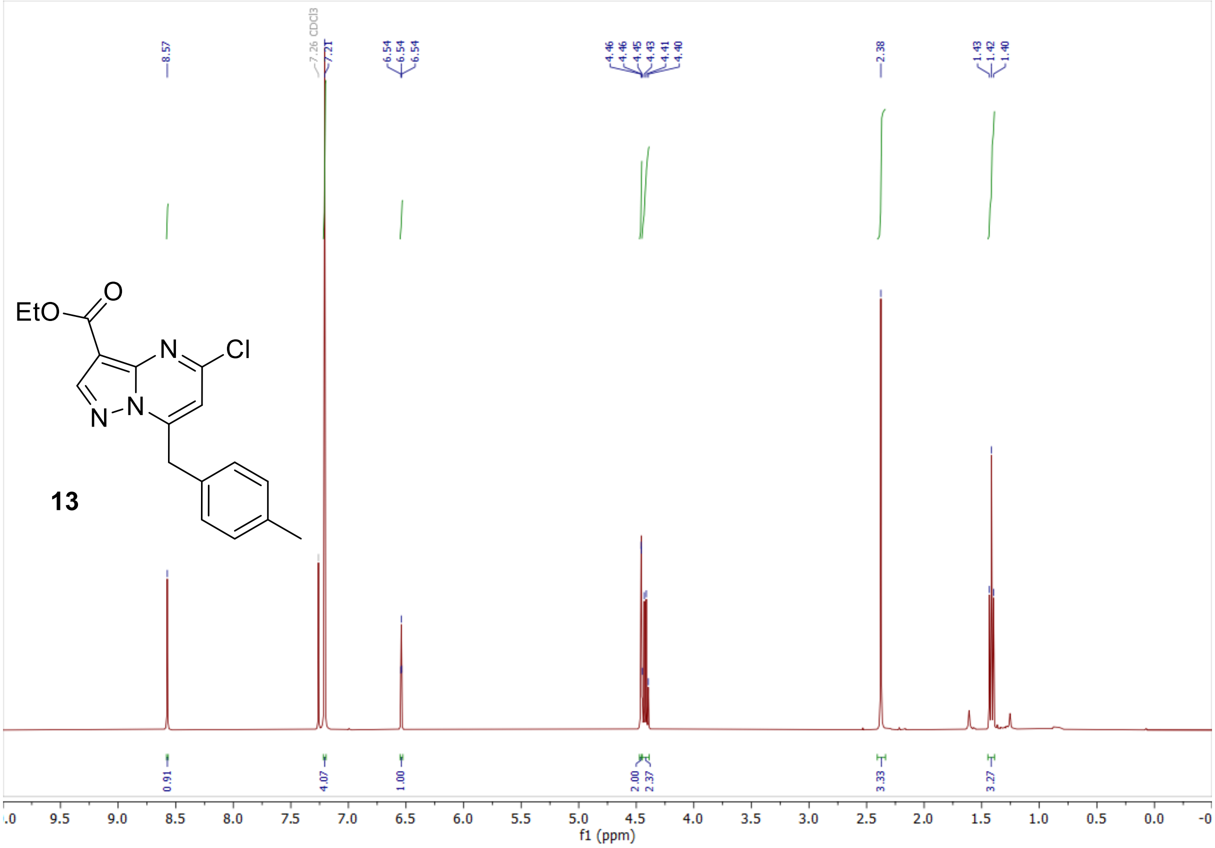

^{13}C NMR (100 MHz, $CDCl_3$) (13)

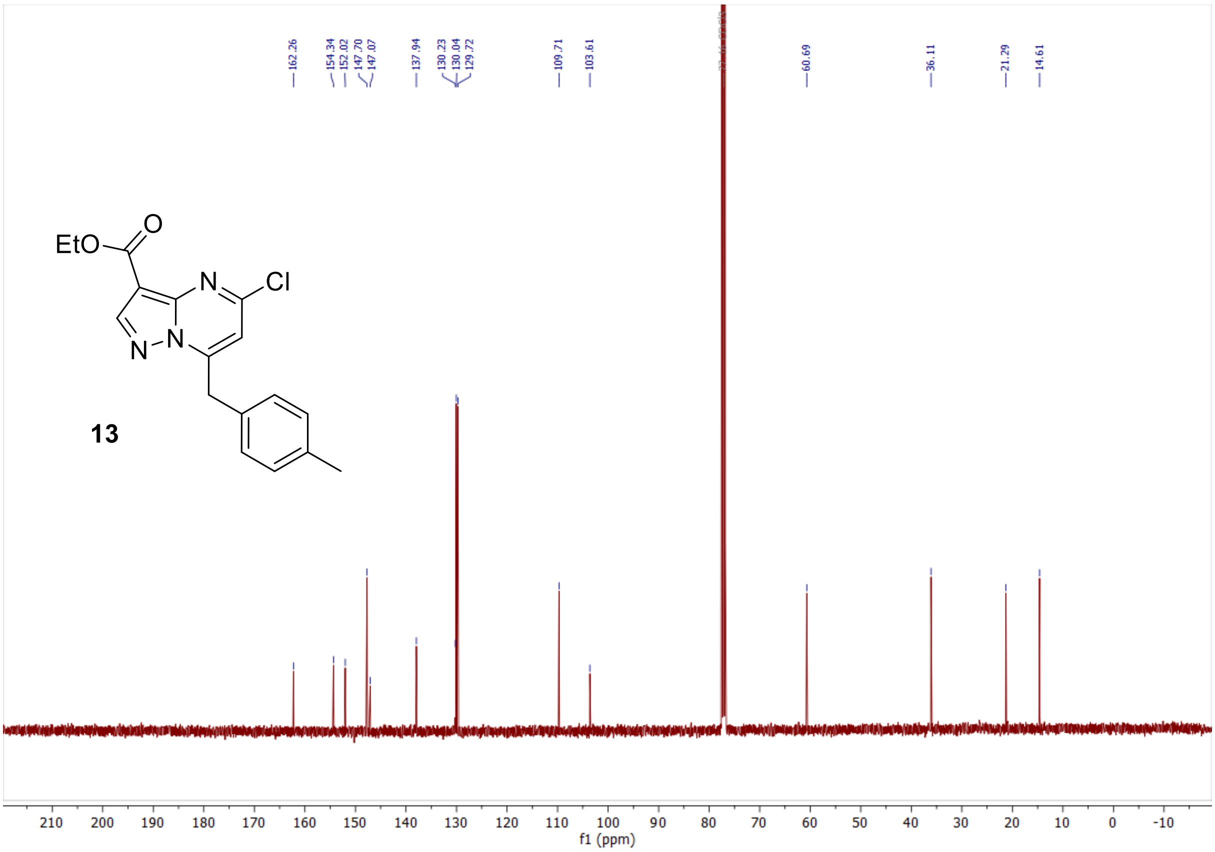

^{1}H NMR (400 MHz, CDCl$_3$) (14)

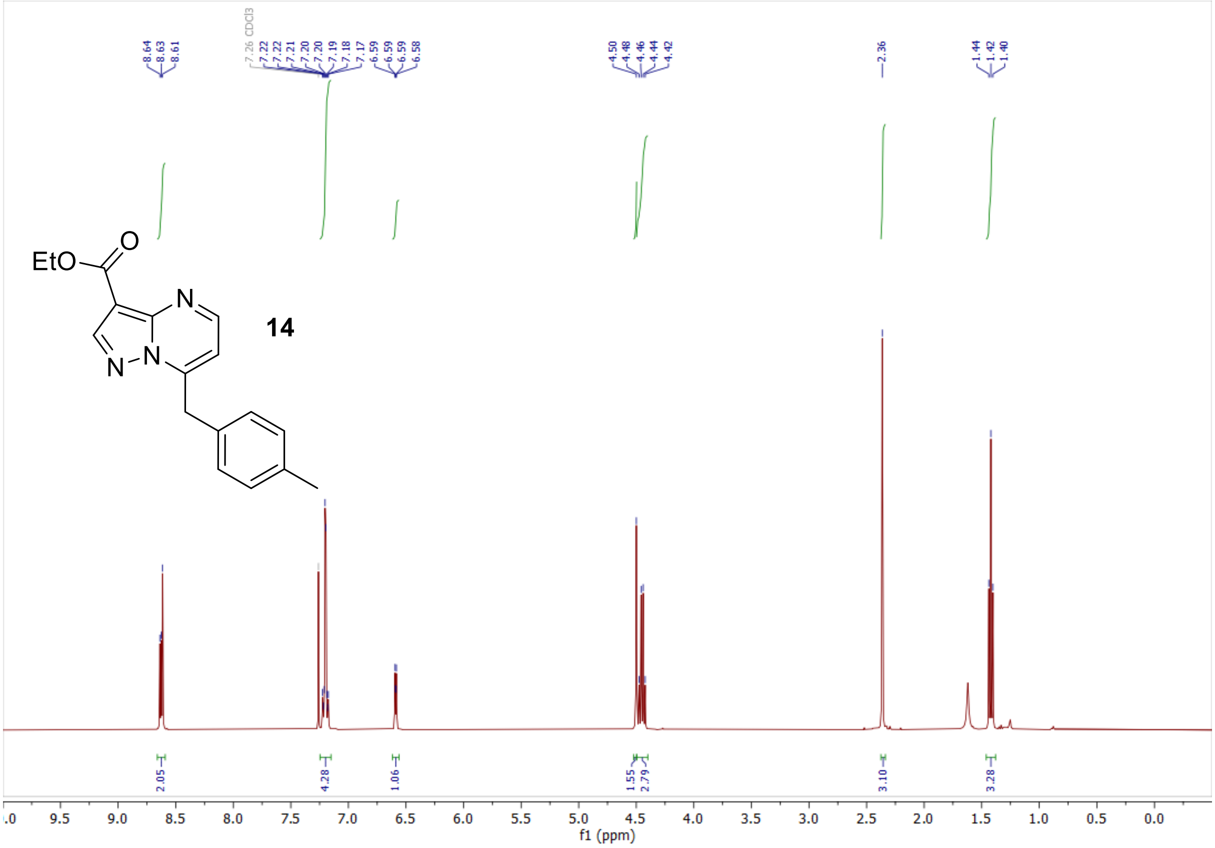

^{13}C NMR (100 MHz, CDCl$_3$) (14)

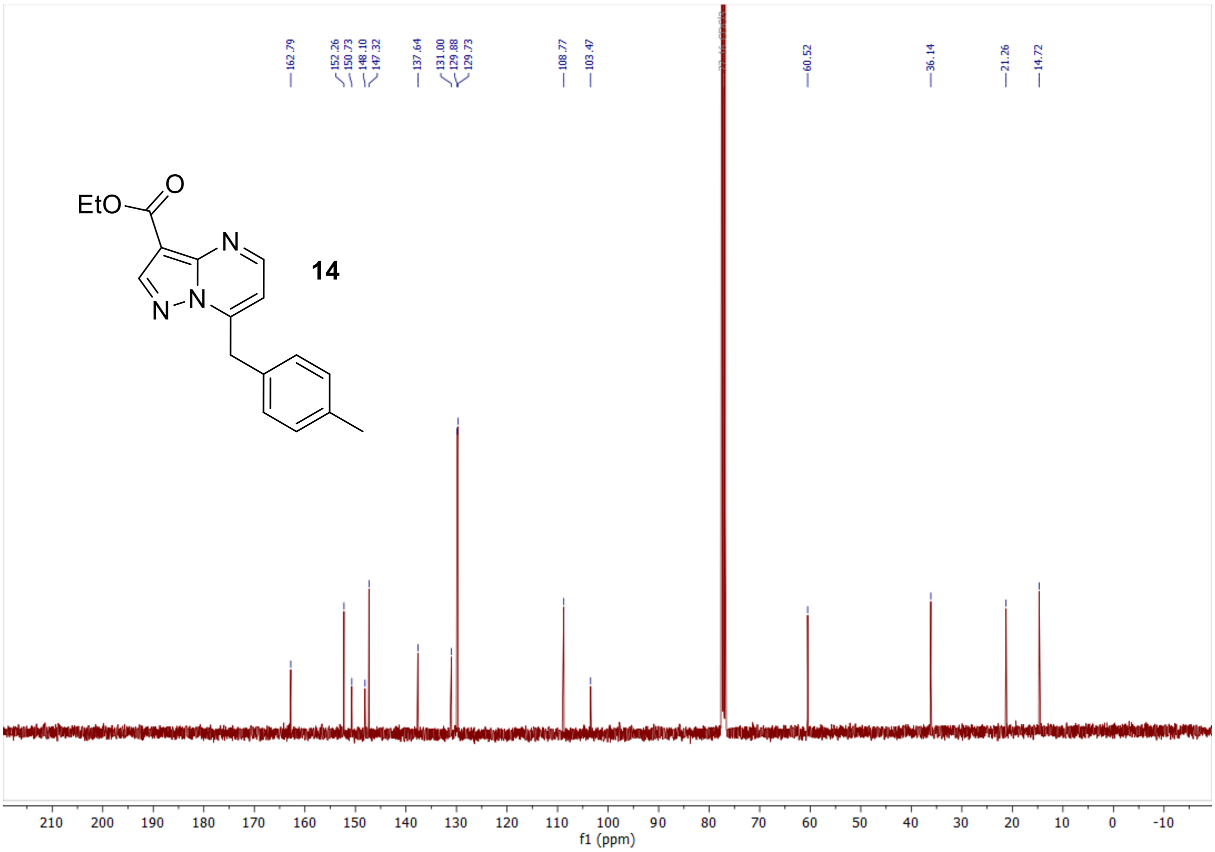

^{1}H NMR (600 MHz, $CDCl_3$) (2a')

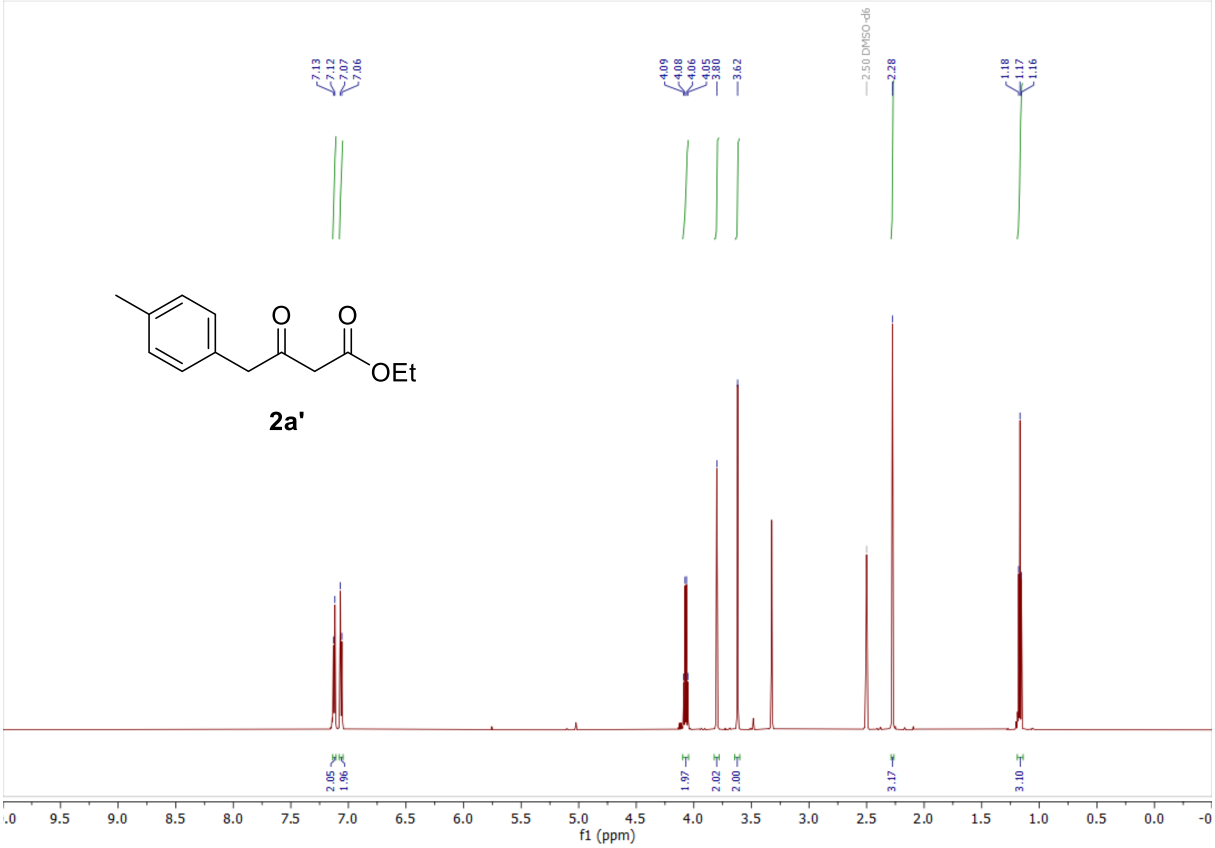

^{13}C NMR (151 MHz, $CDCl_3$) (2a')

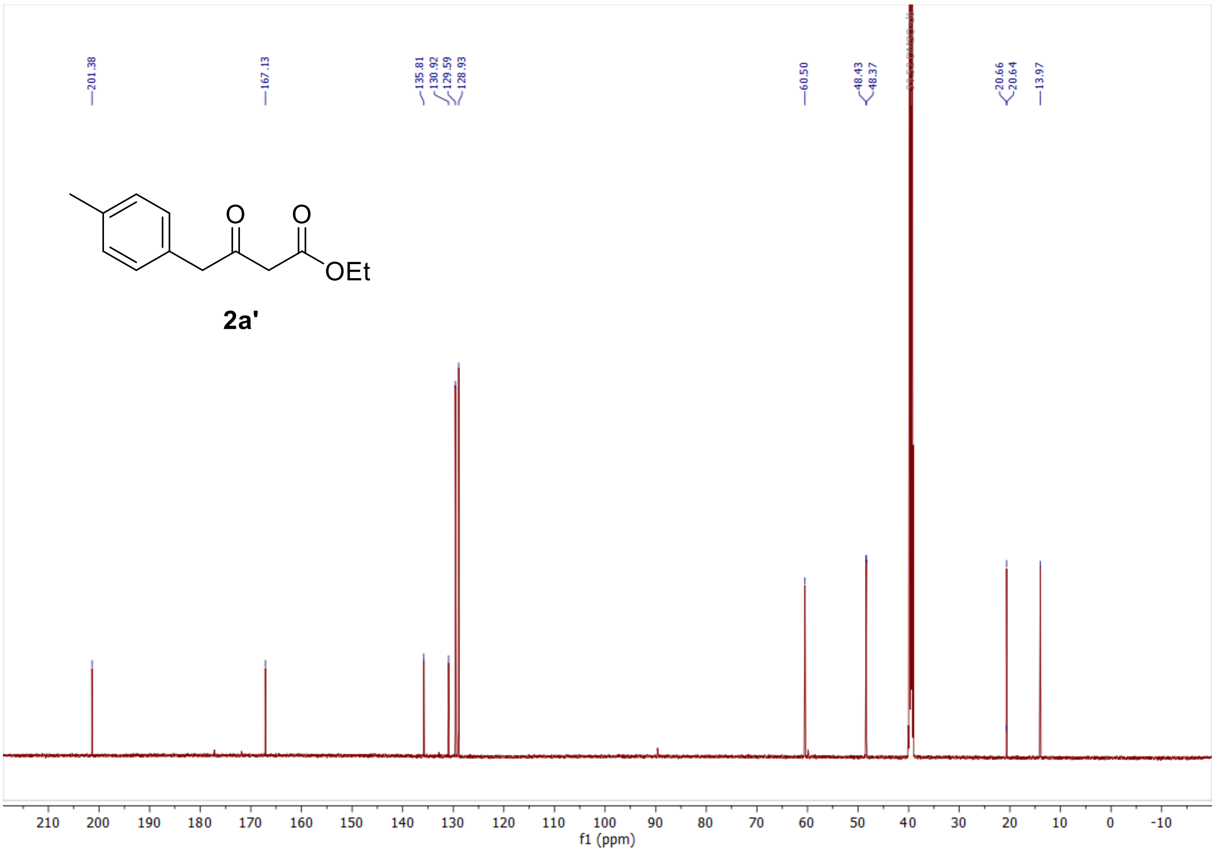

^{1}H NMR (600 MHz, $CDCl_3$) (4a)

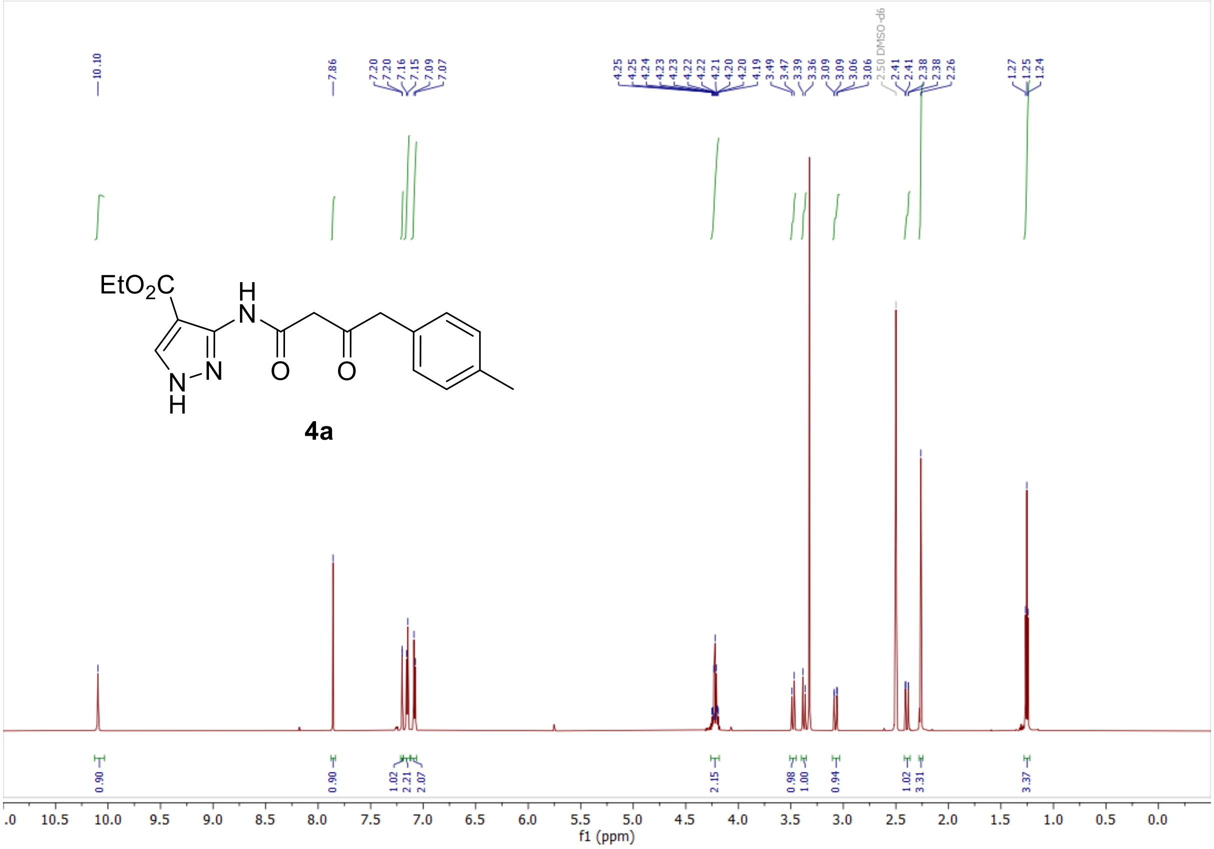

^{13}C NMR (151 MHz, $CDCl_3$) (4a)

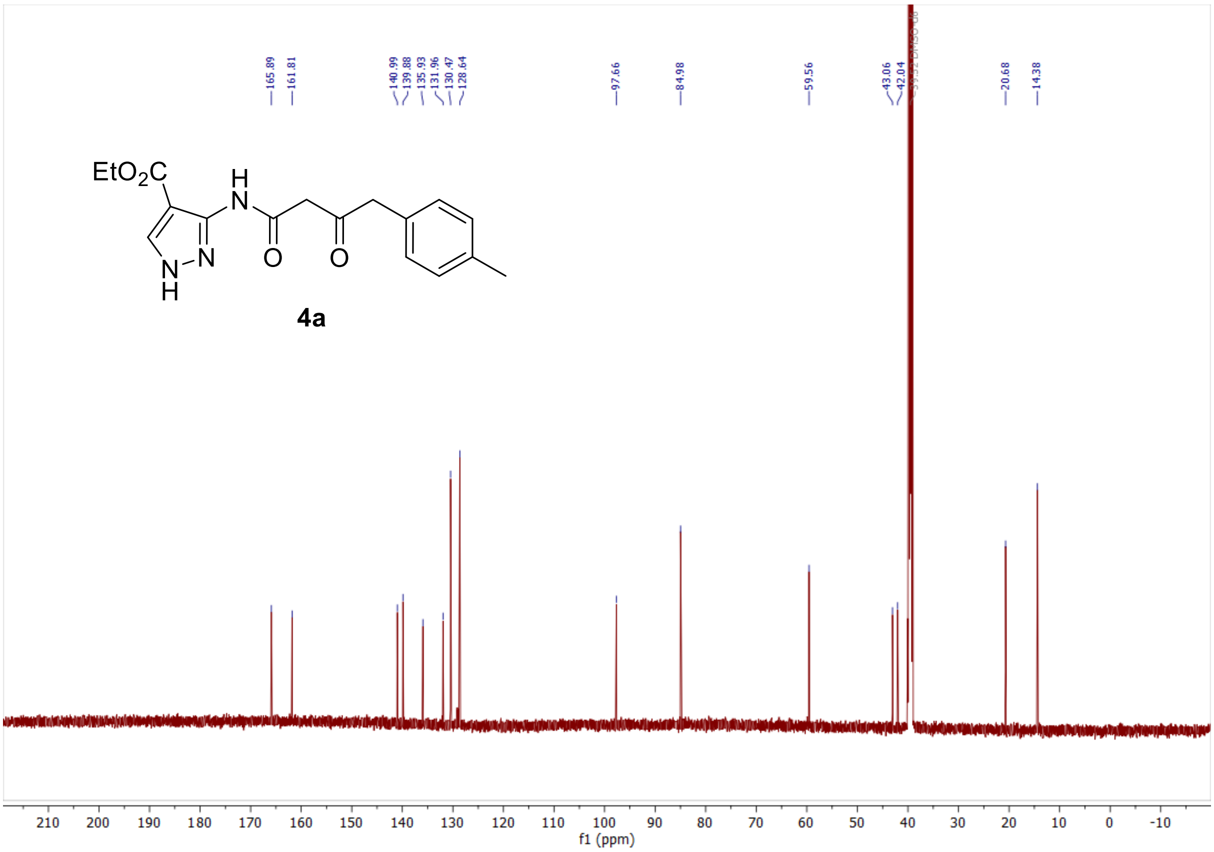

References

(1) Reber, S.; Blumer, N.; Leuenberger, D.; Fleischer, T.; Renneberg, D.; Abele, S.; Schäfer, G. Scalable Synthesis of C5aR1 Antagonist ACT-1014-6470 via N7-Selective Reductive Amination of an Unprotected Pyrazole Starting Material and Intramolecular Urea Formation with 1,1′-Carbonyl-di(1,2,4-triazol) (CDT). *Org. Process Res. Dev.* **2024**, *28* (6), 2269–2283. DOI: 10.1021/acs.oprd.3c00492.

(2) Åberg, V.; Norman, F.; Chorell, E.; Westermark, A.; Olofsson, A.; Sauer-Eriksson, A. E.; Almqvist, F. Microwave-Assisted Decarboxylation of Bicyclic 2-Pyridone Scaffolds and Identification of Aβ-Peptide Aggregation Inhibitors. *Org. Biomol. Chem.* **2005,** *3,* 2817–2823. DOI: 10.1039/B503294F.

(3) Bharate, J. B.; Ådén, J.; Gharibyan, A.; Adolfsson, D. E.; Jayaweera, S. W.; Singh, P.; Vielfort, K.; Tyagi, M.; Bonde, M.; Bergström, S.; Olofsson, A.; Almqvist, F. $K_2S_2O_8$-Mediated Coupling of 6-Amino-7-Aminomethyl-Thiazolino-Pyridones with Aldehydes to Construct Amyloid-Affecting Pyrimidine-Fused Thiazolino-2-Pyridones. *Org. Biomol. Chem.* **2021,** *19,* 9758–9772. DOI: 10.1039/d1ob01580j.

(4) Yamamoto, Y.; Watanabe, Y.; Ohnishi, S. 1,3-Oxazines and Related Compounds. XIII. Reaction of Acyl Meldrum's Acids with Schiff Bases Giving 2,3-Disubstituted 5-Acyl-3,4,5,6-tetrahydro-2*H*-1,3-oxazine-4,6-diones and 2,3,6-Trisubstituted 2,3-Dihydro-1,3-oxazin-4-ones. *Chem. Pharm. Bull.* **1987**, *35* (5), 1860–1870. DOI: 10.1248/cpb.35.1860.

(5) Harsanyi, A.; Lückener, A.; Pasztor, H.; Yilmaz, Z.; Tam, L.; Yufit, D. S.; Sandford, G. α-Fluorotricarbonyl Derivatives as Versatile Fluorinated Building Blocks: Synthesis of Fluoroacetophenone, Fluoroketo Ester and Fluoropyran-4-one Derivatives. *Eur. J. Org. Chem.* **2020**, *3872–3878*. DOI: 10.1002/ejoc.202000503.

(6) Brosge, F.; Kochs, J. F.; Bregu, M.; Truong, K.-N.; Rissanen, K.; Bolm, C. 5-Carbonyl-1,3-oxazine-2,4-diones from N-Cyanosulfoximines and Meldrum's Acid Derivatives. *Org. Lett.* **2020**, *22*, 6667–6670. DOI: 10.1021/acs.orglett.0c02504.

(7) Trost, B. M.; Papillon, J. P. N.; Nussbaumer, T. Ru-Catalyzed Alkene–Alkyne Coupling. Total Synthesis of Amphidinolide P. *J. Am. Chem. Soc.* **2005,** *127,* 17921–17937. DOI: 10.1021/ja055967n.

(8) Sørensen, U. S.; Falch, E.; Krogsgaard-Larsen, P. A Novel Route to 5-Substituted 3-Isoxazolols: Cyclization of *N*,*O*-DiBoc β-Keto Hydroxamic Acids Synthesized via Acyl Meldrum's Acids. *J. Org. Chem.* **2000,** *65,* 1003–1007. DOI: 10.1021/jo991409d.

(9) Sellstedt, M.; Nyberg, A.; Rosenbaum, E.; Engström, P.; Wickström, M.; Gullbo, J.; Bergström, S.; Johansson, L. B.-Å.; Almqvist, F. Synthesis and Characterization of a Multi Ring-Fused 2-Pyridone-Based Fluorescent Scaffold. *Eur. J. Org. Chem.* **2010,** 6171–6178. DOI: 10.1002/ejoc.201000796.

(10) Knoth, T.; Warburg, K.; Katzka, C.; Rai, A.; Wolf, A.; Brockmeyer, A.; Janning, P.; Reubold, T. F.; Eschenburg, S.; Manstein, D. J.; Hübel, K.; Kaiser, M.; Waldmann, H. The Ras Pathway Modulator Melophlin A Targets Dynamins. *Angew. Chem. Int. Ed.* 2009, 48, 7240–7245. DOI: 10.1002/anie.200902023.

(11) Lim, B.; Lee, J.; Kim, B.; Lee, R.; Park, J.; Oh, D.-C.; Gam, J.; Lee, J. Target Identification of a 1,3,4-Oxadiazin-5(6H)-One Anticancer Agent via Photoaffinity Labelling. *Asian J. Org. Chem.* **2019**, *8*, 1626–1630. DOI: 10.1002/ajoc.201900258.

(12) He, Y.-C.; Pan, J.-G. Meso-piperidine Calix[4]pyrrole: Synthesis, Structure and Ion Binding Studies. *Tetrahedron* **2015**, *71*, 8208–8212. DOI: 10.1016/j.tet.2015.08.032.

(13) Krámos, B.; Hadady, Z.; Makó, A.; Szántó, G.; Felföldi, N.; Magdó, I.; Bobok, A. Á.; Bata, I.; Román, V.; Visegrády, A.; Keserű, G. M.; Greiner, I.; Éles, J. *Novel-Type GABAB PAMs: Structure–Activity Relationship in Light of the Protein Structure. ACS Med. Chem. Lett.* **2024**, *15*, 396–405. DOI: 10.1021/acsmedchemlett.3c00560.

(14) Terai, T.; Kikuchi, K.; Iwasawa, S.; Kawabe, T.; Hirata, Y.; Urano, Y.; Nagano, T. Modulation of Luminescence Intensity of Lanthanide Complexes by Photoinduced Electron Transfer and Its Application to a Long-Lived Protease Probe. *J. Am. Chem. Soc.* **2006**, *128*, 6938–6946. DOI: 10.1021/ja060729t.
